# Large language models and bariatric surgery patient education: a comparative readability analysis of GPT-3.5, GPT-4, Bard, and online institutional resources

**DOI:** 10.1007/s00464-024-10720-2

**Published:** 2024-03-12

**Authors:** Nitin Srinivasan, Jamil S. Samaan, Nithya D. Rajeev, Mmerobasi U. Kanu, Yee Hui Yeo, Kamran Samakar

**Affiliations:** 1 Division of Upper GI and General Surgery, Keck School of Medicine of USC, 1510 San Pablo St HCC 3, Los Angeles, CA 90033 USA; 2 Karsh Division of Gastroenterology and Hepatology, Department of Medicine, Cedars-Sinai Medical Center, Los Angeles, CA USA

**Keywords:** GPT-3.5, GPT-4, Bard, Large language models, Bariatric surgery, Readability

## Abstract

**Background:**

The readability of online bariatric surgery patient education materials (PEMs) often surpasses the recommended 6th grade level. Large language models (LLMs), like ChatGPT and Bard, have the potential to revolutionize PEM delivery. We aimed to evaluate the readability of PEMs produced by U.S. medical institutions compared to LLMs, as well as the ability of LLMs to simplify their responses.

**Methods:**

Responses to frequently asked questions (FAQs) related to bariatric surgery were gathered from top-ranked health institutions. FAQ responses were also generated from GPT-3.5, GPT-4, and Bard. LLMs were then prompted to improve the readability of their initial responses. The readability of institutional responses, initial LLM responses, and simplified LLM responses were graded using validated readability formulas. Accuracy and comprehensiveness of initial and simplified LLM responses were also compared.

**Results:**

Responses to 66 FAQs were included. All institutional and initial LLM responses had poor readability, with average reading levels ranging from 9th grade to college graduate. Simplified responses from LLMs had significantly improved readability, with reading levels ranging from 6th grade to college freshman. When comparing simplified LLM responses, GPT-4 responses demonstrated the highest readability, with reading levels ranging from 6th to 9th grade. Accuracy was similar between initial and simplified responses from all LLMs. Comprehensiveness was similar between initial and simplified responses from GPT-3.5 and GPT-4. However, 34.8% of Bard's simplified responses were graded as less comprehensive compared to initial.

**Conclusion:**

Our study highlights the efficacy of LLMs in enhancing the readability of bariatric surgery PEMs. GPT-4 outperformed other models, generating simplified PEMs from 6th to 9th grade reading levels. Unlike GPT-3.5 and GPT-4, Bard’s simplified responses were graded as less comprehensive. We advocate for future studies examining the potential role of LLMs as dynamic and personalized sources of PEMs for diverse patient populations of all literacy levels.

**Supplementary Information:**

The online version contains supplementary material available at 10.1007/s00464-024-10720-2.

Bariatric surgery is an effective long-term treatment option for severe obesity and has been shown to significantly lower the risk of cardiovascular disease, malignancy, and other obesity-related comorbidities [1–4]. Despite its proven efficacy and safety, bariatric surgery is underutilized, with less than 1% of eligible patients undergoing the procedure [5]. Several factors, such as socioeconomic barriers, access to care, general perceptions of bariatric surgery, and notably low health literacy, may contribute to its underutilization [5, 6]. Furthermore, health literacy has been demonstrated to significantly impact both the utilization and outcomes of bariatric surgery [7–9].

The internet has become an essential medium for individuals seeking health information, as evidenced by a 2009 Pew Center survey revealing that 61% of U.S. adults searched for medical information online [10]. Specifically concerning bariatric surgery, studies show that approximately 50% [11] to 75% [12] of individuals considering weight loss surgery consult online resources. This information may impact patients’ decision to undergo surgery, with one study showing that 25% of patients decided on surgery mainly based on online information [12]. Furthermore, a significant number of patients continue to utilize the internet postoperatively for information [12]. Therefore, access to high-quality, easy-to-understand online patient education materials (PEMs) may be a promising intervention for improving utilization rates of bariatric surgery, as well as optimizing surgical outcomes.

Easy-to-understand PEMs are critical to ensuring comprehension among patients of all educational levels. The American Medical Association (AMA) has notably recommended that PEMs is written at the 6th grade reading level or lower [13]. However, previous studies have shown low readability among available online PEMs across multiple specialties, including bariatric surgery [14–19]. Consequently, the lack of readable PEMs may act as a barrier to patients who seek high-quality information from reliable sources, especially to those with low health literacy.

The advent and widespread adoption of large language models (LLMs) has the potential to revolutionize patient education and increase access to information across all fields of medicine. ChatGPT and Bard are two common LLMs used today that have gained unprecedented adoption by the public [20, 21]. These models were trained on a large dataset that helps them respond to queries in a comprehensible and conversational manner. There is a growing body of literature demonstrating the impressive ability of these models to answer clinical questions related to many fields of medicine, including bariatric surgery [22–24]. A recent study highlighted ChatGPT’s ability to answer bariatric surgery-related questions, where the model provided comprehensive responses to 86.8% (131/151) of questions with 90.7% reproducibility [24].

While the knowledge base of ChatGPT in bariatric surgery may be impressive, there are currently no studies examining the readability of content produced by LLMs related to bariatric surgery compared to available online PEMs. Thus, we examined the readability of PEMs produced by top-ranked medical institutions in the United States compared to PEMs produced by LLMs. Furthermore, we assessed the ability of LLMs to simplify their language in real time, as well as investigated the adaptability of LLMs to user-reading grade level.

## Materials and methods

### FAQ and institutional response curation

**Fig. 1:**
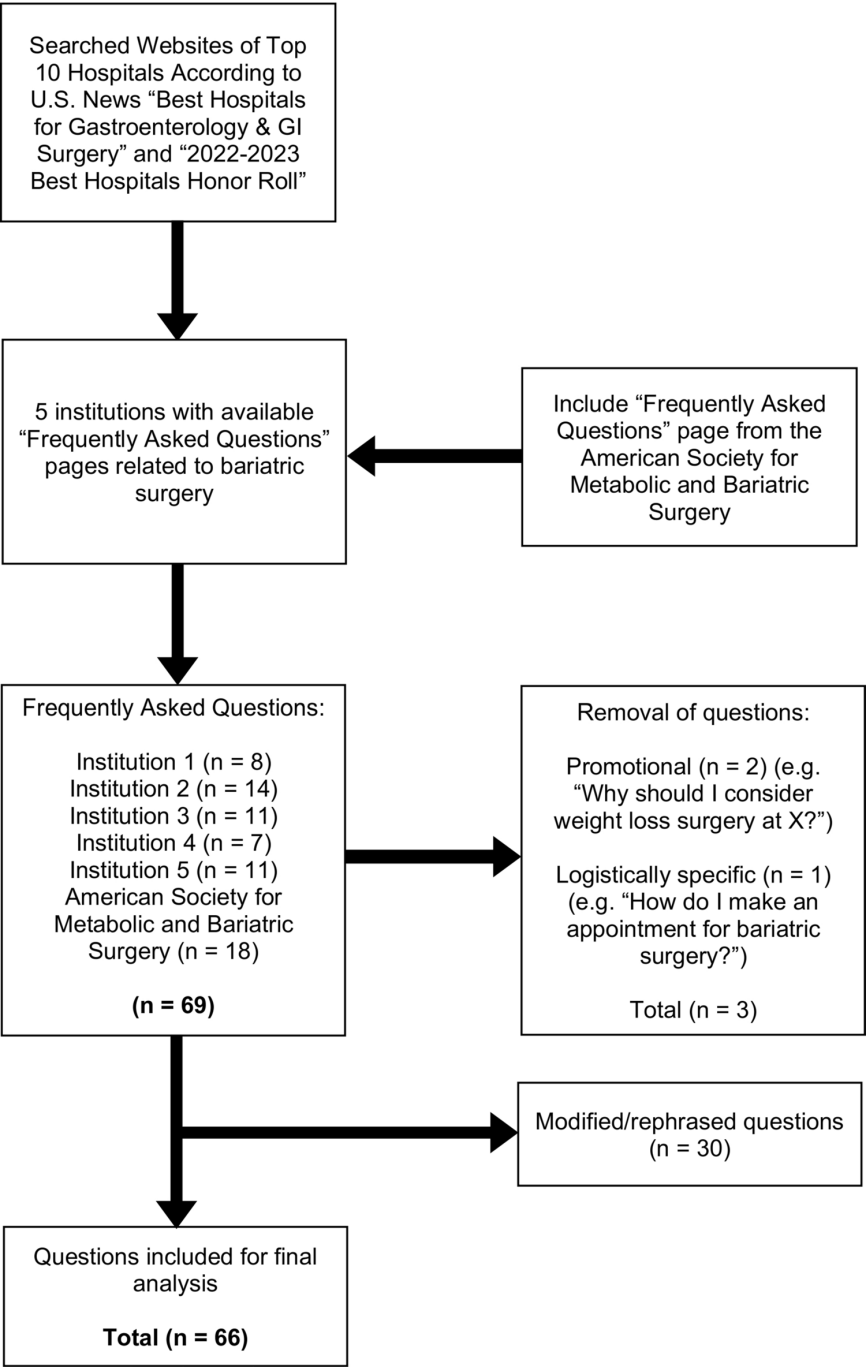
Flow chart illustrating bariatric surgery frequently asked questions, curation, screening, and selection

The Frequently Asked Questions (FAQ) pages of the American Society for Metabolic and Bariatric Surgery (ASMBS), top 10 hospitals listed in the U.S. News Best Hospitals for Gastroenterology & GI Surgery [25] and top 10 hospitals listed in the U.S. News 2022-2023 Best Hospitals Honor Roll [26] were reviewed. Questions were curated, screened, and approved by three authors (N.S., J.S., N.R.) to evaluate their inclusion in the study. Only questions related to bariatric surgery or weight loss surgery were included. Questions that were promotional (e.g., “Why should I consider weight loss surgery at [X institution]?”) or related to logistics (e.g., “How do I make an appointment for bariatric surgery [at X institution]?”) were excluded (Fig. 1). Questions that were vague were rephrased or grammatically modified to eliminate ambiguity (Fig. 1). Duplicate FAQs (i.e., the same FAQ asked by multiple institutions) were preserved in order to analyze the readability of their respective institutions’ individual answers.

### ChatGPT and Bard

ChatGPT and Bard are LLMs that have been trained on extensive datasets from various sources, including websites, literature, and articles [27, 28]. Training data for ChatGPT are limited by information up to September 2021 [27, 29], while Bard does not have a fixed knowledge cutoff year [28]. GPT-3.5 was released in November of 2022; its successor, GPT-4, was released in March 2023 and is believed to have superior performance across multiple metrics [30]. Bard was also released in March 2023 [28]. When prompted with inquiries, these models can provide well-formulated, conversational, and easy-to-understand responses. The models were refined using reinforcement learning from human feedback (RLHF) to adhere to a wide range of commands and written instructions, with human preferences serving as a reward signal to fine-tune their responses [31, 32]. These models were also trained to align with user intentions and minimize bias or harmful responses. The specific sources of information used to train ChatGPT and Bard are not entirely known.

### LLM response generation

To generate responses, each FAQ was prompted to GPT-3.5 and GPT-4 (version May 24th, 2023), as well as Bard (version June 7th, 2023). Each individual question was inputted once using the “new chat” function. After the model generated a response, we further prompted the model to simplify its response by asking “Can you explain that in simpler terms?” in the same chat. Thus, each FAQ received two recorded responses from each LLM: an initial response and a simplified response.

### Question grading

To grade the readability of responses, we used a freely available online readability scoring tool (https://readabilityformulas.com/) that has been previously utilized in several studies [33–37]. This tool analyzes text using seven established and validated readability scoring systems: Flesch Reading Ease Formula (FRE), Gunning Fog Index (GFI), Flesch–Kincaid Grade Level (FKGL), Simplified Measure of Gobbledegook Index (SMOG), Coleman–Liau Index (CLI), Automatic Readability Index (ARI), and the Linsear Write Formula (LWF).

These scoring systems use a variety of parameters (e.g., sentence length, number of syllables, number of letters, number of words, etc.) to grade the readability of text provided. The FRE generates a score from 0 to 100 [38] (Supplementary Table 1), while the GFI [39], FKGL [40], SMOG [41], CLI [42], ARI [43], and LWF [44] generate a score corresponding to the U.S. school grade level at which an average student in that grade level can effectively read and understand the given material [45].

Across all responses, punctuation (e.g., periods, commas, exclamation points, etc.) and characters indicating that information is being presented in a list (e.g., bullet points, asterisks, dashes, numbers, etc.) were included in readability score calculations. Formatting characters, such as “**” (indicating bolded text), were excluded. For responses that contained information presented in tables, only the text and punctuation from these tables were included.

### Accuracy and comprehensiveness

A board-certified, fellowship-trained active practice academic bariatric surgeon (K.S.) compared the accuracy and comprehensiveness of initial and simplified responses for each LLM using the following scales.

When comparing accuracy:

1. The Simplified Response is more accurate than the Initial Response.
2. The Simplified Response is equal in accuracy to the Initial Response.
3. The Simplified Response is less accurate than the Initial Response.

When comparing comprehensiveness:

1. The Simplified Response is more comprehensive than the Initial Response.
2. The Simplified Response is equal in comprehensiveness to the Initial Response.
3. The Simplified Response is less comprehensive than the Initial Response.

### Statistical analysis

Descriptive analyses are presented as means and standard deviations (SD). Readability scores for answers to FAQs across institutions and LLMs were compared using Student’s *t* test. A *p* value less than 0.05 was considered statistically significant for all analyses. All analyses were conducted by author N.S. using Microsoft Excel (version 16.75), with statistical expertise provided by author Y.Y.

Institutional Review Board approval and written consent were not required for this study.

## Results

Five institutions that contained bariatric surgery FAQ pages on their websites were included in our study. In combination with the ASMBS website, [46] we gathered a total of 69 FAQs; three questions were excluded, leaving 66 FAQs to be included in the study (Fig. 1, Supplementary Tables 2, 3 and 4). Individual readability scores associated with each institution (anonymized) and the ASMBS are presented in Supplementary Table 5.

### Institutional and LLM responses

**Fig. 2:**
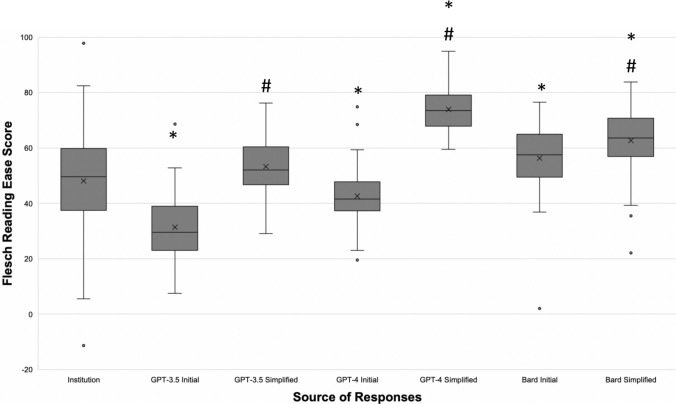
Illustration of flesch reading ease scores for institutional, initial LLM, and simplified LLM responses to bariatric surgery frequently asked questions. Box and whisker plots were constructed for institutional, initial LLM, and simplified LLM readability scores. Horizontal line inside the box represents the median, and an *X* represents the mean. *LLM* large language model; *p < 0.05 when compared to institutional scores; ^#^*p* < 0.05 when comparing initial and simplified responses of the same LLM

**Fig. 3:**
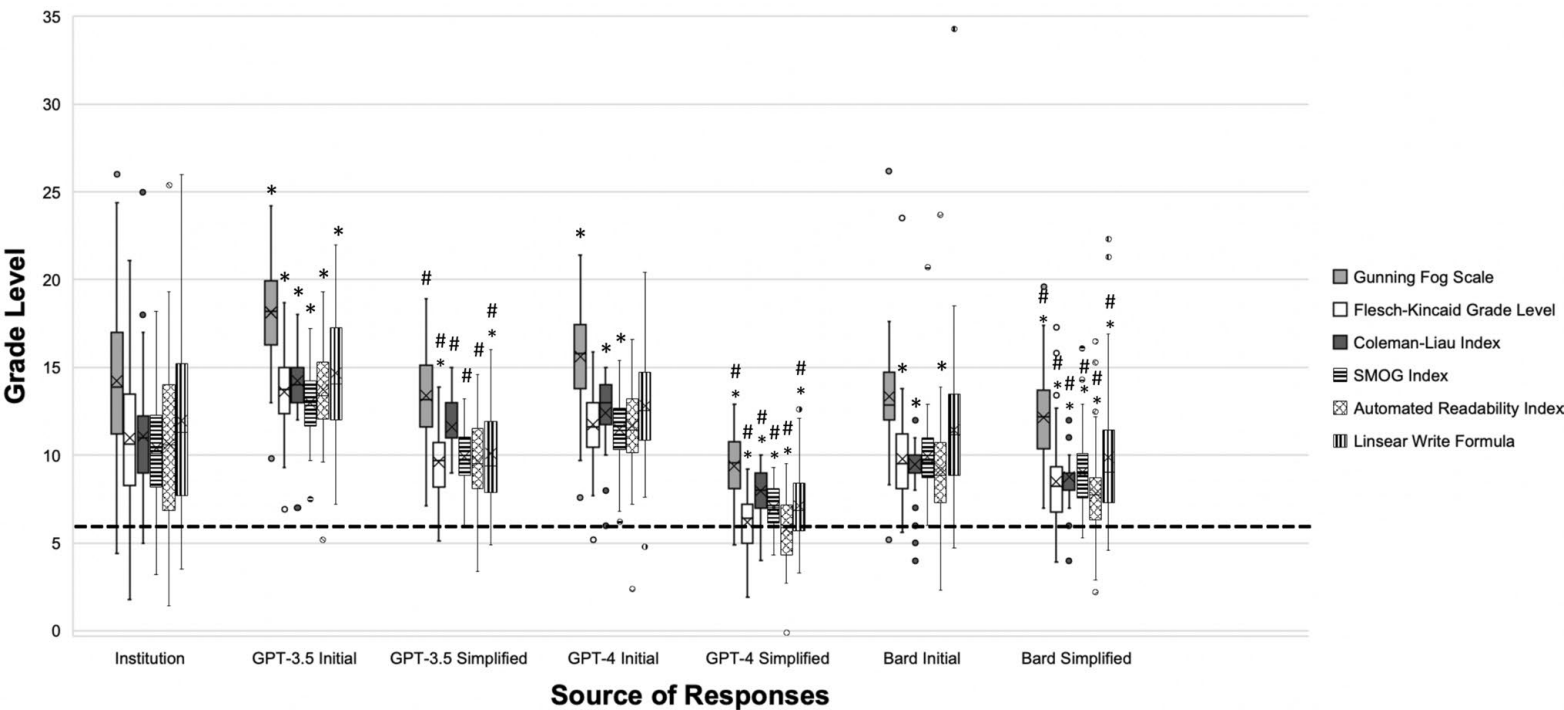
Illustration of reading grade levels measured by the Gunning Fog Scale, Flesch–Kincaid Grade Level, Coleman–Liau Index, SMOG Index, Automated Readability Index, and Linsear Write Formula, for Institutional, Initial LLM and Simplified LLM Responses to Bariatric Surgery Frequently Asked Questions. Box and whisker plots were constructed for institutional, initial LLM, and simplified LLM readability scores. Horizontal line inside the box represents the median, and an *X* represents the mean. *LLM* large language model; *p < 0.05 when compared to institutional scores; ^#^*p* < 0.05 when comparing initial and simplified responses of the same LLM; “---”: American Medical Association-recommended grade level for patient educational materials (6th grade)

The mean FRE score of institutional responses was 48.1 (SD = 19.0), which corresponded to “difficult to read,” while initial responses from GPT-3.5, GPT-4.0 and Bard achieved mean scores of 31.4 (SD = 11.4), 42.7 (SD=9.7), and 56.3 (SD = 11.6), which corresponded to “difficult to read,” “difficult to read,” and “fairly difficult to read,” respectively (Table 1, Fig. 2). When examined by grade level, institutional response readability ranged from 10th grade to college sophomore (Table 2, Fig. 3). On the other hand, readability of initial LLM responses ranged from college freshman to college graduate for GPT-3.5, 12th grade to college senior for GPT-4, and 9th grade to college freshman for Bard (Table 2, Fig. 3).

**Table 1:** Comparison of Flesch Reading Ease Scores between institutional, initial LLM, and simplified LLM responses to bariatric surgery frequently asked questions

Source of responses	Mean (standard deviation)	Score interpretation
Institutions	48.1 (19.0)	College level (difficult to read)
GPT-3.5 Initial	31.4 (11.4)*	College level (difficult to read)
GPT-3.5 Simplified	53.2 (10.7)	10th to 12th grade level (fairly difficult to read)
GPT-4 Initial	42.7 (9.7)*	College level (difficult to read)
GPT-4 Simplified	74.0 (7.2)*	7th Grade level (fairly easy to read)
Bard Initial	56.3 (11.6)*	10th to 12th grade level (fairly difficult to read)
Bard Simplified	62.8 (11.1)*	8th and 9th Grade level (plain English)

Score interpretation: derived from Chapter 2 of “How to Write Plain English” by Rudolf Flesch (https://web.archive.org/web/20160712094308/http://www.mang.canterbury.ac.nz/writing_guide/writing/flesch.shtml)

*LLM* large language model

*p < 0.05 when compared to institutional score

**Table 2:** Comparison of reading grade levels between institutional, initial LLM and simplified LLM responses to bariatric surgery frequently asked questions

	Grade range	Gunning Fog Score	Flesch–Kincaid Grade Level	Coleman–Liau Index	SMOG Index	Automated Readability Index	Linsear–Write Formula
Institutions	10th grade to college sophomore	14.2 (4.4)	11.0 (3.8)	11.1 (3.3)	10.4 (3.0)	10.6 (4.7)	12.0 (5.5)
GPT-3.5 Initial	College freshman to college graduate	18.1 (2.7)*	13.6 (2.3)*	14.2 (1.8)*	13.0 (1.8)*	13.8 (2.7)*	14.7 (3.4)*
GPT-3.5 Simplified	10th grade to college freshman	13.4 (2.6)	9.6 (2.0)*	11.6 (1.6)	9.9 (1.7)	9.7 (2.3)	10.1 (2.8)*
GPT-4 Initial	12th grade to college senior	15.6 (2.6)*	11.8 (2.0)	12.4 (1.6)*	11.5 (1.7)*	11.7 (2.4)	12.8 (3.3)
GPT-4 Simplified	6th grade to 9th grade	9.4 (1.9)*	6.2 (1.5)*	8.0 (1.4)*	7.0 (1.2)*	5.8 (1.9)*	7.1 (2.1)*
Bard Initial	9th grade to college freshman	13.3 (2.7)	9.8 (2.6)*	9.5 (1.6)*	9.9 (2.0)	9.2 (2.9)*	11.4 (4.1)
Bard Simplified	8th grade to 12th grade	12.1 (2.6)*	8.5 (2.4)*	8.8 (1.4)*	9.0 (2.0)*	7.8 (2.6)*	9.9 (3.5)*

All values are presented as mean (standard deviation)

*LLM* large language model

*p < 0.05 when compared to institutional scores

Simplified responses from GPT-3.5, GPT-4.0, and Bard achieved mean FRE scores of 53.2 (SD = 10.7), 74.0 (SD = 7.2), and 62.8 (SD = 11.1), which corresponded to “fairly difficult to read,” “fairly easy to read,” and “plain English,” respectively (Table 1, Fig. 2). When examined by grade level, simplified response readability ranged from 10th grade to college freshman for GPT-3.5, 6th to 9th grade for GPT-4, and 8th to 12th grade for Bard (Table 2, Fig. 3).

### Comparisons between institutions and LLMs

#### Institutions vs GPT-3.5

Initial responses from GPT-3.5 received a lower FRE score than that of institutions (*p* < 0.05) (Table 1, Fig. 2), as well as greater grade levels across all instruments (*p* < 0.05) (Table 2, Figure 3). Simplified responses, however, received a similar FRE score to that of institutions (*p* = 0.059) (Table 1, Fig. 2, Supplementary Table 6). When examining grade levels, GPT-3.5 provided simplified responses with similar readability to that of institutions across most instruments, except for FKGL (*p* < 0.05) and LWF (*p* < 0.05) (Table 2, Fig. 3).

#### Institutions vs. GPT-4

Initial responses from GPT-4 received a lower FRE score than that of institutions (*p* < 0.05) (Table 1, Fig. 2). When examining grade levels, GPT-4 provided responses with lower readability than that of institutions across most instruments (*p* < 0.05), except for FKGL (*p* = 0.142), ARI (*p* = 0.105), and LWF (*p* = 0.265) (Table 2, Fig. 3, Supplementary Table 6). Simplified responses from GPT-4 received a higher FRE than that of institutions (*p* < 0.05) (Table 1, Fig. 2), as well as lower grade levels across all instruments (*p* < 0.05) (Table 2, Fig. 3).

#### Institutions vs. Bard

Initial responses from Bard received a higher FRE score than that of institutions (*p* < 0.05) (Table 1, Fig. 2). When examining grade levels, Bard produced responses with higher readability than that of institutions across most instruments (*p* < 0.05), except for GFI (*p* = 0.160), SMOG (*p* = 0.285), and LWF (*p* = 0.543) (Table 2, Fig. 3, Supplementary Table 6). Simplified responses from Bard received a higher FRE score than that of institutions (Table 1, Fig. 2), as well as lower grade levels across all instruments (Table 2, Fig. 3) (*p* < 0.05).

### Comparisons between LLMs

Simplified responses from GPT-3.5, GPT-4, and Bard received higher FRE scores, as well as lower grade levels across all instruments, compared to those of initial responses from GPT-3.5, GPT-4, and Bard, respectively (*p* < 0.05) (Table 3, Supplementary Table 7). Initial and simplified responses from GPT-4 received higher FRE scores, as well as lower grade levels across all instruments, compared to those of initial and simplified responses from GPT-3.5, respectively (*p* < 0.05) (Table 3, Supplementary Table 7). Initial and simplified responses from GPT-4 received higher FRE scores, as well as lower grade levels across all instruments, compared to those of initial and simplified responses from Bard, respectively (*p* < 0.05) (Table 3, Supplementary Table 7).

**Table 3:** Comparison of Readability Scores between initial LLM and simplified LLM responses to bariatric surgery frequently asked questions

Comparison	Readability Test	Readability Scores
GPT-3.5 Initial vs. GPT-3.5 Simplified	Flesch Reading Ease Formula	31.4 (11.4) vs. 53.2 (10.7)*
	Gunning Fog Scale	18.1 (2.7) vs. 13.4 (2.6)*
	Flesch–Kincaid Grade Level	13.6 (2.3) vs. 9.6 (2.0)*
	Coleman–Liau Index	14.2 (1.8) vs. 11.6 (1.6)*
	SMOG Index	13.0 (1.8) vs. 9.9 (1.7)*
	Automated Readability Index	13.8 (2.7) vs. 9.7 (2.3)*
	Linsear Write Formula	14.7 (3.4) vs. 10.1 (2.8)*
GPT-4 Initial vs. GPT-4 Simplified	Flesch Reading Ease Formula	42.7 (9.7) vs. 74.0 (7.2)*
	Gunning Fog Scale	15.6 (2.6) vs. 9.4 (1.9)*
	Flesch–Kincaid Grade Level	11.8 (2.0) vs. 6.2 (1.5)*
	Coleman–Liau Index	12.4 (1.6) vs. 8.0 (1.4)*
	SMOG Index	11.5 (1.7) vs. 7.0 (1.2)*
	Automated Readability Index	11.7 (2.4) vs. 5.8 (1.9)*
	Linsear Write Formula	12.8 (3.3) vs. 7.1 (2.1)*
Bard Initial vs. Bard Simplified	Flesch Reading Ease Formula	56.3 (11.6) vs. 62.8 (11.1)*
	Gunning Fog Scale	13.3 (2.7) vs. 12.1 (2.6)*
	Flesch–Kincaid Grade Level	9.8 (2.6) vs. 8.5 (2.4)*
	Coleman–Liau Index	9.5 (1.6) vs. 8.8 (1.4)*
	SMOG Index	9.9 (2.0) vs. 9.0 (2.0)*
	Automated Readability Index	9.2 (2.9) vs. 7.8 (2.6)*
	Linsear Write Formula	11.4 (4.1) vs. 9.9 (3.5)*
GPT-3.5 Initial vs. GPT-4 Initial	Flesch Reading Ease Formula	31.4 (11.4) vs. 42.7 (9.7)*
	Gunning Fog Scale	18.1 (2.7) vs. 15.6 (2.6)*
	Flesch–Kincaid Grade Level	13.6 (2.3) vs. 11.8 (2.0)*
	Coleman–Liau Index	14.2 (1.8) vs. 12.4 (1.6)*
	SMOG Index	13.0 (1.8) vs. 11.5 (1.7)*
	Automated Readability Index	13.8 (2.7) vs. 11.7 (2.4)*
	Linsear Write Formula	14.7 (3.4) vs. 12.8 (3.3)*
GPT-3.5 Simplified vs. GPT-4 Simplified	Flesch Reading Ease Formula	53.2 (10.7) vs. 74.0 (7.2)*
	Gunning Fog Scale	13.4 (2.6) vs. 9.4 (1.9)*
	Flesch–Kincaid Grade Level	9.6 (2.0) vs. 6.2 (1.5)*
	Coleman–Liau Index	11.6 (1.6) vs. 8.0 (1.4)*
	SMOG Index	9.9 (1.7) vs. 7.0 (1.2)*
	Automated Readability Index	9.7 (2.3) vs. 5.8 (1.9)*
	Linsear Write Formula	10.1 (2.8) vs. 7.1 (2.1)*
GPT-4 Initial vs. Bard Initial	Flesch Reading Ease Formula	42.7 (9.7) vs. 56.3 (11.6)*
	Gunning Fog Scale	15.6 (2.6) vs. 13.3 (2.7)*
	Flesch–Kincaid Grade Level	11.8 (2.0) vs. 9.8 (2.6)*
	Coleman–Liau Index	12.4 (1.6) vs. 9.5 (1.6)*
	SMOG Index	11.5 (1.7) vs. 9.9 (2.0)*
	Automated Readability Index	11.7 (2.4) vs. 9.2 (2.9)*
	Linsear Write Formula	12.8 (3.3) vs. 11.4 (4.1)*
GPT-4 Simplified vs. Bard Simplified	Flesch Reading Ease Formula	74.0 (7.2) vs. 62.8 (11.1)*
	Gunning Fog Scale	9.4 (1.9) vs. 12.1 (2.6)*
	Flesch–Kincaid Grade Level	6.2 (1.5) vs. 8.5 (2.4)*
	Coleman–Liau Index	8.0 (1.4) vs. 8.8 (1.4)*
	SMOG Index	7.0 (1.2) vs. 9.0 (2.0)*
	Automated Readability Index	5.8 (1.9) vs. 7.8 (2.6)*
	Linsear Write Formula	7.1 (2.1) vs. 9.9 (3.5)*

All scores presented as mean (standard deviation)

*LLM* large language model

*p < 0.05

### Accuracy and comprehensiveness of LLM responses

The majority of simplified LLM responses were rated as equal in accuracy to initial responses. The majority of simplified GPT-3.5 and GPT-4 responses (92.4% and 92.4%, respectively) were rated as equal in comprehensiveness to initial responses. However, 34.8% of simplified Bard responses were rated as less comprehensive than initial responses (Supplementary Table 8).

## Discussion

Access to high-quality and easy-to-understand PEMs may better serve bariatric surgery patients and the public. We evaluated the readability of bariatric surgery PEMs from medical institutions compared to those generated by LLMs. We then evaluated the ability of LLMs to rephrase and improve the readability of their responses when prompted to do so. Finally, we compared the accuracy and comprehensiveness of initial and simplified LLM responses to determine the impact of simplification on content quality. Our analysis shows poor readability among all institutions as well as initial LLM responses, where average reading levels ranged from 9th grade to college graduate. When prompted to explain their initial responses in simpler terms, all LLMs generated significantly more readable text compared to their initial responses. Among all the LLMs, GPT-4 provided the most readable simplified responses, with reading levels ranging from 6th to 9th grade. Additionally, while GPT-4 and GPT-3.5 maintained high levels of accuracy and comprehensiveness with simplification, Bard demonstrated a notable decrease in comprehensiveness among 34.8% of its simplified responses, while maintaining accuracy. Our study highlights the ability of LLMs, especially GPT-4, to increase their output readability on demand, highlighting their potential in enhancing access to easy-to-understand PEMs for all patients considering and undergoing bariatric surgery. We also highlight variability in LLM performance regarding maintaining accuracy and comprehensiveness when simplifying PEMs, with GPT-3.5 and GPT-4.0 outperforming Bard.

Our analysis shows that institutional websites’ PEMs remain too complex for the public, falling short of the AMA recommendation that PEMs be written at a 6th grade level or below [13]. These findings echo the results of previous studies that showed poor readability of bariatric surgery PEMs online [47, 48]. Furthermore, initial responses from LLMs were also found to have poor readability and in some instances worse readability than the institutions. These findings are concerning, as multiple studies have found an association between lower health literacy and reduced short-term and long-term weight loss post-bariatric surgery [7–9]. Furthermore, other studies have demonstrated an association between lower health literacy and reduced medical appointment follow-up 1 year after surgery [49] as well as diminished likelihood of eventually undergoing the surgery itself [50].

Considering this, we also examined the ability of LLMs to rephrase their initial responses in simpler terms. GPT-4, when prompted to simplify its responses, demonstrated superior adaptability to reader grade level by generating responses with greater readability, compared to institutional, simplified GPT-3.5, and simplified Bard responses. Furthermore, simplified GPT-4 responses met the AMA recommendation [13] for 2 out of the 6 readability instruments, with “fairly easy to read” readability based on the FRE scale (Table 2). Our findings demonstrate the ability of LLMs, particularly GPT-4, to simplify language in real time when prompted to do so. This may be valuable for patients seeking information from LLMs or healthcare providers, who may utilize this technology to improve the readability of their existing PEMs. The superior performance of GPT-4 over GPT-3.5 also highlights the rapid improvement in model performance with each iteration in a short period of time, further bolstering the potential of these models in the future.

The notable decrease in comprehensiveness of Bard responses when simplified highlights a critical issue regarding the balance between readability and content quality produced by LLMs, especially in the context of PEMs. While enhancing the readability of health information is an important goal, it is critical that we consider how the process of oversimplification may inadvertently impact PEM quality. Encouragingly, GPT-4 and GPT-3.5 maintained both accuracy and comprehensiveness, highlighting a potential area of improvement for Bard. We recommend further evaluation of the accuracy and comprehensiveness of rephrased or simplified PEMs in future studies, given the discrepancies in performance found in our analysis. Future iterations of LLMs should ensure that increased readability does not compromise the quality of PEMs delivered to patients.

The discrepancy in performance across the multiple LLMs evaluated in our research also underscores the need for a comprehensive discourse on the ability of LLMs to generate easy-to-understand materials for patients in the healthcare sector. This point is especially salient in light of the rapid evolution and roll-out of new LLMs (Llama 2.0, Med-PaLM 2), highlighting the urgency to ensure readability standards. The utilization of LLMs in healthcare necessitates a balance between sophisticated clinical vernacular and personalized patient-centered delivery of information. Models that generate language beyond the comprehension of the average patient may engender confusion, which may exacerbate existing health literacy disparities and potentially compromise the quality of healthcare. Thus, it is essential for these advanced models to optimize their output for readability and comprehension, thereby elevating the standard of patient-centered care and harnessing the full potential of artificial intelligence in medicine.

### Limitations and future directions

The readability assessment tools we selected for our study are widely recognized and utilized [33–37]. However, they possess inherent limitations, focusing predominantly on quantifiable aspects of text complexity such as sentence length and syllable count, rather than qualitative aspects such as subject familiarity, conceptual difficulty, and context. These tools also do not consider the popularity of certain words and phrases, which can significantly affect the readability of a given text. For example, while the Gunning Fog scale accounts for the number of syllables, it does not recognize that not all multisyllabic words are inherently complex [51] if they are familiar to the reader (e.g., the word “responsibility” has six syllables). The formulas also do not evaluate the organizational structure or layout of a text, which can significantly impact its navigability. Furthermore, our study also revealed that the assigned grade level for a text varies based on the assessment tool used, which may limit their reliability. Overall, while these formulas offer a standardized approach to assessing text readability, they do not account for the entire spectrum of factors that contribute to the ease of comprehension [52]. Looking forward, we encourage multifaceted approaches to readability studies and hope that more sophisticated tools that measure all aspects of text comprehension are developed in the near future.

The LLMs also have limitations that are currently under investigation. The sources of datasets used to train ChatGPT and Bard are largely unknown. Both OpenAI and Google acknowledge that the current versions of their respective LLMs may produce inaccurate information but hope to improve their performance via user feedback and model adjustments with future iterations. We hope that these constraints will diminish with ongoing enhancements to these models, resulting in even more accurate and consistent responses over time.

## Conclusion

Our study highlights the potential of large language models, particularly GPT-4, to enhance the readability of patient education materials related to bariatric surgery, aligning more closely with recommended readability standards. The ability of LLMs to adapt and simplify language in real time underscores their potential to democratize access to high-quality easy-to-understand medical information. Our study also revealed that the simplification of PEMs by LLMs may impact their quality. While all LLMs significantly improved the readability of PEMs, the comprehensiveness of simplified responses varied, underscoring the importance of evaluating both the readability and quality of PEMs generated by LLMs. The rapid evolution of these models, as evidenced by the superior performance of GPT-4 over GPT-3.5, emphasizes the urgency to harness their full potential in the healthcare sector. We recommend future investigation of the integration of artificial intelligence in patient-centered care, which will pave the way for more accessible and personalized approaches to medicine in the future.

### Supplementary Information

Below is the link to the electronic supplementary material.

Supplementary file1 (DOCX 13 KB)

Supplementary file2 (DOCX 123 KB)

Supplementary file3 (DOCX 111 KB)

Supplementary file4 (DOCX 121 KB)

Supplementary file5 (DOCX 8 KB)

Supplementary file6 (DOCX 9 KB)

Supplementary file7 (DOCX 16 KB)

Supplementary file8 (DOCX 7 KB)
