# Supplementary material for: Large language models and bariatric surgery patient education: a comparative readability analysis of GPT-3.5, GPT-4, Bard, and online institutional resources
Source: Surg Endosc. 2024 Mar 12;38(5):2522–32. doi: 10.1007/s00464-024-10720-2 (PMC11078810; doi:10.1007/s00464-024-10720-2)
Supplement: Supplementary file 1 — Supplementary file1 (DOCX 13 KB) [file 464_2024_10720_MOESM1_ESM.docx]

**Supplementary Table 1**. Flesch Reading Ease Formula Score Interpretation

| **Score** | **School Level** | **Notes** |
| --- | --- | --- |
| 90.0-100.0 | 5th Grade | Very easy to read. Easily understood by an average 11-year-old student. |
| 80.0-90.0 | 6th Grade | Easy to read. Conversational English for consumers. |
| 70.0-80.0 | 7th Grade | Fairly easy to read. |
| 60.0-70.0 | 8th and 9th Grade | Plain English. Easily understood by 13- to 15-year-old students. |
| 50.0-60.0 | 10th to 12th grade | Fairly difficult to read. |
| 30.0-50.0 | College | Difficult to read. |
| 10.0-30.0 | College graduate | Very difficult to read. Best understood by university graduates. |
| 0.0-10.0 | Professional | Extremely difficult to read. Best understood by university graduates. |

Score interpretation: derived from Chapter 2 of “How to Write Plain English” by Rudolf Flesch (https://web.archive.org/web/20160712094308/http://www.mang.canterbury.ac.nz/writing_guide/writing/flesch.shtml).
