# Supplementary material for: Large language models and bariatric surgery patient education: a comparative readability analysis of GPT-3.5, GPT-4, Bard, and online institutional resources
Source: Surg Endosc. 2024 Mar 12;38(5):2522–32. doi: 10.1007/s00464-024-10720-2 (PMC11078810; doi:10.1007/s00464-024-10720-2)
Supplement: Supplementary file 2 — Supplementary file2 (DOCX 123 KB) [file 464_2024_10720_MOESM2_ESM.docx]

**Supplementary Table 2**. GPT-3.5 Responses

| Institution | Question | Institutional Answer | Modified Question | GPT-3.5 Answer | ChatGPT Entry Date (Month/Day/Year) | “Can you explain that in simpler terms?” |
| --- | --- | --- | --- | --- | --- | --- |
| 1 | Why should I consider weight loss surgery? | Weight loss surgery is the most effective way to lose weight and keep weight off. Weight loss surgery can improve or resolve obesity-related conditions such as diabetes, high blood pressure, acid reflux and sleep apnea. It also decreases obesity-related cancer risk by 30%. Weight loss surgery can help improve your overall quality of life. |  | Weight loss surgery, also known as bariatric surgery, is a potential option for individuals who are struggling with obesity and have been unable to lose weight through traditional methods such as diet and exercise. It involves surgical procedures that aim to reduce the size of the stomach or modify the digestive system, leading to weight loss and potential improvements in overall health. Here are some reasons why you might consider weight loss surgery:  1. Effective weight loss: Bariatric surgery can help you achieve significant and sustained weight loss. It can be particularly beneficial for individuals with severe obesity who are at a higher risk of developing obesity-related health conditions.  2. Health benefits: Losing excess weight can have a positive impact on your overall health. Weight loss surgery has been associated with improvements or remission of various obesity-related conditions, such as type 2 diabetes, high blood pressure, sleep apnea, and joint pain.  3. Enhanced quality of life: Shedding excess weight can lead to an improved quality of life. You may experience increased mobility, reduced discomfort, and improved self-esteem and body image, which can positively impact your mental well-being.  4. Long-term weight management: Weight loss surgery can provide a long-lasting solution to obesity. While it's not a magic cure and requires lifestyle changes, it can help you establish healthier eating habits and facilitate weight maintenance in the long run.  5. Potential for reducing medication usage: As you lose weight and improve your health, you may be able to reduce or eliminate medications you were taking to manage obesity-related conditions such as diabetes, high blood pressure, or cholesterol.  6. Support and guidance: Undergoing weight loss surgery typically involves comprehensive support from a multidisciplinary team, including surgeons, dietitians, psychologists, and other healthcare professionals. They can provide guidance, education, and support throughout your weight loss journey.  However, it's important to note that weight loss surgery is not suitable for everyone. It is typically recommended for individuals with a body mass index (BMI) of 40 or higher (or 35 or higher with obesity-related health conditions). The decision to undergo weight loss surgery should be made in consultation with your healthcare provider, who can assess your individual circumstances and help you determine if it's a suitable option for you. | 6/25/2023 | Of course! Weight loss surgery is a type of surgery that can help people who are very overweight and haven't been able to lose weight through diet and exercise alone. Here are some reasons why you might consider it:  1. Effective weight loss: The surgery helps you lose a lot of weight and keep it off.  2. Health benefits: Losing weight can improve or even cure health problems related to obesity, like diabetes, high blood pressure, sleep apnea, and joint pain.  3. Better quality of life: Losing weight can make you feel better, move more easily, and feel happier about yourself.  4. Long-term solution: The surgery can help you make lasting changes to your lifestyle and keep the weight off.  5. Less need for medications: As you lose weight and get healthier, you may not need to take as much medicine for obesity-related conditions.  6. Support and guidance: You'll have a team of doctors, dietitians, and other professionals to help you before and after the surgery.  It's important to know that weight loss surgery isn't right for everyone. Your doctor can help you decide if it's a good option for you based on your weight and health. |
| 1 | What are my options for weight loss surgery at X? | Our surgeons offer Roux-en-Y gastric bypass, sleeve gastrectomy and the laparoscopic adjustable gastric band. We also offer revision surgery for patients who have had weight loss surgery before and have not achieved desired results. |  | As an AI language model, I don't have access to real-time information, and my knowledge is limited to what was available up until September 2021. Therefore, I cannot provide the most current information about specific medical institutions like X or their services after that date. It's best to visit X’s official website or contact their medical center directly to get the most up-to-date and accurate information about weight loss surgery options they offer.  However, as of my last update in 2021, some common weight loss surgeries that medical centers like X might offer include:  1. Gastric Bypass: Also known as Roux-en-Y Gastric Bypass, this procedure involves creating a smaller stomach pouch and rerouting a portion of the small intestine, which helps in reducing the amount of food you can eat and absorb.  2. Sleeve Gastrectomy: In this surgery, a large portion of the stomach is removed, leaving a smaller, banana-shaped stomach. This reduces the capacity of the stomach, leading to a feeling of fullness with smaller portions of food.  3. Adjustable Gastric Banding: This involves placing an adjustable band around the upper part of the stomach, creating a small pouch above the band. The band can be tightened or loosened to control food intake.  4. Biliopancreatic Diversion with Duodenal Switch (BPD/DS): A more complex surgery that involves both removing a portion of the stomach and rerouting the small intestine to reduce food absorption.  Before considering any weight loss surgery, it's crucial to consult with a qualified healthcare professional who specializes in bariatric surgery. They can assess your individual health situation and help determine the most appropriate option for you, along with discussing the potential risks and benefits of each procedure.  Please remember that weight loss surgery is a serious medical decision and should only be considered after careful consideration and evaluation of all available options. | 6/25/2023 | Of course! Let me explain weight loss surgeries in simpler terms:  1. Gastric Bypass: In this surgery, doctors create a smaller stomach pouch and connect it directly to the small intestine, bypassing a large part of the stomach. This makes you feel full with less food and reduces the number of calories your body absorbs.  2. Sleeve Gastrectomy: This surgery involves removing a large portion of the stomach, leaving behind a smaller, banana-shaped stomach. The smaller stomach makes you feel full faster and helps you eat less.  3. Adjustable Gastric Banding: Doctors place an adjustable band around the upper part of the stomach, creating a small pouch above it. The band can be tightened or loosened to control how much food you can eat.  4. Biliopancreatic Diversion with Duodenal Switch (BPD/DS): This is a more complex surgery where a part of the stomach is removed, and the small intestine is rerouted. It reduces the amount of food you can eat and decreases the calories your body absorbs.  These surgeries are only considered for people who have serious weight-related health issues and have not been able to lose weight through diet and exercise alone. It's essential to talk to a specialized doctor who can explain the options and help you decide which surgery, if any, might be suitable for your situation. Weight loss surgery is a significant decision that should be thoroughly discussed with medical professionals. |
| 1 | Who is eligible for weight loss surgery? | To qualify for weight loss surgery, you must have either a:  - BMI of 40 or greater  - BMI of 35-40 with obesity-related conditions such as diabetes, high blood pressure, sleep apnea or gastroesophageal reflux disease (GERD) |  | Weight loss surgery, also known as bariatric surgery, is a medical procedure typically recommended for individuals who are severely obese and have been unable to lose weight through traditional methods such as diet and exercise. Eligibility criteria for weight loss surgery may vary slightly depending on the specific procedure and the guidelines of the treating medical institution. However, there are some general criteria that are commonly considered for eligibility. These include:  1. Body Mass Index (BMI): Generally, individuals with a BMI of 40 or higher (severe obesity) or a BMI of 35 or higher with obesity-related health conditions, such as diabetes or hypertension, may be eligible for weight loss surgery. In some cases, individuals with a BMI of 30 to 34.9 who have significant obesity-related health problems may also be considered.  2. Failed attempts at weight loss: Candidates for weight loss surgery are typically required to have attempted other weight loss methods, including dietary changes, exercise, and supervised weight loss programs, without significant and sustainable results.  3. Age: There is usually a minimum age requirement for weight loss surgery, which is generally around 18 years. However, some procedures may be performed on adolescents or young adults in exceptional cases with severe obesity-related health issues.  4. Medical conditions: Weight loss surgery is often recommended for individuals who have obesity-related health conditions such as type 2 diabetes, sleep apnea, hypertension, or severe joint problems.  5. Psychological evaluation: Candidates for weight loss surgery may undergo a psychological evaluation to assess their mental readiness and ability to adhere to post-surgery lifestyle changes.  It's important to note that each case is unique, and eligibility for weight loss surgery is determined on an individual basis. A comprehensive evaluation by a qualified healthcare professional, such as a bariatric surgeon or a multidisciplinary weight loss team, is necessary to determine if someone is a suitable candidate for weight loss surgery. | 6/25/2023 | Certainly! Weight loss surgery, also known as bariatric surgery, is a procedure for people who are very overweight and have tried other methods to lose weight without success. To be eligible for weight loss surgery, certain criteria are usually considered:  1. Body Mass Index (BMI): It's a measure that combines your weight and height. If your BMI is 40 or higher (severe obesity) or 35 or higher with weight-related health problems, you may be eligible. Sometimes, people with a BMI of 30 to 34.9 and significant weight-related health issues can also be considered.  2. Attempts at weight loss: You need to have tried other ways to lose weight, such as changing your diet, exercising, or joining weight loss programs, but couldn't achieve significant and lasting results.  3. Age: Generally, you need to be at least 18 years old. However, in some exceptional cases, weight loss surgery may be considered for younger individuals with severe weight-related health problems.  4. Health conditions: Weight loss surgery is often recommended for people who have health issues like type 2 diabetes, sleep apnea, high blood pressure, or severe joint problems that are related to their obesity.  5. Psychological evaluation: Before the surgery, you may undergo an evaluation to assess your mental readiness and ability to make necessary lifestyle changes after the procedure.  It's important to remember that these are general guidelines, and each case is unique. A qualified healthcare professional, like a bariatric surgeon or a weight loss team, will assess your specific situation to determine if weight loss surgery is a suitable option for you. |
| 1 | What is the recovery time after weight loss surgery? | On average, most patients stay in the hospital one to two days after surgery. Most return to work two to three weeks after surgery. |  | The recovery time after weight loss surgery can vary depending on the specific procedure performed and the individual's overall health. In general, the initial recovery period after weight loss surgery typically lasts about 2 to 4 weeks. However, it's important to note that this is just the initial recovery phase, and the overall recovery process can take several months.  During the first few days after surgery, you will likely be in the hospital under close medical supervision. The healthcare team will monitor your condition, manage pain, and ensure that you're healing properly. You may also receive intravenous fluids and medications.  After being discharged from the hospital, you will need to follow a specific post-operative plan provided by your surgeon and healthcare team. This plan typically includes a modified diet, exercise guidelines, and regular follow-up appointments. Adhering to this plan is crucial for successful recovery and long-term weight loss.  It's important to note that weight loss surgery is a major procedure, and recovery can involve some discomfort, fatigue, and adjustments to your lifestyle. You may experience temporary side effects such as pain, swelling, bruising, or nausea. Your surgeon and healthcare team will provide guidance on managing these symptoms and monitoring your progress.  In terms of returning to normal activities, most individuals can typically resume light activities and work within a few weeks after surgery. However, more strenuous activities and exercise may need to be gradually introduced over time, based on your surgeon's recommendations.  Remember that each person's recovery is unique, and it's important to consult with your healthcare team for personalized guidance and support throughout the recovery process. They will provide you with specific instructions based on your procedure and individual circumstances. | 6/25/2023 | Certainly! After weight loss surgery, the time it takes to recover can vary from person to person and depends on the type of surgery you had. In general, the initial recovery phase typically lasts about 2 to 4 weeks.  During the first few days after surgery, you will be in the hospital. The doctors and nurses will take care of you, manage any pain you may have, and make sure you're healing well. You might receive fluids and medications through an IV.  Once you're discharged from the hospital, you'll need to follow a specific plan provided by your surgeon and healthcare team. This plan will include guidelines for your diet, exercise, and regular check-up appointments. It's important to follow this plan to recover well and achieve long-term weight loss.  Keep in mind that weight loss surgery is a major procedure, and it may take some time to feel better. You may experience temporary side effects like pain, swelling, bruising, or nausea. Your surgeon and healthcare team will give you advice on how to manage these symptoms and keep track of your progress.  As for getting back to your normal activities, you can usually start light activities and return to work within a few weeks after surgery. However, you should gradually reintroduce more intense activities and exercise, following your surgeon's recommendations.  Remember that everyone's recovery is different, so it's important to talk to your healthcare team for personalized guidance and support throughout the recovery process. They will give you specific instructions based on your surgery and individual needs. |
| 1 | How much weight can I expect to lose after surgery and how quickly? | Maximum weight loss typically occurs 12-24 months after surgery. Individual weight loss varies depending on:  - Age  - Weight before surgery  - Level of physical activity  - Diet  - Type of surgery | How much weight can I expect to lose after weight loss surgery and how quickly? | The amount of weight you can expect to lose after weight loss surgery, also known as bariatric surgery, can vary depending on several factors, including the type of surgery performed, your starting weight, your overall health, and your adherence to the post-surgical guidelines.  Generally, weight loss surgery can lead to significant weight loss, but the exact amount varies from person to person. On average, patients can typically expect to lose 50% to 70% of their excess body weight within the first two years after surgery. Excess weight refers to the weight above what is considered a healthy weight for your height and body type.  In terms of speed, weight loss usually occurs most rapidly in the first six months following surgery. During this time, patients may experience more significant weight loss, often referred to as the "honeymoon period." The rate of weight loss tends to slow down gradually after that initial phase.  It's important to note that weight loss surgery is not a magic solution, and success depends on making long-term lifestyle changes, including adopting a healthy diet, engaging in regular physical activity, and following the guidelines provided by your healthcare team. It's crucial to work closely with your healthcare professionals to establish realistic expectations and create a personalized plan for your weight loss journey. | 6/25/2023 | Certainly! After weight loss surgery, the amount of weight you can expect to lose varies from person to person. On average, patients lose about 50% to 70% of their excess body weight within the first two years.  In terms of speed, weight loss is usually fastest in the first six months after surgery. This is often called the "honeymoon period." After that, the rate of weight loss slows down gradually.  It's important to remember that weight loss surgery is not a quick fix. To be successful, you need to make long-term changes to your lifestyle, like eating a healthy diet, being physically active, and following the guidance of your healthcare team.  In simpler terms, weight loss surgery can help you lose a significant amount of weight, but the exact amount varies. The weight loss is fastest in the first six months, but it's important to make long-term changes to your lifestyle for lasting success. |
|  | What should I expect after surgery? | You will need to follow a bariatric diet and exercise daily, take bariatric vitamins lifelong, refrain from using nicotine (including cigarettes) and minimize alcohol use. | What should I expect after weight loss surgery? | After weight loss surgery, there are several physical and lifestyle changes that you can expect. It's important to note that the specific outcomes may vary depending on the type of weight loss surgery you undergo, such as gastric bypass, gastric sleeve, or gastric banding. However, here are some general expectations:  1. Weight loss: Weight loss surgery is designed to help you lose a significant amount of weight. In the first year after surgery, you may typically lose 50% to 70% of your excess body weight. Over time, the weight loss may slow down, and you can expect to reach a stable weight within 1 to 2 years.  2. Improved health conditions: Weight loss surgery often leads to improvements in various obesity-related health conditions, such as type 2 diabetes, high blood pressure, sleep apnea, and joint pain. Many people experience a reduction or even resolution of these conditions post-surgery.  3. Dietary changes: Following weight loss surgery, you will need to make significant changes to your diet. Initially, you will be on a liquid or pureed diet for a few weeks, gradually transitioning to solid foods. Your healthcare team will provide specific guidelines on portion sizes, nutrient intake, and dietary modifications to support your weight loss and ensure adequate nutrition.  4. Lifestyle adjustments: Weight loss surgery requires long-term commitment to a healthy lifestyle. You will need to adopt regular exercise habits and engage in physical activity to support weight loss and maintain muscle tone. Additionally, you'll need to develop new eating habits, such as eating smaller meals, chewing food thoroughly, and avoiding high-calorie, sugary, and fatty foods.  5. Emotional changes: Weight loss surgery can bring about emotional and psychological changes. While the surgery can positively impact self-esteem and body image, it's important to address any underlying emotional issues related to food, body image, or past experiences with weight. Support from a therapist or counselor can be beneficial during this process.  6. Follow-up care: After weight loss surgery, you will have regular follow-up appointments with your healthcare team to monitor your progress, adjust medications, and ensure that you're adapting well to the lifestyle changes. It's crucial to attend these appointments and follow the guidance of your healthcare professionals.  Remember that weight loss surgery is a tool to assist in weight loss, but it requires active participation and dedication on your part to achieve long-term success. The specific outcomes and experiences can vary, so it's essential to consult with your healthcare provider for personalized information based on your individual circumstances. | 6/25/2023 | Certainly! After weight loss surgery, here are some things you can expect:  1. Weight loss: You'll lose a significant amount of weight, usually around half of your excess body weight within the first year. The weight loss may continue for up to two years until it stabilizes.  2. Improved health: Weight loss surgery often improves health conditions related to obesity, like diabetes, high blood pressure, sleep apnea, and joint pain. Some of these conditions may even go away completely.  3. Changes in eating: You'll need to make changes to your diet. At first, you'll have to eat liquids or pureed foods, and then gradually transition to solid foods. You'll need to eat smaller meals, avoid high-calorie foods, and chew your food thoroughly.  4. Lifestyle adjustments: Regular exercise is important for weight loss and maintaining muscle tone. You'll need to develop new habits like being physically active and avoiding unhealthy foods.  5. Emotional changes: Weight loss surgery can affect your emotions and self-image. It's important to address any emotional issues related to food or body image and seek support from a therapist if needed.  6. Follow-up care: You'll have regular check-ups with your healthcare team to monitor your progress, adjust medications if necessary, and ensure you're adapting well to the changes.  Remember, weight loss surgery is a tool to help you lose weight, but you'll need to actively participate and make long-term lifestyle changes for it to be successful. It's important to talk to your healthcare provider for personalized advice based on your situation. |
|  | What is body mass index (BMI), and how can I calculate mine? | Body mass index (BMI) is a tool to screen for obesity, factoring in your height and weight. Learn your BMI with our BMI calculator. |  | Body Mass Index (BMI) is a numerical value that is used to assess the relationship between a person's weight and height. It provides a rough estimation of whether an individual has a healthy body weight or if they are underweight, overweight, or obese. While BMI doesn't directly measure body fat, it is commonly used as a screening tool to identify potential weight-related health issues.  To calculate your BMI, you can use the following formula:  BMI = weight (kg) / (height (m))^2  Here's an alternative formula if you prefer using pounds and inches:  BMI = (weight (lbs) / (height (in))^2) x 703  Here's a step-by-step guide on calculating BMI:  1. Convert your weight to kilograms if necessary. To convert pounds to kilograms, divide the weight in pounds by 2.2046.  2. Convert your height to meters if necessary. To convert inches to meters, divide the height in inches by 39.37.  3. Square the height in meters (height x height) or square the height in inches (height x height) before multiplying by 703.  4. Divide your weight in kilograms by the result obtained in step 3. This will give you your BMI.  Once you have calculated your BMI, you can refer to standard BMI categories to interpret the result:  - Underweight: BMI less than 18.5  - Normal weight: BMI between 18.5 and 24.9  - Overweight: BMI between 25 and 29.9  - Obesity: BMI of 30 or higher  Keep in mind that BMI is a general guideline and may not be applicable to certain individuals, such as athletes with a higher muscle mass. It's always a good idea to consult with a healthcare professional for a comprehensive assessment of your health and body weight. | 6/25/2023 | Certainly! Body Mass Index (BMI) is a number that helps determine if a person's weight is within a healthy range for their height. It's a basic calculation that gives an idea of whether someone is underweight, normal weight, overweight, or obese.  To calculate your BMI, you need to know your weight and height. You can use either kilograms (kg) or pounds (lbs) for weight and either meters (m) or inches (in) for height. Here's a simple step-by-step process:  1. Measure your weight. If you use pounds, divide it by 2.2046 to convert it to kilograms.  2. Measure your height. If you use inches, divide it by 39.37 to convert it to meters.  3. Square your height by multiplying it by itself. This gives you the result of height multiplied by height.  4. Divide your weight (in kilograms) by the result obtained in step 3. If you're using pounds and inches, multiply the result from step 3 by 703 before dividing your weight.  The number you get is your BMI. After calculating your BMI, you can refer to the following categories:  - Underweight: BMI less than 18.5  - Normal weight: BMI between 18.5 and 24.9  - Overweight: BMI between 25 and 29.9  - Obesity: BMI of 30 or higher  Remember that BMI is a general guideline and may not account for certain factors like muscle mass. If you have concerns about your weight or health, it's always a good idea to consult with a healthcare professional who can provide a more comprehensive evaluation. |
| 2 | Why should someone consider having bariatric surgery? | Obesity is harmful to a person’s health. Someone who is 40 percent overweight is twice more likely to die prematurely than an average-weight person. (This effect is seen after 10 to 30 years of being obese.) In this country alone, about 300,000 deaths per year can be blamed on obesity. Obesity is a risk factor for many serious, life-threatening diseases, including:  - Diabetes.  - Cancer.  - High blood pressure (hypertension).  - Heart disease.  - Obstructive sleep apnea.  - Stroke.  Studies show that the risk of death from these conditions returns to normal after weight loss. Within the first six months of having weight loss surgery, patients usually no longer need to take medications for these conditions. In addition, women who were struggling with infertility before surgery find that conception is possible after surgery. |  | Bariatric surgery, also known as weight loss surgery, is a medical procedure that is considered for individuals who are severely obese and have been unable to lose weight through other methods such as diet and exercise. Here are some reasons why someone might consider bariatric surgery:  1. Effective weight loss: Bariatric surgery can result in significant weight loss, which can lead to improvements in overall health and a reduction in obesity-related medical conditions. It can help individuals lose a substantial amount of weight and maintain that weight loss in the long term.  2. Health benefits: Obesity is associated with various health issues such as type 2 diabetes, high blood pressure, sleep apnea, heart disease, and joint problems. Bariatric surgery can help improve or even resolve these conditions in many cases. It has been shown to have positive effects on blood sugar control, cholesterol levels, and overall cardiovascular health.  3. Increased quality of life: Losing a significant amount of weight can have a profound impact on a person's quality of life. Bariatric surgery can improve physical mobility, increase energy levels, enhance self-esteem and body image, and reduce the risk of depression and anxiety associated with obesity.  4. Long-term sustainability: Bariatric surgery is not a quick fix or an easy way out. It is a tool that helps individuals make long-term lifestyle changes by reducing the capacity of the stomach or altering the digestive system. Following surgery, patients are required to make dietary and behavioral changes to achieve sustainable weight loss and maintain their health.  5. Multidisciplinary support: Bariatric surgery typically involves a comprehensive approach that includes pre-operative evaluations, counseling, dietary guidance, and ongoing support from a multidisciplinary team, including surgeons, dietitians, psychologists, and support groups. This support system can help individuals navigate the challenges associated with weight loss and maintain long-term success.  It is important to note that bariatric surgery is not suitable for everyone, and the decision to undergo the procedure should be made in consultation with a qualified healthcare professional. Factors such as overall health, body mass index (BMI), and previous attempts at weight loss will be taken into consideration to determine the appropriateness of bariatric surgery as a treatment option. | 6/25/2023 | Certainly! Bariatric surgery is a type of weight loss surgery for people who are very overweight and have tried other methods to lose weight without success. Here are some reasons why someone might consider it:  1. Effective weight loss: Bariatric surgery helps people lose a lot of weight and keep it off.  2. Health benefits: Losing weight through surgery can improve or even cure health problems related to obesity, like diabetes, high blood pressure, and sleep apnea.  3. Better quality of life: Losing weight can make it easier to move around, increase energy levels, boost self-confidence, and reduce the risk of depression or anxiety.  4. Long-term success: Bariatric surgery is a tool that helps people make long-term changes to their lifestyle. It's not a quick fix, but with the right changes, weight loss can be maintained.  5. Support system: Bariatric surgery comes with a team of healthcare professionals who offer support and guidance before and after the surgery. They help with diet, emotional well-being, and provide ongoing support.  Remember, bariatric surgery is not for everyone. It's important to consult with a healthcare professional to determine if it's the right option based on your health and weight loss history. |
| 2 | What bariatric surgical procedures are performed by surgeons at X? | X offers many surgical options for patients who are interested in bariatric surgery.  The most commonly performed malabsorptive procedure is called the Roux-en-Y gastric bypass. This method makes the stomach smaller and alters digestion. Less food can be eaten and fewer calories can be absorbed. The bariatric surgeon creates a small pouch by dividing the upper end of the stomach. Next, a Y-shaped section of the small intestine is attached to the pouch to allow food to bypass the lower stomach, the duodenum (the first segment of the small intestine), as well as the first portion of the jejunum (the second segment of the small intestine). The procedure creates a direct connection from the stomach to the lower segment of the small intestine, literally bypassing portions of the digestive tract that absorb calories and nutrients.  We can also perform the sleeve gastrectomy as a laparoscopic procedure. This involves making five or six small incisions in the abdomen and performing the procedure using a video camera (laparoscope) and long instruments that are placed through these small incisions. During the laparoscopic sleeve gastrectomy (LSG), about 75% of the stomach is removed leaving a narrow gastric “tube” or “sleeve”. No intestines are removed or bypassed during the sleeve gastrectomy. The LSG takes one to two hours to complete. We have an animated video of a sleeve gastrectomy procedure available as well.  In select patients, depending on their situations and needs, X bariatric surgeons can perform other surgical options such as duodenal switch. |  | As of my last update in September 2021, X is a renowned medical institution that offers various bariatric surgical procedures to help patients with obesity and related health conditions. The specific procedures performed by surgeons at X may vary over time, so it's essential to consult their official website or directly contact them for the most up-to-date information.  However, some of the common bariatric surgical procedures that were traditionally offered at leading institutions like X include:  1. Gastric Bypass (Roux-en-Y): This procedure involves creating a small stomach pouch and rerouting a portion of the small intestine, which restricts the amount of food you can eat and reduces nutrient absorption.  2. Sleeve Gastrectomy: In this procedure, a large portion of the stomach is removed, leaving a smaller, banana-shaped stomach. It works by reducing the stomach's capacity and producing hormones that decrease appetite.  3. Adjustable Gastric Banding: Also known as Lap-Band surgery, this procedure involves placing an adjustable band around the upper part of the stomach, creating a small pouch that limits the amount of food the stomach can hold.  4. Biliopancreatic Diversion with Duodenal Switch (BPD/DS): This complex procedure involves two stages. First, a portion of the stomach is removed, similar to a sleeve gastrectomy. Then, the small intestine is rearranged to reduce nutrient absorption further.  5. Revisional Bariatric Surgery: For patients who have already undergone a bariatric procedure but haven't achieved the desired results or have experienced complications, revisional surgeries may be considered to correct or modify the initial surgery.  Please remember that advancements in medical practices and technology might have introduced new procedures or variations to the traditional ones mentioned above. For the most accurate and current information about bariatric surgical procedures offered by X, I recommend visiting their official website or contacting them directly. | 6/25/2023 | Of course! Bariatric surgical procedures are operations done to help people who have obesity (severe overweight) and related health issues. These surgeries aim to reduce the size of the stomach or change how it works, so patients feel full with less food and absorb fewer calories. This helps them lose weight and improve their health.  Here are some common bariatric surgeries:  1. Gastric Bypass: The surgeon makes the stomach smaller and changes the way food goes through the intestine, so the patient eats less and absorbs fewer nutrients.  2. Sleeve Gastrectomy: The surgeon removes a large part of the stomach, leaving a smaller one shaped like a banana. This limits the amount of food the stomach can hold and helps the patient feel full faster.  3. Adjustable Gastric Banding (Lap-Band): The surgeon places an adjustable band around the upper part of the stomach, creating a small pouch. This restricts the amount of food the stomach can take in.  4. Biliopancreatic Diversion with Duodenal Switch (BPD/DS): This is a complex surgery that involves two steps. First, a portion of the stomach is removed (similar to sleeve gastrectomy). Then, the small intestine is rearranged to reduce nutrient absorption.  5. Revisional Bariatric Surgery: Sometimes, people who already had bariatric surgery might need further adjustments or corrections to achieve better results or deal with complications.  Each procedure has its benefits and considerations, and the best choice depends on the patient's individual needs and medical condition. It's essential for patients to consult with a qualified bariatric surgeon to determine the most suitable approach for their situation. |
| 2 | Who is eligible for bariatric surgery? | At X, we carefully evaluate all patients being considered for bariatric surgery. Nutritional and behavioral counseling are conducted, and patients are required to attend at least one weekly support group session prior to weight loss surgery.  To be eligible for bariatric surgery, you must be between 16 and 70 years of age (with some exceptions) and morbidly obese (weighing at least 100 pounds over your ideal body weight and having a BMI of 40). A BMI between 35 and 39 also may qualify you for the procedure if you also have certain pre-existing co-morbidities, such as diabetes, cardiovascular disease, hypertension, or sleep apnea.  If you are a woman of childbearing age and planning a pregnancy, it’s vital to know that you must not get pregnant within the first 18 months to two years following surgery. The rapid weight loss and nutritional deficiencies associated with bariatric surgery make pregnancy very dangerous for you and for a developing fetus.  A consultation with a X bariatric surgeon is a mandatory step prior to surgery, and will help you screen for eligibility. |  | Bariatric surgery, also known as weight loss surgery, is typically recommended for individuals who are severely obese and have not been able to achieve significant weight loss through other methods such as diet and exercise. The specific eligibility criteria can vary depending on the country, healthcare provider, and type of bariatric surgery being considered. However, here are some general guidelines:  1. Body Mass Index (BMI): Bariatric surgery is generally considered for individuals with a BMI of 40 or higher, or a BMI of 35-39.9 with obesity-related health conditions such as type 2 diabetes, high blood pressure, or sleep apnea. In some cases, individuals with a BMI between 30 and 34.9 may be considered for bariatric surgery if they have significant obesity-related health problems.  2. Failed Weight Loss Attempts: Bariatric surgery is typically recommended for individuals who have tried and failed to lose weight through non-surgical methods such as dieting, exercise, and lifestyle changes. Documentation of previous weight loss attempts is often required.  3. Age: The age requirements can vary, but most bariatric surgery programs have a minimum age requirement of 18. Some programs may consider surgery for individuals aged 16-18 in exceptional cases, such as if the person has severe obesity-related health problems.  4. Medical and Psychological Evaluation: Candidates for bariatric surgery usually undergo a thorough medical and psychological evaluation to assess their overall health and readiness for the procedure. This evaluation helps identify any potential risks or contraindications for surgery.  5. Commitment to Lifestyle Changes: Bariatric surgery is not a quick fix for weight loss. Candidates are required to demonstrate a commitment to long-term lifestyle changes, including dietary modifications, regular exercise, and ongoing medical follow-up after surgery.  It's important to note that eligibility criteria may vary based on individual circumstances and the specific recommendations of healthcare professionals. If you are considering bariatric surgery, it is best to consult with a qualified healthcare provider who specializes in bariatric surgery to determine if you meet the criteria and to discuss the potential risks and benefits of the procedure. | 6/25/2023 | Certainly! Bariatric surgery, or weight loss surgery, is an option for people who are very overweight and have tried other ways to lose weight without success. Here are some things that are considered when determining if someone is eligible for this surgery:  1. Body Mass Index (BMI): Doctors look at a person's BMI, which is a number calculated using their weight and height. Bariatric surgery is usually considered for people with a BMI of 40 or higher, or a BMI of 35-39.9 if they have health problems related to their weight.  2. Failed Weight Loss Attempts: Doctors want to see that a person has tried other methods to lose weight, like dieting and exercise, but haven't been able to lose enough weight.  3. Age: Most bariatric surgery programs have a minimum age requirement of 18. In some exceptional cases, younger individuals with severe health problems related to their weight may be considered.  4. Medical and Psychological Evaluation: Candidates for surgery go through a thorough evaluation to make sure they are healthy enough for the procedure and mentally prepared for the lifestyle changes that come with it.  5. Commitment to Lifestyle Changes: Bariatric surgery is not a quick fix. People need to show that they are willing to make long-term changes to their diet, exercise, and follow-up with their doctor after the surgery.  It's important to remember that these are general guidelines, and individual circumstances may vary. If you're thinking about bariatric surgery, it's best to talk to a doctor who specializes in this area to find out if you meet the criteria and to discuss the risks and benefits involved. |
| 2 | What is the recovery time following weight loss surgery? | X’s medical team has an immense wealth of experience in bariatric surgery, this combined with advances in the field of bariatric care, means that most patients recover from surgery quickly and without complications. On average the hospital stay for patients who undergo the Roux-en-Y gastric bypass is usually two to four days. |  | The recovery time following weight loss surgery can vary depending on the specific procedure performed and the individual's overall health. Generally, patients can expect a recovery period of several weeks to a few months. Here is a general timeline of what you might expect after weight loss surgery:  1. Hospital Stay: Most weight loss surgeries require a hospital stay of 1 to 3 days. During this time, you will be closely monitored by medical professionals to ensure your safety and recovery.  2. Initial Recovery: The first few days after surgery are focused on rest and healing. You may experience some pain, discomfort, or nausea, which can be managed with medications prescribed by your surgeon. You will gradually transition from a liquid diet to pureed foods during this time.  3. Transition to Solid Foods: Over the following weeks, you will gradually introduce solid foods back into your diet. Your surgeon and dietitian will provide specific guidelines on what and how to eat to promote proper healing and weight loss.  4. Follow-up Visits: You will have regular follow-up visits with your surgeon and healthcare team to monitor your progress, address any concerns, and make adjustments to your diet or medications as needed.  5. Physical Activity: Your surgeon will provide guidance on when you can start incorporating physical activity into your routine. Initially, it may be limited to light exercises or short walks, gradually increasing in intensity and duration as your body heals.  6. Long-Term Recovery: Full recovery from weight loss surgery can take several months. During this time, your body will adapt to the changes, and you will continue to lose weight. It's important to maintain a healthy lifestyle, including a nutritious diet and regular exercise, to maximize the benefits of the surgery and support long-term weight loss.  Remember, the recovery time can vary from person to person, so it's crucial to follow your surgeon's instructions and consult with your healthcare team for personalized advice based on your specific situation. | 6/25/2023 | Certainly! After weight loss surgery, here's what you can generally expect in terms of recovery:  1. Hospital Stay: You'll stay in the hospital for a few days, typically 1 to 3 days, where doctors will take care of you.  2. Initial Recovery: Right after the surgery, you'll need to rest and let your body heal. You might feel some pain, discomfort, or nausea, but medications can help manage those. During this time, you'll start with liquids and gradually move on to pureed foods.  3. Transition to Solid Foods: Over the next few weeks, you'll slowly reintroduce solid foods into your diet. Your surgeon and dietitian will give you specific instructions on what and how to eat to help you heal and lose weight.  4. Follow-up Visits: You'll have regular check-ups with your surgeon and healthcare team to monitor your progress, address any concerns, and make any necessary adjustments to your diet or medications.  5. Physical Activity: Your surgeon will guide you on when you can start including physical activity in your routine. Initially, it might be light exercises or short walks, and you'll gradually increase the intensity and duration as you heal.  6. Long-Term Recovery: It takes several months to fully recover from weight loss surgery. During this time, your body will adjust to the changes, and you'll continue to lose weight. To make the most of the surgery and support long-term weight loss, it's important to maintain a healthy lifestyle, including eating nutritious foods and exercising regularly.  Remember, everyone's recovery time can be different, so it's important to follow your surgeon's instructions and talk to your healthcare team for personalized advice based on your situation. |
| 2 | How much weight loss can be expected? | Most patients will lose between 66 and 80 percent of their excess body weight, most of which is lost 18 to 24 months following bariatric surgery. | How much weight loss can be expected following weight loss surgery? | The amount of weight loss that can be expected following weight loss surgery, also known as bariatric surgery, can vary depending on several factors. These factors include the type of surgery performed, the individual's starting weight, their adherence to post-operative guidelines, and their overall commitment to lifestyle changes.  On average, individuals who undergo weight loss surgery can expect to lose a significant amount of weight. The most common types of weight loss surgeries are gastric bypass, gastric sleeve, and adjustable gastric banding (also known as lap band surgery).  Gastric bypass and gastric sleeve surgeries are considered more effective for significant and sustained weight loss compared to adjustable gastric banding. Generally, patients can expect to lose 50% to 70% of their excess body weight within the first two years following gastric bypass or gastric sleeve surgery. This can amount to an average weight loss of 60 to 80 pounds (27 to 36 kilograms) within the first year.  However, it's important to note that weight loss results can vary. Some individuals may lose more weight, while others may lose less. The commitment to lifestyle changes, such as adopting a healthy diet and regular exercise, plays a crucial role in achieving and maintaining weight loss after surgery.  It's also worth mentioning that weight loss surgery is not a standalone solution. It is typically recommended for individuals who have tried and failed to lose weight through other methods and who have a body mass index (BMI) of 40 or higher, or a BMI of 35 or higher with obesity-related health conditions. The goal of weight loss surgery is to help individuals achieve a healthier weight and improve or resolve obesity-related health issues. | 6/25/2023 | Certainly! Weight loss surgery, also known as bariatric surgery, can help people who are struggling with severe obesity to lose a significant amount of weight. The amount of weight loss can vary depending on factors like the type of surgery, starting weight, following guidelines, and making lifestyle changes.  The most common types of weight loss surgeries are gastric bypass, gastric sleeve, and adjustable gastric banding. Gastric bypass and gastric sleeve surgeries are more effective for significant weight loss compared to adjustable gastric banding. On average, people who undergo gastric bypass or gastric sleeve surgery can expect to lose around 50% to 70% of their excess body weight within the first two years. This can amount to an average weight loss of 60 to 80 pounds (27 to 36 kilograms) within the first year.  However, it's important to remember that weight loss results can vary from person to person. Some individuals may lose more weight, while others may lose less. Following a healthy diet, exercising regularly, and making long-term lifestyle changes are important for achieving and maintaining weight loss after surgery.  It's also essential to understand that weight loss surgery is not a quick fix. It is typically recommended for individuals who have tried other methods to lose weight without success and who have a high body mass index (BMI) or obesity-related health conditions. The goal of weight loss surgery is to help individuals reach a healthier weight and improve or resolve obesity-related health problems. |
| 2 | What are the dietary restrictions following weight loss surgery? | Following surgery, you will need to follow specific eating guidelines. Registered dietitians at X work directly with bariatric surgery patients to help plan healthful, appropriate meals. Additionally, you will need to stop smoking and discontinue use of NSAIDS. A list of NSAIDS is available for you to reference. |  | Following weight loss surgery, also known as bariatric surgery, there are certain dietary restrictions that patients typically need to follow. These restrictions are put in place to promote proper healing, aid in weight loss, and prevent complications. However, it's important to note that specific guidelines may vary depending on the type of weight loss surgery performed and the recommendations of your healthcare team. Here are some general dietary guidelines that are often recommended:  1. Clear liquid phase: Immediately after surgery, you will typically start with a clear liquid diet, which includes water, broth, sugar-free gelatin, and clear protein drinks. This phase usually lasts for a few days to a week.  2. Full liquid phase: Following the clear liquid phase, you will gradually transition to a full liquid diet. This includes items like protein shakes, skim milk, low-fat yogurt, strained cream soups, and sugar-free pudding. This phase usually lasts for about one to two weeks.  3. Pureed food phase: Once you tolerate the full liquid diet, you can move on to pureed or mashed foods. This includes foods like pureed lean meats, soft cooked vegetables, pureed fruits, and cottage cheese. The duration of this phase varies but is typically a few weeks.  4. Soft food phase: After the pureed food phase, you can progress to soft, solid foods. These foods should be easily chewable and well-cooked. Examples include tender meats, cooked vegetables, soft fruits, and low-fat dairy products.  5. Regular diet: Eventually, you will transition to a regular, well-balanced diet. However, portion control is crucial, and you will likely need to eat smaller meals more frequently. Your healthcare team will provide guidance on calorie intake, macronutrient distribution, and appropriate food choices.  In addition to these dietary phases, it's essential to:  - Stay well-hydrated by drinking enough fluids, especially water.  - Avoid carbonated beverages and high-calorie liquids, as they can contribute to weight regain.  - Limit the consumption of high-sugar, high-fat, and processed foods.  - Chew your food thoroughly and eat slowly to aid digestion.  - Follow any specific vitamin and mineral supplementation recommendations provided by your healthcare team.  Remember, these guidelines are general and may vary depending on your specific surgery and the advice of your healthcare provider. It's important to work closely with your medical team, including a registered dietitian experienced in bariatric nutrition, to develop an individualized plan that meets your unique needs. | 6/25/2023 | Certainly! After weight loss surgery, there are certain dietary guidelines you'll need to follow. Here's a simplified explanation:  1. Clear liquid phase: Right after surgery, you'll start with clear liquids like water, broth, and sugar-free gelatin.  2. Full liquid phase: You'll then move on to more filling liquids like protein shakes, skim milk, and low-fat yogurt.  3. Pureed food phase: Next, you'll eat foods that are pureed or mashed, like pureed lean meats, soft-cooked vegetables, and cottage cheese.  4. Soft food phase: After that, you can have soft, easy-to-chew foods like tender meats, cooked vegetables, and soft fruits.  5. Regular diet: Eventually, you'll return to a normal diet, but you'll need to eat smaller meals more often and choose nutritious foods.  Other important points are:  - Drink plenty of fluids, especially water.  - Avoid sugary drinks and unhealthy foods.  - Chew your food well and eat slowly.  - Take any vitamin or mineral supplements as advised.  Remember to work closely with your healthcare team, including a dietitian, to create a plan that's right for you. |
| 2 | Is it possible to gain the weight back after surgery? | Bariatric surgery has an excellent long-term track record for helping morbidly obese individuals lose weight. However, there are no guarantees with any method of weight loss, even surgery. Success is possible only if you are committed to making lifestyle and dietary changes for the rest of your life. | Is it possible to gain the weight back after weight loss surgery? | Yes, it is possible to gain weight back after weight loss surgery. Weight loss surgery, such as gastric bypass or gastric sleeve surgery, can be effective in helping individuals achieve significant weight loss. However, it is not a guarantee that the weight loss will be permanent.  Several factors can contribute to weight regain after weight loss surgery, including:  1. Poor dietary choices: Some individuals may revert to unhealthy eating habits, such as consuming high-calorie or nutrient-poor foods, which can lead to weight regain.  2. Inadequate physical activity: Insufficient exercise or physical activity can hinder weight maintenance and contribute to weight gain.  3. Stretching of the stomach pouch: Over time, the stomach pouch created during surgery may stretch, allowing for larger food intake and potentially reducing the effectiveness of the procedure.  4. Psychological factors: Emotional and psychological factors, such as stress, depression, or disordered eating, can influence eating behaviors and contribute to weight regain.  5. Hormonal changes: Hormonal changes following weight loss surgery can affect appetite and metabolism, potentially leading to weight regain.  It's important to note that weight loss surgery is not a standalone solution for weight management. It requires a comprehensive approach that includes long-term lifestyle changes, such as adopting a healthy diet, regular exercise, and ongoing support from healthcare professionals and support groups. | 6/25/2023 | Certainly! After weight loss surgery, it is possible for a person to gain back the weight they lost. Here are some reasons why this can happen:  1. Eating unhealthy foods: If someone starts eating high-calorie or low-nutrient foods again, they can gain weight.  2. Not being active enough: If a person doesn't exercise or move their body regularly, it can make it harder to maintain their weight loss.  3. The stomach stretching: Over time, the stomach pouch created during surgery may become bigger, allowing for larger amounts of food to be eaten and making the surgery less effective.  4. Emotional factors: Feeling stressed, sad, or having an unhealthy relationship with food can lead to overeating and weight gain.  5. Hormonal changes: After weight loss surgery, hormones can change, affecting appetite and metabolism, which can impact weight.  Remember, weight loss surgery is not a magic fix. It requires making long-term changes to your lifestyle, like eating a healthy diet, staying active, and getting support from professionals and support groups to help you maintain your weight loss. |
| 2 | What are the risks for bariatric surgery? | All surgical procedures have risks, particularly when the patient is morbidly obese. Different procedures involve different risks, and depending upon your individual circumstances, your risks may be higher or lower than average. It’s also important to know that bariatric surgeons with more experience performing weight loss surgery techniques report fewer complications. Potential risks of surgery should be discussed with your surgeon so you can make an informed choice. |  | Bariatric surgery, also known as weight loss surgery, is a major medical procedure that carries both benefits and risks. While it can be an effective treatment for severe obesity and related health conditions, it's important to consider the potential risks involved. Some of the risks associated with bariatric surgery include:  1. Surgical complications: As with any surgical procedure, there are risks of complications such as bleeding, infection, blood clots, adverse reactions to anesthesia, or leaks from the surgical connections made during the procedure.  2. Nutritional deficiencies: Bariatric surgery alters the digestive system, which can impact the body's ability to absorb nutrients effectively. Patients may be at risk of developing deficiencies in essential vitamins and minerals, such as iron, vitamin B12, calcium, and others. Regular monitoring and appropriate supplementation are essential to prevent deficiencies and associated health problems.  3. Dumping syndrome: This condition can occur after certain types of bariatric surgeries, such as gastric bypass. It happens when food moves too quickly from the stomach to the small intestine, leading to symptoms like nausea, vomiting, diarrhea, dizziness, and sweating.  4. Gallstones: Rapid weight loss following bariatric surgery can increase the risk of developing gallstones, which may require additional treatment.  5. Bowel obstruction: In some cases, bariatric surgery can lead to the development of scar tissue or adhesions that cause blockages in the intestines, resulting in bowel obstruction. This may require further surgery to correct.  6. Ulcers or gastritis: Changes in the stomach anatomy can increase the risk of developing ulcers or inflammation of the stomach lining (gastritis).  7. Psychological and emotional challenges: Bariatric surgery is a life-altering procedure that can have a significant impact on a person's body image, self-esteem, and emotional well-being. Some individuals may experience psychological issues such as depression, body dysmorphia, or eating disorders following surgery.  8. Long-term maintenance: Bariatric surgery is not a cure for obesity. Long-term success depends on adopting a healthy lifestyle, including a balanced diet, regular exercise, and ongoing medical follow-up. Failure to maintain these lifestyle changes can result in weight regain.  It's crucial to have a thorough discussion with your healthcare provider about the potential risks and benefits of bariatric surgery, as well as your individual circumstances and medical history, to make an informed decision. The risks and outcomes may vary depending on the specific type of bariatric surgery chosen, as there are different procedures available, including gastric bypass, gastric sleeve, and gastric banding. | 6/25/2023 | Certainly! Bariatric surgery, which is a weight loss surgery, can be helpful for people who have severe obesity. However, it's important to understand that there are risks involved. Here are some of the potential risks:  1. Surgical complications: Like with any surgery, there is a risk of bleeding, infection, blood clots, and problems with anesthesia. Sometimes, there may be leaks where the surgery was done.  2. Nutritional deficiencies: After the surgery, the body may have trouble absorbing important nutrients like vitamins and minerals. This can lead to deficiencies, so it's important to take supplements and get regular check-ups.  3. Dumping syndrome: This can happen after certain types of surgery. It occurs when food moves too quickly from the stomach to the intestines, causing symptoms like nausea, vomiting, diarrhea, dizziness, and sweating.  4. Gallstones: Rapid weight loss can increase the risk of developing gallstones, which may require further treatment.  5. Bowel obstruction: Surgery can sometimes lead to blockages in the intestines, which may require additional surgery to fix.  6. Ulcers or gastritis: Changes to the stomach can increase the risk of developing ulcers or inflammation in the stomach lining.  7. Psychological and emotional challenges: Bariatric surgery is a big change and can affect a person's body image, self-esteem, and emotions. Some people may experience depression, problems with body image, or eating disorders after the surgery.  8. Long-term maintenance: Bariatric surgery is not a cure for obesity. It's important to maintain a healthy lifestyle with a balanced diet and regular exercise. If these changes aren't maintained, weight regain can occur.  It's essential to talk to your doctor about the risks and benefits of bariatric surgery, considering your specific situation and medical history. There are different types of bariatric surgery, so the risks and outcomes may vary depending on the procedure chosen. |
| 2 | Will insurance cover bariatric surgery? | Many insurance companies now recognize obesity as a substantial health risk and are paying for bariatric surgery. You can check our list of accepted insurers or contact your insurance provider to find out if gastric bypass surgery (CPT code 43644) is a covered benefit under your insurance contract. If you have other questions, please contact Debra Cash at 216.445.1745 / 800.223.2273, ext. 51745. |  | Whether insurance covers bariatric surgery depends on several factors, including the specific insurance plan, the individual's policy coverage, and the criteria set by the insurance company. In many cases, insurance companies do provide coverage for bariatric surgery, but there are typically certain requirements that need to be met.  Insurance companies may require individuals to meet certain criteria before approving coverage for bariatric surgery. These criteria often include having a body mass index (BMI) above a certain threshold (typically 40 or higher) or a BMI of 35 or higher with significant obesity-related health conditions. Other requirements might include documentation of previous weight loss attempts, a period of supervised dietary counseling, or medical documentation demonstrating the need for surgery.  Additionally, insurance plans may have specific guidelines regarding the type of bariatric surgery covered, such as gastric bypass, gastric sleeve, or gastric banding. It is important to review the policy details and consult with the insurance provider to understand the specific coverage and requirements.  It's worth noting that insurance coverage can vary widely, and some plans may exclude coverage for bariatric surgery altogether. It is recommended to contact the insurance company directly to understand the coverage details and requirements specific to your plan. Additionally, discussing the matter with a healthcare professional who specializes in bariatric surgery can provide further guidance on insurance coverage and the necessary steps to pursue it. | 6/25/2023 | Certainly! Whether insurance covers bariatric surgery depends on your specific insurance plan and its requirements. In many cases, insurance does cover bariatric surgery, but there are conditions you need to meet.  Insurance companies usually have certain criteria that you must fulfill to be eligible for coverage. These criteria typically include having a high body mass index (BMI) or having obesity-related health issues. You may also need to show proof of previous attempts at losing weight and have medical documentation supporting the need for surgery.  Different insurance plans may cover different types of bariatric surgery, such as gastric bypass or gastric sleeve. It's important to check your policy details or contact your insurance provider to understand what they cover and the specific requirements you need to meet.  It's important to remember that insurance coverage can vary, and some plans may not cover bariatric surgery at all. To find out if your insurance covers it, it's best to speak directly with your insurance company. You can also consult with a healthcare professional who specializes in bariatric surgery for further guidance on insurance coverage and the necessary steps to pursue it. |
| 2 | Can bariatric surgery be reversed? | Laparoscopic gastric banding surgery is reversible in a minimally invasive manner. Gastric bypass surgery is potentially reversible. Reversal requires another operation of the same, or greater, magnitude with the same, or greater, risks. |  | Bariatric surgery, such as gastric bypass or gastric sleeve surgery, is typically considered a permanent procedure. However, in certain circumstances, it is possible to reverse or revise bariatric surgery.  Reversing bariatric surgery is not a common or routine procedure, and it is generally recommended only in specific situations. Some reasons for reversing bariatric surgery may include severe complications, intolerable side effects, or inadequate weight loss.  The feasibility of reversing bariatric surgery depends on the specific type of procedure that was originally performed. For instance, gastric band surgery is relatively easier to reverse compared to gastric bypass or gastric sleeve surgery.  Gastric band removal is a surgical procedure that involves removing the adjustable band placed around the stomach. This procedure can usually be performed laparoscopically. After the removal, the stomach generally returns to its original size and shape.  On the other hand, reversing gastric bypass or gastric sleeve surgery is more complex and may involve converting the procedure to a different type of bariatric surgery or restoring the anatomy to its pre-surgery state. This type of revision surgery is more invasive and carries higher risks than the initial surgery.  It's important to note that bariatric surgery reversal is not a guaranteed solution and should not be taken lightly. It requires careful consideration and consultation with a bariatric surgeon to evaluate the potential risks, benefits, and individual circumstances.  If you have undergone bariatric surgery and are considering reversal, it is crucial to consult with your surgeon or a qualified healthcare professional who can provide personalized advice based on your specific situation. | 6/25/2023 | Certainly! Bariatric surgery is usually considered a permanent procedure, meaning it's not intended to be reversed. However, in some cases, it is possible to undo or change the surgery.  Reversing bariatric surgery is not common, and it's only recommended in certain situations. Some reasons for reversing the surgery could be serious complications, side effects that are too difficult to handle, or not achieving the desired weight loss.  The ability to reverse bariatric surgery depends on the specific type of surgery that was done. For example, removing a gastric band (a device placed around the stomach) is generally easier compared to reversing gastric bypass or gastric sleeve surgery.  Removing a gastric band involves a surgical procedure where the band is taken out. The stomach usually returns to its original size and shape after the removal.  Reversing gastric bypass or gastric sleeve surgery is more complicated. It may involve converting the procedure to a different type of surgery or restoring the stomach to its pre-surgery state. This kind of revision surgery is riskier and more invasive than the initial surgery.  It's important to understand that bariatric surgery reversal is not a guaranteed solution and should be carefully considered. It requires consulting with a bariatric surgeon to assess the potential risks, benefits, and your specific circumstances.  If you've had bariatric surgery and are thinking about reversal, it's crucial to talk to your surgeon or a qualified healthcare professional who can provide personalized advice based on your situation. |
| 2 | What if I need revision surgery? | Weight loss surgery is not a magic bullet and while most patients are successful after weight loss surgery, there are instances where revision weight loss surgery is required. Whether reasons for seeking revision weight loss surgery are due to inadequate weight loss/weight regain, unresolved co-morbidities and/or medical complications, there is possibly a revision solution to these problems.  Revisional bariatric operations, regardless of the previous surgery, are always challenging for a surgeon. They require adequate expertise to achieve desired results. Bariatric Centers of Excellence certified by the American Society for Bariatric Surgery provide a multi-disciplinary team and facilities for managing patients requiring revision of their surgery. It is highly recommended that patients requiring gastric bypass revision or any other kind of revisional surgery find a surgeon at a bariatric center that has much experience with revisional bariatric surgery. | What if I need revision surgery after weight loss surgery? | If you have undergone weight loss surgery and require revision surgery, it is important to consult with your healthcare provider or bariatric surgeon. Revision surgery after weight loss surgery may be necessary for various reasons, such as inadequate weight loss, weight regain, or complications from the initial procedure. Here are a few points to consider:  1. Evaluation: Your healthcare provider will conduct a thorough evaluation to determine the reasons for revision surgery. This may involve assessing your weight loss progress, reviewing your medical history, and conducting diagnostic tests.  2. Reasons for revision: The specific reasons for revision surgery can vary depending on individual circumstances. Some common reasons include inadequate weight loss, complications like band slippage or erosion (for adjustable gastric banding procedures), persistent or recurring health issues, or the need to convert to a different weight loss surgery procedure.  3. Risks and benefits: Your surgeon will discuss the potential risks and benefits associated with revision surgery. It's important to understand that any surgical procedure carries some risks, such as infection, bleeding, anesthesia complications, or complications specific to the type of revision being performed.  4. Preoperative preparations: Prior to the revision surgery, you may need to undergo certain preoperative preparations, which can include dietary modifications, medical assessments, and discussions about the expected outcomes.  5. Surgical options: The specific type of revision surgery will depend on the reasons for the procedure and the original weight loss surgery you had. Some common revision procedures include conversion to a different weight loss surgery technique (e.g., converting from gastric banding to gastric bypass), removal of a previously placed device (e.g., adjustable gastric band removal), or repairing/revising an existing procedure.  6. Postoperative care: Following the revision surgery, you will require careful postoperative care and follow-up. This may involve dietary changes, monitoring for complications, regular check-ups with your healthcare provider, and support from a multidisciplinary team, including nutritionists and mental health professionals.  Remember, each case is unique, and the decision to undergo revision surgery should be made in consultation with your healthcare provider. They will consider various factors, such as your overall health, weight loss goals, and the potential benefits and risks of revision surgery, to guide you in making an informed decision. | 6/25/2023 | Certainly! If you have previously had weight loss surgery and now need another surgery to fix any issues or improve the results, it's important to talk to your doctor or surgeon. Here are a few key points to understand:  1. Evaluation: Your doctor will assess your situation by looking at your weight loss progress, medical history, and doing tests to understand why you need revision surgery.  2. Reasons for revision: There can be different reasons for needing another surgery, such as not losing enough weight, regaining weight, or complications from the first surgery.  3. Risks and benefits: Your surgeon will discuss the potential risks and benefits of the revision surgery. All surgeries have some risks, like infections or bleeding, and there can be specific risks related to the type of revision you need.  4. Preoperative preparations: Before the revision surgery, you might need to make some changes to your diet, undergo medical assessments, and discuss what you can expect after the surgery.  5. Surgical options: The type of revision surgery will depend on why you need it and the original weight loss surgery you had. Some options include converting to a different surgery technique, removing a previously placed device, or repairing/revising the existing procedure.  6. Postoperative care: After the revision surgery, you'll need to take care of yourself and follow the instructions given by your healthcare team. This may involve changes to your diet, monitoring for complications, regular check-ups, and support from nutritionists and mental health professionals.  Remember, it's important to have a discussion with your doctor to understand your specific situation and make an informed decision about revision surgery. They will consider your overall health, weight loss goals, and the potential benefits and risks to guide you through the process. |
| 2 | What is my ideal body weight? | X's Bariatric and Metabolic Institute follows the guidelines set out by the body mass index scale. This scale recommends a BMI of between 18.5 and 24.9. You can use the calculator above to find your current BMI and ideal BMI. |  | Determining your ideal body weight depends on several factors, including your height, age, gender, body composition, and overall health. One commonly used method to estimate ideal body weight is the Body Mass Index (BMI). The BMI is calculated by dividing your weight in kilograms by the square of your height in meters.  However, it's important to note that the BMI has its limitations, as it does not take into account individual variations in muscle mass, bone density, and other factors. Therefore, it's always best to consult with a healthcare professional or a registered dietitian who can provide personalized advice based on your specific circumstances.  If you provide me with your height, gender, age, and any additional relevant information, I can help provide a general range for your ideal body weight. | 6/25/2023 | Certainly! Your ideal body weight is the weight that is considered healthy and appropriate for your body based on factors like your height, age, gender, and overall health. It's a weight range that is associated with good health and well-being.  To estimate your ideal body weight, a common method is to use the Body Mass Index (BMI). The BMI is calculated by dividing your weight (in kilograms) by the square of your height (in meters). The resulting number gives you an indication of whether you are underweight, normal weight, overweight, or obese.  However, keep in mind that the BMI doesn't consider individual differences such as muscle mass and bone density. Therefore, it's always a good idea to consult with a healthcare professional or a registered dietitian who can give you personalized advice based on your specific situation and goals. |
| 2 | Is there support from surgeons and staff following weight loss surgery? | X's gastric bypass surgery health care team is there to guide patients after each procedure. Regular follow-up visits are scheduled during the first year after weight loss surgery to check overall physical and mental health, metabolism and nutritional status. |  | Yes, there is typically support from surgeons and healthcare staff following weight loss surgery. Bariatric surgery, which includes procedures such as gastric bypass, gastric sleeve, and gastric banding, is a significant medical intervention that requires comprehensive care and support both before and after the procedure.  Before surgery, patients typically undergo an extensive evaluation process, which involves consultations with surgeons, dietitians, psychologists, and other healthcare professionals. This evaluation helps ensure that the patient is well-informed about the procedure, understands the potential risks and benefits, and is mentally and physically prepared for the surgery.  After the surgery, a multidisciplinary approach is often adopted to provide comprehensive support. This may include regular follow-up appointments with the surgical team, dietitians, and other healthcare professionals to monitor progress, manage any complications, and provide guidance on dietary and lifestyle changes. The frequency of these follow-up visits may vary depending on the patient's individual needs and the specific surgical procedure performed.  Support may also extend beyond the immediate postoperative period. Many weight loss surgery programs offer ongoing support groups, counseling services, and educational resources to help patients adjust to their new lifestyle, manage their weight loss, and address any emotional or psychological challenges that may arise.  It's important to note that the level of support and specific resources available may vary between healthcare providers and surgical centers. It is advisable to inquire about the postoperative support and resources available when considering weight loss surgery, and choose a provider that offers comprehensive care and assistance throughout the entire process. | 6/25/2023 | Certainly! After weight loss surgery, surgeons and healthcare staff are there to support you throughout your journey. Before the surgery, you'll meet with different healthcare professionals who will help you understand the procedure and make sure you're ready for it.  After the surgery, you'll have regular check-up appointments with the surgical team, dietitians, and other healthcare professionals. They will monitor your progress, help you with any issues or complications, and guide you on making healthy changes to your diet and lifestyle.  You'll also have access to support groups, counseling services, and educational materials to help you adapt to your new lifestyle, manage your weight loss, and address any emotional or psychological challenges that may come up.  The specific support and resources available may vary depending on the healthcare provider or surgical center, so it's important to ask about what kind of support they offer before choosing a provider. |
| 3 | How do I know if I am eligible? | Patients must meet specific criteria before they will be considered for an evaluation with the X Bariatric & Metabolic Surgery program.  - Have a Body Mass Index (BMI) of greater than 40, or a BMI of35 accompanied by other issues related to health (co-morbidities). Calculate your BMI. These co-morbidities include diabetes, high blood pressure, coronary artery disease, obstructive sleep apnea, osteoarthritis, hyperlipidemia (high cholesterol, triglycerides, etc), metabolic syndrome, polycystic ovarian disease, pseudotumor cerebri, etc.  - Be mobile, medically stable, and have an actively involved and supportive primary care physician.  - Have a stable psychological status with adequate social support system to help them through the course of surgery and the postoperative period. | How do I know if I am eligible for bariatric or weight loss surgery? | Determining your eligibility for bariatric or weight loss surgery typically involves a comprehensive evaluation by a healthcare professional specializing in bariatric surgery. While I can provide some general information, please note that it's important to consult with a qualified healthcare provider for an accurate assessment based on your specific circumstances. Here are some factors that are commonly considered:  1. Body Mass Index (BMI): Bariatric surgery is generally recommended for individuals with a BMI of 40 or higher (severe obesity) or a BMI of 35-39.9 (obesity) along with obesity-related health conditions such as diabetes, high blood pressure, or sleep apnea. However, lower BMI thresholds may be considered for certain individuals.  2. Failed Weight Loss Attempts: Bariatric surgery is usually considered for individuals who have made concerted efforts to lose weight through diet, exercise, and lifestyle changes but have not achieved significant and sustainable weight loss.  3. Obesity-related Health Conditions: If you have obesity-related health conditions like type 2 diabetes, high blood pressure, heart disease, sleep apnea, or mobility issues, bariatric surgery may be an option to improve these conditions.  4. Psychological Evaluation: A psychological assessment is often conducted to evaluate your readiness for surgery and ensure that you have realistic expectations, understand the potential risks and benefits, and can comply with the necessary lifestyle changes.  5. Age and Medical History: Age is not a sole determining factor, but your overall medical history, including any previous surgeries, current medications, and pre-existing conditions, will be taken into account when evaluating your eligibility.  6. Commitment to Lifestyle Changes: Bariatric surgery is not a quick fix but a tool that requires lifelong commitment to dietary changes, regular physical activity, and follow-up care. Your willingness and ability to make these lifestyle changes will be assessed.  It's essential to have an open and honest discussion with a qualified bariatric surgeon or healthcare professional who can evaluate your individual circumstances and provide personalized advice. They will guide you through the process, explain the potential risks and benefits, and help you make an informed decision about whether bariatric surgery is suitable for you. | 6/25/2023 | Certainly! Here are the main points in simpler terms:  1. Body Mass Index (BMI): Bariatric surgery is considered for people who have a high BMI (a measure of weight in relation to height). Generally, if your BMI is 40 or higher or between 35 and 39.9 with obesity-related health problems, you may be eligible.  2. Failed Weight Loss Attempts: If you've tried different methods to lose weight, like diets and exercise, without success, bariatric surgery may be an option.  3. Obesity-related Health Conditions: If you have health problems such as diabetes, high blood pressure, heart disease, or sleep apnea, and they are related to your weight, surgery might help improve those conditions.  4. Psychological Evaluation: You may need to undergo a psychological assessment to make sure you're mentally prepared for surgery, have realistic expectations, and can handle the necessary lifestyle changes.  5. Age and Medical History: Your age and medical background will be considered, including any previous surgeries, medications you're taking, and other existing health conditions.  6. Commitment to Lifestyle Changes: Bariatric surgery is not a quick fix. It's important that you're ready to make long-term changes to your diet, exercise, and overall lifestyle after surgery.  Remember, it's crucial to talk to a qualified bariatric surgeon or healthcare professional who can assess your specific situation and provide guidance based on your needs and health status. |
| 3 | Do I have to wait to get pregnant after bariatric surgery? Will the baby be healthy? | Women generally have significantly increased fertility after weight loss surgery. Birth control pills do NOT work as well in heavy patients and during the time of rapid weight loss. We strongly recommend all child bearing age women to use other ways of contraception after bariatric surgery.  Most surgeons recommend waiting 12-18 months after weight loss surgery before getting pregnant. After losing most of the excess weight, there is much less risk of having problems during pregnancy (gestational diabetes, eclampsia, macrosomia) and during childbirth. There is also decreased risk of having miscarriages and stillbirths. The kids born after mon’s bariatric surgery are less prone to being obese later in their life. Bariatric surgery also reduces the chance of needing a C-section. |  | If you've recently undergone bariatric surgery and are considering getting pregnant, it's important to consult with your healthcare provider for personalized advice. However, I can provide you with some general information.  After bariatric surgery, such as gastric bypass or gastric sleeve surgery, it's generally recommended to wait for a period of time before trying to conceive. The specific duration of the waiting period can vary depending on the type of surgery and individual factors, so it's essential to discuss this with your surgeon or healthcare team. This waiting period allows your body to heal, adjust to the changes in anatomy and physiology, and reach a more stable weight.  Pregnancy after bariatric surgery is usually possible and can be safe, provided you follow appropriate medical guidance. However, there are a few considerations to keep in mind:  1. Nutritional status: Bariatric surgery can affect nutrient absorption and may require careful monitoring of nutritional intake, especially during pregnancy. Adequate nutrition is crucial for both your health and the development of the baby.  2. Weight management: Maintaining a healthy weight gain during pregnancy is important for your well-being and the baby's health. Your healthcare provider can guide you on appropriate weight gain goals based on your individual circumstances.  3. Vitamin and mineral supplementation: Bariatric surgery can impact the absorption of certain vitamins and minerals, such as vitamin B12, iron, calcium, and folic acid. Your healthcare provider will likely recommend specific supplements to ensure you meet the necessary nutrient requirements during pregnancy.  4. Potential risks: While the majority of pregnancies after bariatric surgery are successful, some studies have shown a slightly increased risk of certain complications, such as preterm birth and gestational diabetes. Regular prenatal care and close monitoring by your healthcare team are crucial to identify and manage any potential risks.  Remember, every individual is unique, and the guidance provided by your healthcare team will be tailored to your specific situation. It's important to consult with them to ensure a safe and healthy pregnancy after bariatric surgery. | 6/25/2023 | Certainly! After bariatric surgery (weight loss surgery), it's generally recommended to wait for a while before getting pregnant. This gives your body time to heal and adjust to the changes from the surgery. The specific waiting period will depend on the type of surgery and your individual circumstances, so it's best to talk to your doctor.  Once you're ready to get pregnant, it is usually possible and safe, but there are a few things to consider:  1. Nutrition: Bariatric surgery can affect how your body absorbs nutrients, so it's important to make sure you're getting enough vitamins and minerals, especially during pregnancy. Your doctor may recommend specific supplements to help with this.  2. Weight management: It's important to maintain a healthy weight gain during pregnancy. Your doctor will guide you on how much weight you should aim to gain based on your situation.  3. Potential risks: While most pregnancies after bariatric surgery go well, there may be a slightly higher risk of certain complications like preterm birth or gestational diabetes. Regular check-ups with your doctor during pregnancy will help monitor for any potential issues.  Remember, it's important to talk to your doctor about your plans to get pregnant after bariatric surgery. They will provide personalized advice and guidance based on your specific needs and situation to ensure a safe and healthy pregnancy. |
| 3 | How long will I stay in the hospital after bariatric surgery? | All surgeries are performed at the new X Medical Center. The average surgical time for gastric bypass is about 2 hours and sleeve gastrectomy is about 1-2 hours. The laparoscopy totally changed bariatric surgery. Almost all our patients can walk a few hours after surgery. They start to have liquids the day after surgery. The majority (more than 95%) of patients only stay in the hospital for one night or two nights. |  | The length of your hospital stay after bariatric surgery can vary depending on several factors, including the specific surgical procedure performed, your overall health, and any potential complications that may arise during or after the surgery. In general, the hospital stay for bariatric surgery typically ranges from one to four days.  Minimally invasive procedures such as laparoscopic gastric bypass or gastric sleeve surgery often result in shorter hospital stays, usually around one to two days. These procedures involve smaller incisions and generally have quicker recovery times.  On the other hand, more invasive surgeries or surgeries involving additional procedures may require a longer hospital stay. For example, if you undergo a revision surgery or a complex procedure like a duodenal switch, your hospital stay could be extended to three or four days.  It's important to note that the length of your hospital stay is determined by your medical team based on your individual needs and progress during recovery. They will closely monitor your condition and make decisions regarding your discharge based on various factors such as pain management, wound healing, ability to tolerate oral intake, and overall recovery.  Please consult with your healthcare provider or surgeon for a more accurate estimate based on your specific circumstances. They will be able to provide you with the most relevant information based on their expertise and your medical history. | 6/25/2023 | Certainly! After bariatric surgery, the time you'll spend in the hospital can vary. It depends on factors like the type of surgery you have, your overall health, and if any complications arise. Typically, people stay in the hospital for one to four days.  If you have a less invasive procedure like laparoscopic gastric bypass or gastric sleeve surgery, you may stay for one to two days. These surgeries involve smaller incisions and usually have quicker recoveries.  However, if you have a more complex surgery or need additional procedures, your hospital stay may be longer. For example, revision surgeries or procedures like duodenal switch could require three to four days in the hospital.  Ultimately, your medical team will decide how long you need to stay based on your progress and individual circumstances. They'll consider factors like pain management, wound healing, ability to eat, and overall recovery.  It's always best to consult with your healthcare provider or surgeon for a more accurate estimate based on your specific situation. They have the expertise to give you the most relevant information based on your medical history. |
| 3 | When can I go back to work after surgery? | After surgery, most patients return to work after two to four weeks. Some may be able to work from home as early as one week after surgery. Because of the significantly reduced calorie intake, many patients have low energy for 1-2 weeks after surgery. However, most patients gain their energy back once they start to have soft food. | When can I go back to work after weight loss surgery? | The specific timeframe for returning to work after weight loss surgery can vary depending on several factors, including the type of procedure you undergo, the extent of the surgery, your overall health, and the nature of your job. It is crucial to consult with your healthcare team, including your surgeon, to get personalized advice tailored to your situation. They will be able to provide you with specific guidelines based on your progress and recovery.  In general, the recovery period for weight loss surgery can range from a few days to several weeks. During this time, your body needs to heal, and you will gradually adjust to changes in your diet and lifestyle. Initially, you may experience some discomfort, fatigue, and dietary restrictions.  For less invasive weight loss procedures, such as laparoscopic surgeries, some individuals can return to work within a week or two. However, for more complex procedures like gastric bypass or sleeve gastrectomy, a longer recovery period of two to four weeks may be necessary.  Factors to consider when determining your readiness to return to work include:  1. Physical recovery: Ensure that your surgical incisions have healed properly, and you have regained enough strength and mobility to perform your job without undue strain.  2. Energy levels: Weight loss surgery can initially cause fatigue as your body adjusts to a reduced calorie intake. Make sure you have regained enough energy to sustain a regular workday.  3. Follow-up appointments: Attend all necessary follow-up visits with your surgeon to monitor your progress and receive their approval before returning to work.  4. Job requirements: Consider the physical demands of your job. If it involves heavy lifting, strenuous physical activity, or prolonged standing, you may require a longer recovery period or modifications to your work duties.  Remember, every individual's recovery is unique, and it's crucial to prioritize your health and well-being during this period. Follow the guidance of your medical team, listen to your body, and gradually ease back into your work routine to ensure a successful return. | 6/25/2023 | Certainly! After weight loss surgery, the time it takes to go back to work can vary. It depends on the type of surgery, how extensive it was, your overall health, and the kind of work you do.  It's important to talk to your surgeon or healthcare team about when it's safe for you to return to work. They will consider your specific circumstances and give you personalized advice.  Generally, the recovery period can range from a few days to a few weeks. During this time, your body needs to heal, and you'll be adjusting to changes in your diet and lifestyle.  For less invasive surgeries, like laparoscopic procedures, some people can go back to work within a week or two. But for more complex surgeries, like gastric bypass or sleeve gastrectomy, it may take two to four weeks before you can return to work.  There are a few things to think about when deciding if you're ready to go back to work:  1. Physical recovery: Make sure your surgical incisions have healed properly and you've regained enough strength and movement to do your job comfortably.  2. Energy levels: Weight loss surgery can make you feel tired at first as your body adjusts to eating less. Make sure you have enough energy to get through a regular workday.  3. Doctor visits: Keep up with your follow-up appointments to monitor your progress. Your surgeon will let you know when it's okay to go back to work.  4. Job requirements: Consider the physical demands of your job. If it involves heavy lifting, lots of physical activity, or standing for a long time, you might need more time to recover or adjustments to your duties.  Remember, everyone's recovery is different. It's important to take care of yourself and listen to your body. Gradually ease back into your work routine to ensure a successful return. |
| 3 | How bad is the pain after surgery? | Most patients have some pain after surgery. However, the pain is usually just a little bit more than laparoscopic gallbladder surgery. Most patients just need to take oral pain medications for a few days after surgery. The majority of our patients felt that pain was not a major issue during their recovery. | How bad is the pain after weight loss surgery? | Weight loss surgery, also known as bariatric surgery, is a significant medical procedure that aims to help individuals achieve weight loss and manage obesity-related health conditions. The experience of pain after weight loss surgery can vary from person to person, and it depends on several factors, including the specific type of surgery performed, individual pain tolerance, and the overall recovery process.  Immediately after surgery, it is common to experience some level of pain or discomfort. The incision sites and the surgical area may be sore, and you may also feel some internal discomfort due to the alteration of your digestive system. Your healthcare team will provide you with pain management strategies, including medication, to help alleviate your discomfort during the initial recovery period.  In the days and weeks following the surgery, the pain typically decreases gradually as the body heals. However, it's important to note that weight loss surgery is a major surgical procedure, and some level of pain or discomfort can persist for several weeks or even months as the body adjusts to the changes. It's essential to follow your surgeon's post-operative instructions, including proper wound care, pain medication management, and gradual reintroduction of activities and diet.  While pain is a normal part of the recovery process, it's crucial to communicate any excessive or worsening pain to your healthcare team. They can assess your condition and ensure that you receive appropriate care and support throughout your recovery journey.  Remember, each person's experience with weight loss surgery and the associated pain can differ. It is best to consult with your healthcare provider or surgeon for personalized information and guidance regarding your specific situation. | 6/25/2023 | Certainly! After weight loss surgery, it's common to experience some pain or discomfort. This can vary from person to person and depends on factors like the type of surgery and your own pain tolerance.  Right after the surgery, you may feel soreness and discomfort in the areas where the surgeon made incisions and in your digestive system. Your medical team will give you medicine to help manage the pain during your recovery.  As time goes on, the pain will usually get better as your body heals. However, since weight loss surgery is a major procedure, you may still have some pain for weeks or even months as your body adjusts to the changes. It's important to follow your surgeon's instructions for taking care of yourself and managing the pain.  If you have any concerns about your pain levels, it's important to talk to your healthcare team. They can assess your situation and make sure you're getting the right care and support.  Remember, everyone's experience with weight loss surgery and pain is different. It's best to talk to your healthcare provider or surgeon for personalized information and guidance based on your specific situation. |
| 3 | When can I start exercising again after surgery? | As soon as patients totally wake up from anesthesia, we encourage patients to walk. The key is to start slow and listen to your body. If you lift weights or do sports, stay “low impact” for the first month (avoid competition, think participation). If you experience pain or discomfort, stop and relax. Avoid lifting heavy objects for the first 6 weeks. If you swim, your wounds need to be healed over before you get back in the water and always swim slowly at the beginning. | When can I start exercising again after weight loss surgery? | After weight loss surgery, it's essential to follow the guidance and instructions provided by your healthcare team, as they will have specific recommendations tailored to your individual situation. Generally, the timeline for resuming exercise after weight loss surgery can vary depending on the type of surgery performed, your overall health, and any complications or factors that may influence your recovery.  In the early stages following weight loss surgery, your body needs time to heal and adjust to the changes. During this period, your surgeon or healthcare team will likely advise you to focus on rest and recovery. They may encourage you to engage in light activities such as walking to promote circulation and prevent blood clots, but rigorous exercise is typically not recommended during this initial healing phase.  The specific timeframe for resuming exercise will vary, but in many cases, individuals can begin gradually incorporating exercise into their routine around 4 to 6 weeks after surgery. However, this can vary, and it's crucial to consult with your healthcare team to determine the appropriate timing for you. They will consider factors such as the type of surgery you underwent, your overall health, any specific post-operative complications, and your individual progress.  Your healthcare team will provide guidance on the types of exercises that are suitable for you and how to gradually increase the intensity and duration as you regain strength and endurance. They may recommend a combination of aerobic activities, strength training, and flexibility exercises to help you maintain muscle mass, enhance cardiovascular fitness, and improve overall mobility.  Remember to communicate openly with your healthcare team about your desire to start exercising again, and they will guide you through the process based on your specific circumstances. | 6/25/2023 | Certainly! After weight loss surgery, it's important to listen to your doctor's advice and follow their instructions regarding when you can start exercising again. The exact timing can vary based on factors like the type of surgery you had and your overall health.  Right after the surgery, your body needs time to heal and adjust to the changes. During this time, your doctor will likely recommend focusing on resting and recovering. They may suggest light activities like walking, but not intense exercise.  In general, most people can start adding exercise back into their routine around 4 to 6 weeks after surgery. However, this can differ for each person, so it's essential to talk to your doctor about the best time for you to start exercising again.  Your doctor will also provide guidance on the types of exercises that are suitable for you. They may recommend a combination of activities like walking, strength training, and stretching to help you build muscle, improve your heart health, and increase flexibility.  Remember to talk to your doctor about your desire to start exercising, and they will give you specific advice based on your situation. |
| 3 | Do most patients need to have plastic surgery after weight loss? | After losing most of the excess weight, most patients may have some loose or sagging skin.  For many patients, this might be just temporary. The degree of loose or sagging skin depends upon several things, including how much weight you lose, your age, your genetics and whether or not you exercise or smoke.  Generally, many patients can hide loose skin by clothing or compression garments.  We generally recommend wait for at least 18 months before having plastic surgery.  Plastic surgery to remove excess abdominal and breast skin is often covered by insurance because it may cause rash.  You may have to pay yourself for the areas such as arms because it is considered cosmetic by your insurer. | Do most patients need to have plastic surgery after weight loss following weight loss surgery? | The need for plastic surgery after weight loss surgery, such as gastric bypass or gastric sleeve surgery, varies from patient to patient. While significant weight loss can lead to improvements in health and overall appearance, it can also result in excess sagging skin and tissue due to the loss of volume. Plastic surgery procedures, such as body contouring, are often considered by patients who want to address these aesthetic concerns.  It's important to note that not all weight loss surgery patients require or choose to undergo plastic surgery. The decision to pursue plastic surgery after weight loss is highly individual and depends on factors such as the amount of weight lost, individual body composition, age, genetics, and personal preferences.  Excess skin can be a common issue after substantial weight loss, particularly when there is a rapid loss of weight. It can occur in various areas of the body, including the abdomen, arms, thighs, buttocks, and breasts. Some patients may find that the excess skin causes discomfort, limits physical activities, or affects their self-confidence and body image. In such cases, plastic surgery can help remove the excess skin and reshape the body contours.  Procedures commonly considered after weight loss surgery include:  1. Tummy tuck (abdominoplasty): Removes excess skin and fat from the abdomen while tightening the underlying muscles.  2. Arm lift (brachioplasty): Addresses sagging skin in the upper arms.  3. Thigh lift (thighplasty): Removes excess skin from the inner or outer thighs.  4. Breast lift (mastopexy): Raises and reshapes the breasts to a more youthful position.  5. Body lift: Targets multiple areas, such as the abdomen, buttocks, hips, and thighs, in one comprehensive procedure.  It's crucial for weight loss surgery patients to have realistic expectations and consult with a qualified plastic surgeon to discuss their specific goals, concerns, and potential surgical options. Plastic surgery should be approached as a personal choice and not an obligatory step following weight loss surgery. | 6/25/2023 | Certainly! After weight loss surgery, some patients may have excess sagging skin and tissue due to the loss of weight and volume. This can happen in different parts of the body like the abdomen, arms, thighs, buttocks, and breasts.  Plastic surgery, also known as body contouring, is an option for patients who want to address these aesthetic concerns. It involves procedures like tummy tucks, arm lifts, thigh lifts, breast lifts, and body lifts. These surgeries help remove the excess skin and reshape the body to improve its appearance.  However, not all weight loss surgery patients need or choose to have plastic surgery. The decision to have plastic surgery after weight loss is personal and depends on factors like the amount of weight lost, individual body composition, age, genetics, and personal preferences. Some patients may experience discomfort or have body image concerns due to excess skin, which may lead them to consider plastic surgery.  It's important for patients to have realistic expectations and consult with a qualified plastic surgeon to discuss their goals, concerns, and available surgical options. Plastic surgery is not mandatory after weight loss surgery and should be seen as a personal choice. |
| 3 | Will I lose hair after bariatric surgery? | For the reasons we still don’t fully understand, hair loss after bariatric surgery is common between 3 and 6 months following surgery. However, hair loss is almost always temporary. Adequate intake of protein, vitamins, biotin and minerals will help to ensure hair re-growth, and avoid longer term thinning. |  | Hair loss is a potential side effect of bariatric surgery, but it is usually temporary and reversible. It is commonly known as telogen effluvium, which is a type of hair loss that occurs due to a disturbance in the hair growth cycle. The exact cause of hair loss after bariatric surgery is not fully understood, but it is believed to be related to the rapid weight loss and changes in nutritional status following the procedure.  After bariatric surgery, your body goes through significant changes, including a reduced calorie intake, altered hormone levels, and potential nutrient deficiencies. These factors can disrupt the normal hair growth cycle, leading to excessive shedding of hair. The hair loss usually begins around three to six months after surgery and can continue for several months before eventually resolving on its own.  It's important to note that not everyone who undergoes bariatric surgery will experience hair loss, and the severity of hair loss can vary from person to person. Additionally, taking certain precautions and maintaining a healthy lifestyle can help minimize the extent of hair loss. Here are a few tips to support hair health after bariatric surgery:  1. Ensure proper nutrition: Follow the dietary guidelines provided by your healthcare team to meet your nutritional needs. This may include taking vitamin and mineral supplements, such as biotin, zinc, iron, and B-complex vitamins, which can support hair health.  2. Protein intake: Consume an adequate amount of protein, as it is essential for hair growth. Incorporate lean protein sources like poultry, fish, eggs, dairy, legumes, and tofu into your diet.  3. Stay hydrated: Drink plenty of water throughout the day to maintain hydration, which is vital for overall health, including hair health.  4. Avoid crash diets: Rapid and extreme weight loss can contribute to hair loss. It's important to aim for a gradual, steady weight loss instead of trying crash diets or extreme calorie restrictions.  5. Be patient: Hair loss after bariatric surgery is typically temporary. It may take several months for your hair to start regrowing and returning to its normal thickness. In the meantime, be patient and focus on overall health and well-being.  If you have concerns about hair loss after bariatric surgery, it's recommended to discuss them with your healthcare provider. They can provide personalized advice and monitor your nutritional status to minimize any potential complications. | 6/25/2023 | Sure! After bariatric surgery, some people may experience temporary hair loss. This happens because the surgery and the changes in your body can disrupt the normal hair growth cycle. The hair loss usually starts a few months after surgery and can last for several months before it gets better on its own.  Here are some things you can do to take care of your hair after bariatric surgery:  1. Eat a balanced diet: It's important to follow the diet recommended by your healthcare team. They may suggest taking certain vitamins and minerals that can help your hair grow back.  2. Get enough protein: Eating foods that are rich in protein, like poultry, fish, eggs, dairy, legumes, and tofu, can help your hair grow stronger.  3. Stay hydrated: Drink plenty of water throughout the day to keep your body and hair healthy.  4. Avoid extreme diets: Losing weight too quickly can make hair loss worse. It's better to aim for a gradual and steady weight loss instead of trying crash diets.  5. Be patient: Hair loss after bariatric surgery is usually temporary. It may take a few months for your hair to start growing back, so try to be patient and take care of your overall health in the meantime.  If you're worried about hair loss after bariatric surgery, it's a good idea to talk to your doctor. They can give you personalized advice and make sure you're getting the right nutrients to support hair regrowth. |
| 3 | Do I need to take vitamins and minerals after surgery? | You will need to take a multivitamin for life. You may need higher doses of certain vitamins or minerals, especially Iron, Calcium, and Vitamin D. You will also need to have at least yearly lab checks. Insurance almost never pays for vitamin and mineral supplements but usually does pay for labs. You can pay for supplements out of a flex medical account. | Do I need to take vitamins and minerals after weight loss surgery? | After weight loss surgery, such as gastric bypass or gastric sleeve surgery, it is common for individuals to experience changes in their nutritional needs. These procedures can significantly impact the absorption and digestion of nutrients, leading to potential deficiencies. As a result, many healthcare professionals recommend vitamin and mineral supplementation following weight loss surgery.  The specific supplementation requirements may vary depending on the type of surgery, individual factors, and your healthcare provider's recommendations. However, some common vitamin and mineral supplements often recommended after weight loss surgery include:  1. Multivitamin: A high-potency multivitamin is typically recommended to help cover a broad range of nutrient needs. It should include vitamins and minerals such as vitamin D, vitamin B12, iron, zinc, and others.  2. Calcium and Vitamin D: These nutrients are crucial for bone health. Since the body's ability to absorb calcium may be reduced after weight loss surgery, supplementation is often necessary.  3. Iron: Iron deficiency is common after weight loss surgery and can lead to anemia. Iron supplementation may be required, and it is usually taken in a separate form from the multivitamin, as calcium can interfere with iron absorption.  4. Vitamin B12: Due to changes in the digestive system, vitamin B12 absorption may be compromised. Supplementation, often in the form of sublingual tablets or injections, is typically necessary.  5. Other Minerals: Depending on individual needs, supplements such as zinc, copper, selenium, and magnesium may be recommended.  It is important to note that the specific supplementation guidelines can vary among different surgical procedures and individual circumstances. Therefore, it is crucial to consult with your healthcare provider or a registered dietitian who specializes in weight loss surgery to determine your specific nutrient needs and develop a personalized supplement plan.  Regular monitoring of nutrient levels through blood tests is often recommended to assess any deficiencies or imbalances and make appropriate adjustments to your supplementation regimen. | 6/25/2023 | Certainly! After weight loss surgery, your body's ability to absorb and digest nutrients may change. This can lead to deficiencies in vitamins and minerals. To ensure you're getting the nutrients your body needs, it is commonly recommended to take certain supplements after weight loss surgery.  Here are the main supplements often suggested:  1. Multivitamin: This covers a wide range of nutrients in one pill. It usually includes vitamins like D and B12, as well as minerals like iron and zinc.  2. Calcium and Vitamin D: These are important for keeping your bones healthy. Since your body may have trouble absorbing calcium after surgery, you'll likely need supplements.  3. Iron: Low iron levels can lead to anemia, so iron supplementation is often needed after weight loss surgery. It's usually taken separately from the multivitamin.  4. Vitamin B12: Your body may have difficulty absorbing enough B12, so additional supplementation is often necessary. It can come in the form of sublingual tablets or injections.  5. Other Minerals: Depending on your individual needs, your healthcare provider may recommend supplements like zinc, copper, selenium, and magnesium.  It's important to work closely with your healthcare provider or a registered dietitian who specializes in weight loss surgery. They can assess your specific needs and develop a personalized plan for you. Regular blood tests will help monitor your nutrient levels and make any necessary adjustments to your supplement regimen. |
|  | If my insurance company will not pay for the surgery, can I pay the surgery myself? | We do offer bariatric surgery for patients who want to pay cash. Please call our office to get a quote. | If my insurance company will not pay for weight loss surgery, can I pay the surgery myself? | Yes, if your insurance company will not cover weight loss surgery or any other medical procedure, you have the option to pay for the surgery yourself if you can afford it. This is commonly referred to as "self-pay" or "out-of-pocket" payment.  Weight loss surgery can be costly, so it's essential to consider the financial implications before making this decision. Here are some steps to consider if you're thinking about paying for weight loss surgery yourself:  1. Consult with a healthcare professional: Before deciding on weight loss surgery, it's crucial to talk to a qualified healthcare professional, such as a bariatric surgeon, to understand your options, the potential risks, benefits, and whether you are a suitable candidate for the procedure.  2. Obtain a cost estimate: Get a detailed cost estimate from the hospital or clinic where you plan to have the surgery. This will include the surgeon's fees, facility fees, anesthesia, and any additional costs related to pre-operative evaluations and post-operative care.  3. Consider financing options: If paying the full amount upfront is not feasible for you, inquire about financing options offered by the healthcare facility. Some hospitals or clinics may have arrangements for payment plans or loans designed specifically for medical expenses.  4. Research tax deductions or credits: In some cases and depending on your country's tax laws, medical expenses (including weight loss surgery) that exceed a certain percentage of your income may be tax-deductible or eligible for tax credits. Consult a tax professional to understand the tax implications.  5. Weigh the risks and benefits: Apart from the financial aspect, consider the potential health benefits and risks associated with weight loss surgery. Make an informed decision based on your overall health and well-being.  6. Explore other insurance options: If your current insurance doesn't cover weight loss surgery, check if there are other insurance plans available in your area that do cover it. However, keep in mind that some insurance policies may have waiting periods or exclusions for pre-existing conditions.  7. Seek advice from support groups: Reach out to support groups for people considering or who have undergone weight loss surgery. They can provide valuable insights and personal experiences to help you in your decision-making process.  Remember that weight loss surgery is a significant medical procedure that requires careful consideration and consultation with healthcare professionals. It is not a decision to be taken lightly, and your health should always be the top priority. | 6/25/2023 | Of course! If your insurance company won't pay for weight loss surgery, you can choose to pay for the surgery yourself. It means you'll be responsible for covering all the costs related to the procedure. Here are some things you can do:  1. Talk to a doctor: Before making any decisions, talk to a doctor who specializes in weight loss surgery. They can help you understand if it's the right option for you.  2. Find out the cost: Figure out how much the surgery will cost. This includes the surgeon's fees, hospital charges, and other related expenses.  3. Consider payment options: If paying everything at once is too expensive, ask if you can set up a payment plan with the hospital or clinic. This way, you can pay in smaller amounts over time.  4. Check tax benefits: In some cases, you might get tax deductions or credits for medical expenses. Ask a tax expert to see if this applies to you.  5. Think about the risks and benefits: Make sure you understand the potential health benefits and risks of the surgery before making a decision.  6. Look for other insurance options: Check if there are other insurance plans that cover weight loss surgery. However, remember that some plans might have waiting periods or not cover certain conditions.  7. Seek support: Talk to others who have gone through weight loss surgery or are considering it. They can provide valuable advice and insights.  Remember, weight loss surgery is a big decision, so take your time to think about it carefully and talk to the right people to make an informed choice. |
| 3 | Will bariatric surgery last? | Many patients (up to 50%) may regain a small amount of weight (approximately 5 percent) two years or more following their surgery.  However, longitudinal studies find that most bariatric surgery patients maintain successful weight-loss long-term.  One study summarizing the results from 22 reports with a total of 4206 patient cases reported good long-term durability of gastric bypass surgery.  The average excess weight loss was 66.5%, and there was no significant association between excess weight loss and length of follow-up.  Now we have data to support the durability of gastric bypass surgery up to 15 years.  Studies also have shown that sleeve gastrectomy patients were able to maintain its well-documented weight loss outcome at 5 or more years after surgery, with the overall mean excess weight loss at 5 or more years still remaining>50%.  Such significant and sustained weight loss after surgery is in sharp contrast to medical management |  | Bariatric surgery is a medical procedure that aims to assist individuals with significant weight loss by altering the digestive system. While bariatric surgery can be an effective tool for weight management, it's important to understand that its long-term success depends on various factors.  The effectiveness of bariatric surgery can vary from person to person. Generally, it has been shown to produce significant and sustained weight loss for many individuals. However, the long-term success of the surgery is influenced by factors such as the patient's commitment to lifestyle changes, including dietary modifications and regular physical activity.  It's crucial to note that bariatric surgery is not a standalone solution. It is typically recommended as part of a comprehensive treatment plan that includes ongoing medical supervision, nutritional counseling, and psychological support. These elements are essential for maintaining weight loss and overall well-being after the surgery.  While bariatric surgery can yield long-term benefits, it's important for individuals to understand that it requires a commitment to lifestyle changes and ongoing medical care. Regular follow-ups with healthcare professionals and adherence to recommended guidelines are crucial to ensure the lasting success of bariatric surgery. | 6/25/2023 | Certainly! Bariatric surgery is a medical procedure that helps people who are severely overweight lose weight. It changes the way their digestive system works to help them eat less and feel full faster.  However, the success of bariatric surgery in the long run depends on a few things. First, it requires the person to make lasting changes in their lifestyle, like eating healthier and being more active. Simply having the surgery isn't enough; they need to commit to these changes.  Bariatric surgery is usually part of a bigger plan for weight management. This includes ongoing medical check-ups, getting advice on what to eat, and getting support for any emotional challenges that may arise.  While bariatric surgery can be effective, it's important to understand that it's not a quick fix. It requires ongoing effort and support to maintain the weight loss and overall health benefits. Regular check-ups with doctors and following the recommended guidelines are important to make sure the surgery has lasting effects. |
| 4 | Do I qualify for weight loss surgery? | Patients will meet with a Mass General care team to evaluate their overall health and candidacy for bariatric surgery. Generally, patients must meet the following requirements to be eligible for weight loss surgery:  - Have a body mass index (BMI) of 35 or higher  - Have a BMI between 30 and 35 in conjunction with a weight-related comorbidity, such as heart disease, diabetes, or high blood pressure  - Have a stable psychological status | How do I qualify for weight loss surgery? | Qualifying for weight loss surgery, also known as bariatric surgery, typically involves meeting certain criteria. However, it's important to note that specific requirements may vary depending on the country, healthcare provider, and type of weight loss surgery. Here are some general guidelines often considered by medical professionals:  1. Body Mass Index (BMI): Your BMI is a measurement of your body fat based on your height and weight. Generally, individuals with a BMI of 40 or higher, or a BMI of 35-39.9 with obesity-related health conditions, may be eligible for weight loss surgery. In some cases, people with a BMI of 30-34.9 and significant obesity-related health issues may also be considered.  2. Failed attempts at non-surgical weight loss: You may need to demonstrate that you have made sustained efforts to lose weight through non-surgical methods, such as lifestyle changes, diet, and exercise. This may involve documentation of previous weight loss attempts and participation in supervised weight loss programs.  3. Obesity-related health conditions: Having obesity-related health issues, such as type 2 diabetes, sleep apnea, high blood pressure, or heart disease, may increase your chances of qualifying for weight loss surgery. These conditions often improve or resolve after weight loss surgery.  4. Age requirements: There may be minimum and maximum age limits for weight loss surgery, typically ranging from 18 to 65 years old. These age limits can vary depending on the specific surgical procedure and healthcare provider's guidelines.  5. Psychological evaluation: Weight loss surgery can have significant physical and emotional impacts. Some healthcare providers may require a psychological evaluation to assess your readiness for surgery and ensure you have realistic expectations and the necessary support systems in place.  6. Commitment to post-surgery lifestyle changes: Weight loss surgery is not a quick fix. It requires a lifelong commitment to dietary changes, regular exercise, and ongoing medical follow-up. Healthcare providers may assess your willingness and ability to make these long-term changes.  It's important to consult with a qualified healthcare professional, such as a bariatric surgeon or a weight loss specialist, who can evaluate your specific situation, provide guidance, and determine your eligibility for weight loss surgery based on the available options in your area. | 6/25/2023 | Certainly! To qualify for weight loss surgery, you generally need to meet certain criteria. Here are the main points:  1. Body Mass Index (BMI): Your BMI is a measure of body fat based on your height and weight. If your BMI is 40 or higher, or if it's between 35 and 39.9 with obesity-related health problems, you may be eligible for weight loss surgery. Sometimes, people with a BMI of 30-34.9 and significant health issues may also qualify.  2. Previous attempts at losing weight: You may need to show that you have tried to lose weight through methods like lifestyle changes, diet, and exercise before considering surgery. This could involve providing documentation of your weight loss attempts and participating in supervised weight loss programs.  3. Health conditions related to obesity: If you have health problems like type 2 diabetes, sleep apnea, high blood pressure, or heart disease related to obesity, it can increase your chances of qualifying for weight loss surgery. These conditions often improve or go away after surgery.  4. Age requirements: There may be minimum and maximum age limits for weight loss surgery, usually between 18 and 65 years old. The specific limits can vary depending on the surgery and the healthcare provider.  5. Psychological evaluation: Weight loss surgery can have both physical and emotional effects. Some providers may want to evaluate your mental well-being to make sure you're ready for the surgery and have realistic expectations. They may also check if you have the necessary support systems in place.  6. Commitment to lifestyle changes after surgery: Weight loss surgery is not a quick fix. You need to be committed to making long-term changes in your diet, exercise, and medical follow-up. Providers will want to make sure you're ready to make these changes and stick with them.  It's important to consult with a qualified healthcare professional, like a bariatric surgeon or weight loss specialist, who can evaluate your specific situation, guide you, and determine your eligibility based on the available options in your area. |
| 4 | Will insurance cover bariatric surgery? | Many insurance companies do cover weight loss surgery as long as you meet the qualification requirements. To learn more about insurance coverage, call our Weight Center staff at 617-726-4400. |  | The coverage for bariatric surgery, like any other medical procedure, can vary depending on several factors. Insurance coverage for bariatric surgery is influenced by the specific policy you have and the guidelines set by the insurance company. It is essential to review your insurance policy or contact your insurance provider directly to determine the coverage details.  In many cases, insurance companies may cover bariatric surgery, but they often have specific requirements that need to be met before approval. These requirements commonly include:  1. Documentation of a body mass index (BMI) above a certain threshold (usually 40 or 35 with obesity-related health conditions).  2. Evidence of previous attempts at non-surgical weight loss methods under medical supervision.  3. Completion of a comprehensive evaluation process, including psychological and medical evaluations.  4. Demonstrating a commitment to long-term lifestyle changes and participation in post-surgical care programs.  5. Insurance companies may also have specific criteria for the type of bariatric surgery they cover, such as gastric bypass, gastric sleeve, or gastric banding. Some policies may cover all approved procedures, while others might only cover certain types.  It is crucial to be aware that pre-authorization from your insurance company is typically required before proceeding with bariatric surgery. This involves submitting all the necessary documentation and obtaining approval from the insurance provider.  To get accurate and up-to-date information about your specific insurance coverage for bariatric surgery, it is best to consult your insurance policy documents or contact your insurance provider directly. They will be able to provide you with the most relevant details regarding your coverage and any specific requirements you need to meet. | 6/25/2023 | Certainly! Insurance coverage for bariatric surgery can vary depending on your insurance policy and the guidelines set by your insurance company. Here are some key points in simpler terms:  - Insurance companies may cover bariatric surgery, but they have certain requirements that need to be met.  - These requirements usually include having a high body mass index (BMI) and trying other weight loss methods under medical supervision without success.  - You will need to go through evaluations to determine if you are a suitable candidate for surgery.  - Insurance companies may cover specific types of bariatric surgery, such as gastric bypass or gastric sleeve.  - Before getting the surgery, you will typically need approval from your insurance company. This involves submitting necessary documents and receiving their authorization.  To get accurate and up-to-date information about your insurance coverage for bariatric surgery, it's best to review your insurance policy or directly contact your insurance provider. They will provide you with the specific details about your coverage and any requirements you need to fulfill. |
| 4 | Will I need plastic surgery after bariatric surgery? | Sudden weight loss can result is loose or sagging skin, either temporary or permanent. The severity of it depends upon several things, including the percentage of weight loss, genetics, and lifestyle habits (i.e., exercise, smoking, etc.). It’s often recommended to allow some time to pass before undergoing plastic surgery.  Board-certified plastic surgeons in the Mass General Body Contouring Program perform procedures to remove excess skin and reshape the body to its desired proportions following weight loss surgery. |  | The need for plastic surgery following bariatric surgery varies from person to person. Bariatric surgery is primarily performed to help individuals lose a significant amount of weight and improve their overall health. However, substantial weight loss can lead to excess sagging skin in various areas of the body, including the abdomen, arms, thighs, breasts, and face.  Plastic surgery procedures, such as body contouring, are often considered by individuals who have undergone significant weight loss and desire to address the excess skin and tissue. These procedures can help reshape and tighten areas of the body affected by sagging skin.  Whether or not you will require plastic surgery after bariatric surgery depends on factors such as the amount of weight you lose, your age, your overall health, and your personal preferences. It's important to consult with a qualified plastic surgeon who can assess your specific situation and provide recommendations tailored to your needs.  Remember that plastic surgery is a personal choice, and not everyone who undergoes bariatric surgery chooses to have additional procedures. It's essential to have realistic expectations and understand the potential risks and benefits associated with plastic surgery before making any decisions. | 6/25/2023 | Certainly! After bariatric surgery, which helps you lose a lot of weight, some people may have loose or sagging skin in different parts of their body. Plastic surgery can be an option to remove or tighten this excess skin, giving your body a more contoured appearance.  Whether or not you'll need plastic surgery after bariatric surgery depends on how much weight you lose, your age, your overall health, and what you personally want. It's important to talk to a qualified plastic surgeon who can evaluate your situation and give you advice based on your specific needs.  Remember that plastic surgery is a personal choice, and not everyone who has bariatric surgery decides to have additional procedures. It's important to have realistic expectations and understand the possible risks and benefits of plastic surgery before making any decisions. |
| 4 | How effective is bariatric surgery? | Bariatric surgery is considered to be the most effective treatment for obesity in terms of sustained weight loss and improvement in other obesity-related conditions. The amount of weight you lose depends on the type of bariatric surgery and lifestyle habits following surgery. It is important to keep all scheduled follow-up visits so that your care team can monitor your weight loss.  The benefits of bariatric surgery include:  - An improved quality of life  - A reduced risk of other obesity-related diseases such as cardiovascular disease, sleep apnea, and type 2 diabetes  - Improved blood pressure  - A longer lifespan |  | Bariatric surgery, also known as weight loss surgery, is an effective treatment option for individuals with severe obesity. The primary goal of bariatric surgery is to promote weight loss and improve overall health. There are several types of bariatric procedures, including gastric bypass, gastric sleeve, and gastric banding.  The effectiveness of bariatric surgery can be evaluated based on various factors, including weight loss outcomes, improvement or resolution of obesity-related health conditions, and quality of life improvements. Here are some key points regarding the effectiveness of bariatric surgery:  1. Weight loss: Bariatric surgery can lead to significant and sustained weight loss. Most individuals can expect to lose a substantial amount of their excess weight within the first year after surgery. On average, gastric bypass and gastric sleeve procedures result in about 60-80% excess weight loss, while gastric banding is associated with a slightly lower weight loss range. However, individual results may vary.  2. Health improvements: Bariatric surgery has been shown to improve or resolve many obesity-related health conditions, such as type 2 diabetes, high blood pressure, sleep apnea, and joint pain. These improvements often occur even before significant weight loss takes place, suggesting that the surgery has metabolic effects beyond just weight reduction.  3. Long-term outcomes: Studies have demonstrated that bariatric surgery can lead to sustained weight loss over the long term, with many patients maintaining a significant portion of their initial weight loss even several years after the procedure. However, it's important to note that maintaining weight loss requires long-term lifestyle changes, including a healthy diet and regular physical activity.  4. Quality of life: Bariatric surgery can also have a positive impact on a person's quality of life. Many individuals experience improved mobility, increased self-esteem, reduced depressive symptoms, and a better overall sense of well-being following the surgery.  5. Risks and considerations: Like any surgical procedure, bariatric surgery carries risks, including the potential for complications such as infection, blood clots, or gastrointestinal issues. Additionally, individuals considering bariatric surgery should be aware of the necessary lifestyle changes, potential side effects, and the need for long-term follow-up care.  It's important to consult with a qualified healthcare professional who specializes in bariatric surgery to discuss the potential benefits and risks, and to determine if you are a suitable candidate for the procedure. | 6/25/2023 | Certainly! Bariatric surgery is a type of weight loss surgery for people who have severe obesity. It's a treatment option that aims to help them lose weight and improve their health.  Here's a simplified explanation of its effectiveness:  1. Weight loss: Bariatric surgery can help people lose a lot of their excess weight. On average, people can expect to lose a significant amount of weight in the first year after surgery.  2. Health improvements: Bariatric surgery can also improve or even cure health problems related to obesity, such as type 2 diabetes, high blood pressure, sleep apnea, and joint pain. These improvements can happen even before major weight loss occurs.  3. Long-term results: Studies show that people who have bariatric surgery can maintain their weight loss over the long term. However, it's important to make long-term lifestyle changes, like eating a healthy diet and being physically active, to keep the weight off.  4. Quality of life: Bariatric surgery can have a positive impact on a person's life. It can improve mobility, boost self-esteem, reduce feelings of depression, and overall make people feel better about themselves.  5. Risks and considerations: Like any surgery, there are risks involved with bariatric surgery, such as infections or problems with the digestive system. It's also important to know that lifestyle changes and ongoing follow-up care are necessary after the surgery.  It's best to talk to a healthcare professional who specializes in bariatric surgery to understand more about its benefits and risks and to see if it's the right option for you. |
| 4 | Is bariatric surgery safe? | While any surgical procedure has risks, bariatric surgery has been found to be one of the safest surgeries to undergo in comparison with other elective procedures. The Mass General Weight Center team performs a high volume of weight loss surgeries, which studies have shown relates to lower complication rates and improved outcomes related to quality and safety.  It is important to discuss any concerns you may have with your bariatric surgery care team. They will review and answer all your questions ahead of surgery. |  | Bariatric surgery, also known as weight loss surgery, can be considered safe for many individuals, but it does carry risks, as with any surgical procedure. The safety of bariatric surgery depends on various factors, including the individual's overall health, the specific type of surgery performed, the skill and experience of the surgeon, and the quality of post-operative care.  When performed by qualified surgeons in appropriate medical facilities, bariatric surgery can have significant benefits for people with severe obesity and related health conditions. It can help individuals achieve substantial weight loss and improve or resolve obesity-related conditions such as type 2 diabetes, high blood pressure, sleep apnea, and joint problems.  However, it's essential to recognize that bariatric surgery is not without risks. Potential complications can include infections, bleeding, blood clots, leaks in the gastrointestinal system, adverse reactions to anesthesia, and post-operative nutritional deficiencies. Additionally, like any surgical procedure, there is always a risk of adverse events related to anesthesia, wound healing, or other factors.  It's crucial for individuals considering bariatric surgery to undergo a thorough evaluation and consultation with a qualified healthcare professional specializing in bariatric surgery. They can assess the person's overall health, provide guidance on the risks and benefits, and help determine if the procedure is appropriate and safe for the individual.  Ultimately, the decision to undergo bariatric surgery should be made in consultation with a healthcare professional, considering individual circumstances, overall health, and the potential risks and benefits involved. | 6/25/2023 | Certainly! Bariatric surgery, which is a weight loss surgery, can be considered safe for many people, but it does have risks, like any other surgery. The safety of the surgery depends on different things, like a person's overall health, the specific type of surgery, the experience of the surgeon, and the care after the surgery.  When done by qualified surgeons in good medical facilities, bariatric surgery can have big benefits for people who are very obese and have health problems related to their weight. It can help them lose a lot of weight and improve or even cure conditions like diabetes, high blood pressure, sleep apnea, and joint problems.  However, it's important to know that bariatric surgery has risks. Some possible problems include infections, bleeding, blood clots, leaks in the stomach or intestines, reactions to the medicine used during surgery, and not getting enough nutrients after the surgery. Also, like with any surgery, there can be risks related to the anesthesia (medicine that makes you sleep) or healing wounds.  If you're thinking about having bariatric surgery, it's really important to talk to a qualified healthcare professional who knows a lot about these surgeries. They will check your health, explain the risks and benefits, and help you decide if the surgery is safe and right for you.  In simple terms, bariatric surgery can help people lose weight and improve their health, but it has risks, and it's important to talk to a doctor who knows about these surgeries to decide if it's safe for you. |
| 4 | When can I expect to return to daily life after weight loss surgery? | Patients generally are able to stand and walk a few hours after surgery and are discharged within one to two days. Avoiding high impact exercise and heavy weight lifting is recommended for at least one month, with an emphasis on listening to your body and resting as needed. Many patients return to work about four weeks after surgery.  Because of a lower calorie intake, many patients feel a lower energy than usual for a couple of weeks after surgery. Energy tends to elevate after transitioning from a liquid diet to solid foods. |  | The recovery time and the return to daily life after weight loss surgery can vary depending on the type of surgery performed and individual factors. Generally, it takes several weeks to months to fully recover and resume normal activities. Here are some general guidelines for the different types of weight loss surgery:  1. Gastric Bypass Surgery: It typically takes about 2 to 4 weeks to recover from gastric bypass surgery. During this time, you may experience pain, fatigue, and need to follow a strict diet plan. You should be able to resume most normal activities within a month but may need to avoid heavy lifting and strenuous exercise for a longer period.  2. Gastric Sleeve Surgery: Recovery from gastric sleeve surgery is usually quicker than gastric bypass surgery. You may need about 1 to 3 weeks to recover before returning to work and resuming light activities. Strenuous exercise and heavy lifting should be avoided for a few weeks.  3. Laparoscopic Adjustable Gastric Banding: Recovery time for laparoscopic adjustable gastric banding is generally shorter compared to other surgeries. You may be able to resume normal activities within 1 to 2 weeks. However, it's important to follow the specific guidelines provided by your surgeon.  It's crucial to note that these are general timelines, and individual experiences may vary. Your surgeon will provide you with detailed post-operative instructions, including dietary guidelines, activity restrictions, and follow-up appointments. It's essential to follow these instructions and have regular check-ups with your healthcare team to ensure a safe and successful recovery.  Remember that weight loss surgery is a significant procedure, and your body needs time to heal. It's important to prioritize your health, follow a proper recovery plan, and consult with your medical team regarding your specific circumstances. | 6/25/2023 | Certainly! After weight loss surgery, the time it takes to return to your regular daily life can vary. Here's a simplified explanation for each type of surgery:  1. Gastric Bypass Surgery: It usually takes about 2 to 4 weeks to recover. During this time, you may feel some pain and tiredness. You'll need to follow a strict diet. After a month, you can resume most normal activities, but you should avoid heavy lifting and strenuous exercise for a while.  2. Gastric Sleeve Surgery: Recovery from this surgery is faster. You may need about 1 to 3 weeks to recover before going back to work and doing light activities. Wait a few weeks before doing intense exercise or lifting heavy things.  3. Laparoscopic Adjustable Gastric Banding: Recovery time for this surgery is usually shorter. You can typically go back to normal activities within 1 to 2 weeks. Still, it's important to follow your surgeon's instructions.  Remember, these are just general estimates, and everyone's experience may be different. Your surgeon will give you specific instructions on what to do after the surgery, including what to eat and what activities to avoid. It's important to follow these instructions and have regular check-ups with your medical team to ensure a safe and successful recovery.  Just take your time to heal and prioritize your health. |
| 5 | What are the risks of the operation? | Weight loss surgery is major abdominal surgery with significant risks. However, we know experience counts and high-volume centers like X have an extremely low complications rate.  The following potential complications include, but are not limited to:  - Risk from general anesthesia  - Bleeding  - Infection  - Pneumonia  - Blood clot in the legs or lungs  - Hernias (internally or externally)  - Blockage or obstruction  - Narrowing where surgeons suture the pouch to the intestine, which can require another endoscopy and dilation  - Leakage from the stomach or the intestines  - Injury to liver and/or spleen  - Pulmonary and/or cardiac and/or renal failure  With the adjustable gastric band (lap-band), there's a risk that the band can either slip or erode. There's a risk that you will not lose weight if you don't follow the diet and exercise program. There's a risk of needing another operation or conversion to an open operation with a long incision. While there's a risk of death with each procedure: 1-in-200 with the gastric bypass, 1-in-500 with sleeve gastrectomy, and 1-in-10,000 with the lap band. | What are the risks of weight loss surgery? | Weight loss surgery, also known as bariatric surgery, can be an effective treatment option for individuals with severe obesity. However, like any surgical procedure, it carries certain risks and potential complications. Here are some of the risks associated with weight loss surgery:  1. Surgical risks: As with any surgery, there are general risks involved, such as adverse reactions to anesthesia, blood clots, infection, bleeding, and even death. However, advances in surgical techniques and pre-operative care have significantly reduced the occurrence of these risks.  2. Leakage and infection: After weight loss surgery, there is a risk of leakage or infection at the site where the stomach or intestines are stapled or divided. This can lead to complications such as peritonitis (inflammation of the abdominal cavity) or abscess formation.  3. Nutritional deficiencies: Weight loss surgeries, such as gastric bypass or sleeve gastrectomy, can affect nutrient absorption and lead to deficiencies in essential vitamins and minerals. Common deficiencies include iron, vitamin B12, calcium, vitamin D, and folate. Long-term vitamin and mineral supplementation is typically required to prevent these deficiencies.  4. Dumping syndrome: Dumping syndrome can occur after gastric bypass surgery. It happens when food moves too quickly from the stomach to the small intestine, causing symptoms like nausea, vomiting, diarrhea, abdominal cramps, and lightheadedness after eating certain foods, especially those high in sugar or fat.  5. Gallstones: Rapid weight loss after bariatric surgery can increase the risk of developing gallstones. The risk can be minimized by taking medications that help prevent gallstone formation or by removing the gallbladder during the weight loss surgery.  6. Bowel obstruction: In some cases, adhesions or scar tissue can develop after weight loss surgery, causing a blockage in the intestines. This can lead to symptoms such as abdominal pain, bloating, nausea, and vomiting, which may require further surgical intervention.  7. Psychological and emotional challenges: Weight loss surgery is a life-altering procedure, and individuals may experience psychological and emotional challenges following the surgery. Some individuals may struggle with body image issues, feelings of guilt or regret, or difficulty adjusting to changes in eating habits.  It's important to note that while these risks exist, weight loss surgery can also provide significant health benefits for individuals with obesity. The decision to undergo weight loss surgery should be made after a thorough evaluation of the risks and benefits, in consultation with healthcare professionals specializing in bariatric surgery. | 6/25/2023 | Certainly! Weight loss surgery, also called bariatric surgery, can help people with severe obesity lose weight. However, like any surgery, it has risks and possible problems. Here are some of the main risks:  1. Surgical risks: Any surgery can have general risks like bad reactions to anesthesia, blood clots, infections, bleeding, or even death. These risks are much lower now thanks to better techniques and care.  2. Leakage and infection: After the surgery, there is a chance of leaks or infections where the stomach or intestines were cut. This can lead to serious complications like inflammation or abscesses in the abdomen.  3. Nutritional deficiencies: Some weight loss surgeries can make it harder for the body to absorb important nutrients like vitamins and minerals. This can cause deficiencies in things like iron, vitamin B12, calcium, vitamin D, and folate. Taking supplements long-term can help prevent these deficiencies.  4. Dumping syndrome: After gastric bypass surgery, some people may experience "dumping syndrome." This happens when food moves too quickly from the stomach to the small intestine, causing symptoms like nausea, vomiting, diarrhea, abdominal cramps, and dizziness, especially after eating sugary or fatty foods.  5. Gallstones: Rapid weight loss after the surgery can increase the risk of developing gallstones. Medications can help prevent them, or sometimes the gallbladder is removed during the weight loss surgery.  6. Bowel obstruction: In some cases, scar tissue can form after the surgery and block the intestines. This can cause symptoms like abdominal pain, bloating, nausea, and vomiting, and may require further surgery.  7. Psychological and emotional challenges: Weight loss surgery is a big change, and people may face psychological and emotional challenges afterwards. Some may struggle with body image issues, feelings of guilt or regret, or difficulty adjusting to new eating habits.  It's important to know that while these risks exist, weight loss surgery can bring important health benefits for people with obesity. The decision to have the surgery should be made after carefully considering the risks and benefits, and with the guidance of healthcare professionals who specialize in bariatric surgery. |
| 5 | How successful are these operations? | The gastric bypass operation allows you to lose up to 82% of your excess body weight, however it's almost 100% effective in reversing obesity related co-morbidities including sleep apnea, hypertension and diabetes. The adjustable gastric banding (lap-band) operation allows you to lose 50% of your excess body weight at two years. It's also effective in reversing obesity related co-morbidities. The sleeve gastrectomy is between the gastric bypass and gastric banding in terms of weight loss with 60% excess weight loss at 1 year. | How successful are the various bariatric surgery operations? | Bariatric surgery, also known as weight loss surgery, is a medical procedure performed on individuals with obesity to help them lose weight and improve their overall health. There are several types of bariatric surgery, each with its own level of effectiveness and potential risks. Here's an overview of the commonly performed bariatric surgery procedures and their success rates:  1. Roux-en-Y Gastric Bypass (RYGB): This procedure involves creating a small stomach pouch and bypassing a portion of the small intestine. RYGB is considered one of the most effective weight loss surgeries. On average, patients can expect to lose 60-80% of their excess weight within the first year following surgery. Long-term studies have shown that RYGB can maintain significant weight loss over a period of 10 years or more.  2. Sleeve Gastrectomy: In this procedure, a large portion of the stomach is removed, leaving a banana-shaped sleeve. Sleeve gastrectomy has gained popularity and is known for its effectiveness in weight loss. It can result in an average excess weight loss of 50-70% within the first year. Long-term data is still emerging, but studies suggest that the weight loss can be maintained for at least five years.  3. Adjustable Gastric Banding: This surgery involves placing an inflatable band around the upper part of the stomach, creating a smaller pouch. The band can be adjusted to control the rate of food passage. While gastric banding was popular in the past, its effectiveness has been relatively modest compared to other procedures. The average weight loss varies widely, but it can be around 40-50% of excess weight within the first year. However, long-term results with gastric banding have been less consistent, with some patients experiencing weight regain.  4. Biliopancreatic Diversion with Duodenal Switch (BPD-DS): This is a complex procedure that involves removing a large portion of the stomach and rerouting the small intestine. BPD-DS is generally reserved for individuals with severe obesity. It has been shown to produce substantial weight loss, with an average excess weight loss of 70-80% within the first year. Long-term studies have demonstrated that the weight loss can be maintained for up to 10 years or more.  Success rates can vary depending on individual factors, such as initial weight, overall health, adherence to post-surgery guidelines, and lifestyle changes. It's important to note that bariatric surgery is not a magic solution and requires commitment to long-term lifestyle changes, including a healthy diet and regular exercise, to maintain weight loss and improve health outcomes. Consultation with a qualified healthcare professional is crucial to determine the most suitable option and understand the potential risks and benefits of each procedure. | 6/25/2023 | Certainly! Bariatric surgery is a type of surgery that helps people with obesity lose weight and improve their health. There are different types of surgeries, and each one has its own level of success.  1. Roux-en-Y Gastric Bypass (RYGB): This surgery makes the stomach smaller and bypasses part of the intestine. It's very effective, and people usually lose 60-80% of their extra weight within the first year. Studies have shown that the weight loss can be maintained for many years.  2. Sleeve Gastrectomy: This surgery removes a large part of the stomach, leaving a smaller "sleeve." It's also effective, and people can expect to lose around 50-70% of their extra weight within the first year. The long-term results look promising, with weight loss maintained for at least five years.  3. Adjustable Gastric Banding: This surgery involves placing an adjustable band around the upper part of the stomach to make it smaller. It's not as effective as the other surgeries, and people typically lose about 40-50% of their extra weight within the first year. However, maintaining weight loss in the long term can be challenging.  4. Biliopancreatic Diversion with Duodenal Switch (BPD-DS): This complex surgery removes a large part of the stomach and reroutes the small intestine. It's for people with severe obesity. It leads to significant weight loss, with around 70-80% of extra weight lost within the first year. Studies have shown that the weight loss can be maintained for more than 10 years.  It's important to remember that success rates can vary depending on individual factors. Bariatric surgery is not a quick fix and requires making lasting lifestyle changes like eating a healthy diet and exercising regularly. Talking to a healthcare professional is important to understand which surgery is right for you and to learn about the risks and benefits involved. |
| 5 | Why is there a 10% pre-operative weight loss required? | We require patients to begin a diet and exercise program that allows them to lose 10% of their weight pre-operatively for three reasons:  1) Patients who undergo weight loss before surgery have fewer complications during and after surgery.  2) This allows patients to truly understand the lifestyle changes that will occur after surgery and therefore make an informed consent about the diet and exercise programs that they will have to be on for the rest of their lives.  3) This allows us to identify the patients who are truly motivated and therefore who will be most successful following surgery. Most important, we'll work with you to develop a weight-loss program to achieve the 10% reduction goal. | Why is there a 10% pre-operative weight loss required for weight loss surgery? | The requirement of a 10% pre-operative weight loss before weight loss surgery is not a universal rule and may vary depending on the specific guidelines of the surgical center or the recommendations of the medical professionals involved in the patient's care. However, the purpose behind such a requirement can be attributed to several reasons:  1. Reducing surgical risks: Weight loss surgery is a major procedure that carries certain risks and complications. By losing a portion of excess weight before the surgery, patients can improve their overall health and reduce the potential risks associated with anesthesia, surgery, and post-operative recovery.  2. Shrinking the liver: The liver is an organ located in the upper abdomen, and it often becomes enlarged in individuals with obesity. Enlarged livers can obstruct the surgeon's access to the stomach during the operation. Pre-operative weight loss helps shrink the liver size, making the surgical procedure technically easier and safer.  3. Assessing patient commitment: Weight loss surgery is not a quick fix; it requires significant lifestyle changes and long-term commitment to ensure successful outcomes. Requiring a pre-operative weight loss demonstrates a patient's willingness to make necessary lifestyle modifications, follow post-operative instructions, and adopt healthier habits to maximize the benefits of the surgery.  4. Psychological preparation: Weight loss surgery brings about significant changes in a person's body and lifestyle. Requiring pre-operative weight loss allows patients to mentally prepare for the upcoming changes, become familiar with dietary modifications, and establish healthy routines that can aid in their post-operative recovery and long-term success.  It is important to note that the specific pre-operative weight loss requirements and guidelines can differ among surgical centers and healthcare providers. If you are considering weight loss surgery, it is recommended to consult with a qualified healthcare professional who can provide you with personalized advice and guidance based on your unique situation. | 6/25/2023 | Of course! Before weight loss surgery, some medical professionals may ask patients to lose about 10% of their excess weight. Here's why they might do that:  1. Safer surgery: Weight loss surgery is a big operation with risks. Losing some weight before the surgery can make the surgery safer and reduce the chances of complications during and after the procedure.  2. Easier access: The liver, which is an organ in the abdomen, can become bigger in people with obesity. A large liver can get in the way during surgery. Losing weight before the operation helps shrink the liver, making it easier for the surgeon to reach the stomach and perform the surgery.  3. Commitment and preparation: Weight loss surgery is not a quick fix. It requires long-term changes in lifestyle and habits. Requiring some weight loss before the surgery shows that a person is committed to making these changes and is mentally prepared for the surgery and the changes that come with it.  4. Getting ready mentally: Weight loss surgery brings big changes to a person's body and life. Losing some weight before the surgery allows the person to get mentally ready, practice new eating habits, and start making healthier choices. This can help them recover better after the surgery and have better long-term results.  It's important to remember that these requirements can vary among healthcare providers. If you're considering weight loss surgery, it's best to talk to a qualified healthcare professional who can give you personalized advice based on your situation. |
| 5 | What is the conversion rate? | Conversion to open surgery is required in less than 1% of patients. The risk of conversion is higher in patients who have had previous upper abdominal surgery. | What is the conversion rate from laparoscopic to open, with regards to weight loss surgery? | The conversion rate from laparoscopic to open surgery in weight loss surgery can vary depending on several factors, including the surgeon's expertise, the patient's individual circumstances, and the complexity of the procedure. Laparoscopic surgery, also known as minimally invasive surgery, is generally preferred in weight loss surgeries because it involves smaller incisions, reduces postoperative pain, promotes faster recovery, and has lower rates of complications compared to open surgery.  However, in some cases, laparoscopic surgery may need to be converted to an open procedure due to certain complications or technical challenges that arise during the operation. These complications can include difficulty visualizing or accessing the surgical site, excessive bleeding, or unexpected anatomical variations.  The conversion rate from laparoscopic to open surgery in weight loss procedures can vary widely depending on the specific procedure and the surgeon's experience. Generally, conversion rates range from 1% to 10% in bariatric surgeries such as gastric bypass or sleeve gastrectomy. However, it's important to note that these rates can vary significantly between different surgeons and institutions.  It's best to consult with a qualified bariatric surgeon to discuss the specific risks and potential conversion rates associated with your individual case, as they can provide more accurate information based on their experience and your medical history. | 6/25/2023 | Certainly! In weight loss surgery, laparoscopic surgery is the preferred method because it involves smaller incisions, less pain, and faster recovery. However, sometimes during the surgery, the surgeon may need to switch from laparoscopic to open surgery due to complications or difficulties they encounter.  The conversion rate refers to how often this switch from laparoscopic to open surgery happens. It can vary depending on factors like the surgeon's experience, the patient's condition, and the complexity of the procedure. On average, the conversion rate ranges from 1% to 10% in weight loss surgeries like gastric bypass or sleeve gastrectomy.  It's important to remember that these rates can differ between surgeons and hospitals. To get the most accurate information about your specific situation, it's best to consult with a qualified bariatric surgeon who can provide personalized advice based on their experience and your medical history. |
| 5 | How do I know which operation is for me? | Deciding which procedure is best for you is largely a personal one best achieved in discussion with your surgeon. | How do I know which type of weight loss surgery operation is for me? | Choosing the right weight loss surgery procedure is a significant decision that should be made in consultation with a qualified healthcare professional, such as a bariatric surgeon or a specialized weight loss team. They will consider various factors related to your health, medical history, lifestyle, and weight loss goals to determine the most suitable option for you. However, it's important to note that I can provide some general information about common weight loss surgery procedures to help you understand the options available.  1. Gastric Sleeve (Sleeve Gastrectomy): This procedure involves removing a portion of the stomach, creating a smaller, banana-shaped stomach pouch. It restricts the amount of food you can eat and reduces hunger by removing the part of the stomach responsible for producing the hunger hormone ghrelin.  2. Roux-en-Y Gastric Bypass (RGB): This surgery involves creating a small stomach pouch and then connecting it to the middle section of the small intestine, bypassing the remaining stomach and upper small intestine. It restricts food intake and reduces calorie absorption by altering the digestive process.  3. Adjustable Gastric Band (Lap-Band): This procedure involves placing an inflatable band around the upper part of the stomach, creating a smaller stomach pouch. The band is adjustable, allowing for varying levels of restriction. It reduces the amount of food you can consume and promotes a feeling of fullness.  4. Duodenal Switch: This surgery is a combination of a sleeve gastrectomy and intestinal bypass. It involves removing a large portion of the stomach and rerouting the small intestine to separate digestive juices from food, resulting in reduced calorie absorption.  The choice of surgery depends on several factors, including:  1. Body Mass Index (BMI): Your BMI helps determine your eligibility for certain procedures. Higher BMI individuals may benefit from surgeries with greater weight loss potential, such as gastric bypass or duodenal switch.  2. Overall Health: Your overall health and any existing medical conditions play a crucial role in determining the appropriate surgery. Some procedures are more suitable for individuals with certain health conditions.  3. Lifestyle and Eating Habits: Your lifestyle, eating habits, and ability to commit to dietary and lifestyle changes are important considerations. Some procedures require more strict dietary modifications and adherence to dietary guidelines.  4. Weight Loss Goals: Your weight loss goals and expectations should be discussed with your healthcare provider. Different procedures offer varying degrees of weight loss, and your surgeon can guide you regarding the expected outcomes.  5. Potential Risks and Benefits: Each procedure carries its own set of risks and benefits. Your surgeon will discuss these with you in detail to ensure you have a clear understanding.  It's important to have a comprehensive evaluation by a qualified bariatric surgeon who can assess your individual situation and guide you toward the most suitable weight loss surgery option. They will consider your unique circumstances and help you make an informed decision based on your health, goals, and preferences. | 6/25/2023 | Of course! Choosing the right weight loss surgery depends on factors like your health, weight loss goals, and lifestyle. Here are some basic explanations of common weight loss surgery options:  1. Gastric Sleeve: This surgery removes a large part of your stomach, making it smaller. It helps you eat less and reduces hunger.  2. Gastric Bypass: This surgery creates a small stomach pouch and connects it to a lower part of your small intestine. It limits food intake and reduces calorie absorption.  3. Adjustable Gastric Band: This surgery involves placing a band around the upper part of your stomach to create a smaller pouch. It restricts food intake and makes you feel full more quickly.  4. Duodenal Switch: This surgery combines a stomach reduction with bypassing part of the intestine. It reduces calorie absorption and provides significant weight loss.  When deciding on the right surgery, your doctor will consider your weight, health, lifestyle, and goals. They will discuss the different procedures with you, including their benefits and risks, to help you make an informed decision.  Remember, it's important to consult with a qualified healthcare professional who can assess your specific situation and provide personalized advice. |
| 5 | What will happen when I’m in the hospital? | In general, you'll be in the hospital for two nights. We'll expect you to get up and walk while you're in the hospital – this is your best defense against blood clots. You'll meet with the dietician before going home and while you're here, you'll have a clear liquid diet that will be limited to a medicine cup every 15 minutes.  Once home, you can drink until full. Please walk, drink once every hour, and take in at least 40 - 60 grams of protein daily for women, and 60 - 80 grams of protein daily for men | What will happen when I’m in the hospital after weight loss surgery? | When you are in the hospital after weight loss surgery, you can expect to go through several stages of care and recovery. Here's a general overview of what you might experience:  1. Postoperative Recovery: Immediately after the surgery, you will be taken to the recovery room, where medical staff will monitor your vital signs and ensure that you wake up safely from anesthesia. You may feel groggy or experience some pain or discomfort during this time.  2. Hospital Stay: Following the recovery period, you will be transferred to a hospital room. The length of your hospital stay will depend on the type of weight loss surgery you undergo and your individual progress. It can range from one to several days.  3. Pain Management: Weight loss surgery can cause discomfort and pain in the incision areas. Your healthcare team will provide you with appropriate pain medications to help manage your pain. They will also guide you on how to use them effectively and safely.  4. Fluid Intake: In the initial stages after surgery, you may be on a clear liquid diet, gradually transitioning to full liquids, pureed foods, and eventually solid foods. The healthcare team will closely monitor your fluid intake and provide guidelines to ensure proper hydration and nutrition.  5. Monitoring and Testing: During your hospital stay, you will undergo various tests and evaluations to monitor your progress. These may include blood tests, imaging studies, and assessments of your surgical site. These measures help identify any complications and ensure a smooth recovery.  6. Mobility and Physical Activity: As you recover, the medical staff will encourage you to start moving around and engage in light physical activity. This helps prevent complications like blood clots and aids in your overall recovery process.  7. Education and Support: The hospital staff will provide you with information on post-surgical care, dietary guidelines, lifestyle changes, and any necessary follow-up appointments. They may also connect you with a dietitian or support groups to help you adapt to your new lifestyle and provide ongoing support.  Remember, the specifics of your hospital experience may vary depending on the type of weight loss surgery you have and the protocols of the hospital where you undergo the procedure. It's essential to consult with your healthcare team to get personalized information and address any concerns you may have. | 6/25/2023 | Certainly! After weight loss surgery, when you're in the hospital, here's what you can expect in simpler terms:  1. Recovery Room: Right after the surgery, you'll be taken to a room where doctors and nurses will make sure you wake up safely from the anesthesia. You might feel a bit groggy and uncomfortable.  2. Hospital Room: Once you're stable, you'll be moved to a regular hospital room. How long you stay there depends on the type of surgery and how well you're doing. It could be a few days.  3. Pain Management: Surgery can cause pain, but the medical team will give you medicine to help you feel better. They'll explain how to take it and manage your pain effectively.  4. Eating and Drinking: In the beginning, you'll start with clear liquids like water and broth. Then you'll gradually move to other liquids, pureed foods, and finally solid foods. The doctors will guide you on what and how much to eat.  5. Tests and Monitoring: The hospital staff will regularly check your progress by doing tests, like blood tests and imaging. They'll also keep an eye on your surgical site to make sure everything is healing well.  6. Moving Around: It's important to start moving and doing light activities as you recover. This helps prevent problems like blood clots and helps you get better faster.  7. Learning and Support: The hospital team will teach you about taking care of yourself after surgery, including what to eat and how to make lifestyle changes. They might connect you with a dietitian or support groups to help you along the way.  Remember, the details can vary based on the type of surgery and the hospital's procedures. It's best to talk to your healthcare team for personalized information and to address any concerns you have. |
| 5 | Is there an age restriction for the surgery? | Surgery is done on patients between the ages of 18 - 65. If you're younger than 18, please refer to our Adolescent Bariatric Surgery Program at Lucile Packard Children's Hospital. If you are older than 65, cases are evaluated on a case by case basis. Please keep in mind your insurance may restrict your options. | Is there an age restriction for weight loss surgery? | The age restrictions for weight loss surgery, also known as bariatric surgery, can vary depending on the country, medical guidelines, and individual circumstances. In general, weight loss surgery is typically considered for individuals who are severely obese and have not been successful in losing weight through non-surgical methods.  Most guidelines suggest that weight loss surgery should be considered for individuals who:  1. Have a body mass index (BMI) of 40 or higher (extreme obesity).  2. Have a BMI between 35 and 39.9 (obesity) and are experiencing significant weight-related health problems such as diabetes, hypertension, or sleep apnea.  When it comes to age, the guidelines often recommend that individuals should be at least 18 years old before undergoing weight loss surgery. This is primarily because the body is still developing during adolescence, and it is generally advised to exhaust non-surgical weight loss options and allow for further growth and maturity before considering surgical interventions.  However, there may be exceptions to the age restriction in certain cases. For adolescents with severe obesity and significant medical issues, bariatric surgery may be considered after thorough evaluation and discussion with a multidisciplinary team of healthcare professionals. These decisions are typically made on an individual basis, taking into account the person's overall health, emotional well-being, and ability to understand the risks and commitments associated with the surgery.  It's important to consult with a qualified healthcare professional or a bariatric surgeon who can evaluate your specific situation and provide you with accurate information based on the latest medical guidelines and practices. | 6/25/2023 | Certainly! Weight loss surgery, also called bariatric surgery, is a procedure for people who are very overweight and have tried other ways to lose weight without success. There is usually an age restriction for this type of surgery.  In general, weight loss surgery is recommended for people who:  1. Are extremely obese, with a body mass index (BMI) of 40 or higher.  2. Have a BMI between 35 and 39.9 and are experiencing serious health problems related to their weight, like diabetes or high blood pressure.  When it comes to age, most guidelines suggest that individuals should be at least 18 years old before considering weight loss surgery. This is because the body is still developing during the teenage years, and it's important to try other weight loss methods first and let the body grow and mature.  However, there may be exceptions for teenagers with severe obesity and significant health issues. In those cases, bariatric surgery could be an option after careful evaluation and discussion with a team of healthcare professionals. The decision is made on a case-by-case basis, considering the person's overall health, emotional well-being, and understanding of the risks and commitments involved in the surgery.  It's important to talk to a qualified healthcare professional or a bariatric surgeon who can assess your specific situation and provide you with accurate information based on the latest medical guidelines. |
| 5 | Is there a weight limit for this operation? | We look at Body Mass Index (BMI), which is the weight over the body surface area squared. Patients who have a BMI of 35-40 and have obesity related co-morbidities are candidates for this surgery. Patients who have a BMI greater than 40, even if they don't have co-morbidities, are also candidates for this operation. | Is there a weight limit for weight loss surgery? | Yes, there is typically a weight limit for weight loss surgery, also known as bariatric surgery. The weight limit is determined by various factors and may vary depending on the specific type of weight loss surgery being considered.  The most common types of weight loss surgery include gastric bypass, sleeve gastrectomy, and adjustable gastric banding. Each procedure has its own criteria and weight limits, which are generally based on the individual's body mass index (BMI). BMI is a calculation based on a person's height and weight and is used to determine if someone is underweight, normal weight, overweight, or obese.  The specific weight limits for weight loss surgery can vary between medical institutions and countries, and they may also consider other factors such as the individual's overall health, medical history, and any previous attempts at weight loss. Generally, weight loss surgery is recommended for individuals with a BMI of 40 or higher (severe obesity) or a BMI of 35 or higher (obesity) with obesity-related health conditions such as diabetes, high blood pressure, or sleep apnea.  It's important to note that weight loss surgery is not a one-size-fits-all solution, and each person's situation is unique. If you're considering weight loss surgery, it's best to consult with a qualified healthcare professional who can evaluate your specific circumstances and provide appropriate recommendations. | 6/25/2023 | Certainly! Weight loss surgery, also known as bariatric surgery, is a procedure that helps people who are severely overweight or obese lose weight. However, not everyone who is overweight is a candidate for this surgery.  To determine if someone is eligible for weight loss surgery, doctors use a calculation called body mass index (BMI). BMI takes into account a person's height and weight to estimate how much body fat they have. Generally, weight loss surgery is recommended for people with a BMI of 40 or higher (severe obesity) or a BMI of 35 or higher (obesity) if they also have obesity-related health problems like diabetes or high blood pressure.  These BMI thresholds serve as guidelines to ensure that weight loss surgery is performed on individuals who are at a higher risk due to their weight. However, it's important to remember that each person's situation is unique, and decisions about weight loss surgery should be made in consultation with a healthcare professional who can evaluate your specific needs and provide appropriate recommendations. |
| 5 | What are obesity related comorbidities? | These include diabetes, hypertension, arthritis, degenerative joint disease, sleep apnea, low pack pain, gastroesophageal reflux disease, stress incontinence, high cholesterol and triglyceride levels and depression. |  | Obesity is a complex medical condition that can have a significant impact on overall health. It is associated with various comorbidities, which are additional medical conditions that often coexist with obesity. Some of the most common obesity-related comorbidities include:  1. Type 2 Diabetes: Obesity is a major risk factor for developing type 2 diabetes. Excess body fat can lead to insulin resistance, making it harder for the body to regulate blood sugar levels.  2. Cardiovascular Diseases: Obesity increases the risk of developing heart disease, high blood pressure (hypertension), and other cardiovascular conditions. Excess weight can contribute to the accumulation of plaque in the arteries, leading to atherosclerosis and an increased risk of heart attacks and strokes.  3. Sleep Apnea: Obesity is a significant risk factor for sleep apnea, a sleep disorder characterized by pauses in breathing or shallow breathing during sleep. Excess fat around the neck can obstruct the airway, leading to breathing difficulties during sleep.  4. Joint Problems: The excessive weight that accompanies obesity can put additional stress on the joints, leading to conditions such as osteoarthritis. Weight loss often helps alleviate joint pain and improve mobility.  5. Respiratory Issues: Obesity can lead to reduced lung function and increased risk of respiratory problems such as asthma, chronic obstructive pulmonary disease (COPD), and pulmonary hypertension.  6. Fatty Liver Disease: Obesity increases the likelihood of developing non-alcoholic fatty liver disease (NAFLD), a condition characterized by the accumulation of fat in the liver. In some cases, NAFLD can progress to more severe conditions like liver cirrhosis.  7. Certain Cancers: Obesity has been linked to an increased risk of various types of cancer, including breast, colon, endometrial, kidney, and pancreatic cancer.  8. Mental Health Disorders: Obesity is associated with an increased risk of mental health issues such as depression, anxiety, and low self-esteem.  9. Reproductive Disorders: Obesity can contribute to hormonal imbalances and fertility problems in both men and women. In women, obesity is linked to conditions like polycystic ovary syndrome (PCOS) and menstrual irregularities.  10. Gallbladder Disease: Obesity is a risk factor for gallstones and gallbladder disease. The excess weight can alter the balance of cholesterol and bile salts in the gallbladder, leading to the formation of gallstones.  It's important to note that not everyone who is obese will develop these comorbidities, and individuals may experience different combinations and severities of conditions. However, managing obesity through lifestyle changes, including a healthy diet, regular exercise, and weight loss, can help reduce the risk and severity of these comorbidities. If you have concerns about obesity or related health issues, it's advisable to consult with a healthcare professional for personalized advice and guidance. | 6/25/2023 | Certainly! Obesity-related comorbidities are additional medical conditions that often occur alongside obesity. These conditions are more likely to develop in individuals who are overweight or obese. Here are some examples of these conditions in simpler terms:  1. Type 2 Diabetes: When a person is obese, they have a higher chance of getting a disease called type 2 diabetes, which affects how the body controls blood sugar levels.  2. Heart Problems: Being obese increases the risk of heart diseases like high blood pressure, blocked arteries, and a higher chance of having heart attacks or strokes.  3. Sleep Problems: Obesity can cause a sleep disorder called sleep apnea, where a person's breathing gets disrupted during sleep, often due to extra fat around the neck blocking the airway.  4. Joint Pain: The extra weight on the body can put stress on the joints, leading to conditions like arthritis, which causes pain and stiffness in the joints.  5. Breathing Issues: Obesity can make it harder to breathe properly and increase the likelihood of conditions such as asthma or chronic obstructive pulmonary disease (COPD).  6. Fatty Liver: When a person is obese, fat can build up in the liver and lead to a condition called non-alcoholic fatty liver disease (NAFLD), which can cause liver damage.  7. Certain Cancers: Obesity increases the risk of developing different types of cancer, such as breast, colon, endometrial (uterine), kidney, and pancreatic cancer.  8. Mental Health Problems: Being obese can have a negative impact on mental health, leading to conditions like depression, anxiety, and low self-esteem.  9. Reproductive Issues: Obesity can cause hormonal imbalances and fertility problems in both men and women. In women, it can lead to conditions like polycystic ovary syndrome (PCOS) and irregular periods.  10. Gallbladder Disease: Obesity raises the chances of developing gallstones and problems with the gallbladder, which stores a substance called bile that helps with digestion.  Remember, not everyone who is obese will experience all of these conditions, and the severity may vary. However, taking steps to manage obesity through healthy eating, regular exercise, and weight loss can help reduce the risk and severity of these additional health problems. It's always a good idea to speak with a healthcare professional for personalized advice and support. |
| 5 | What are the pre-operative requirements in order to undergo either operation? | You must have a stable relationship with a primary care physician who has been treating you continuously for one year prior to consideration for surgery.  You must have a dietician evaluation.  You must have a psychological evaluation. Patients with bipolar disease are generally excluded from consideration for surgery.  If you have a history of heart disease, if you've taken Fen-Phen in the past, if you're over the age of 50, or other cardiac risk factors, you'll require an echocardiogram.  At some point prior to surgery, you'll have to undergo a chest x-ray, an EKG and blood tests. | What are the pre-operative requirements in order to undergo weight loss surgery? | The specific pre-operative requirements for weight loss surgery, also known as bariatric surgery, can vary depending on the type of procedure and the guidelines of the healthcare provider or surgical center. However, here are some common pre-operative requirements that are typically considered:  1. Body Mass Index (BMI): Generally, individuals who are candidates for weight loss surgery have a BMI of 40 or higher, or a BMI of 35-39.9 with significant obesity-related health conditions. BMI is a calculation based on a person's height and weight.  2. Medical Evaluation: A comprehensive medical evaluation is usually conducted to assess the overall health of the individual and to determine if they are suitable for surgery. This may involve reviewing medical history, performing physical examinations, and conducting tests or screenings.  3. Psychological Evaluation: Weight loss surgery can have significant psychological and emotional implications. Therefore, a psychological evaluation is often conducted to assess the individual's mental health, emotional stability, and their understanding of the procedure and its potential outcomes.  4. Nutritional Assessment: It is important to evaluate the individual's nutritional status prior to surgery. This may involve meeting with a registered dietitian or nutritionist to assess dietary habits, identify nutritional deficiencies, and provide education on post-surgical dietary requirements.  5. Weight Loss Attempts: Many healthcare providers require individuals to have attempted previous non-surgical methods of weight loss, such as lifestyle modifications, dieting, and exercise, before considering weight loss surgery. This requirement demonstrates a commitment to long-term lifestyle changes.  6. Education and Counseling: Individuals are often required to participate in educational programs and counseling sessions to gain a better understanding of the risks, benefits, and expectations associated with weight loss surgery. This helps ensure that individuals are well-informed and prepared for the procedure.  7. Smoking and Alcohol Cessation: Smoking and excessive alcohol consumption can increase the risks associated with weight loss surgery. Therefore, individuals may be required to quit smoking and limit alcohol intake for a specified period before surgery.  8. Documentation: Pre-operative requirements may include providing documentation such as medical records, test results, and insurance approvals. This helps ensure that the individual meets the necessary criteria for surgery and facilitates the authorization process.  It's important to note that these requirements can vary among healthcare providers and surgical centers. If you are considering weight loss surgery, it's best to consult with a healthcare professional or a bariatric surgeon who can provide you with specific information based on your individual circumstances. | 6/25/2023 | Certainly! Before undergoing weight loss surgery, there are several things that doctors and healthcare providers typically check or ask for. Here are the main requirements in simpler terms:  1. Body Mass Index (BMI): They check your BMI, which is a number calculated from your height and weight. If your BMI is very high, usually 40 or higher, or if it's between 35 and 39.9 with obesity-related health problems, you may be considered a candidate for weight loss surgery.  2. Medical Evaluation: The doctor will review your medical history, give you a physical check-up, and may ask you to do some tests to make sure you're healthy enough for surgery.  3. Psychological Evaluation: They want to know how you're doing emotionally and mentally because weight loss surgery can have an impact on your emotions and mental health. They will assess your mental well-being and make sure you understand what the surgery involves and what the results might be.  4. Nutritional Assessment: They will check your eating habits and nutrition to see if there are any deficiencies or problems that need to be addressed before surgery. You may meet with a nutritionist who can help you with a proper diet plan.  5. Weight Loss Attempts: Doctors often want to see that you have tried other methods of losing weight, like changing your lifestyle, dieting, and exercising, before considering surgery. This shows that you are committed to making long-term changes.  6. Education and Counseling: You may be asked to attend classes or counseling sessions to learn more about the surgery, its benefits, and risks. This helps make sure you have the right information and understand what to expect.  7. Smoking and Alcohol: Smoking and drinking too much alcohol can make the surgery riskier, so you may be asked to quit smoking and reduce alcohol intake for a certain period before the surgery.  8. Documentation: You will need to provide important documents like medical records, test results, and insurance approvals to show that you meet the requirements for the surgery.  It's important to remember that these requirements can vary depending on the healthcare provider or surgeon you choose. If you're considering weight loss surgery, it's best to talk to a doctor or a surgeon who can explain the specific requirements based on your situation. |
| 5 | What does laparoscopic surgery mean? | Laparoscopic surgery means that instead of making one large incision in your abdomen, we make five small incisions in the middle and to the left side of your abdomen. These smaller incisions allow you to recover faster. You'll also experience less pain, leave the hospital sooner and will be back to your normal activity sooner. In general, we start all of our cases laparoscopically unless the patient has had previous surgery on their stomach. |  | Laparoscopic surgery, also known as minimally invasive surgery or keyhole surgery, is a surgical technique that allows surgeons to perform procedures through small incisions in the body using specialized instruments and a laparoscope.  The laparoscope is a long, thin tube with a high-resolution camera and light source attached to its tip. It is inserted through a small incision, typically in the abdomen, allowing the surgeon to visualize the internal organs on a monitor.  During the procedure, additional small incisions may be made to insert other surgical instruments needed to perform the surgery. These instruments are specifically designed for laparoscopic surgery and allow the surgeon to manipulate, cut, and suture tissues as required.  The benefits of laparoscopic surgery include:  1. Reduced trauma: The small incisions used in laparoscopic surgery cause less trauma to the body compared to traditional open surgery, which involves larger incisions.  2. Less scarring: The small incisions result in smaller scars, which are generally less noticeable and may heal more quickly.  3. Faster recovery: Laparoscopic surgery often leads to shorter hospital stays and quicker recovery times compared to open surgery.  4. Reduced pain and discomfort: The smaller incisions and less tissue manipulation can result in less postoperative pain and discomfort.  5. Lower risk of complications: Laparoscopic surgery may have a lower risk of certain complications, such as infection and bleeding, due to the smaller incisions and reduced tissue exposure.  Laparoscopic surgery is used for various procedures, including gallbladder removal, hernia repair, appendectomy, ovarian cyst removal, hysterectomy, and gastrointestinal surgeries. However, not all surgeries can be performed laparoscopically, and the feasibility depends on the specific condition and the surgeon's expertise. | 6/25/2023 | Certainly! Laparoscopic surgery is a type of surgery where doctors use small cuts in the body instead of large ones. They insert a special tool called a laparoscope through one of the small cuts. The laparoscope has a tiny camera that lets the doctors see inside the body on a screen. They can also use other small tools to perform the surgery through these small cuts.  The advantages of laparoscopic surgery are:  1. Less damage to the body: The small cuts used in this surgery cause less harm to the body compared to big cuts used in traditional surgery.  2. Smaller scars: Because the cuts are small, the scars left behind are smaller and less noticeable.  3. Faster recovery: People who have laparoscopic surgery often spend less time in the hospital and recover more quickly compared to those who have traditional surgery.  4. Less pain and discomfort: The small cuts and gentle handling of tissues during laparoscopic surgery usually result in less pain and discomfort after the procedure.  5. Lower risk of complications: Laparoscopic surgery has a lower chance of certain problems, such as infections and bleeding, because the cuts are smaller and less tissue is exposed.  Laparoscopic surgery is used for different procedures, like removing the gallbladder, repairing hernias, removing appendix, treating ovarian cysts, performing hysterectomies, and surgeries related to the digestive system. However, not all surgeries can be done this way, and it depends on the specific condition and the surgeon's expertise. |
| ASMBS | When can I start exercising after surgery? | Right away! To start, you will take easy, short walks while you are in the hospital. The key is to start slow. Listen to your body and your surgeon. If you lift weights or do sports, stay “low impact” for the first month. | When can I start exercising after weight loss surgery? | After weight loss surgery, the exact timing for starting exercise can vary depending on the type of surgery you underwent and your individual circumstances. It is crucial to follow the specific guidelines provided by your healthcare team, as they are familiar with your unique situation. Generally, your surgeon or healthcare provider will provide instructions on when you can begin exercising and what types of activities are appropriate for you.  In the early stages following weight loss surgery, your focus will primarily be on recovery and allowing your body to heal. Typically, you will start with light physical activity, such as short walks, as soon as you are able to do so comfortably. This aids in promoting circulation, preventing blood clots, and maintaining muscle tone.  As time progresses and you heal, your healthcare team will gradually increase your activity level and may suggest incorporating more rigorous exercises. The timeline for this progression varies, but it is often around 4 to 6 weeks after surgery. However, it is essential to consult with your surgeon or healthcare provider to ensure you are ready for increased physical activity.  Remember, the guidelines may differ based on the type of weight loss surgery you had. Procedures like gastric bypass, gastric sleeve, and gastric banding can have different recovery periods and exercise recommendations. Your surgeon will provide specific advice based on the surgery you underwent.  Always listen to your body and be cautious when starting or intensifying an exercise routine. It's important to start slowly and gradually increase the duration and intensity of your workouts. Regular communication with your healthcare team is vital throughout the process, as they can provide personalized guidance and make adjustments based on your progress and any potential complications.  Please consult with your healthcare provider for specific advice regarding your post-surgery exercise plan. | 6/25/2023 | Certainly! After weight loss surgery, the time to start exercising varies depending on the type of surgery and your personal situation. It's important to follow the instructions given by your healthcare team. In the beginning, you'll focus on recovering and allowing your body to heal. You may start with light activities like short walks to promote circulation and maintain muscle tone.  As you heal, your healthcare team will gradually increase your activity level and may suggest more intense exercises. This usually happens around 4 to 6 weeks after surgery, but it depends on your specific circumstances.  The guidelines can be different for different types of weight loss surgery, so it's crucial to consult with your surgeon or healthcare provider for specific advice.  Start slowly and listen to your body. Increase the duration and intensity of your workouts gradually. Stay in touch with your healthcare team to get personalized guidance and make any necessary adjustments based on your progress.  Remember, always consult with your healthcare provider for specific advice regarding your post-surgery exercise plan. |
| ASMBS | Will I have to exercise after the procedure? | For many patients, exercise is important for stress control and appetite control, as well as burning off calories.  As we age, lack of activity can lead to being frail or fragile, which is quite dangerous to overall health. Healthy bones and avoiding muscle loss partly depends on doing weekly exercise.  Most patients also think of exercise as something that must be intense and painful (like “boot camp”). Regular, modest activity is far more useful in the long term. Even elite athletes can’t stay at a “peak” every week of the year. Instead, work with your surgeon’s program to find a variety of activities that can work for you. There is no “one-size-fits-all” plan. Expect to learn and change as you go! | Will I have to exercise after bariatric surgery? | Yes, exercise is generally recommended after bariatric surgery as part of a comprehensive approach to weight loss and overall health. While bariatric surgery can help you achieve significant weight loss, it is important to incorporate regular physical activity to optimize your results and maintain a healthy lifestyle.  Exercise offers numerous benefits, such as improving cardiovascular health, increasing muscle strength and endurance, boosting metabolism, enhancing mood and mental well-being, and preventing muscle loss during weight loss. It also helps with weight maintenance and can aid in toning and shaping your body as you lose weight.  However, it's important to note that the specific exercise recommendations may vary depending on various factors such as your overall health, the type of bariatric surgery you undergo, and any specific guidelines provided by your healthcare team. It is crucial to consult with your healthcare provider, including your surgeon and a qualified exercise professional, to determine the most appropriate exercise program for you.  They can provide personalized recommendations based on your individual needs and abilities, gradually increasing the intensity and duration of your exercise routine as you recover from surgery. Remember to listen to your body, start slowly, and gradually progress to more challenging exercises over time.  Additionally, incorporating a balanced and healthy diet along with regular exercise will help you achieve optimal results and maintain your weight loss in the long term. | 6/25/2023 | Certainly! After bariatric surgery, it's generally a good idea to include exercise as part of your weight loss and overall health plan. Exercise has many benefits, such as improving your heart health, making your muscles stronger, boosting your metabolism, and helping you feel better mentally. It can also help you maintain your weight loss and shape your body as you lose weight.  However, the specific exercise recommendations may vary depending on factors like your overall health, the type of surgery you had, and any guidelines from your healthcare team. It's important to talk to your healthcare provider, including your surgeon and an exercise professional, to figure out the best exercise plan for you.  They can give you personalized advice based on your needs and abilities. They'll likely suggest starting slowly and gradually increasing the intensity and duration of your exercises as you recover from surgery. It's important to listen to your body and not push yourself too hard.  Remember, combining exercise with a healthy diet will give you the best results and help you maintain your weight loss in the long run. |
| ASMBS | Will I have to go on a diet before I have surgery? | Yes. Many insurance companies require patients to be on a weight loss program before qualifying for surgery.  Many bariatric surgeons put their patients on a special pre-operative diet, usually 2 or 3 weeks just before surgery. The reason for the pre-operative diet is to shrink the liver and reduce fat in the abdomen. This helps during the procedure and makes it safer.  Some insurance companies require a physician-monitored diet three to six months prior to surgery as part of their coverage requirement. These diets are very different from the short-term diets and usually are more about food education and showing a willingness to complete appointments and to learn. | Will I have to go on a diet before I have bariatric surgery? | The requirements for undergoing bariatric surgery can vary depending on several factors, including the specific procedure you're considering, your overall health, and the recommendations of your healthcare team. In many cases, however, a pre-operative diet is often a standard part of the preparation for bariatric surgery.  The purpose of a pre-operative diet is typically to reduce the size of your liver, which makes the surgery safer and easier for the surgeon to perform. The liver is often enlarged in individuals who are overweight or obese, and by following a low-calorie diet for a period of time before the surgery, you can help shrink the liver and decrease the risk of complications during the procedure.  The specifics of the pre-operative diet will depend on your healthcare provider's recommendations. It may involve consuming a liquid diet or a very low-calorie diet for a certain period leading up to the surgery. It's important to follow your healthcare team's instructions closely to ensure the best possible outcome from your surgery.  It's worth noting that bariatric surgery is a major procedure that requires careful consideration and planning. It's essential to consult with a qualified healthcare professional who specializes in bariatric surgery to assess your individual situation, provide personalized recommendations, and guide you through the entire process. | 6/25/2023 | Certainly! Before undergoing bariatric surgery, it is common for doctors to recommend a pre-operative diet. The purpose of this diet is to help make the surgery safer and more successful.  When you are overweight or obese, your liver tends to be larger in size. By following a low-calorie diet for a period of time before the surgery, you can help reduce the size of your liver. This makes the surgery easier for the surgeon and reduces the risk of complications.  The pre-operative diet may involve consuming mostly liquids or very low-calorie foods for a certain period before the surgery. The specific details of the diet will depend on your healthcare provider's instructions.  It's important to follow the diet instructions given by your healthcare team closely, as it will help prepare your body for the surgery and improve the chances of a positive outcome.  Remember, bariatric surgery is a major procedure, so it's crucial to consult with a qualified healthcare professional who specializes in this area. They will provide personalized guidance based on your specific needs and circumstances. |
| ASMBS | Will I have to be on a diet after the surgery? | No. Most people think of a “diet” as a plan that leaves you hungry. That is not the way people feel after surgery.  Eventually, most patients get some form of appetite back 6-18 months after surgery. Your appetite is much weaker, and easier to satisfy than before.  This does not mean you can whatever and whenever you want. Healthier food choices are important for best results, but most patients still enjoy tasty food, and even occasional “treats”. | Will I have to be on a diet after bariatric or weight loss surgery? | Yes, after undergoing bariatric or weight loss surgery, it is generally necessary to follow a specific diet plan to support your weight loss goals and ensure a healthy recovery. The exact diet recommendations may vary depending on the type of surgery you undergo, as well as individual factors and medical considerations. It is essential to follow the guidelines provided by your healthcare team, including your surgeon, nutritionist, or dietitian, as they will provide tailored advice based on your specific needs.  In the initial stages following surgery, you will typically be on a liquid or pureed diet, gradually transitioning to soft foods and then solid foods over time. This progressive diet plan allows your body to adjust to the changes and ensures proper healing of the surgical site. Portion control and eating small, frequent meals are common aspects of the post-surgery diet.  Long-term dietary guidelines after bariatric or weight loss surgery typically involve consuming a balanced, nutrient-dense diet with an emphasis on lean proteins, vegetables, fruits, and whole grains. You may be advised to limit or avoid certain foods that are high in sugar, fat, and calories, as well as beverages that are high in sugar or carbonation. Adequate hydration and taking recommended vitamin and mineral supplements are also crucial to meet your nutritional needs.  Remember that after bariatric or weight loss surgery, the diet plays a significant role in your overall success, weight management, and health. It is important to work closely with your healthcare team and follow their guidance to optimize your results and maintain long-term success. | 6/25/2023 | Certainly! After bariatric or weight loss surgery, it's important to follow a specific diet plan to help you lose weight and recover properly. The exact plan will depend on the type of surgery you have and your personal needs. Here's a simplified explanation:  1. Right after surgery: You'll start with liquids or pureed foods and gradually move to softer foods. This helps your body adjust and heal.  2. Long-term diet: You'll need to eat a balanced diet with foods that have lots of nutrients. Focus on lean proteins (like chicken or fish), vegetables, fruits, and whole grains.  3. Avoid certain foods: You may be asked to limit or avoid foods that are high in sugar, fat, and calories. You'll also want to stay away from sugary drinks and carbonated beverages.  4. Portion control: Eating smaller, more frequent meals is important. You'll learn how to control your portion sizes to avoid overeating.  5. Stay hydrated: Drinking enough water is crucial. It's also common to take vitamin and mineral supplements to make sure you get all the nutrients you need.  It's essential to work closely with your healthcare team, including your surgeon and nutritionist, to follow their advice and have the best results. They will provide you with more detailed guidance and support along the way. |
| ASMBS | Is weight loss surgery a ‘cop-out’? To lose weight, should people with obesity just go on a diet and exercise? | Many people who suffer from obesity find it hard to lose weight and keep the weight off with diet and exercise alone. This is likely due to foods available and genes.  The National Institutes of Health (NIH) Expert Panel stated that, without surgery, long-term weight loss is nearly impossible for those affected by severe obesity. Studies show little long-term success with diet and exercise alone.(3) Weight loss surgeries are effective in maintaining long-term weight loss, in part, because these procedures change the body’s natural responses to dieting that make weight loss so difficult. When a person goes on a diet, their body produces more hormones that cause an increase in hunger and a decrease in calories burned. This decrease in calories the body burns is more than explained by the decrease in body size. Therefore, there are significant differences between someone who has lost weight by diet and someone of the same size who has never lost weight. For example, the body of the person who reduces their weight from 200 to 170 pounds burns fewer calories than the body of someone weighing 170 pounds who has never been on a diet. This means that in order to maintain weight loss, the person who has been on a diet will have to eat fewer calories than someone who naturally weighs the same. Weight loss procedures, unlike diet, also cause biological changes that help reduce food intake. Energy (in the form of food) intake is decreased with surgery by restricting stomach size and limiting absorption. In addition, weight loss surgery changes the production of certain gut hormones (or signals) that communicate with the brain to reduce hunger, decrease appetite, and enhance the feeling of being full. In these ways, weight loss surgery, unlike dieting, produces long-term weight loss. |  | Weight loss surgery is not a cop-out. It is a valid and sometimes necessary option for individuals with obesity who have tried various methods of weight loss without success. While diet and exercise are important components of a healthy lifestyle, they may not always be sufficient for significant and sustainable weight loss for everyone.  Obesity is a complex condition influenced by various factors, including genetics, metabolism, environment, and behavior. For some individuals, these factors make it extremely challenging to lose weight through traditional methods alone. In such cases, weight loss surgery, also known as bariatric surgery, can be an effective tool to help people achieve significant weight loss and improve their overall health.  Weight loss surgery works by altering the anatomy of the digestive system, which can lead to reduced hunger, increased satiety, and changes in nutrient absorption. It is typically recommended for individuals with severe obesity (usually a body mass index [BMI] of 40 or higher) or those with a BMI of 35 or higher with obesity-related health complications.  It's important to note that weight loss surgery is not a standalone solution. It is typically accompanied by ongoing lifestyle changes, including a healthy diet and regular exercise. These modifications are crucial for long-term success and to maintain the weight loss achieved through surgery.  Ultimately, the decision to undergo weight loss surgery is a personal one that should be made in consultation with healthcare professionals who can assess an individual's specific circumstances and help them determine the most appropriate approach for weight management. | 6/25/2023 | Certainly! Weight loss surgery is not a cop-out. It is a helpful option for people with obesity who have tried diets and exercise but haven't been able to lose enough weight. Obesity is a complicated condition, and for some people, it's really hard to lose weight through traditional methods alone.  Weight loss surgery changes how the digestive system works, which can make people feel less hungry and fuller faster. This helps them eat less and lose weight. It's usually recommended for people with severe obesity or those who have obesity-related health problems.  However, weight loss surgery is not a quick fix. People who undergo surgery also need to make long-term changes to their lifestyle, like eating healthy and being active, to maintain their weight loss.  In short, weight loss surgery is a tool that can help people with obesity lose weight when other methods haven't worked. It's a decision that should be made with the guidance of healthcare professionals who can assess each person's situation and recommend the best approach. |
| ASMBS | When can I get pregnant after weight loss surgery? | Most groups advise waiting 12-18 months after surgery before getting pregnant.  Most women are much more fertile after surgery, even with moderate weight loss.  Birth control pills do NOT work as well in patients who are overweight. Birth control pills are not very reliable during the time your weight is changing. For this reason, having an IUD or using condoms and spermicide with ALL intercourse is needed. Menstrual periods can be very irregular, and you can get pregnant when you least expect it! |  | Weight loss surgery, also known as bariatric surgery, can have an impact on fertility and pregnancy. The timing of when you can get pregnant after weight loss surgery depends on various factors, including the type of surgery you had, your overall health, and your weight loss progress.  Generally, it is recommended to wait for a period of time after weight loss surgery before attempting pregnancy. This waiting period allows your body to heal and adjust to the changes caused by the surgery. It also gives you time to achieve stable weight loss and improve your nutritional status.  The specific waiting period can vary depending on the type of weight loss surgery:  1. Gastric Bypass: Many healthcare providers recommend waiting at least 12 to 18 months before attempting pregnancy after gastric bypass surgery. This waiting period allows for significant weight loss and stabilization of your body's nutrient absorption.  2. Adjustable Gastric Banding: With adjustable gastric banding (such as LAP-BAND), some providers recommend waiting for at least 6 to 12 months before getting pregnant. This period allows for weight loss and adjustment to the band.  3. Sleeve Gastrectomy: The waiting period after sleeve gastrectomy is usually shorter compared to gastric bypass surgery. Some healthcare providers recommend waiting for around 6 to 12 months before attempting pregnancy.  It's important to consult with your healthcare provider who specializes in bariatric surgery and reproductive health. They can provide personalized guidance based on your specific circumstances and monitor your progress to ensure it's safe for you to conceive.  During this waiting period, it's crucial to use effective contraception to prevent unintended pregnancies if you're sexually active and not yet ready for pregnancy.  Remember, each person's situation is unique, and individual factors may influence the recommended waiting period. Your healthcare provider will be able to provide the most accurate advice based on your medical history and progress after weight loss surgery. | 6/25/2023 | Certainly! After weight loss surgery, it's generally recommended to wait for a period of time before trying to get pregnant. This waiting period allows your body to heal and adjust to the changes caused by the surgery, and it also gives you time to achieve stable weight loss and improve your nutrition.  The waiting time depends on the type of weight loss surgery you had:  1. Gastric Bypass: You may need to wait 12 to 18 months before attempting pregnancy after gastric bypass surgery. This allows for significant weight loss and helps your body adjust to absorbing nutrients properly.  2. Adjustable Gastric Banding: If you had a gastric banding surgery like LAP-BAND, you might need to wait 6 to 12 months before getting pregnant. This allows time for weight loss and adjustment to the band.  3. Sleeve Gastrectomy: After a sleeve gastrectomy, the waiting period is usually shorter, around 6 to 12 months, before trying to conceive.  It's important to talk to your doctor who specializes in weight loss surgery and reproductive health. They can give you personalized advice based on your situation and monitor your progress to ensure it's safe for you to get pregnant.  During the waiting period, it's crucial to use contraception if you're sexually active and not ready for pregnancy.  Remember, everyone is different, and your doctor will provide you with the most accurate advice based on your medical history and progress after weight loss surgery. |
| ASMBS | Will the baby be healthy? | Yes.  After surgery, there is much less risk of experiencing problems during pregnancy and during childbirth. There are also fewer miscarriages and stillbirths than in women with obesity who have not had surgery and weight loss.  Kids born after mom’s surgery are LESS at risk of being affected by obesity later, due to activation of certain genes during fetal growth. There is also less risk of needing a C section. | Will the baby be healthy (posted in the same chat as the previous question) | The health of a baby is influenced by various factors, including the health of the parents, prenatal care, genetics, and overall lifestyle choices. After weight loss surgery, it's important to work closely with your healthcare provider to optimize your health before attempting pregnancy. This can help improve the chances of having a healthy pregnancy and baby.  Weight loss surgery can have positive effects on fertility and pregnancy outcomes for individuals who were previously obese or had obesity-related health conditions. Losing weight and improving overall health can reduce the risk of complications during pregnancy, such as gestational diabetes, high blood pressure, and pre-eclampsia.  However, it's essential to note that pregnancy after weight loss surgery may still carry some risks and considerations:  1. Nutritional Status: Weight loss surgery can affect nutrient absorption and may lead to nutritional deficiencies. It's important to work with a healthcare provider or a registered dietitian to ensure you're meeting your nutritional needs before and during pregnancy. Adequate intake of essential nutrients, such as folic acid, iron, calcium, and protein, is crucial for a healthy pregnancy and fetal development.  2. Weight Management: Pregnancy after weight loss surgery requires careful weight management. While it's essential to gain weight during pregnancy, it should be done in a controlled manner. Your healthcare provider will guide you on appropriate weight gain targets based on your pre-pregnancy weight and overall health.  3. Monitoring and Support: Regular prenatal care and close monitoring by your healthcare provider are vital during pregnancy after weight loss surgery. They can monitor your nutritional status, screen for any complications, and provide appropriate guidance and support throughout the pregnancy journey.  It's important to discuss your plans for pregnancy with a healthcare provider who specializes in both bariatric surgery and reproductive health. They will be able to provide personalized advice based on your specific circumstances and help ensure the best possible outcomes for you and your baby. | 6/25/2023 | Certainly! After weight loss surgery, the health of your baby can be influenced by various factors. Here's a simplified explanation:  1. Improved Health: Weight loss surgery can improve your overall health and reduce the risk of certain complications during pregnancy. Losing weight and becoming healthier can lower the chances of problems like diabetes and high blood pressure.  2. Nutritional Needs: Weight loss surgery can affect how your body absorbs nutrients. It's important to work with a healthcare provider or dietitian to make sure you're getting the right amount of essential nutrients. This is crucial for a healthy pregnancy and the development of your baby.  3. Managing Weight: During pregnancy, it's normal to gain some weight, but it should be done in a controlled way. Your healthcare provider will guide you on how much weight you should aim to gain based on your weight before pregnancy and overall health.  4. Regular Check-ups: It's important to have regular check-ups with your healthcare provider throughout your pregnancy. They will monitor your health, nutrition, and screen for any potential complications. They can also offer support and guidance during this special time.  It's a good idea to talk to a healthcare provider who specializes in both weight loss surgery and pregnancy. They can provide personalized advice and help you have a healthy pregnancy and a healthy baby. |
| ASMBS | How long after weight loss surgery will I have to be from work? | After surgery, most patients return to work in one or two weeks. |  | The duration of time you may need to take off from work after weight loss surgery can vary depending on several factors, including the type of surgery you undergo, your individual recovery progress, and the nature of your job. It's important to note that I am an AI language model and not a medical professional, so it's crucial to consult with your surgeon or healthcare provider for personalized advice. However, I can provide you with some general information.  In many cases, individuals who undergo weight loss surgery can expect to take around two to four weeks off from work to allow for initial recovery and healing. This timeframe can vary, and some individuals may need additional time off if their job involves physically demanding tasks or if they experience complications during recovery.  The first few days after surgery typically involve hospitalization, and you may need to stay in the hospital for a few days to a week, depending on the procedure. Once you are discharged, you will need some time at home to recover further.  Weight loss surgery is a significant procedure that requires a period of adjustment, as your body adapts to the changes. During the initial weeks after surgery, you may experience fatigue, discomfort, dietary restrictions, and follow-up appointments with your healthcare team. It's crucial to allow yourself enough time to rest, follow your surgeon's instructions, and focus on your recovery.  Ultimately, the decision about when to return to work should be made in consultation with your healthcare provider, who can evaluate your progress, review any job-related factors, and provide personalized recommendations based on your individual circumstances. | 6/25/2023 | Certainly! After weight loss surgery, the amount of time you'll need to take off from work can vary. Typically, people take about two to four weeks off to recover, but this can be different for each person. Factors like the type of surgery you have and the nature of your job can affect how much time you'll need.  Right after the surgery, you'll stay in the hospital for a few days to a week. After being discharged, you'll need some more time at home to recover. During this time, you might feel tired, uncomfortable, and have certain dietary restrictions. You'll also have follow-up appointments with your healthcare team.  Returning to work depends on how well you're healing and the physical demands of your job. It's important to listen to your healthcare provider's advice and give yourself enough time to rest and recover before going back to work. It's best to talk to your healthcare provider to get personalized advice based on your situation. |
| ASMBS | Will I lose my hair after surgery? | Some hair loss is common between 3 and 6 months following surgery but almost always temporary.  The reasons for hair loss are not totally understood. Even if you take all recommended supplements and meet protein requirements, hair loss will be noticed until the follicles come back. Adequate intake of protein, vitamins, and minerals will help to ensure hair re-growth, and avoid longer term thinning. | Will I lose my hair after bariatric surgery? | Bariatric surgery, also known as weight loss surgery, can potentially result in hair loss. This is a common occurrence after any major surgery, not just bariatric procedures. The medical term for this condition is telogen effluvium.  Telogen effluvium is a type of temporary hair loss that typically occurs a few months after surgery. It happens because the body undergoes significant stress and experiences nutritional changes during the recovery period. This can disrupt the hair growth cycle, causing a larger number of hairs to enter the resting phase (telogen) and eventually fall out.  Hair loss after bariatric surgery is usually temporary and tends to resolve on its own within six to twelve months as the body adjusts to the changes. It is important to note that not everyone who undergoes bariatric surgery will experience hair loss, and the severity of hair loss can vary from person to person.  To minimize the risk of hair loss and support hair health during the recovery phase after bariatric surgery, it is advisable to follow a balanced and nutritious diet, including adequate protein intake. You may also benefit from taking vitamin and mineral supplements, as recommended by your healthcare provider. It's essential to discuss any concerns about hair loss with your surgeon or a qualified healthcare professional who can provide personalized guidance based on your specific situation. | 6/25/2023 | Certainly! After bariatric surgery, it's possible to experience hair loss, but it's usually temporary. The medical term for this type of hair loss is telogen effluvium. It happens because the body goes through a lot of stress and changes after surgery, which can affect the normal hair growth cycle.  Normally, our hair goes through different phases: a growing phase, a resting phase, and a falling out phase. After surgery, more hairs can enter the resting phase and eventually fall out, causing hair loss. However, this hair loss is temporary and typically resolves on its own within six to twelve months as your body adjusts.  Not everyone who has bariatric surgery will experience hair loss, and the severity can vary from person to person. To support hair health during your recovery, it's important to eat a balanced diet with enough protein. Your doctor may also recommend taking vitamin and mineral supplements. If you're concerned about hair loss, it's best to talk to your surgeon or a healthcare professional who can give you personalized advice based on your situation. |
| ASMBS | Will I need to have plastic surgery after weight loss? | Some patients may choose to have plastic surgery, but this depends on many factors. | Will I need to have plastic surgery after weight loss from bariatric surgery? | Whether or not you will need plastic surgery after weight loss from bariatric surgery depends on various factors, including the amount of weight you lose, your age, genetics, skin elasticity, and your personal aesthetic goals.  Bariatric surgery, such as gastric bypass or gastric sleeve, can lead to significant weight loss. As you lose a substantial amount of weight, your skin and tissues may not fully retract or tighten, resulting in excess sagging skin. This is particularly common in areas such as the abdomen, arms, breasts, thighs, and face.  Plastic surgery procedures, such as body contouring, abdominoplasty (tummy tuck), brachioplasty (arm lift), breast lift, thigh lift, or facelift, can be options to address excess skin and improve body shape and contour after weight loss. These procedures can help remove the excess skin, tighten the underlying tissues, and provide a more aesthetically pleasing appearance.  However, it's important to note that not everyone who undergoes bariatric surgery will require or choose to have plastic surgery afterward. Some individuals may find that their skin gradually adjusts and retracts over time, while others may be comfortable with the excess skin or prefer non-surgical methods, such as exercise and strength training, to improve their body shape.  If you are considering bariatric surgery, it's advisable to discuss potential concerns about excess skin and the need for plastic surgery with your bariatric surgeon or a qualified plastic surgeon. They can assess your individual situation, provide guidance, and discuss the available options based on your specific needs and goals. | 6/25/2023 | Of course! After bariatric surgery, which helps you lose a lot of weight, your skin may not bounce back completely. This can lead to loose or sagging skin in different parts of your body like the stomach, arms, breasts, thighs, or face.  If you're bothered by the excess skin and want a tighter, more defined appearance, you may consider plastic surgery. Procedures like body contouring, tummy tuck, arm lift, breast lift, thigh lift, or facelift can remove the extra skin and give you a better shape.  However, not everyone who has bariatric surgery needs or chooses to have plastic surgery. Some people find that their skin adjusts over time, while others may be fine with the excess skin or prefer exercise to improve their body shape.  If you're thinking about bariatric surgery, it's a good idea to talk to your bariatric surgeon or a plastic surgeon. They can help you understand if excess skin might be a concern for you and discuss the options available based on your specific needs and goals. |
| ASMBS | Do most people who have weight loss surgery regain their weight? | No. A small number of patients can regain their weight but the vast majority lose significant weight and keep this weight off.  More than 95% of patients will successfully lose half of their extra body weight or more after surgery for weight loss.  (1) Roux en Y gastric bypass patients, for example, on average lose approximately 70% of their extra body weight initially, and 2 years or more after surgery will regain approximately 5% of their extra body weight.  (2) Compared to these results, patients who attempt weight loss without surgery, on average, are able to lose little weight and may gain weight in the long-term. |  | Weight regain after weight loss surgery can occur in some individuals, but it is not accurate to say that "most" people who have weight loss surgery regain their weight. The success rates and long-term outcomes of weight loss surgery can vary depending on various factors, including the type of surgery, adherence to dietary and lifestyle changes, and individual differences.  Weight loss surgery, such as gastric bypass or gastric sleeve surgery, can be effective in helping individuals achieve significant weight loss and improve their overall health. Studies have shown that many people who undergo weight loss surgery are able to maintain substantial weight loss in the long term.  However, it is important to recognize that weight loss surgery is not a magic solution and requires ongoing commitment to lifestyle changes. Some individuals may experience weight regain if they do not follow the recommended dietary guidelines, engage in regular physical activity, or address any underlying psychological or emotional factors that may contribute to overeating or unhealthy eating behaviors.  It's crucial for individuals who undergo weight loss surgery to work closely with healthcare professionals, including dietitians, psychologists, and support groups, to establish healthy habits, address any challenges, and maintain long-term success. With appropriate lifestyle changes and ongoing support, many people can achieve and sustain significant weight loss after surgery. | 6/25/2023 | Certainly! Weight loss surgery can be effective for helping people lose weight and improve their health. However, it's not a guarantee that everyone will keep the weight off forever. Some people may regain weight after surgery if they don't make necessary changes to their diet and lifestyle.  To increase the chances of long-term success, it's important for individuals to follow the advice of their healthcare team, which may include nutritionists, psychologists, and support groups. They can help establish healthy habits, address any emotional or psychological factors related to overeating, and provide ongoing support.  In summary, weight loss surgery can be helpful, but it's not a magical solution. It requires commitment to making healthy choices and getting support from professionals to maintain weight loss over time. |
| ASMBS | Can I go off some of my medications after weight loss surgery? | Many patients are able to stop using some medications. |  | I'm not a doctor, but I can provide some general information that may be helpful. Weight loss surgery, such as gastric bypass or gastric sleeve surgery, can have a significant impact on your overall health, including the management of certain medical conditions. However, the decision to go off medications should always be made in consultation with your healthcare provider or a specialist who is familiar with your specific medical history and conditions.  Weight loss surgery can lead to significant weight loss, which may result in improvements or resolution of certain medical conditions that are related to obesity. These may include type 2 diabetes, high blood pressure, high cholesterol, and sleep apnea, among others. As you lose weight and these conditions improve, it's possible that your healthcare provider may recommend reducing or discontinuing some medications.  However, it's important to note that not all medications can be stopped immediately after weight loss surgery. Some medications may still be necessary to manage certain conditions, even if they improve. Your healthcare provider will assess your individual situation, consider your overall health, and make recommendations based on their expertise and your specific needs.  It's crucial to maintain regular follow-up appointments with your healthcare provider after weight loss surgery to monitor your progress, adjust medications as needed, and ensure your overall well-being. They will guide you through the process of medication management as you continue on your weight loss journey.  Remember, the information provided here is not a substitute for professional medical advice, diagnosis, or treatment. Always consult with a qualified healthcare professional for personalized guidance regarding your medications and health. | 6/25/2023 | Certainly! Weight loss surgery can have a positive impact on your health and may lead to improvements in certain medical conditions related to obesity, such as diabetes, high blood pressure, and high cholesterol. As you lose weight, your doctor may consider reducing or stopping some medications you are taking for these conditions. However, it's important to talk to your doctor before making any changes to your medications, as they will assess your specific situation and make recommendations based on your health needs. It's essential to keep regular follow-up appointments with your doctor to monitor your progress and make sure you are staying healthy throughout your weight loss journey. Always consult with your doctor for personalized advice about your medications and health. |
| ASMBS | Will I have to take vitamins after surgery? | You will need to take a multivitamin for life.  You may need higher doses of certain vitamins or minerals, especially Iron, Calcium, and Vitamin D. You will also need to have at least yearly lab checks. Insurance almost never pays for vitamin and mineral supplements but usually does pay for labs. You can pay for supplements out of a flex medical account. | Will I have to take vitamins after bariatric surgery? | After bariatric surgery, it is generally recommended to take vitamin and mineral supplements to ensure adequate nutrition. Bariatric surgery alters the digestive system, which can affect the absorption of essential nutrients from food. The specific recommendations may vary depending on the type of bariatric surgery you undergo and your individual needs. However, here are some common vitamin and mineral supplements that are often recommended after bariatric surgery:  1. Multivitamin: A high-potency multivitamin is typically recommended to provide a broad range of vitamins and minerals.  2. Calcium: Bariatric surgery can affect the body's ability to absorb calcium, leading to a risk of deficiency. Calcium supplements, often combined with vitamin D, are commonly recommended.  3. Vitamin B12: Bariatric surgery can impair the absorption of vitamin B12 from food. Supplementation with sublingual tablets, injections, or nasal sprays may be necessary.  4. Iron: Iron deficiency is a common concern after bariatric surgery. Iron supplements may be needed to prevent or treat anemia.  5. Vitamin D: Many people, including those who haven't undergone bariatric surgery, are deficient in vitamin D. However, after bariatric surgery, the risk of deficiency may be higher. Vitamin D supplementation is often recommended.  It is important to consult with your healthcare provider or a registered dietitian who specializes in bariatric nutrition to determine the specific vitamin and mineral supplementation plan that is best suited for you. They can evaluate your individual needs and guide you on the appropriate dosages and frequency of supplementation based on your surgery type, nutrient levels, and overall health. | 6/25/2023 | Certainly! After bariatric surgery, your body may have difficulty absorbing all the necessary nutrients from the food you eat. To ensure you get the right amount of vitamins and minerals, doctors often recommend taking supplements. Here are the main ones:  1. Multivitamin: This is a pill that contains many vitamins and minerals to make sure you're getting a wide range of nutrients.  2. Calcium: Your body might struggle to absorb calcium after surgery, so taking a calcium supplement (often with vitamin D) can help prevent any deficiencies.  3. Vitamin B12: Surgery can make it harder for your body to absorb vitamin B12 from food. Taking B12 supplements, which can come in different forms like tablets, injections, or nasal sprays, can help you get enough.  4. Iron: Iron deficiency is common after bariatric surgery and can cause anemia. Taking iron supplements can help prevent or treat this.  5. Vitamin D: Many people, even those who haven't had surgery, lack vitamin D. After bariatric surgery, the risk of deficiency may be higher, so taking vitamin D supplements is often recommended.  It's important to talk to your doctor or a dietitian who specializes in bariatric nutrition. They can give you specific advice on which supplements to take, how much to take, and how often to take them based on your surgery type, nutrient levels, and overall health. |
| ASMBS | Do weight loss surgery patients have serious health problems caused by vitamin deficiency? | As long as patients take their appropriate vitamins, it is rare to have health problems from vitamin deficiency.  Weight loss surgery can lead to reduced amounts of vitamins and minerals because people eat less and may absorb less in the intestines. Weight loss surgeries have different effects on vitamins and minerals based on how much change in absorption they cause. Deficiencies in vitamins, minerals, and protein can negatively affect health causing:  - fatigue (feeling tired)  - anemia (low levels of red blood cells that carry oxygen through your body)  - bone and muscle loss  - impaired night vision  - low immunity (your body’s ability to stay healthy)  - loss of nerve function (can affect your senses such as touch, taste, and smell)  - mental function deficits (changes how clearly you think)  Fortunately, nutrient deficiencies following surgery can be easily avoided with a good diet and the use of supplements including vitamins, minerals, and sometimes protein supplements. Before and after surgery, patients are advised of the appropriate diet and vitamin supplement needed. Most weight loss surgery programs also require patients to have their vitamins and minerals checked on a regular basis after surgery. Nutrient deficiencies and any associated health problems are preventable with monitoring and patients following diet and supplement (vitamin and mineral) recommendations. Health problems due to deficiencies usually occur in patients who do not regularly follow-up to monitor healthy vitamin and mineral levels. Be sure you stay in touch with your weight loss surgery team for best results! |  | Weight loss surgery, also known as bariatric surgery, can lead to significant weight loss in individuals struggling with obesity. While it can have many positive effects on a person's health, including improvements in conditions like diabetes and hypertension, it is true that weight loss surgery patients are at risk of developing certain vitamin deficiencies.  The most common types of weight loss surgeries are gastric bypass, sleeve gastrectomy, and gastric banding. These procedures can affect the absorption of essential nutrients, including vitamins and minerals, leading to potential deficiencies over time.  Some of the vitamins that weight loss surgery patients may be at risk of deficiency in include:  1. Vitamin B12: Gastric bypass surgery, in particular, can reduce the absorption of vitamin B12. Deficiency in this vitamin can lead to anemia, fatigue, neurological problems, and impaired cognitive function.  2. Vitamin D: Reduced stomach size and changes in the intestine can impair the absorption of vitamin D. Vitamin D deficiency can result in weakened bones, muscle weakness, and an increased risk of fractures.  3. Calcium: Calcium absorption may also be compromised after weight loss surgery, especially with procedures like gastric bypass. Inadequate calcium intake can lead to weakened bones and an increased risk of osteoporosis.  4. Iron: Reduced stomach size and changes in the intestine can impair iron absorption. Iron deficiency can cause anemia, fatigue, and reduced exercise tolerance.  5. Folate: Gastric bypass surgery can affect the absorption of folate, a B vitamin important for the production of red blood cells and DNA synthesis. Folate deficiency can lead to anemia and impaired cell function.  To mitigate the risk of these deficiencies, weight loss surgery patients typically require lifelong vitamin and mineral supplementation. They are usually prescribed a combination of supplements, including a multivitamin, calcium with vitamin D, and potentially additional individual vitamins or minerals based on their specific needs.  It's important for weight loss surgery patients to closely follow their healthcare provider's guidelines, undergo regular monitoring of their nutrient levels, and have ongoing nutritional counseling to prevent and address any potential deficiencies that may arise. | 6/25/2023 | Certainly! Weight loss surgery can sometimes lead to a lack of certain vitamins and minerals in the body. This happens because the surgery changes how the stomach and intestines work, making it harder for the body to absorb these essential nutrients.  The main vitamins that weight loss surgery patients may have a deficiency in are vitamin B12, vitamin D, calcium, iron, and folate. These deficiencies can cause problems such as anemia (low red blood cell count), fatigue, weakened bones, and difficulty with cognitive function.  To prevent these deficiencies, patients need to take vitamin and mineral supplements for the rest of their lives. These supplements usually include a multivitamin, calcium with vitamin D, and sometimes additional individual vitamins or minerals based on their specific needs.  It's important for weight loss surgery patients to follow their doctor's instructions, have regular check-ups to monitor their nutrient levels, and receive ongoing guidance on their diet and nutrition to avoid any potential problems caused by these deficiencies. |
| ASMBS | Is obesity caused by food addiction, similar to alcoholism or drug dependency? | “Food addiction” as a cause of obesity is extremely rare.  Although some people with obesity have eating disorders, such as binge eating disorder syndrome, most people have obesity caused by many factors. When treating addiction, such as alcohol and drugs, one of the first steps is to stop using drugs or alcohol. This does not work with obesity as we need to eat to live. Also, there may be other issues causing a person’s weight gain. Weight gain generally occurs when the amount of food eaten is greater than the number of calories burned. There are other conditions, however, that affect weight gain that do not involve too much eating or a less active lifestyle including:  - Poor sleeping habits  - Eating foods that may increase body fat (sugar, high fructose corn syrup, trans fat, processed meats and processed grains)  - Low intake of fat-fighting foods (fruits, vegetables, legumes, nuts, seeds, quality protein)  - Stress and mental distress  - Many types of medications  - Pollutants  Obesity also leads to more obesity, which is one of the reasons why the disease is considered progressive. Weight gain causes a number of signals (hormones) in the body that increase the risk for even greater weight gain and obesity. To make matters worse, obesity affects certain body functions that control appetite and hunger in a manner that can cause an increase in the amount of food eaten at any given meal and the desire to eat more often. There are many causes for obesity and the disease of obesity is far more than just an ‘addiction’ toward food. The treatment of obesity only as an addiction may help for a very small percentage of individuals whose only underlying cause for obesity is excessive and addictive eating, but would be unlikely to benefit most people, particularly those individuals affected by severe obesity. |  | The causes of obesity are complex and multifactorial, and it is influenced by a combination of genetic, environmental, behavioral, and psychological factors. While there is evidence to suggest that some people may have a tendency to overeat or develop an unhealthy relationship with food, it is important to note that obesity is not solely caused by food addiction in the same way that alcoholism or drug dependency are linked to substance addiction.  Food addiction is a controversial concept in the scientific community, and there is ongoing debate about its definition and classification. While certain individuals may experience symptoms that resemble addiction-like behaviors with regards to food, such as loss of control, cravings, and continued use despite negative consequences, it is not yet clear whether food addiction meets the same criteria as substance addiction.  There are similarities between addiction and certain aspects of overeating or excessive food consumption. Both may involve the brain's reward and pleasure centers, with certain foods triggering a release of dopamine, a neurotransmitter associated with pleasure and reward. However, it is important to recognize that the relationship between food and the brain is complex, and factors such as emotional well-being, stress, cultural influences, and social environment also play significant roles.  It is worth noting that obesity can result from a combination of factors, including genetic predisposition, poor dietary choices, sedentary lifestyle, psychological factors, socioeconomic factors, and environmental influences. While food addiction may contribute to overeating and weight gain in some individuals, it is only one potential factor among many.  Managing obesity typically requires a comprehensive approach that includes a balanced diet, regular physical activity, behavioral changes, and, in some cases, medical intervention. If you have concerns about your weight or eating habits, it is always recommended to consult with a healthcare professional who can provide personalized advice and guidance. | 6/25/2023 | Certainly! Obesity is caused by a combination of factors, including genetics, environment, behavior, and psychology. While some people may have a tendency to overeat or develop an unhealthy relationship with food, it's important to note that obesity is not solely caused by food addiction in the same way that alcoholism or drug dependency are caused by addiction to substances.  Food addiction is a concept that is still being debated among scientists. Some individuals may show addiction-like behaviors with food, such as feeling out of control, having cravings, or continuing to eat despite negative consequences. However, it's not yet clear if food addiction meets the same criteria as substance addiction.  While there are similarities between addiction and overeating, such as the brain's reward system being involved, the relationship between food and the brain is complex. Factors like emotions, stress, culture, and social environment also play important roles.  Obesity is usually caused by a combination of factors, including genetics, unhealthy eating habits, lack of physical activity, psychological factors, and the world around us. Food addiction may contribute to overeating and weight gain in some people, but it's just one of many potential factors.  Managing obesity usually requires a comprehensive approach, including a healthy diet, regular exercise, behavior changes, and sometimes medical help. If you're concerned about your weight or eating habits, it's best to consult with a healthcare professional who can give you personalized advice. |
| ASMBS | Does weight loss surgery increase the risk for alcohol abuse? | There is a small group of patients after weight loss surgery who have a problem with alcohol abuse. Most of those people, but not all, already had experiences with alcohol abuse before surgery.  Recommended precautions after weight loss surgery:  - Avoid alcoholic beverages during the rapid weight loss period, and then drink only rarely  - Be aware that even small amounts of alcohol can cause intoxication (‘being drunk’)  - Avoid driving or operating heavy equipment after drinking any alcohol  - Remember that you may feel less intoxicated than you are, and will not be a reliable judge of whether it is safe for you to drive after drinking.  - Seek help if drinking becomes a problem  If you feel drinking alcohol may be an issue for you after surgery, please contact your primary care doctor or surgeon and discuss this further. They will be able to help you identify resources available to address any alcohol-related issues.  It has been found in scientific research that a small percentage of weight loss surgery patients report having problems with alcohol after surgery (7-10%). (6) Many who abuse alcohol after surgery had problems with alcohol abuse at some time prior to surgery, but occasionally alcohol problems can arise in patients who never had such issues before their surgery. Alcohol sensitivity is increased after weight loss surgery so that the effects of alcohol are felt with fewer drinks than before surgery. Studies also find that with certain weight loss procedures (such as the gastric bypass or sleeve gastrectomy), drinking an alcoholic beverage increases blood alcohol to levels that are considerably higher than before surgery or in comparison to the alcohol levels of individuals who have not had a weight loss procedure. In addition, studies have found that alcohol takes much longer to clear out of a person’s system if they have had weight loss surgery, which can pose safety and legal hazards for driving. Furthermore, it has been found that some patients may feel as though they are sober (‘not drunk’) even when they are over the legal blood alcohol limit for driving. For all of these reasons, weight loss surgery patients are advised to take precautions regarding alcohol. |  | Weight loss surgery, also known as bariatric surgery, is a medical procedure that helps individuals with severe obesity lose weight by reducing the size of the stomach or bypassing a portion of the digestive tract. While weight loss surgery has been shown to be an effective treatment for obesity and related health conditions, it is important to note that it can have various impacts on individuals, including potential changes in behavior and psychological well-being.  Some studies have suggested a potential link between weight loss surgery and an increased risk of alcohol abuse or alcohol-related problems. However, it's important to recognize that these findings are not universal, and the relationship between weight loss surgery and alcohol abuse is complex and multifactorial.  Several factors may contribute to the increased risk of alcohol abuse after weight loss surgery:  1. Physiological changes: Weight loss surgery can result in physiological changes that affect alcohol metabolism. After surgery, alcohol is absorbed more rapidly into the bloodstream, leading to increased blood alcohol levels and intensified effects. This can make individuals more susceptible to alcohol-related problems.  2. Psychosocial factors: Weight loss surgery can have a significant impact on a person's emotional and psychological well-being. Some individuals may turn to alcohol as a coping mechanism for stress, depression, anxiety, or body image issues. In these cases, the underlying psychological factors may contribute to alcohol abuse rather than the surgery itself.  3. Pre-existing risk factors: It's important to consider that individuals who undergo weight loss surgery often have a history of obesity, which itself can be associated with a higher risk of alcohol abuse. Factors such as genetics, family history, social environment, and personal vulnerabilities can contribute to both obesity and alcohol-related problems.  4. Substance transfer addiction: Some individuals who undergo weight loss surgery may substitute their food addiction with other addictive behaviors, such as alcohol or substance abuse. This transfer addiction can occur due to the underlying psychological issues driving addictive behavior.  While the research suggests a potential association between weight loss surgery and alcohol abuse, it's essential to remember that not everyone who undergoes weight loss surgery will develop alcohol-related problems. The majority of individuals successfully manage their weight and make positive lifestyle changes without developing alcohol abuse issues.  To minimize the risks, healthcare professionals closely monitor patients after weight loss surgery and provide comprehensive pre- and post-operative counseling and support. This includes addressing psychological concerns, offering nutritional guidance, and encouraging healthy coping mechanisms.  If you are considering weight loss surgery or have already undergone the procedure, it is crucial to discuss any concerns or questions you have with your healthcare provider. They can provide personalized guidance based on your specific circumstances and help you navigate the potential risks associated with the surgery. | 6/25/2023 | Certainly! Weight loss surgery, also known as bariatric surgery, can sometimes be linked to an increased risk of alcohol abuse or problems. There are a few reasons why this might happen:  1. Changes in how alcohol is processed: After weight loss surgery, alcohol can affect the body more quickly and strongly. This means that people may get drunk faster and experience stronger effects from alcohol than they did before surgery.  2. Emotional and psychological changes: Weight loss surgery can have a big impact on a person's emotions and mental well-being. Some people may turn to alcohol as a way to cope with stress, sadness, or body image issues. In these cases, the surgery itself might not be the main cause of alcohol problems, but rather the emotional difficulties people face after the surgery.  3. Existing risk factors: People who undergo weight loss surgery often have a history of obesity, and obesity itself can increase the risk of alcohol problems. Things like genetics, family history, and personal vulnerabilities can make someone more likely to struggle with both obesity and alcohol abuse.  4. Substituting one addiction for another: In some cases, people who had a food addiction before weight loss surgery may end up replacing it with an addiction to alcohol or other substances. This can happen because the underlying psychological issues driving addiction are still present.  It's important to remember that not everyone who has weight loss surgery will develop problems with alcohol. Many people successfully manage their weight and make positive changes without experiencing alcohol-related issues.  To reduce the risks, healthcare professionals closely monitor patients after weight loss surgery and offer support and guidance. They address emotional concerns, provide nutrition advice, and encourage healthy ways to cope with stress.  If you're considering weight loss surgery or have already had the procedure, it's important to talk to your doctor about any concerns or questions you have. They can give you personalized advice and help you navigate the potential risks involved. |
| ASMBS | Does weight loss surgery increase the risk for suicide? | After surgery, most patients have improved mental health but there may be a small increase in the risk of suicide. This is why patients are required to have a mental health screening before surgery.  People affected by severe obesity who are seeking weight loss surgery are more likely to suffer from depression or anxiety and to have lower self-esteem and overall quality of life than someone who is normal weight. Weight loss surgery results in significant improvement in mental health for the majority of patients. However, studies have found a small but significant increase in suicide following weight loss surgery. (7, 8) In some cases, these may have been patients with undiagnosed mental disorders, and in others, patients may have experienced major life stressors that can increase the risk for suicide, but in rare cases, the suicides are unexplained. For this reason, comprehensive bariatric programs require psychological evaluations prior to weight loss surgery. Many programs have therapists available for patient counseling after surgery. In addition, if a patient sees a psychologist or psychiatrist before surgery, they should continue seeing them after surgery. |  | Weight loss surgery, also known as bariatric surgery, is a medical procedure designed to help individuals with severe obesity lose weight. While weight loss surgery can be an effective treatment for obesity-related health issues, including diabetes and cardiovascular diseases, it is important to consider the potential risks and complications associated with any surgical procedure.  Studies have examined the potential link between weight loss surgery and mental health outcomes, including the risk of suicide. While some early studies suggested a potential increase in suicide risk following weight loss surgery, more recent and larger-scale studies have provided contradictory findings. The relationship between weight loss surgery and suicide risk is complex, and additional research is needed to fully understand the factors involved.  It is important to note that severe obesity itself is associated with an increased risk of mental health issues, including depression and suicide. Weight loss surgery may provide physical and psychological benefits for individuals struggling with obesity, improving their overall well-being and potentially reducing the risk of certain mental health problems. However, it is crucial for healthcare professionals to assess and monitor the mental health of individuals considering weight loss surgery and provide appropriate support and resources throughout the process.  If you or someone you know is struggling with mental health issues, including thoughts of self-harm or suicide, it is important to seek help from a healthcare professional or a mental health hotline immediately. | 6/25/2023 | Certainly! Weight loss surgery is a procedure that helps people who have severe obesity lose weight. Some studies have looked at whether this surgery increases the risk of suicide, but the findings are mixed.  In the past, some studies suggested that there might be an increased risk of suicide after weight loss surgery. However, more recent and larger studies have shown conflicting results. It's a complicated issue, and researchers need to do more studies to understand it better.  It's important to know that severe obesity itself can lead to mental health problems, like depression and thoughts of suicide. Weight loss surgery can actually improve people's mental well-being because they feel better physically and emotionally after losing weight.  However, doctors and healthcare professionals should carefully evaluate a person's mental health before and after weight loss surgery. They should provide support and resources to ensure that the person's mental well-being is taken care of throughout the process.  If you or someone you know is struggling with mental health issues or having thoughts of self-harm or suicide, it's important to seek help from a healthcare professional or a mental health hotline right away. |
| ASMBS | Is the chance of dying from weight loss surgery more than the chance of dying from obesity? | No. Weight loss surgery is very safe and decreases the chance of dying from obesity. In fact, weight loss surgery is safer than removing the gallbladder or having a knee replacement.  A recent study of 209,116 patients found the risk of death from weight loss surgery was 0.16%, or approximately 1 in 600. (4) This rate is considerably less than most other surgeries, including gallbladder and hip replacement surgery. Large studies find that the risk of death from any cause is considerably less for patients after weight loss surgery than for those who have severe obesity and have never had the surgery. Patients who have weight loss surgery have a reduction in their risk of death by 40%. Death related to diabetes is reduced by more than 90% and from heart disease by more than 50% for patients after weight loss surgery. (3) The benefits of weight loss surgery far outweigh the risks. As with any serious surgical operation, the decision to have weight loss surgery should be discussed with your surgeon, family members, and loved ones. |  | Weight loss surgery, also known as bariatric surgery, is a medical procedure performed on individuals who are severely obese and have been unable to lose weight through other methods. While weight loss surgery carries certain risks, it is generally considered safe when performed by experienced surgeons in appropriate healthcare settings.  The risk of dying from weight loss surgery varies depending on several factors, including the specific procedure performed, the patient's overall health, and the surgeon's expertise. It is essential to note that the mortality rate associated with weight loss surgery has significantly decreased over the years due to advancements in surgical techniques and improved post-operative care.  On the other hand, obesity itself can have serious health consequences and is associated with a higher risk of various medical conditions such as heart disease, type 2 diabetes, certain cancers, and respiratory disorders. These conditions can potentially lead to premature death.  Comparing the risk of dying from weight loss surgery to the risk of dying from obesity is a complex task. While weight loss surgery carries some inherent risks, it is primarily performed to address the significant health risks posed by obesity. The decision to undergo weight loss surgery is typically made after carefully evaluating the individual's overall health, their ability to lose weight through non-surgical means, and the potential benefits of the procedure.  It's important to consult with a qualified healthcare professional who can assess your specific situation, provide accurate information, and guide you in making an informed decision regarding weight loss surgery or other weight management options. | 6/25/2023 | Certainly! Here's a simplified explanation:  Weight loss surgery is a procedure done to help very overweight people lose weight when other methods haven't worked. Like any surgery, there are risks involved, but it's generally considered safe when performed by experienced doctors.  Being extremely overweight, or obese, can also be dangerous to your health. It increases the chances of developing serious medical conditions like heart disease, diabetes, cancer, and breathing problems, which can lead to premature death.  Comparing the risk of dying from weight loss surgery to the risk of dying from obesity is complicated. Weight loss surgery is done to address the health risks of obesity, but it does carry some risks itself. The decision to have the surgery is made after considering factors like your overall health, your ability to lose weight without surgery, and the potential benefits of the procedure.  It's important to talk to a doctor who can assess your specific situation, give you accurate information, and help you make an informed decision about weight loss surgery or other ways to manage your weight. |
