# Supplementary material for: Large language models and bariatric surgery patient education: a comparative readability analysis of GPT-3.5, GPT-4, Bard, and online institutional resources
Source: Surg Endosc. 2024 Mar 12;38(5):2522–32. doi: 10.1007/s00464-024-10720-2 (PMC11078810; doi:10.1007/s00464-024-10720-2)
Supplement: Supplementary file 3 — Supplementary file3 (DOCX 111 KB) [file 464_2024_10720_MOESM3_ESM.docx]

**Supplementary Table 3**. GPT-4 Responses

| Institution | Question | Institutional Answer | Modified Question | GPT-4 Answer | ChatGPT Entry Date (Month/Day/Year) | “Can you explain that in simpler terms?” |
| --- | --- | --- | --- | --- | --- | --- |
| 1 | **Why should I consider weight loss surgery?** | Weight loss surgery is the most effective way to lose weight and keep weight off. Weight loss surgery can improve or resolve obesity-related conditions such as diabetes, high blood pressure, acid reflux and sleep apnea. It also decreases obesity-related cancer risk by 30%. Weight loss surgery can help improve your overall quality of life. |  | Weight loss surgery, also known as bariatric surgery, is a significant decision that should be carefully considered and discussed with your healthcare providers. It is not the first option for weight loss; typically, it's considered when other options such as diet, exercise, and medication have not worked or when the patient's health is at serious risk due to obesity.  Here are several reasons you might consider weight loss surgery:  1. **Serious health issues related to obesity:** Obesity is associated with a number of serious health conditions such as type 2 diabetes, high blood pressure, heart disease, sleep apnea, gastroesophageal reflux disease (GERD), and certain types of cancer. If your weight is causing or worsening these conditions, weight loss surgery can potentially help alleviate these problems.  2. **Difficulty losing weight through traditional methods:** If you've tried diet, exercise, and medication but have been unable to lose weight or maintain weight loss, weight loss surgery might be an option.  3. **Quality of life:** Excessive weight can limit mobility and interfere with routine tasks. It can also impact your social and emotional health, leading to issues like depression and low self-esteem. If obesity is severely impacting your quality of life, surgery might be an avenue to consider.  4. **Body mass index (BMI):** Weight loss surgery is usually considered for people with a BMI of 40 or higher, or a BMI of 35 or higher along with an obesity-related disease, such as diabetes or high blood pressure.  5. **You're ready to commit to lifestyle changes:** Bariatric surgery is not a quick fix. It requires a lifelong commitment to diet and lifestyle changes in order for it to be effective.  Remember, weight loss surgery isn't for everyone. There are risks and complications associated with these procedures, including infection, bleeding, nutrient deficiencies, and even a small risk of death. Always discuss the risks and benefits with your healthcare provider before deciding on such a significant medical procedure. | 7/2/2023 | Sure, I'd be happy to simplify that for you.  Weight loss surgery is a big operation that helps people lose weight. It's usually considered when other things like diets or exercise haven't worked, or when a person's weight is seriously hurting their health.  Here's why you might think about it:  1. **Health Problems:** Being very overweight can lead to serious health problems, like diabetes, heart disease, and sleep problems. Weight loss surgery can help with these.  2. **Trouble Losing Weight:** If you've tried hard to lose weight by eating better and exercising but it didn't work, this surgery could be an option.  3. **Quality of Life:** If your weight is causing problems in your life, like making it hard to move around or causing you to feel sad or left out, then surgery might help.  4. **Body Mass Index:** This is a number that doctors use to measure obesity. If your number is very high (40 or above), or a bit lower (35 or above) but you also have other health problems, then weight loss surgery might be an option.  5. **Ready for Change:** The surgery isn't a magic fix. Afterward, you'll still need to eat healthily and stay active. It's important to be ready to make these life changes.  But remember, this surgery can also have risks and side effects, like getting infections, bleeding, or not getting enough nutrients from food. So, it's important to talk with your doctor about these things before deciding. |
| 1 | What are my options for weight loss surgery at X? | Our surgeons offer Roux-en-Y gastric bypass, sleeve gastrectomy and the laparoscopic adjustable gastric band. We also offer revision surgery for patients who have had weight loss surgery before and have not achieved desired results. |  | X is a leading healthcare institution based in X, and they typically provide a range of bariatric surgery options. As of my last update in September 2021, these are the typical types of weight loss surgeries you could expect:  1. **Gastric Bypass (Roux-en-Y):** This is a procedure where the surgeon creates a small pouch at the top of the stomach and connects it directly to the middle portion of the small intestine, bypassing the rest of the stomach and the upper portion of the small intestine.  2. **Sleeve Gastrectomy:** In this procedure, about 80% of the stomach is removed, leaving a tube-shaped "sleeve" that can hold much less food. This helps you feel full sooner.  3. **Adjustable Gastric Banding (Lap-Band):** A band is placed around the upper part of the stomach to create a small pouch that can hold less food. The band's tightness can be adjusted over time to control how quickly food leaves the pouch.  4. **Biliopancreatic Diversion with Duodenal Switch (BPD/DS):** A portion of the stomach is removed, but the remaining part is connected to the lower portion of the small intestine. This bypasses a significant part of the small intestine, limiting calorie absorption.  5. **Revisional Bariatric Surgery:** If a previous weight loss surgery has failed, a revision surgery might be performed to adjust the initial procedure or to switch to a different procedure.  Please note that the availability of these procedures can vary and may have changed since my last update in 2021. Not all patients are candidates for all procedures; the best procedure for you would depend on various factors such as your current health status, your weight loss goals, and your surgeon's recommendation.  Remember to consult with your healthcare provider for the most accurate and up-to-date information. Your doctor can help you understand the risks and benefits of each procedure and determine which one might be best for you. | 6/25/2023 | Of course! Here's a simpler explanation of the different weight loss surgeries you might find at X or similar hospitals:  1. **Gastric Bypass (Roux-en-Y):** The doctor makes a small pouch in your stomach. They then connect this pouch directly to the middle of your small intestines. This means food skips most of your stomach and some of your small intestines, so you absorb fewer calories.  2. **Sleeve Gastrectomy:** The doctor removes most of your stomach, leaving just a small part that looks like a tube or "sleeve". This smaller stomach can't hold as much food, so you feel full more quickly.  3. **Adjustable Gastric Banding (Lap-Band):** The doctor puts a band around the top part of your stomach. This creates a small pouch that fills up quickly, so you don't feel like eating as much. The doctor can tighten or loosen the band over time to change how much food you can eat.  4. **Biliopancreatic Diversion with Duodenal Switch (BPD/DS):** The doctor removes part of your stomach and connects the remaining part to the lower part of your small intestines. This means food skips a large part of your small intestines, so you don't absorb as many calories.  5. **Revisional Bariatric Surgery:** If you've had weight loss surgery before but it didn't work or caused problems, the doctor might do another surgery to fix it or try a different kind.  Remember, these surgeries can be very helpful for some people, but they also have risks. It's important to talk to a doctor to understand if one of these surgeries might be a good option for you. They can help you understand the benefits and risks. |
| 1 | Who is eligible for weight loss surgery? | To qualify for weight loss surgery, you must have either a:  - BMI of 40 or greater  - BMI of 35-40 with obesity-related conditions such as diabetes, high blood pressure, sleep apnea or gastroesophageal reflux disease (GERD) |  | Weight loss surgery, also known as bariatric surgery, is a serious surgical procedure designed to help individuals who struggle with obesity and have had difficulty losing weight through more traditional methods such as diet and exercise. Bariatric surgery can lead to significant weight loss and improve many obesity-related conditions, such as type 2 diabetes or high blood pressure.  As of my last training data in September 2021, the general eligibility criteria for weight loss surgery include:  1. **Body Mass Index (BMI):** Individuals who have a BMI of 40 or more, which is classified as extreme or severe obesity. Some individuals may be eligible with a BMI between 35 and 39.9 (classified as obesity) if they also have a serious weight-related health problem such as diabetes, high blood pressure, or severe sleep apnea.  2. **Failed Attempts at Other Weight Loss Methods:** Typically, before considering surgery, individuals should have tried but failed to lose weight through diet, exercise, or medication.  3. **Health Evaluation:** A thorough health evaluation will be conducted to assess the individual's overall health, the risks of surgery, and the potential benefits of the procedure. This often includes physical examinations, lab tests, nutritional evaluation, psychological evaluation, and in some cases, consultations with specialists (like cardiologists, pulmonologists, etc).  4. **Understanding and Commitment:** Individuals must understand the risks and benefits of the surgery, and be committed to lifelong changes in their diet, exercise, and overall lifestyle. This includes regular follow-up visits for monitoring and support.  5. **Age:** Although there's no specific age limit for weight loss surgery, the safety and effectiveness may vary, and the individual's overall health and well-being will be taken into account. In general, it's considered for adults, but in some cases, it might be an option for adolescents with severe obesity.  Please note, these guidelines might vary among different health organizations and in different countries, and more updated guidelines may have been released after my training data in 2021. Therefore, if you or someone you know is considering bariatric surgery, it's important to consult with a healthcare professional or a bariatric surgeon to understand the most current eligibility criteria and to discuss if it's the right choice. | 6/25/2023 | Sure! Weight loss surgery is a big operation that helps people who are very overweight and can't lose weight with just diet and exercise. Here's who usually can get weight loss surgery:  1. **Weight:** People who are really overweight. This is often measured with something called BMI (Body Mass Index). For most people to get the surgery, their BMI needs to be 40 or more. If their BMI is a little lower (35-40), they can still get the surgery if they also have health problems like diabetes or high blood pressure because of their weight.  2. **Trying Other Ways First:** Usually, the person should have tried to lose weight with diet, exercise, or medicine and it didn't work.  3. **Health Check:** The doctor will do a lot of checks to make sure the person is healthy enough for the surgery and that the surgery can help them.  4. **Ready for Change:** The person needs to understand what the surgery involves, and be ready to change their eating and exercise habits for life. They will also need to see the doctor regularly after surgery.  5. **Age:** The surgery is mostly for adults. But sometimes, teenagers who are very overweight might get it too.  But remember, the person should talk to their doctor to know if the surgery is a good choice for them. |
| 1 | What is the recovery time after weight loss surgery? | On average, most patients stay in the hospital one to two days after surgery. Most return to work two to three weeks after surgery. |  | The recovery time after weight loss surgery can vary greatly depending on the type of surgery performed, the individual patient's health status, and their commitment to post-operative care and lifestyle changes. As of my knowledge cutoff in 2021, here are the general timeframes for the most common types of weight loss surgery:  1. Gastric Bypass: After a Roux-en-Y gastric bypass surgery, patients usually stay in the hospital for 2 to 3 days, and full recovery typically takes 2 to 3 weeks.  2. Sleeve Gastrectomy: For a sleeve gastrectomy, the hospital stay is usually 1 to 2 days, with a full recovery time of 2 to 3 weeks.  3. Adjustable Gastric Banding: This surgery typically involves a hospital stay of less than 24 hours (often outpatient), and full recovery typically takes about 1 week.  4. Biliopancreatic Diversion with Duodenal Switch (BPD/DS): This complex surgery often requires a hospital stay of 2 to 4 days, with a full recovery time of 3 to 4 weeks.  Remember, "full recovery" means returning to normal activities. However, these surgeries require long-term lifestyle changes to diet and physical activity. It's important to consult with your healthcare provider for personalized information, as recovery times can vary based on individual health circumstances. Also, developments and advancements may have occurred in the field of weight loss surgery after my last training data in September 2021. | 6/25/2023 | Sure, I'll simplify it.  Weight loss surgeries help people lose weight but recovery time is different for each type:  1. Gastric Bypass: After this surgery, you would usually stay in the hospital for 2 to 3 days. You should feel fully recovered in about 2 to 3 weeks.  2. Sleeve Gastrectomy: After this surgery, you'd stay in the hospital for 1 to 2 days. Full recovery usually takes 2 to 3 weeks.  3. Adjustable Gastric Banding: This surgery often lets you go home the same day. You should feel better and be able to do your normal activities in about a week.  4. Biliopancreatic Diversion with Duodenal Switch: This complex surgery might keep you in the hospital for 2 to 4 days. It may take 3 to 4 weeks to fully recover.  "Full recovery" means you can go back to your usual activities. But remember, these surgeries mean you have to make long-term changes to what you eat and how much you exercise. Always talk to your doctor to understand what's best for you. And remember, this information is based on data up until 2021, and there might be newer techniques or advancements now. |
| 1 | How much weight can I expect to lose after surgery and how quickly? | Maximum weight loss typically occurs 12-24 months after surgery. Individual weight loss varies depending on:  - Age  - Weight before surgery  - Level of physical activity  - Diet  - Type of surgery | How much weight can I expect to lose after weight loss surgery and how quickly? | The amount of weight that you can expect to lose and the rate at which you lose it after weight loss surgery will depend on several factors. These include the type of weight loss surgery, your starting weight, your diet and exercise habits post-surgery, and your overall commitment to a new lifestyle.  1. **Type of surgery:** Different weight loss surgeries tend to produce different results.  - Gastric Bypass (Roux-en-Y): On average, people lose about 60 to 80% of their excess weight over a period of 12 to 18 months.  - Sleeve Gastrectomy: People typically lose around 50 to 70% of their excess weight within a year or two following surgery.  - Adjustable Gastric Band (Lap-Band): This typically results in slower and less overall weight loss, with patients losing about 40 to 60% of their excess weight over a period of 2 to 3 years.  2. **Rate of weight loss:** Most rapid weight loss occurs within the first six months to a year after surgery. After this initial period, weight loss tends to slow down.  3. **Post-surgery habits:** After weight loss surgery, it is essential to follow a proper diet and exercise regularly to maintain weight loss. Failing to follow these guidelines can slow down the rate of weight loss or even cause weight regain.  Remember, weight loss surgery is not a magic solution. It's a tool that can aid in weight loss, but its effectiveness largely depends on the patient's commitment to maintaining lifestyle changes post-surgery. It's also important to note that the success of weight loss surgery is usually measured not just in pounds lost, but also in improvements to overall health, such as reductions in diabetes and hypertension.  Always consult with your healthcare provider or a bariatric specialist to understand what you can realistically expect in your personal circumstances. The figures given here are averages and individual results can vary significantly. | 6/25/2023 | Of course! After weight loss surgery, most people lose weight pretty quickly for the first 6 months to a year. Then, the weight loss slows down.  - If you have Gastric Bypass surgery, you might lose between 60-80% of your extra weight in the first 12 to 18 months.  - If you have Sleeve Gastrectomy surgery, you might lose around 50-70% of your extra weight within a year or two.  - If you get a Gastric Band (or Lap-Band), the weight loss is slower. You might lose 40-60% of your extra weight over 2 to 3 years.  But remember, everyone is different, and your weight loss could be more or less. Also, you need to eat a healthy diet and exercise regularly after the surgery to help lose weight and keep it off.  Weight loss surgery isn't a quick fix. It's a tool to help you lose weight. But you also need to change your lifestyle for the surgery to work well. |
| 1 | What should I expect after surgery? | You will need to follow a bariatric diet and exercise daily, take bariatric vitamins lifelong, refrain from using nicotine (including cigarettes) and minimize alcohol use. | What should I expect after weight loss surgery? | Weight loss surgery, also known as bariatric surgery, can help you lose a significant amount of weight, but it's not a one-step solution. It is an ongoing journey that involves significant lifestyle changes. Here's what you can generally expect after undergoing weight loss surgery:  1. **Immediate Post-Surgery Recovery**: You will likely stay in the hospital for a few days after your surgery for monitoring. You may experience some pain and discomfort in the immediate aftermath, which can be managed with medication. Depending on the surgery, you may also have drains and catheters in place.  2. **Dietary Changes**: Immediately after surgery, you'll start on a liquid diet and gradually progress to pureed food, then soft foods, and eventually, after several weeks, solid foods. It's important to eat protein-rich foods to help with healing. You will also need to take vitamins and minerals to prevent deficiencies.  3. **Physical Activity**: Exercise is a crucial part of recovery and maintaining weight loss after surgery. You'll likely be encouraged to start walking shortly after surgery, and gradually increase your activity level over the following weeks and months. Regular physical activity not only supports weight loss but also helps with overall health.  4. **Weight Loss**: The amount and rate of weight loss can vary depending on the type of surgery and your individual circumstances, but most people lose weight rapidly in the first six months to a year after surgery.  5. **Regular Follow-Up**: You'll need regular follow-up appointments with your healthcare team to monitor your recovery and weight loss, and to make sure you're getting the necessary nutrients.  6. **Psychological Changes**: Many people experience a range of emotions after surgery, from elation at the weight loss to depression or anxiety. Psychological support, such as counseling or support groups, can be very beneficial.  7. **Long-term Lifestyle Changes**: You will need to make long-term changes to your diet and exercise habits to maintain your weight loss. This might include smaller meal portions, nutrient-dense foods, regular physical activity, and possibly counseling to help address any emotional or behavioral issues related to food.  8. **Possible Complications**: As with any surgical procedure, there can be complications. These might include infection, blood clots, and leaks in your gastrointestinal system. Longer term, some people experience malnutrition, gallstones, hernias, or low blood sugar.  Remember that everyone's experience with weight loss surgery is unique, so it's crucial to discuss with your healthcare provider what you can expect based on your personal health history and the specific type of surgery you're having. | 6/25/2023 | Of course, here's a simplified version of what might happen after weight loss surgery:  1. **Recovery**: After the surgery, you will stay in the hospital for a few days. You might feel some pain, but doctors will give you medicine to help.  2. **Eating Habits**: You'll start with liquids like soup, then move to pureed food (like baby food), then soft foods, and finally normal foods after a few weeks. You'll need to eat foods high in protein and take vitamins to keep healthy.  3. **Exercise**: You'll be asked to move around and walk a little bit soon after surgery. As time goes on, you'll need to exercise more to help with weight loss and to keep healthy.  4. **Losing Weight**: You'll start losing weight pretty quickly after surgery, especially in the first 6 months to a year.  5. **Doctor Visits**: You'll need to visit your doctor regularly to check how you're doing, how much weight you're losing, and if your body is getting all the nutrients it needs.  6. **Feelings**: You might feel all sorts of things after surgery. You might be really happy about losing weight, or you might feel sad or worried. It can be helpful to talk to a counselor or a support group about these feelings.  7. **Life Changes**: You'll need to eat smaller meals and exercise regularly to keep the weight off. You might also need to see a counselor to talk about how you feel about food and eating.  8. **Risks**: Like any surgery, there can be problems. These might include infections, blood clots, or leaks in your stomach. Later on, you might get gallstones or hernias, or you might not get all the nutrients you need.  Remember, everyone's different, so what happens to you might not be exactly like this. Always talk to your doctor about what you can expect. |
| 1 | What is body mass index (BMI), and how can I calculate mine? | Body mass index (BMI) is a tool to screen for obesity, factoring in your height and weight. Learn your BMI with our BMI calculator. |  | Body Mass Index (BMI) is a widely used metric that helps estimate a healthy body weight based on a person's weight and height. It's used as a screening tool to identify possible weight issues in adults. However, it's not a diagnostic tool - if you're concerned about your weight, it's best to consult with a healthcare professional.  Here's how you can calculate your BMI:  1. First, measure your weight in kilograms (kg).  2. Then, measure your height in meters (m).  3. Finally, use the formula: BMI = weight(kg) / [height(m)]^2  For example, if your weight is 70kg and your height is 1.75m, your BMI would be:  BMI = 70 / (1.75)^2 = 22.9  The resulting value is interpreted as follows:  - Underweight: BMI is less than 18.5.  - Normal weight: BMI is 18.5 to 24.9.  - Overweight: BMI is 25 to 29.9.  - Obesity: BMI is 30 or more.  Remember, the BMI is a simple tool that does not take into account muscle mass or distribution of fat, among other factors. It can give you a general idea of whether you're in a healthy weight range, but it's not definitive or individualized. Always consult with healthcare professionals for personalized advice regarding your health. | 6/25/2023 | Sure, I can simplify it for you:  BMI is a number that can tell us if we are a healthy weight for our height.  To calculate it, you need to know how much you weigh and how tall you are.  Here are the steps:  1. You'll need your weight in kilograms (kg). If you know your weight in pounds, you can convert it by multiplying it by 0.45.  2. Then, you'll need your height in meters (m). If you know your height in inches, you can convert it by multiplying it by 0.0254.  3. Take your weight and divide it by your height squared (your height times itself).  The number you get is your BMI.  If your BMI is less than 18.5, you're considered underweight. If it's between 18.5 and 24.9, you're at a healthy weight. If it's between 25 and 29.9, you're considered overweight. If it's 30 or above, you're considered obese.  Remember, this is a simple check. If you have concerns about your weight, you should speak with a doctor. |
| 2 | Can bariatric surgery be reversed? | Laparoscopic gastric banding surgery is reversible in a minimally invasive manner. Gastric bypass surgery is potentially reversible. Reversal requires another operation of the same, or greater, magnitude with the same, or greater, risks. |  | Yes, some forms of bariatric surgery can be reversed, but this depends on the type of procedure that was originally performed.  1. Adjustable Gastric Banding (Lap-Band): This procedure is reversible. The band that is placed around the stomach can be removed, returning the stomach to its original size and shape.  2. Gastric Bypass (Roux-en-Y): Reversing this procedure is technically possible but it is a complex and risky operation. This is due to the significant rearrangement of the digestive system. Due to these risks, reversal is usually only considered in cases of severe complications.  3. Sleeve Gastrectomy: This procedure involves removing a large portion of the stomach and it is not reversible because the removed portion of the stomach cannot be replaced.  4. Duodenal Switch: This is a more complex procedure that is more difficult to reverse, given that it involves both a reduction in stomach size and rerouting of the small intestine.  It's important to note that reversal of bariatric surgery is typically a last resort. This is not only due to the technical difficulty and inherent risks of the procedure, but also because reversal often leads to weight regain and the return of obesity-related health problems.  As with all medical procedures, it's crucial to discuss all the risks and benefits with a healthcare provider before making a decision. It's also important to remember that bariatric surgery is typically accompanied by lifestyle modifications, including diet and exercise, to ensure its success. | 7/2/2023 | Sure, I can simplify that for you!  1. Adjustable Gastric Banding (Lap-Band): This is like a belt around your stomach to make it smaller. It can be taken off if needed.  2. Gastric Bypass (Roux-en-Y): This operation makes your stomach smaller and changes the path of your food in your body. It's possible to undo it, but it's difficult and can be risky.  3. Sleeve Gastrectomy: This surgery cuts off a part of your stomach to make it smaller. This can't be undone because the part that's removed can't be put back.  4. Duodenal Switch: This complex surgery makes your stomach smaller and also changes where your food goes in your body. Undoing this is difficult.  Undoing (reversing) these surgeries is usually only done if there are serious problems because it's risky and can lead to gaining the weight back. It's very important to remember that these surgeries need to go along with healthy eating and regular exercise to work well. As always, a person should talk about the pros and cons with their doctor before deciding. |
| 2 | What if I need revision surgery? | Weight loss surgery is not a magic bullet and while most patients are successful after weight loss surgery, there are instances where revision weight loss surgery is required. Whether reasons for seeking revision weight loss surgery are due to inadequate weight loss/weight regain, unresolved co-morbidities and/or medical complications, there is possibly a revision solution to these problems.  Revisional bariatric operations, regardless of the previous surgery, are always challenging for a surgeon. They require adequate expertise to achieve desired results. Bariatric Centers of Excellence certified by the American Society for Bariatric Surgery provide a multi-disciplinary team and facilities for managing patients requiring revision of their surgery. It is highly recommended that patients requiring gastric bypass revision or any other kind of revisional surgery find a surgeon at a bariatric center that has much experience with revisional bariatric surgery. | What if I need revision surgery after weight loss surgery? | If you need revision surgery after weight loss surgery, there are a few things you should know.  First, it's important to note that the need for revision surgery after weight loss surgery is not uncommon. This can occur for a variety of reasons such as insufficient weight loss, weight regain, or complications related to the original surgery such as ulcers, strictures, or device-related issues in the case of adjustable gastric bands.  Here are some steps and considerations if you're in this situation:  1. **Discussion with Your Healthcare Provider:** Your first step should always be to discuss your concerns with your primary healthcare provider or your bariatric surgeon. They will help you understand why you may need a revision surgery and what your options might be.  2. **Understand the Procedure:** Revisional bariatric surgery is typically more complex than the initial operation due to alterations in the normal anatomy and the presence of scar tissue from the first surgery. Consequently, it carries a higher risk of complications. Ensure you have a detailed discussion about these risks and benefits with your surgeon.  3. **Explore Other Options:** Before opting for revision surgery, consider exploring other options such as diet modification, increasing physical activity, or addressing any behavioral or emotional issues that may be contributing to weight regain or insufficient weight loss.  4. **Preparation for the Surgery:** If you and your surgeon agree that revision surgery is the best option, you'll need to go through a similar process to the one you experienced for your first surgery. This usually involves nutritional counseling, psychological evaluation, and various medical tests.  5. **Post-Surgery Care:** Just like with the original surgery, post-operative care will be essential. This might involve nutritional counseling, support groups, and regular follow-ups with your healthcare provider.  Remember, every patient's situation is unique. It's important to have these discussions with a healthcare provider who knows your specific situation. This information should provide a good starting point for those discussions. | 7/2/2023 | Of course, I'd be happy to explain that in simpler terms.  Sometimes, the first weight loss surgery might not work as well as expected. Maybe it didn't help you lose enough weight, or you started to gain weight back, or it caused other health problems. When this happens, you might need a second surgery, which is called a revision surgery.  Here are the steps you would follow:  1. **Talk to Your Doctor:** Tell them what's happening and ask them what you should do.  2. **Learn About the Surgery:** The second surgery might be more difficult and could have more risks because your body has already been through one surgery. Make sure you ask your doctor about this.  3. **Think About Other Options:** Before deciding on a second surgery, think about whether there are other things you can do. Could you eat healthier or exercise more? Are there emotional issues that need to be addressed?  4. **Get Ready for the Surgery:** If you and your doctor decide that another surgery is the best choice, you'll need to prepare just like you did for the first one. This means you might need to talk to a dietitian or a counselor, and you'll have some medical tests.  5. **Take Care of Yourself After Surgery:** Just like the first time, taking care of yourself after the surgery is very important. This might include eating a healthy diet, attending support groups, and seeing your doctor regularly.  Remember, each person's experience is different. It's very important to talk to your own doctor, who knows your personal situation and can give you the best advice. |
| 2 | What is my ideal body weight? | Xs Bariatric and Metabolic Institute follows the guidelines set out by the body mass index scale. This scale recommends a BMI of between 18.5 and 24.9. You can use the calculator above to find your current BMI and ideal BMI. |  | To calculate your ideal body weight, I'd need more information such as your height, gender, and body frame size. It's also worth noting that what's considered "ideal" can vary greatly from person to person, as it depends on factors like muscle mass, body fat percentage, bone density, and overall health.  However, there are common methods to calculate a range of healthy weight, one of which is the Body Mass Index (BMI). For most adults, a BMI in the range of 18.5 to 24.9 is considered to be healthy. However, the BMI does not differentiate muscle from fat and doesn't take into consideration other factors such as age, gender, and muscle mass. This means it can sometimes be inaccurate, particularly for athletes or people with a lot of muscle mass.  Another method of determining a healthy weight range is by using the Hamwi method, which takes into account a person's gender and height.  For men:  - Start with 106 pounds for 5 feet in height.  - Add 6 pounds for every inch over 5 feet.  For women:  - Start with 100 pounds for 5 feet in height.  - Add 5 pounds for every inch over 5 feet.  This method then suggests that a healthy weight range can be +/- 10% from the calculated weight.  Please consult with a healthcare professional to get a better understanding of what might be an ideal body weight for you, based on your personal health profile and goals. It's important to focus on overall health and fitness rather than just weight alone. | 7/2/2023 | Of course, I'd be happy to simplify!  Your ideal body weight can depend on a lot of things like how tall you are, whether you're a man or a woman, and how much muscle you have.  A common way to check if your weight is healthy is called BMI, or Body Mass Index. It uses your height and weight to give you a number. For adults, if your BMI is between 18.5 and 24.9, it usually means you're at a healthy weight.  Another method is called the Hamwi method. It also uses your height, but the calculations are a little different. Here's how you do it:  For men:  - If you're 5 feet tall, the calculation starts at 106 pounds.  - For every inch taller than 5 feet, add 6 pounds.  For women:  - If you're 5 feet tall, the calculation starts at 100 pounds.  - For every inch taller than 5 feet, add 5 pounds.  The result is your ideal weight. But people can healthily be 10% lighter or heavier than that number, too.  But remember, it's not just about weight. It's about being healthy overall. And everyone is different, so these numbers may not be perfect for everyone. If you're unsure, it's always a good idea to talk to a doctor or a dietitian. They can give you advice that's specific to you. |
| 2 | Is there support from surgeons and staff following weight loss surgery? | X's gastric bypass surgery health care team is there to guide patients after each procedure. Regular follow-up visits are scheduled during the first year after weight loss surgery to check overall physical and mental health, metabolism and nutritional status. |  | Yes, typically, a comprehensive post-operative care program is provided following weight loss surgery (also known as bariatric surgery). The specifics can vary depending on the healthcare provider, the type of surgery, and the patient's individual needs, but the care program often includes:  1. **Follow-up appointments with the surgeon:** These allow for the surgeon to assess your recovery, evaluate your progress, and address any complications or concerns.  2. **Nutritional counseling:** Given the significant changes to the digestive system following bariatric surgery, patients often require guidance from a dietitian or nutritionist to help them adapt their diet and ensure they are meeting their nutritional needs.  3. **Physical therapy or exercise guidance:** As patients lose weight and their mobility increases, they may benefit from a guided exercise program to help improve their strength and cardiovascular fitness.  4. **Psychological support:** Significant weight loss and lifestyle changes can bring about emotional challenges. Many programs include access to mental health professionals to support patients through these changes.  5. **Support groups:** Connecting with others who have undergone similar experiences can provide emotional support, shared strategies for coping with lifestyle changes, and a sense of community.  6. **Long-term follow-up care:** Bariatric surgery isn't a quick fix; it's a tool that can facilitate long-term weight loss when combined with lifestyle changes. Regular follow-ups over the long term help track progress and adjust the plan as needed.  Remember, each patient's journey with weight loss surgery is unique. Some may require more intensive support in certain areas than others. It's important to communicate openly with your healthcare team to ensure you're getting the support you need. | 7/2/2023 | Absolutely! After weight loss surgery, you'll have a lot of help to make sure you're doing well and are on the right track. Here's what you can expect:  1. **Check-ups with your surgeon:** They will see how you're healing and answer any questions.  2. **Diet advice:** Since your stomach has changed, you'll need to learn how to eat in a new way. A diet expert can help you with this.  3. **Exercise help:** You might need help learning the best way to exercise as you start losing weight.  4. **Emotional support:** Big changes can be tough to handle emotionally. Talking to a mental health professional can help a lot.  5. **Support groups:** You can meet with people who are going through the same thing. They can offer support, tips, and friendship.  6. **Long-term check-ups:** Losing weight takes time. Regular visits with your doctor will help you stay on track.  Remember, it's okay to tell your doctor or nurse if you need more help. They're there to support you on this journey. |
| 3 | How do I know if I am eligible? | Patients must meet specific criteria before they will be considered for an evaluation with the X Bariatric & Metabolic Surgery program.  - Have a Body Mass Index (BMI) of greater than 40, or a BMI of35 accompanied by other issues related to health (co-morbidities). Calculate your BMI. These co-morbidities include diabetes, high blood pressure, coronary artery disease, obstructive sleep apnea, osteoarthritis, hyperlipidemia (high cholesterol, triglycerides, etc), metabolic syndrome, polycystic ovarian disease, pseudotumor cerebri, etc.  - Be mobile, medically stable, and have an actively involved and supportive primary care physician.  - Have a stable psychological status with adequate social support system to help them through the course of surgery and the postoperative period. | How do I know if I am eligible for weight loss surgery? | If you're considering weight loss surgery, also known as bariatric surgery, you should consult with your healthcare provider or a bariatric specialist who can provide comprehensive information based on your individual health profile. However, general criteria often include:  1. **Body Mass Index (BMI):** Typically, eligibility for weight loss surgery includes having a body mass index (BMI) of 40 or higher (known as severe or morbid obesity). In some cases, a person with a BMI of 35 or higher and a serious weight-related health problem such as type 2 diabetes, heart disease, or severe sleep apnea might also be a candidate.  2. **Failed attempts at diet and exercise:** Usually, weight loss surgeries are considered for people who have tried but failed to lose weight and improve their health through diet and exercise.  3. **Medical necessity:** Some people might need surgery if their weight is causing serious health problems. For example, if someone has type 2 diabetes that isn't well-controlled with lifestyle changes and medicine, weight loss surgery could be an option.  4. **Understanding the risks and commitment:** You must understand the potential risks and complications of weight loss surgery, and be committed to lifelong changes in eating and physical activity habits.  5. **Age:** While there's not a strict upper age limit for bariatric surgery, the overall health risks can increase with age. The procedure is usually not performed on young children or teenagers, but there may be exceptions in cases of severe health problems related to obesity.  6. **Psychological status:** Patients should not have an untreated mental health disorder that could interfere with the pre-operative preparation period, surgical treatment, or post-operative care.  7. **Support system:** It is generally encouraged to have a support system in place to help with the changes in diet and lifestyle following the surgery.  These are general criteria and may vary slightly depending on the specific surgical procedure and the policies of your healthcare provider or insurance company. Always consult with a healthcare provider to understand your personal eligibility. | 7/2/2023 | Sure, let's break it down in simpler terms:  1. **Your weight:** You might be able to get weight loss surgery if you are very overweight. Doctors often use a measure called Body Mass Index (BMI) to decide. You might be eligible if your BMI is 40 or more. If your BMI is 35 or more and you have health problems like diabetes or heart disease, you might also be eligible.  2. **Other methods didn't work:** Weight loss surgery is usually considered if you've tried to lose weight in other ways (like diet and exercise) but it didn't work.  3. **Your health:** If being overweight is causing you serious health problems, surgery might be an option to help you.  4. **Understanding and commitment:** You need to know that there are risks with the surgery. Also, you have to be ready to make big changes to how you eat and how active you are for the rest of your life.  5. **Your age:** There isn't a strict rule on age, but the surgery is usually not done on very young people or very old people because of the risks.  6. **Mental health:** You should be in a good place mentally. If you have untreated mental health issues, it could make the process harder.  7. **Support from others:** It's good to have people who can help and support you as you go through surgery and make changes afterwards.  Remember, these are general ideas. If you think weight loss surgery might be right for you, talk to a doctor. They can give you advice based on your personal health. |
| 3 | Do I have to wait to get pregnant after bariatric surgery? Will the baby be healthy? | Women generally have significantly increased fertility after weight loss surgery. Birth control pills do NOT work as well in heavy patients and during the time of rapid weight loss. We strongly recommend all child bearing age women to use other ways of contraception after bariatric surgery.  Most surgeons recommend waiting 12-18 months after weight loss surgery before getting pregnant. After losing most of the excess weight, there is much less risk of having problems during pregnancy (gestational diabetes, eclampsia, macrosomia) and during childbirth. There is also decreased risk of having miscarriages and stillbirths. The kids born after mon’s bariatric surgery are less prone to being obese later in their life. Bariatric surgery also reduces the chance of needing a C-section. |  | Yes, it is generally recommended to wait at least 12 to 18 months after bariatric surgery before trying to conceive. This period allows your body to stabilize post-surgery and ensures that you are no longer in the rapid weight loss phase, which can affect nutrition.  During this waiting period, you should focus on reaching a stable and healthy weight, establishing a regular exercise routine, and ensuring that your nutrition is balanced and sufficient. It's important to work with your healthcare provider to monitor your nutritional status and make sure you are getting enough vitamins and minerals, such as iron, calcium, and vitamin B12, which are crucial for a healthy pregnancy.  As for the health of the baby, assuming you're getting proper nutrition and care, there's no reason to believe that having bariatric surgery should negatively affect the health of your baby. In fact, losing weight prior to pregnancy can actually improve outcomes for both mother and baby, especially if you were in an obese category before surgery. Obesity can increase the risk of complications such as gestational diabetes and pre-eclampsia, so losing weight can be beneficial.  However, every person is unique, so these general recommendations may not apply to everyone. It's important to discuss your specific situation and plans with your healthcare provider or a specialist in maternal-fetal medicine. They can provide advice based on your medical history, current health status, and personal goals. | 7/2/2023 | Of course!  If you have had bariatric surgery (a surgery to help you lose weight), it's a good idea to wait about 1 to 1.5 years before trying to have a baby. This gives your body time to get used to the changes from the surgery and for your weight to become stable. During this time, it's important to eat healthy foods and do regular exercise.  When you lose weight and become healthier, it can actually make pregnancy safer for both you and the baby. If you were very overweight before, losing weight can lower your chances of having problems during pregnancy.  But remember, everyone is different. It's always best to talk with your doctor about when it's safe for you to try to get pregnant after surgery. They can give you advice that fits your own health and needs. |
| 3 | How long will I stay in the hospital after bariatric surgery? | All surgeries are performed at the new X Medical Center. The average surgical time for gastric bypass is about 2 hours and sleeve gastrectomy is about 1-2 hours. The laparoscopy totally changed bariatric surgery. Almost all our patients can walk a few hours after surgery. They start to have liquids the day after surgery. The majority (more than 95%) of patients only stay in the hospital for one night or two nights. |  | The length of your hospital stay after bariatric surgery can vary based on several factors, including the type of surgery performed, your overall health, the surgeon's preferences, and how quickly you recover.  Here are some general timelines for various types of bariatric surgery:  1. Gastric bypass: Hospital stay is typically 2 to 3 days, but some patients may need to stay longer.  2. Sleeve gastrectomy: Hospital stay is typically 1 to 2 days.  3. Laparoscopic adjustable gastric banding: Hospital stay is typically less than 24 hours, and some procedures may be performed on an outpatient basis.  4. Duodenal switch: Hospital stay is typically 2 to 4 days.  These are just general timelines and can vary significantly based on individual circumstances. Also, after leaving the hospital, you'll need a period of recovery at home and multiple follow-up appointments with your healthcare team.  Your doctor will give you more precise information based on your health status, the specifics of the procedure, and your recovery progress. Always follow your doctor's advice for optimal recovery. | 7/2/2023 | Sure, here's a simpler explanation:  If you're getting weight loss surgery, you might need to stay in the hospital for a little while after. The time you'll stay depends on what kind of surgery you're getting and your health. Here are some general ideas:  1. Gastric bypass: This surgery might require you to stay in the hospital for 2 to 3 days.  2. Sleeve gastrectomy: After this surgery, you may stay in the hospital for 1 to 2 days.  3. Gastric banding: This one might not need a hospital stay at all, or maybe just overnight.  4. Duodenal switch: With this surgery, you might need to stay in the hospital for 2 to 4 days.  Remember, these are just general ideas and it can change depending on your health and your specific situation. After the surgery, you will need some time at home to get better and you'll need to go to a few check-ups with your doctor. Your doctor will tell you more about what you can expect. |
| 3 | When can I go back to work after surgery? | After surgery, most patients return to work after two to four weeks. Some may be able to work from home as early as one week after surgery. Because of the significantly reduced calorie intake, many patients have low energy for 1-2 weeks after surgery. However, most patients gain their energy back once they start to have soft food. | When can I go back to work after weight loss surgery? | The timeframe for returning to work after weight loss surgery depends on several factors including the type of surgery you had, your overall health, the physical demands of your job, and your body's recovery process.  For less physically demanding jobs, such as office work, you may be able to return to work after 1-2 weeks. If your job involves more physical activity, you might need to wait 2-6 weeks, or possibly longer, depending on your surgeon's guidance.  Remember, you're likely to have dietary restrictions and may still be adjusting to your new eating habits, so it's important to make sure you have the resources and time needed to manage this at work.  This is a general guideline and individual circumstances can vary greatly. Always consult with your surgeon or healthcare provider for personalized advice related to your recovery and return to work.  Also, during your recovery process, it's essential to follow post-operative instructions, eat a healthy diet, get plenty of rest, and gradually reintroduce physical activity as recommended by your healthcare provider. | 7/2/2023 | Sure! After weight loss surgery, you need some time to rest and heal before going back to work. If your job involves sitting at a desk most of the time, you might be able to go back to work in about 1 to 2 weeks. If your job requires more physical work, you may need to wait 2 to 6 weeks, or maybe even longer.  It's important to listen to what your doctor tells you about when you can go back to work. Also, you will need to follow the new eating rules from your doctor, so you have to be ready to manage that when you're back at work.  Everyone heals at a different pace, so what's most important is to take care of yourself and not rush back to work before you're ready. |
| 3 | How bad is the pain after surgery? | Most patients have some pain after surgery. However, the pain is usually just a little bit more than laparoscopic gallbladder surgery. Most patients just need to take oral pain medications for a few days after surgery. The majority of our patients felt that pain was not a major issue during their recovery. | How bad is the pain after weight loss surgery? | Pain following weight loss surgery, like any surgical procedure, varies widely depending on the individual and the specific type of surgery performed. The two most common types of weight loss surgery are laparoscopic adjustable gastric banding (LAGB) and gastric bypass.  Laparoscopic surgeries like LAGB are generally less invasive and may lead to less postoperative pain than more invasive procedures like gastric bypass. However, regardless of the specific procedure, most people will experience some degree of discomfort following surgery. This discomfort can range from mild to severe.  Immediately after the surgery, patients often feel soreness and pain around the incision sites. This can be managed with pain medication provided by the doctors. The pain typically starts to subside after a few days but can last for 1-2 weeks in some cases.  In addition to pain at the surgical site, some patients may also experience other uncomfortable symptoms such as nausea, vomiting, diarrhea, or constipation due to the changes made to their digestive system. These side effects usually subside as the body adjusts to the changes.  Keep in mind that your healthcare team will be able to provide you with appropriate pain management strategies. This often includes medication, rest, and slowly increasing physical activity.  Each person's experience is unique, so it's important to discuss these concerns with your healthcare provider before surgery. They can provide more specific information based on your health history and the details of the planned procedure. | 7/2/2023 | Sure, I can explain it in simpler terms.  When you get weight loss surgery, you may feel some pain afterwards. This is normal for any operation. The amount of pain depends on the person and the type of surgery.  If you have surgery that is done with small cuts, like a gastric band, you might have less pain than if you have a bigger surgery, like a gastric bypass.  Right after the surgery, you will probably feel sore around the area where the doctor made the cuts. This can be controlled with medicine from your doctor. This pain usually starts to get better after a few days, but for some people it can last 1-2 weeks.  You might also feel sick or have upset stomach, because your body is getting used to the changes from the surgery. These feelings usually go away over time.  Your doctor will help you manage any pain you feel. This might include taking medicine, resting, and slowly starting to move more.  Remember, everyone is different. It's important to talk to your doctor about what you might feel after surgery. They can give you more information based on your personal health. |
| 3 | When can I start exercising again after surgery? | As soon as patients totally wake up from anesthesia, we encourage patients to walk. The key is to start slow and listen to your body. If you lift weights or do sports, stay “low impact” for the first month (avoid competition, think participation). If you experience pain or discomfort, stop and relax. Avoid lifting heavy objects for the first 6 weeks. If you swim, your wounds need to be healed over before you get back in the water and always swim slowly at the beginning. | When can I start exercising again after weight loss surgery? | After bariatric surgery (weight loss surgery), it's generally recommended to start light activity as soon as you're able, which may be as soon as the day of or the day after surgery. This can include short walks and gentle stretching, which can help to speed recovery and decrease the risk of complications such as blood clots.  More intense exercise, such as strength training or cardio, is typically not recommended until a few weeks post-surgery, but this can vary greatly depending on the individual, the specifics of the procedure, and the surgeon's guidelines. Here's a general timeline, but remember, everyone's recovery is different and you should follow your healthcare team's advice:  1. First few days after surgery: Light walking and gentle stretching as tolerated.  2. 1-2 weeks post-surgery: Gradually increase the duration and intensity of your walking.  3. 2-4 weeks post-surgery: Low impact exercise, such as swimming or cycling, may be added.  4. 4-6 weeks post-surgery: Strength training can usually be gradually added in, starting with light weights and low intensity.  5. 6+ weeks post-surgery: Typically patients can return to their full range of pre-surgery activities, as long as they're comfortable and there are no complications.  Note that this is a rough guide. It's crucial to consult with your healthcare provider before starting any exercise regimen after surgery, as they'll be able to provide personalized advice based on your health history, the type of surgery you had, and your recovery progress. If at any point you feel pain, discomfort, or experience other worrisome symptoms during exercise, stop and notify your healthcare provider.  Finally, remember that exercise is only one part of a comprehensive post-surgery health plan. Nutrition, mental health support, and regular check-ups with your healthcare provider are equally important. | 7/3/2023 | Of course! Here is a simple explanation:  After weight loss surgery, you want to start moving as soon as you can. This might be the same day or the day after. Start with easy stuff like short walks or gentle stretches.  After a week or two, you can walk for longer times. About 2 to 4 weeks after surgery, you might start doing easy exercises like swimming or bike riding.  Around 4 to 6 weeks after surgery, you can start lifting light weights. After about 6 weeks, you can usually do most of the activities you did before the surgery.  But remember, everyone is different. This is just a general plan. You should talk to your doctor about what is right for you. And if anything hurts or feels wrong when you exercise, stop and tell your doctor.  Also, don't forget that eating healthy food, looking after your mental health, and seeing your doctor regularly are just as important as exercise after surgery. |
| 3 | Do most patients need to have plastic surgery after weight loss? | After losing most of the excess weight, most patients may have some loose or sagging skin.  For many patients, this might be just temporary. The degree of loose or sagging skin depends upon several things, including how much weight you lose, your age, your genetics and whether or not you exercise or smoke.  Generally, many patients can hide loose skin by clothing or compression garments.  We generally recommend wait for at least 18 months before having plastic surgery.  Plastic surgery to remove excess abdominal and breast skin is often covered by insurance because it may cause rash.  You may have to pay yourself for the areas such as arms because it is considered cosmetic by your insurer. | Do most patients need to have plastic surgery after weight loss following weight loss surgery? | It's not accurate to say "most" patients need plastic surgery following weight loss surgery because it depends on the individual's goals, body composition, skin elasticity, and the amount of weight lost. However, many patients do opt for plastic surgery after massive weight loss.  Bariatric surgery (weight loss surgery) often results in significant weight loss. This can lead to excess skin, which some individuals find undesirable or uncomfortable. For example, the skin can cause hygiene issues, skin infections, body image concerns, and even physical discomfort during day-to-day activities.  Body contouring surgeries, including abdominoplasty (tummy tuck), body lift, arm lift (brachioplasty), and others, can be performed to remove excess skin and improve the shape and tone of underlying tissue. The choice to undergo these surgeries is highly personal and often depends on how much the excess skin is affecting a person's quality of life and self-esteem.  Keep in mind that all surgical procedures carry risks, and it's important to have a comprehensive discussion with the healthcare provider about the benefits and risks before making a decision. It's also vital to remember that these surgeries should be considered only after weight loss has stabilized because further significant weight fluctuations can affect surgery results. | 7/3/2023 | Sure, I can make it simpler!  After weight loss surgery, many people lose a lot of weight. This can sometimes leave extra, loose skin on their bodies. Not everybody likes how this looks or feels. The loose skin can sometimes cause problems like infections or discomfort.  To help with this, some people choose to have plastic surgery. This kind of surgery can remove the extra skin and make the body's shape look smoother.  Whether someone wants to have plastic surgery after weight loss is a personal choice. It often depends on how much the extra skin bothers them.  It's important to remember that any kind of surgery can have risks. So, it's always good to talk to a doctor about the pros and cons before deciding. Also, these surgeries work best when a person's weight has stopped changing a lot. This is because losing or gaining more weight can change the surgery's results. |
| 3 | Will I lose hair after bariatric surgery? | For the reasons we still don’t fully understand, hair loss after bariatric surgery is common between 3 and 6 months following surgery. However, hair loss is almost always temporary. Adequate intake of protein, vitamins, biotin and minerals will help to ensure hair re-growth, and avoid longer term thinning. |  | Hair loss after bariatric surgery is a possible side effect for some people, but it's typically temporary. This can occur due to a few different factors:  1. Telogen effluvium: This is a form of temporary hair loss that can occur after major stress, high fever, weight loss, or surgery. During this phase, the hair moves faster than usual from its growing phase into the "resting" phase before moving quickly into the shedding, or telogen, phase.  2. Nutritional deficiency: Rapid weight loss, such as that which can occur after bariatric surgery, can lead to deficiencies in vital nutrients that are essential for hair health. These can include protein, iron, vitamin B12, folate, and zinc. Your body may redirect these nutrients away from less vital functions like hair growth to more important functions in a state of nutritional deficiency.  3. Changes in hormones: Bariatric surgery, particularly the Roux-en-Y gastric bypass, can lead to hormonal changes that can influence hair loss.  The good news is, this hair loss is generally temporary, and as your body adjusts to the changes in your diet, nutrient absorption, and weight, your hair should begin to regrow. It's important to follow the dietary and supplement guidelines provided by your healthcare team to ensure you're getting the nutrients you need.  Remember, if you are experiencing hair loss after bariatric surgery, or have concerns about it, it's best to discuss this with your healthcare provider. They can help identify if there's a particular deficiency or issue that needs to be addressed. | 7/3/2023 | Of course! After a big operation like weight loss surgery, some people might see their hair falling out more than usual. This can happen for a few reasons:  1. The stress from surgery can make hair fall out faster.  2. Because you're eating less food after surgery, your body might not get all the nutrients it usually gets. This can also make your hair fall out.  3. The surgery can change some of your body's hormone levels, which can affect your hair.  This kind of hair loss is usually temporary, meaning your hair should start growing back after your body gets used to the changes from the surgery. You should follow the diet and vitamins your doctor recommends to make sure your body gets what it needs.  If you notice your hair falling out a lot, you should talk to your doctor. They can figure out if something else is causing it. |
| 3 | Do I need to take vitamins and minerals after surgery? | You will need to take a multivitamin for life. You may need higher doses of certain vitamins or minerals, especially Iron, Calcium, and Vitamin D. You will also need to have at least yearly lab checks. Insurance almost never pays for vitamin and mineral supplements but usually does pay for labs. You can pay for supplements out of a flex medical account. | Do I need to take vitamins and minerals after weight loss surgery? | After bariatric (weight loss) surgery, the ability of your body to absorb certain vitamins and minerals may be significantly reduced. This is because the surgery changes the structure of your digestive system, altering the way your body processes food.  The common types of bariatric surgeries, including gastric bypass, sleeve gastrectomy, and adjustable gastric banding, all can affect nutrient absorption.  As such, many patients need to take vitamin and mineral supplements for the rest of their lives after surgery. The common vitamins and minerals that might be recommended include:  - Multivitamin: A daily multivitamin can provide many of the nutrients your body needs.  - Vitamin B12: Some types of weight loss surgery can decrease the body's ability to absorb vitamin B12, which is essential for blood and nerve function.  - Iron: Iron is needed to make new red blood cells. Some types of weight loss surgery can make it harder for the body to absorb iron.  - Calcium and Vitamin D: Both are important for bone health. Calcium absorption can be affected by weight loss surgery, especially procedures that bypass part of the small intestine.  - Vitamin A, D, E, K: These are fat-soluble vitamins. Absorption can be affected in surgeries that cause malabsorption.  The exact vitamins and dosage you'll need will depend on the type of surgery you've had, your diet, your health status, and other factors. Your doctor or dietitian will work with you to develop a plan that meets your needs.  Furthermore, regular follow-up and lab tests are necessary after bariatric surgery to ensure that you're getting the nutrients you need and to adjust supplementation as necessary.  Please note that while taking supplements can help prevent nutrient deficiencies, they're not a substitute for a healthy, balanced diet. It's still important to eat a variety of nutrient-dense foods as tolerated post-surgery.  Remember to always consult with your healthcare provider before starting any new supplement regimen, especially post-surgery. | 7/3/2023 | Sure, I can definitely simplify that.  After weight loss surgery, your body might have a hard time getting enough vitamins and minerals from food alone because the surgery changes the way your body handles food.  That's why doctors often tell patients to take vitamin and mineral supplements. These are like extra doses of the good stuff found in food that your body needs to work properly.  Here's a list of some you might need:  - Multivitamin: It's like a little package of many different vitamins your body needs.  - Vitamin B12: It's important for your nerves and blood.  - Iron: It helps your body make new red blood cells.  - Calcium and Vitamin D: They keep your bones strong.  - Vitamins A, D, E, K: They are needed for many body functions.  Remember, each person might need different vitamins and in different amounts, depending on their health and type of surgery. So, it's very important to talk to your doctor about which supplements you should take.  And, even though you're taking extra vitamins, eating healthy foods is still very important.  Lastly, your doctor will want to see you for check-ups often after your surgery. They might do blood tests to make sure your body is getting all the vitamins and minerals it needs.  Is this simpler explanation helpful? |
| 3 | If my insurance company will not pay for the surgery, can I pay the surgery myself? | We do offer bariatric surgery for patients who want to pay cash. Please call our office to get a quote. | If my insurance company will not pay for weight loss surgery, can I pay the surgery myself? | Yes, if your insurance company doesn't cover weight loss surgery, you can choose to pay for the procedure yourself, provided you have the financial means to do so. However, you should be aware that weight loss surgery can be quite expensive, potentially costing tens of thousands of dollars, depending on the specific procedure, the surgeon's fees, hospital charges, and other associated costs.  It's also important to consider the additional costs after surgery, such as follow-up care, diet and lifestyle changes, potential need for cosmetic surgery to remove excess skin after significant weight loss, and any complications that may arise from the surgery.  Additionally, be aware that in order to be a candidate for weight loss surgery, you usually need to meet certain medical criteria. A physician or surgeon specializing in bariatric procedures can provide you with more information on whether you might be a suitable candidate for the surgery.  Before deciding to pay out of pocket, it may be worthwhile to appeal the insurance company's decision or explore other insurance options. Some companies might cover the procedure if it is deemed medically necessary and other weight loss efforts have been unsuccessful. Check the terms of your insurance policy, and consider discussing your situation with a health care advocate or a legal professional. | 7/3/2023 | Sure, I can simplify that:  If your insurance won't pay for weight loss surgery, you can pay for it yourself if you have enough money. This surgery can cost a lot of money, though - sometimes many thousands of dollars. You'll also need to pay for things like seeing the doctor after surgery and other costs.  But remember, not everyone can have this surgery. Doctors have rules about who can get it, usually based on how healthy you are and how much you weigh.  If you can't afford to pay for the surgery yourself, you could try asking the insurance company again or looking at other insurance plans. Some might pay for the surgery if your doctor says you really need it and other ways to lose weight haven't worked. You might want to talk to a lawyer or someone who knows a lot about insurance to help you understand your options. |
| 3 | Will bariatric surgery last? | Many patients (up to 50%) may regain a small amount of weight (approximately 5 percent) two years or more following their surgery.  However, longitudinal studies find that most bariatric surgery patients maintain successful weight-loss long-term.  One study summarizing the results from 22 reports with a total of 4206 patient cases reported good long-term durability of gastric bypass surgery.  The average excess weight loss was 66.5%, and there was no significant association between excess weight loss and length of follow-up.  Now we have data to support the durability of gastric bypass surgery up to 15 years.  Studies also have shown that sleeve gastrectomy patients were able to maintain its well-documented weight loss outcome at 5 or more years after surgery, with the overall mean excess weight loss at 5 or more years still remaining>50%.  Such significant and sustained weight loss after surgery is in sharp contrast to medical management |  | Bariatric surgery, such as gastric bypass or gastric sleeve, can be a very effective tool for long-term weight loss in individuals who are severely obese or those who have obesity-related health conditions. However, the effectiveness of bariatric surgery isn't solely dependent on the surgical procedure itself, but also on the lifestyle changes that the patient makes after the surgery.  For the physical changes from the surgery to last, a patient will need to make significant dietary and exercise changes. This often involves working with a dietitian to develop a healthy eating plan, and gradually increasing physical activity levels. These lifestyle changes are vital to maintain the weight loss and prevent potential weight regain.  Moreover, some types of bariatric surgery, like the gastric band, might require periodic adjustments or maintenance. There is also a possibility of complications or side effects over time, and in some cases, revisional surgery might be necessary.  It's also important to note that while bariatric surgery can significantly reduce the risk of obesity-related conditions like heart disease, type 2 diabetes, certain types of cancer, and sleep apnea, it is not a guarantee of long-term health if the patient doesn't maintain a healthy lifestyle.  Bariatric surgery is a tool that can greatly assist with weight loss, but it is not a magic cure for obesity. Like any tool, its effectiveness will depend largely on how it is used. In other words, the lasting effects of bariatric surgery will depend on the patient's commitment to lifestyle changes after the procedure. | 7/3/2023 | Absolutely, I can explain it in simpler terms.  Bariatric surgery, like the surgeries that make your stomach smaller, can help people lose a lot of weight. But the surgery by itself is not enough to keep the weight off for good.  After the surgery, you also need to change the way you eat and how much you move. This means eating healthier foods, in smaller amounts, and getting more exercise. These changes help make sure the weight you lose from the surgery stays off.  Some types of this surgery might need check-ups or adjustments over time. And in rare cases, there might be problems later on that need another surgery to fix.  Even though the surgery can help with weight-related health problems, like heart disease or diabetes, it can't keep you healthy all on its own. You still need to eat right and exercise to stay healthy in the long run.  In short, the surgery can help a lot, but you also have to work hard to stay healthy and keep the weight off. |
| 4 | Do I qualify for weight loss surgery? | Patients will meet with a Mass General care team to evaluate their overall health and candidacy for bariatric surgery. Generally, patients must meet the following requirements to be eligible for weight loss surgery:  - Have a body mass index (BMI) of 35 or higher  - Have a BMI between 30 and 35 in conjunction with a weight-related comorbidity, such as heart disease, diabetes, or high blood pressure  - Have a stable psychological status | How do I qualify for weight loss surgery? | Qualifying for weight loss surgery, also known as bariatric surgery, generally involves meeting certain medical guidelines. Please note that these guidelines can vary slightly between different countries and health organizations, but they typically include the following:  1. Body Mass Index (BMI): This is usually the first criterion. In many cases, you might qualify for bariatric surgery if you have a BMI of 40 or higher (extreme obesity). If you have a BMI between 35 and 39.9 (obesity), you may also qualify if you have a serious weight-related health problem such as type 2 diabetes, high blood pressure, or severe sleep apnea.  2. Failed Weight Loss Attempts: Typically, you will need to demonstrate that you've made serious efforts to lose weight through diet and exercise, but haven't been successful.  3. Understanding the Risks and Commitment: You'll need to understand the risks and benefits of the surgery, as well as the lifestyle changes you'll have to make afterwards. This usually includes a commitment to diet, exercise, and possibly behavioral therapy.  4. Evaluation by Professionals: Most programs require an evaluation by a team of health professionals. This could include a dietitian, a psychologist or psychiatrist, and a physician to evaluate your physical and mental health.  5. Age: Some programs have age limits. While there's no strict upper limit, the risks of surgery often increase for older adults. Some programs may also perform bariatric surgery on adolescents, but this usually requires special medical and psychological evaluations.  6. Absence of Alcohol or Drug Abuse: You'll usually need to be free of drug and alcohol problems for at least a year before you can undergo surgery.  Again, these criteria can vary, and not all people who meet these criteria are necessarily suitable candidates for surgery. This decision is usually made in consultation with a medical professional. Additionally, some people may not meet these criteria but may still be considered for surgery in certain circumstances, depending on the judgment of their healthcare provider.  Ultimately, to know if you qualify for weight loss surgery, you would need to consult with a medical professional, usually a bariatric surgeon or a specialist in obesity medicine. | 7/3/2023 | Absolutely, I can simplify that. To qualify for weight loss surgery, usually:  1. You need to be very overweight. Doctors often use a measure called Body Mass Index (BMI). If your BMI is 40 or more, you might qualify. If your BMI is between 35 and 40, you could qualify if you also have serious health problems related to your weight, like diabetes or high blood pressure.  2. You have tried to lose weight in other ways, like diet and exercise, but it didn't work.  3. You understand what the surgery involves, including the risks and the changes you'll need to make to your lifestyle afterwards.  4. You are evaluated by a team of healthcare professionals to see if you are physically and mentally prepared for the surgery.  5. There are certain age limits. The surgery is riskier for older adults. In some cases, teenagers might have the surgery, but they need extra evaluations.  6. You should not have problems with alcohol or drugs.  Remember, just because you meet these points doesn't mean you will definitely have the surgery. You need to discuss this with your doctor. |
| 4 | Will insurance cover bariatric surgery? | Many insurance companies do cover weight loss surgery as long as you meet the qualification requirements. To learn more about insurance coverage, call our Weight Center staff at 617-726-4400. |  | Whether or not bariatric surgery is covered by insurance can vary greatly depending on the specifics of your individual health insurance plan. In many cases, insurance companies will cover weight-loss surgery, but only if you meet certain criteria.  Typically, you might need to demonstrate the following to be eligible for coverage:  1. A body mass index (BMI) over 40, or a BMI over 35 with serious weight-related health problems, such as type 2 diabetes, heart disease, or severe sleep apnea.  2. Evidence that you've unsuccessfully attempted to lose weight through non-surgical means, such as diet and exercise.  3. In some cases, a psychological evaluation may be necessary.  4. Some insurers may require a medically supervised weight loss program before approving the surgery.  However, every insurance plan is different. It's critical to directly contact your insurance provider or check your plan's policy regarding weight loss surgeries to understand what is covered and what isn't. Some plans may cover certain types of bariatric surgery but not others. Furthermore, even if the surgery itself is covered, certain related expenses might not be, such as dietary counseling or follow-up care.  In addition to checking with your insurance company, you should also discuss this with your healthcare provider. They can help you understand what might be involved in the process and can often provide assistance in working with your insurance company.  Remember that while I strive to provide accurate and up-to-date information, I should not replace professional medical advice or consultation with your insurance provider. Always discuss your personal circumstances and options with professionals in those fields. | 7/3/2023 | Of course!  Health insurance might pay for weight-loss surgery, but it depends on the rules of your insurance plan.  Usually, you need to show the following things to have your surgery covered:  1. You are very overweight, or you're pretty overweight but also have serious health problems like diabetes or heart disease.  2. You've tried to lose weight in other ways, like dieting and exercising, but it didn't work.  3. Sometimes, you need to talk to a mental health professional first.  4. Some insurance companies require you to follow a weight-loss plan supervised by a doctor before they approve surgery.  Each insurance plan has different rules. So it's really important that you call your insurance company or read your policy to understand what is covered.  Your doctor can also help explain things and may assist with your insurance company. Just remember, you should talk to your doctor or your insurance company about your personal situation to get the best advice. |
| 4 | Will I need plastic surgery after bariatric surgery? | Sudden weight loss can result is loose or sagging skin, either temporary or permanent. The severity of it depends upon several things, including the percentage of weight loss, genetics, and lifestyle habits (i.e., exercise, smoking, etc.). It’s often recommended to allow some time to pass before undergoing plastic surgery.  Board-certified plastic surgeons in the Mass General Body Contouring Program perform procedures to remove excess skin and reshape the body to its desired proportions following weight loss surgery. |  | Whether or not you'll need plastic surgery after bariatric surgery depends on a few factors. These include:  1. **The amount of weight you lose**: Bariatric surgery can result in significant weight loss. In some cases, this rapid and substantial loss can leave behind loose, excess skin, which some individuals choose to remove through plastic surgery.  2. **How your skin responds to weight loss**: Some people's skin naturally has more elasticity and may "bounce back" more than others' after significant weight loss. Younger people, for example, often have more elastic skin than older people.  3. **Personal preference**: Some people choose to get plastic surgery after bariatric surgery because they are dissatisfied with the appearance of their excess skin. Others, however, are fine with it and decide not to have further surgery.  4. **Health considerations**: Excess skin can sometimes lead to issues such as skin infections or discomfort during physical activity, which might make plastic surgery a beneficial option.  Typical post-bariatric plastic surgeries include tummy tucks (abdominoplasty), body lifts, breast lifts, arm lifts (brachioplasty), and thigh lifts.  Always consult with your healthcare provider or a plastic surgeon to discuss your specific situation and understand the risks and benefits of any procedure. | 7/3/2023 | Of course! After losing a lot of weight from bariatric surgery, some people have extra, loose skin. This can happen because the skin was stretched when the person was heavier, and it doesn't snap back into place when the person loses weight.  Some people are okay with having this extra skin. But others might feel uncomfortable or unhappy with how it looks. In some cases, the extra skin can cause health problems like skin infections.  To fix this, some people choose to get plastic surgery. This can involve removing the extra skin and tightening the remaining skin. This kind of surgery can be done on different parts of the body like the stomach, arms, or legs.  But whether someone should get plastic surgery after weight loss surgery really depends on how they feel and what their doctor thinks is best for their health. It's important to talk to a doctor about all the options. |
| 4 | How effective is bariatric surgery? | Bariatric surgery is considered to be the most effective treatment for obesity in terms of sustained weight loss and improvement in other obesity-related conditions. The amount of weight you lose depends on the type of bariatric surgery and lifestyle habits following surgery. It is important to keep all scheduled follow-up visits so that your care team can monitor your weight loss.  The benefits of bariatric surgery include:  - An improved quality of life  - A reduced risk of other obesity-related diseases such as cardiovascular disease, sleep apnea, and type 2 diabetes  - Improved blood pressure  - A longer lifespan |  | Bariatric surgery, including procedures like gastric bypass, sleeve gastrectomy, adjustable gastric band, and biliopancreatic diversion with duodenal switch, can be highly effective for long-term weight loss. However, it's important to understand that the success of these surgeries depends on multiple factors including the specific type of surgery, patient's commitment to lifestyle changes, and follow-up care.  Here are some key points based on information available up to my training cut-off in September 2021:  1. **Weight Loss**: Bariatric surgery can result in significant weight loss. On average, people lose around 15-30% of their original weight, but this can vary widely. For example, a study published in JAMA Surgery in 2018 reported a 77% median excess weight loss after 10 years for patients undergoing Roux-en-Y gastric bypass surgery.  2. **Comorbidity Improvement**: Bariatric surgery can also help improve or even resolve obesity-related comorbidities such as type 2 diabetes, hypertension, obstructive sleep apnea, and dyslipidemia. The improvement in diabetes control can sometimes occur even before significant weight loss occurs.  3. **Long-Term Success**: The long-term success of bariatric surgery depends heavily on an individual's commitment to lifestyle changes, including diet and exercise. Post-operative follow-ups, ongoing dietary counseling, and support group participation often play critical roles in maintaining weight loss and overall health improvements.  4. **Risks and Complications**: Like any surgical procedure, bariatric surgery comes with risks. Complications can include infection, bleeding, blood clots, and adverse reactions to anesthesia. There can also be longer-term risks such as nutritional deficiencies, gallstones, hernias, and mental health disorders. Some procedures, like the gastric band, may require adjustment or may not work over the long term and require additional surgeries.  5. **Mental Health**: There's increasing recognition of the role of mental health in obesity and weight loss surgery. Some individuals may struggle with adjusting to their new body, changes in relationships, and managing their eating behaviors after surgery. Mental health support before and after surgery can be an important part of care.  In conclusion, bariatric surgery can be very effective for both weight loss and improvement in obesity-related health conditions. However, it is not a magic bullet and requires a serious, lifelong commitment to lifestyle changes. As with any medical procedure, it's important to discuss the potential benefits, risks, and alternatives with a healthcare provider. | 7/3/2023 | Sure, I'd be happy to simplify!  1. **Weight Loss**: Bariatric surgery can help people lose a lot of weight. How much you lose can vary but it's usually between 15-30% of your original weight.  2. **Health Improvement**: The surgery can also help with health problems related to being overweight, like type 2 diabetes, high blood pressure, sleep problems, and high cholesterol.  3. **Long-Term Success**: To keep the weight off and stay healthy, you have to make lifestyle changes after the surgery. This includes eating a healthier diet, exercising, and going to follow-up appointments with your doctor.  4. **Risks**: Like any surgery, there are risks involved. These can include infection, bleeding, and reactions to anesthesia. There can also be longer-term problems like not getting enough nutrients, developing gallstones, or mental health issues.  5. **Mental Health**: It's important to have mental health support before and after surgery. Some people may struggle with adjusting to how they look and feel after they lose weight.  So, bariatric surgery can help a lot with weight loss and improving health problems related to being overweight, but it's not a quick fix. It involves making lifelong changes and comes with its own risks. Always talk to a doctor to understand what's best for you. |
| 4 | Is bariatric surgery safe? | While any surgical procedure has risks, bariatric surgery has been found to be one of the safest surgeries to undergo in comparison with other elective procedures. The Mass General Weight Center team performs a high volume of weight loss surgeries, which studies have shown relates to lower complication rates and improved outcomes related to quality and safety.  It is important to discuss any concerns you may have with your bariatric surgery care team. They will review and answer all your questions ahead of surgery. |  | As of my knowledge cutoff in September 2021, bariatric surgery was considered generally safe and effective for treating severe obesity, especially when combined with a comprehensive treatment plan. However, like any surgery, it comes with risks.  Most people who undergo bariatric surgery experience significant weight loss, leading to improvements in health conditions associated with obesity, like type 2 diabetes, high blood pressure, heart disease, sleep apnea, and certain types of cancer. Many patients experience improved mobility, mood, and quality of life.  Despite these benefits, bariatric surgery can also have risks and side effects, such as:  1. Complications related to anesthesia and surgery: These can include infections, bleeding, blood clots in the legs or lungs, and leaks in the gastrointestinal system.  2. Nutritional deficiencies: Because bariatric surgery affects the way the body absorbs nutrients, it may result in deficiencies, which may require lifelong supplementation with vitamins and minerals.  3. Mental health conditions: Changes in body image, diet, and lifestyle can lead to new or worsened symptoms of depression, anxiety, and other mental health conditions.  4. Dumping syndrome: This is a condition in which food moves too quickly from the stomach to the small intestine, causing symptoms like nausea, vomiting, bloating, diarrhea, dizziness, and sweating.  5. Long-term weight regain: Some people may not lose as much weight as they hoped or may regain weight over time.  Before considering bariatric surgery, patients should talk to their healthcare provider about their individual risks and benefits. They should also commit to lifelong healthy eating and physical activity habits, regular medical follow-up, and possibly mental health counseling to help maintain the benefits of surgery. Keep in mind that new research may have emerged after my last training data in September 2021, so it's always a good idea to check the most recent guidelines and studies. | 7/3/2023 | Sure, I'll explain it in simpler terms!  Bariatric surgery, a type of surgery that helps you lose weight, is usually safe and can help people a lot. It's often used when diet and exercise aren't enough for someone who is very overweight.  After the surgery, people usually lose a lot of weight. This can make them healthier and reduce problems like heart disease, diabetes, and sleep issues. People often feel better and find it easier to move around.  However, like any surgery, it's not without risks:  1. There might be problems during or after surgery: These can include infection, bleeding, or issues with the digestive system.  2. It could cause problems with nutrition: Because the surgery changes how the body takes in food, it can lead to a lack of certain vitamins and minerals. This means that people might have to take supplements for the rest of their lives.  3. It might affect mental health: The changes in someone's body and life after surgery can sometimes cause feelings of depression or anxiety.  4. It can cause a condition called "dumping syndrome": This happens when food moves too quickly from the stomach to the intestines and can cause unpleasant symptoms like feeling sick or weak, or having diarrhea.  5. Weight might come back over time: Some people might not lose as much weight as they hoped, or they might gain some of it back after a while.  Before deciding to have this surgery, it's important to talk to a doctor about the possible risks and benefits. It's also important to be ready to eat healthily and exercise regularly for the rest of one's life after the surgery. |
| 4 | When can I expect to return to daily life after weight loss surgery? | Patients generally are able to stand and walk a few hours after surgery and are discharged within one to two days. Avoiding high impact exercise and heavy weight lifting is recommended for at least one month, with an emphasis on listening to your body and resting as needed. Many patients return to work about four weeks after surgery.  Because of a lower calorie intake, many patients feel a lower energy than usual for a couple of weeks after surgery. Energy tends to elevate after transitioning from a liquid diet to solid foods. |  | After bariatric surgery (weight loss surgery), the time it takes to return to "normal" activities can vary depending on the specific type of surgery, your overall health status, and your individual recovery rate.  Generally, you can expect the following:  - Hospital stay: Depending on the procedure, you may stay in the hospital for anywhere from 1-4 days.  - Return to work: For desk jobs or non-physically demanding roles, you may be able to return to work after 1-3 weeks. If your job involves heavy lifting or other physical activities, you may need to wait 4-6 weeks or more.  - Physical activities: Light, low-impact physical activities can usually be resumed in 1-3 weeks after surgery. More strenuous activities, like heavy lifting or intense workouts, may need to be avoided for up to 6 weeks or until your surgeon clears you.  - Diet: Immediately after surgery, you'll likely be on a liquid diet for a few weeks, which gradually progresses to pureed foods, then to soft foods, and finally to a regular diet after about 2-3 months.  These timelines are rough estimates, and individual experiences can vary widely. It's crucial to follow the specific advice given by your healthcare team to ensure a safe and effective recovery.  Furthermore, it's important to note that "returning to daily life" after weight loss surgery doesn't mean returning to old habits. Permanent lifestyle changes regarding diet and exercise are critical to maintaining the weight loss achieved through the surgery.  As always, it's best to consult with your healthcare provider for the most accurate information based on your specific circumstances. | 7/3/2023 | Sure, I can simplify that:  After weight loss surgery:  - You might stay in the hospital for 1 to 4 days.  - If your job is not too physical, like an office job, you could go back to work in 1 to 3 weeks.  - If your job is physical, you might need to wait 4 to 6 weeks before you can go back to work.  - You can start doing light exercises like walking after 1 to 3 weeks.  - For tougher exercises, you need to wait about 6 weeks.  - You'll start with a liquid diet just after surgery, then slowly start eating more solid food over 2 to 3 months.  Remember, after weight loss surgery, you need to make lasting changes to what you eat and how much you exercise to keep the weight off. And always listen to your doctor's advice. |
| 5 | What are the risks of the operation? | Weight loss surgery is major abdominal surgery with significant risks. However, we know experience counts and high-volume centers like X have an extremely low complications rate.  The following potential complications include, but are not limited to:  - Risk from general anesthesia  - Bleeding  - Infection  - Pneumonia  - Blood clot in the legs or lungs  - Hernias (internally or externally)  - Blockage or obstruction  - Narrowing where surgeons suture the pouch to the intestine, which can require another endoscopy and dilation  - Leakage from the stomach or the intestines  - Injury to liver and/or spleen  - Pulmonary and/or cardiac and/or renal failure  With the adjustable gastric band (lap-band), there's a risk that the band can either slip or erode. There's a risk that you will not lose weight if you don't follow the diet and exercise program. There's a risk of needing another operation or conversion to an open operation with a long incision. While there's a risk of death with each procedure: 1-in-200 with the gastric bypass, 1-in-500 with sleeve gastrectomy, and 1-in-10,000 with the lap band. | What are the risks of weight loss surgery? | Weight loss surgeries, also known as bariatric surgeries, can be highly effective for significant and sustained weight loss, especially for individuals with severe obesity. However, like all surgical procedures, they are not without risks. Potential complications and risks can be immediate (short-term) or long-term.  Immediate risks may include:  1. **Infection:** As with any surgery, there's a risk of infection, especially at the site of the incision.  2. **Bleeding:** Excessive bleeding may occur during or after the surgery.  3. **Reactions to anesthesia:** Some people may have adverse reactions to anesthesia used during surgery, which can range from mild nausea to severe allergic reactions.  4. **Blood clots:** The risk of developing blood clots in the legs or lungs (deep vein thrombosis or pulmonary embolism) is increased after any surgery.  5. **Leaks in the gastrointestinal system:** This could occur where the surgeon has made a new connection in the digestive system.  6. **Breathing or heart problems:** These can occur due to the strain on the heart and lungs from the surgery and anesthesia.  Long-term risks and complications may include:  1. **Malnutrition and vitamin deficiencies:** Due to changes in the digestive system that may reduce the absorption of essential nutrients. This can lead to problems such as anemia, osteoporosis, and others. Regular follow-ups and supplementation are usually necessary.  2. **Dumping syndrome:** This occurs when food moves too quickly from the stomach into the small intestine, causing nausea, vomiting, diarrhea, and other symptoms.  3. **Gallstones:** Rapid weight loss can lead to the formation of gallstones.  4. **Hernias:** A hernia is a bulge of tissue or an organ through an abnormal opening, and these may occur more commonly after weight loss surgery.  5. **Low blood sugar (hypoglycemia):** This can occur in some people after weight loss surgery, especially gastric bypass surgery.  6. **Stomal stenosis or ulcers:** These are complications related to the new connections made in the digestive system during surgery.  7. **Mental health disorders:** Some people experience new or worsened symptoms of depression, anxiety, or other mental health conditions after surgery.  8. **Weight regain:** Some patients may regain some or all of the weight they lost after surgery, especially if they don't maintain healthy lifestyle changes.  Keep in mind that while these risks exist, they are generally low and have to be weighed against the benefits and the risks of severe obesity, which also includes serious health problems. Therefore, the decision to have weight loss surgery should be made in consultation with a doctor or a team of healthcare professionals, considering an individual's overall health, the severity of their obesity, and their ability to maintain lifestyle changes after surgery. | 7/3/2023 | Sure, I can simplify that information.  Weight loss surgery helps people lose a lot of weight, but it has some risks too.  Right after the surgery, people might have:  1. **Infection:** Germs can get into the cut the doctor made during the surgery and make people sick.  2. **Bleeding:** Sometimes, people can bleed too much during or after the surgery.  3. **Bad reactions to sleep medicine:** The medicine to make people sleep during surgery can make some people feel sick.  4. **Blood clots:** Sometimes, blood can get thick and stick together, blocking blood vessels. This can be dangerous.  5. **Leaks in the gut:** The doctor makes changes in the stomach or intestines during surgery, and sometimes these changes can leak.  6. **Breathing or heart problems:** The surgery can be hard on the heart and lungs.  Later on, people might have:  1. **Not enough vitamins:** The surgery changes how the body takes in food, so sometimes people don't get enough vitamins from what they eat.  2. **Sickness from eating:** Sometimes, food goes into the intestines too quickly, which can make people feel sick.  3. **Gallstones:** When people lose weight quickly, hard stones can form in a small organ called the gallbladder.  4. **Hernias:** A part of the body might poke through a weak spot in the muscles.  5. **Low blood sugar:** Sometimes, people's blood sugar can get too low after this surgery.  6. **Ulcers:** Sores can form in the stomach or intestines.  7. **Feeling sad or anxious:** Some people feel worse mentally after surgery.  8. **Gaining weight back:** Sometimes, people gain back the weight they lost.  Even though these risks can sound scary, they usually don't happen and the surgery can really help people who are very overweight. It's important to talk to the doctor about these risks and how to keep healthy after surgery. |
| 5 | How successful are these operations? | The gastric bypass operation allows you to lose up to 82% of your excess body weight, however it's almost 100% effective in reversing obesity related co-morbidities including sleep apnea, hypertension and diabetes. The adjustable gastric banding (lap-band) operation allows you to lose 50% of your excess body weight at two years. It's also effective in reversing obesity related co-morbidities. The sleeve gastrectomy is between the gastric bypass and gastric banding in terms of weight loss with 60% excess weight loss at 1 year. | How successful are the various weight loss surgeries? | Weight loss surgeries, also known as bariatric surgeries, can be quite successful for many people when paired with a commitment to lifestyle changes, like a healthy diet and regular physical activity. However, the success rates can vary significantly depending on the type of procedure, the individual's health, and their commitment to maintaining the lifestyle changes necessary for long-term weight loss.  Here are some general outcomes of the most common types of weight loss surgeries as of my knowledge cutoff in September 2021:  1. **Roux-en-Y Gastric Bypass (RYGB)**: This surgery is considered the gold standard for weight loss surgeries. It has been shown to result in significant long-term weight loss, about 60-80% of excess weight. Beyond weight loss, it can also lead to improvement or resolution of type 2 diabetes, hypertension, and other obesity-related conditions in many patients.  2. **Adjustable Gastric Banding (AGB)**: This type of surgery results in slower and less overall weight loss compared to others, typically about 40-50% of excess weight. The rate of reoperation is also relatively high due to band slippage, erosion, or other complications. However, it is less invasive and has a lower rate of serious complications compared to other surgeries.  3. **Vertical Sleeve Gastrectomy (VSG)**: Sleeve gastrectomy results in weight loss somewhere between that of RYGB and AGB, typically around 60-70% of excess weight. It has a relatively low complication rate, similar to that of AGB, but has better long-term weight loss and improvement in obesity-related conditions.  4. **Biliopancreatic Diversion with Duodenal Switch (BPD/DS)**: This is less commonly performed because it is more complex and carries a higher risk of complications. However, it results in the greatest weight loss, typically around 70-90% of excess weight, and the most significant improvement in conditions like diabetes.  Remember that the success of these surgeries also largely depends on a person's ability to maintain lifestyle changes post-surgery. Complications can occur, and there is a risk of regaining weight over time. It's also important to consider that newer procedures may be developed, and current procedures may be improved over time.  Remember that each individual is different, so the choice of which (if any) weight loss surgery to undergo should be made in consultation with a knowledgeable healthcare provider. They can provide more specific information tailored to a person's health status, goals, and concerns. | 7/3/2023 | Absolutely, I'll make it simpler.  Weight loss surgeries, also known as bariatric surgeries, can help people lose a lot of weight. But, how much weight people lose can depend on the type of surgery and how well they stick to a healthy lifestyle afterward.  Here's a breakdown of the main types of surgeries:  1. **Roux-en-Y Gastric Bypass (RYGB)**: This is a very effective surgery. Most people lose a lot of weight, and it can also help improve health problems like diabetes and high blood pressure.  2. **Adjustable Gastric Banding (AGB)**: This surgery doesn't help people lose as much weight as others, and sometimes it needs to be fixed or redone. However, it's less risky than some other types of surgery.  3. **Vertical Sleeve Gastrectomy (VSG)**: This surgery helps people lose a moderate amount of weight. It's not as risky as some surgeries, and it helps improve health problems like diabetes.  4. **Biliopancreatic Diversion with Duodenal Switch (BPD/DS)**: This surgery helps people lose the most weight, but it's the riskiest. It also helps a lot with health problems like diabetes.  Remember, no matter which surgery someone gets, they still need to eat healthy and exercise to keep the weight off. They should also talk to their doctor to decide which surgery, if any, is best for them. |
| 5 | Why is there a 10% pre-operative weight loss required? | We require patients to begin a diet and exercise program that allows them to lose 10% of their weight pre-operatively for three reasons:  1) Patients who undergo weight loss before surgery have fewer complications during and after surgery.  2) This allows patients to truly understand the lifestyle changes that will occur after surgery and therefore make an informed consent about the diet and exercise programs that they will have to be on for the rest of their lives.  3) This allows us to identify the patients who are truly motivated and therefore who will be most successful following surgery. Most important, we'll work with you to develop a weight-loss program to achieve the 10% reduction goal. | Why is there a 10% pre-operative weight loss required for weight loss surgery? | Preoperative weight loss is often required before bariatric surgery (such as gastric bypass or gastric sleeve procedures) for several reasons:  1. **Safety**: Reducing the size of the liver and visceral fat can decrease the risk of complications during surgery. Weight loss can reduce the size of the liver and the amount of fat around the organs, making it safer and easier for the surgeon to perform the procedure.  2. **Habit change**: Demonstrating the ability to lose weight prior to surgery may show that the individual is capable of making the necessary dietary and lifestyle changes after the surgery. This is crucial because bariatric surgery is not a standalone solution. It needs to be coupled with healthy lifestyle changes to ensure long-term success.  3. **Medical stabilization**: Weight loss prior to surgery can help stabilize comorbid conditions like diabetes or hypertension, reducing surgical risk.  4. **Postoperative outcomes**: Several studies suggest that patients who lose weight before surgery have better outcomes after surgery, including more significant postoperative weight loss.  It's important to note that the exact requirement for preoperative weight loss can vary. While you mentioned a 10% weight loss, the requirement might be different depending on the specific situation, the type of surgery, and the policy of the medical team or insurance company. As always, patients should consult with their healthcare providers to understand what's best in their individual situation. | 7/3/2023 | Absolutely, let's break it down:  1. **Makes the surgery safer**: Losing weight can shrink the size of the liver and the amount of fat around your organs. This can make the operation easier for the surgeon and reduce risks.  2. **Practice for new habits**: If you can lose weight before the surgery, it shows you can stick to a healthy diet and lifestyle. These habits are really important to keep the weight off after the surgery.  3. **Helps your health**: Losing weight can help improve other health conditions you may have, like diabetes or high blood pressure. This can make the surgery safer.  4. **Better results after surgery**: People who lose weight before surgery often lose more weight after the operation.  Remember, the amount of weight you need to lose before surgery can change depending on many things, like your personal situation, the type of surgery, and your insurance company's rules. It's always best to talk to your doctor to understand what's best for you. |
| 5 | What is the conversion rate? | Conversion to open surgery is required in less than 1% of patients. The risk of conversion is higher in patients who have had previous upper abdominal surgery. | What is the conversion rate from laparoscopic to open, with regards to weight loss surgery? | As of my training cutoff in September 2021, the conversion rate from laparoscopic to open surgery varies considerably depending on the procedure, the patient's specific conditions, and the surgeon's experience. For weight loss surgeries such as gastric bypass or sleeve gastrectomy, studies suggest a conversion rate generally between 2% to 10%. However, these are average figures, and the actual conversion rate can be significantly higher or lower depending on the factors mentioned above.  Keep in mind that surgical techniques, equipment, and practices continue to evolve, and more recent data may reflect different conversion rates. Furthermore, the decision to convert from laparoscopic to open surgery is based on the surgeon's judgement, prioritizing patient safety and the likelihood of achieving the surgical objectives. It's crucial to consult with the surgical team to understand the risks and benefits associated with any surgical procedure.  Please consult a recent, reliable source or a healthcare professional for the most accurate and up-to-date information. | 7/3/2023 | Sure, I'll try to explain it more simply.  If a doctor starts a weight loss surgery using a method called "laparoscopic" (which is a less invasive method where they use small cuts instead of a big one), sometimes they might need to switch to the "open" method (which involves a larger cut). The chance that they need to make this switch is usually between 2% and 10% for weight loss surgeries, based on information up to 2021. But, this can change based on things like the type of surgery, the patient's health, and the doctor's experience.  This information might be out of date though, because surgical techniques and tools continue to get better over time. So, the rate might be different now. It's important to talk to the doctor about the risks and benefits of the surgery to understand it better. |
| 5 | How do I know which operation is for me? | Deciding which procedure is best for you is largely a personal one best achieved in discussion with your surgeon. | How do I know which type of weight loss surgery operation is for me? | Choosing the appropriate type of weight loss surgery is a decision you should make in consultation with your healthcare provider. Different types of surgery may be more suitable for different individuals, depending on their overall health, the severity of their obesity, and their personal goals. Here are some factors you'll need to consider:  1. **Your BMI:** Many weight loss procedures have a minimum BMI requirement. For instance, in general, gastric bypass and sleeve gastrectomy are often recommended for individuals with a BMI over 40 or over 35 if they have related health conditions.  2. **Your weight loss goals:** Some procedures typically result in more weight loss than others. Gastric bypass tends to result in the most weight loss, followed by sleeve gastrectomy, and then gastric banding.  3. **Existing health conditions:** If you have severe acid reflux, a gastric bypass might be a better option than a sleeve gastrectomy. If you have a condition that might make it difficult for you to absorb nutrients, such as Crohn's disease, a malabsorptive procedure like gastric bypass might not be the best choice.  4. **Your commitment to lifestyle changes:** All weight loss surgeries require significant lifestyle changes, including changes to diet and physical activity. If you don't feel you can commit to these changes, surgery might not be the right choice.  5. **Your tolerance for risk and side effects:** Each type of surgery comes with its own risks and potential side effects. Gastric banding is less invasive and typically has fewer short-term risks and complications than gastric bypass or sleeve gastrectomy, but it tends to result in less weight loss and more long-term complications.  6. **The surgeon's experience:** The surgeon's skill and experience with a particular procedure can greatly influence the risk of complications and the success of the surgery.  In addition to considering these factors, you should discuss your medical history, lifestyle, and weight loss goals with your healthcare provider. They can help guide you toward the procedure that will be the most beneficial for you.  Remember, weight loss surgery is typically only considered for individuals who are severely obese and have not been able to lose weight or maintain weight loss with diet and exercise alone. It's a significant decision and should be made in consultation with healthcare professionals. | 7/3/2023 | Absolutely, here's a simpler explanation:  1. **Your weight:** These surgeries are usually for people who are very overweight. The more overweight you are, the more likely it is that one of these surgeries might help you.  2. **Your health:** Some surgeries might not be good for you if you have certain health problems. For example, if you often get heartburn, one type of surgery might be better for you than another.  3. **Your goals:** Some surgeries can help you lose more weight than others. So, if you want to lose a lot of weight, you might choose a different surgery than if you only want to lose a little.  4. **Lifestyle changes:** After the surgery, you will need to eat differently and exercise. If you don't think you can make these changes, the surgery might not be a good idea.  5. **Risks and side effects:** All surgeries can have problems or side effects. Some surgeries might have more problems or side effects than others.  6. **Surgeon's experience:** The doctor who does your surgery should be very good at it. If the doctor has done the surgery many times, it might work better.  To decide which surgery is best for you, talk to your doctor. They can help you think about what you want and what is safe for you. It's a big decision, so you should take your time and get all the information you need. |
| 5 | What will happen when I’m in the hospital? | In general, you'll be in the hospital for two nights. We'll expect you to get up and walk while you're in the hospital – this is your best defense against blood clots. You'll meet with the dietician before going home and while you're here, you'll have a clear liquid diet that will be limited to a medicine cup every 15 minutes.  Once home, you can drink until full. Please walk, drink once every hour, and take in at least 40 - 60 grams of protein daily for women, and 60 - 80 grams of protein daily for men | What will happen when I’m in the hospital after weight loss surgery? | After weight loss surgery, such as gastric bypass or sleeve gastrectomy, you will typically stay in the hospital for a few days, depending on the type of procedure and your health condition. Here's a general idea of what to expect during your hospital stay:  1. **Recovery from Anesthesia:** Immediately after the surgery, you will be taken to a recovery area where medical staff will closely monitor you as you wake up from the anesthesia.  2. **Pain Management:** You will likely experience some discomfort and pain due to the surgery. Nurses and doctors will help manage this pain using medications.  3. **Starting Fluids:** Initially, your stomach will need to heal, so you will be put on a liquid diet. This usually includes water, broth, and sugar-free popsicles or gelatin.  4. **Physical Movement:** Early mobilization is important. Nurses will encourage you to get out of bed and walk around as soon as possible to help prevent complications like blood clots.  5. **Monitoring:** Doctors and nurses will monitor your vital signs (like heart rate, blood pressure, and temperature), and check your incision sites for any signs of infection.  6. **Nutrition and Diet Education:** A dietitian will meet with you to explain the dietary changes you'll need to make after surgery. They will provide guidance on transitioning from a liquid diet to pureed food, and then gradually to more solid food.  7. **Breathing Exercises:** You might be asked to perform some breathing exercises or use a device called an incentive spirometer. This helps keep your lungs clear and can prevent pneumonia.  8. **Support and Counseling:** Many hospitals offer support and counseling for patients who have had weight loss surgery. This can help you cope with the emotional changes that often accompany significant weight loss.  Remember, it's important to follow your healthcare provider's instructions after surgery. If you have any questions or concerns, don't hesitate to ask. | 7/3/2023 | Absolutely, I can explain that in simpler terms. Here's what happens after weight loss surgery when you're in the hospital:  1. **Waking Up:** After surgery, you'll be in a special room where doctors watch you as you wake up from the sleep medicine.  2. **Pain Relief:** You might feel some pain from the surgery, but the nurses will give you medicine to help with this.  3. **Drinking Liquids:** You won't be able to eat normal food at first. You'll start by having only liquids like water or soup.  4. **Walking Around:** Even if you might feel a bit sore, it's good to start walking as soon as you can. This helps your body recover faster.  5. **Checking On You:** Doctors and nurses will keep checking on you to make sure you're okay. They'll look at things like your heartbeat, temperature, and the place where they did the surgery.  6. **Learning About Food:** A special food expert will teach you about what you can eat after your surgery. You'll start with liquids, then move to mushy foods, and slowly start eating solid foods.  7. **Breathing Exercises:** You might do some special exercises to help your lungs stay healthy.  8. **Talks and Counseling:** You might have some talks with a counselor to help you understand the changes to your body and how you feel.  Always listen to your doctors and nurses, and if you're not sure about something, just ask! |
| 5 | Is there an age restriction for the surgery? | Surgery is done on patients between the ages of 18 - 65. If you're younger than 18, please refer to our Adolescent Bariatric Surgery Program at Lucile Packard Children's Hospital. If you are older than 65, cases are evaluated on a case by case basis. Please keep in mind your insurance may restrict your options. | Is there an age restriction for weight loss surgery? | Yes, there are age restrictions for weight loss surgery, which are set in place due to safety and ethical reasons. As of my last training cut-off in September 2021, these are the general guidelines:  1. Adults: Most programs only offer bariatric surgery to adults, typically those aged 18 to 65 years old. However, the specific age limit can vary depending on the health status and overall condition of the patient. Some older adults may be candidates for the procedure if their overall health status is good.  2. Adolescents: While it's more uncommon, there are programs that offer weight loss surgery to adolescents. These cases are typically reserved for those who are severely obese and have co-morbid conditions (like type 2 diabetes, sleep apnea, or heart disease) that would benefit significantly from weight loss. The exact age can vary, but typically patients need to be at least 13 to 16 years old for girls and 15 to 19 years old for boys, as they should have achieved near-complete physiological and skeletal growth.  3. Children: As of 2021, bariatric surgery is generally not recommended for pre-adolescent children.  In all cases, potential candidates for weight loss surgery must meet certain criteria beyond age. These often include a Body Mass Index (BMI) over 40, or over 35 with serious weight-related health problems; history of failed attempts at weight loss through diet and exercise; and psychological readiness to make significant lifestyle changes.  Keep in mind that medical guidelines and standards of practice may have changed since my last update in 2021, and the standards can also vary by country and specific medical facility. Always consult with a healthcare provider for the most accurate and up-to-date information. | 7/3/2023 | Sure, I'll break it down more simply.  Weight loss surgery, also known as bariatric surgery, is a serious procedure. It's generally done on:  1. Adults: People aged between 18 to 65 years old, depending on their health.  2. Teenagers: Some teenagers may have the surgery if they are very overweight and it's seriously affecting their health. They should be at least 13 to 19 years old, depending on whether they are a boy or girl.  3. Younger children: Usually, doctors do not recommend this surgery for young kids.  No matter the age, the person must be very overweight and must have tried losing weight in other ways like diet and exercise. They also need to be ready to make big changes to their lifestyle after the surgery.  Remember, the doctor is the best person to decide if someone can have weight loss surgery. The rules can also change over time and may be different in other countries or hospitals. |
| 5 | Is there a weight limit for this operation? | We look at Body Mass Index (BMI), which is the weight over the body surface area squared. Patients who have a BMI of 35-40 and have obesity related co-morbidities are candidates for this surgery. Patients who have a BMI greater than 40, even if they don't have co-morbidities, are also candidates for this operation. | Is there a weight limit for weight loss surgery? | Yes, there are specific weight criteria for weight loss surgery, also known as bariatric surgery. As of my last training cut-off in September 2021, these criteria are typically defined in terms of Body Mass Index (BMI).  According to the National Institutes of Health (NIH), weight loss surgery might be an option for you if:  1. Your BMI is 40 or higher (extreme obesity).  2. Your BMI is 35 to 39.9 (obesity), and you have a serious weight-related health problem, such as type 2 diabetes, high blood pressure, or severe sleep apnea.  Some types of weight loss surgeries have a lower BMI requirement. For example, the U.S. Food and Drug Administration (FDA) has approved use of the adjustable gastric band (for example, Lap-Band) for people in the BMI range of 30 to 40, who also have at least one serious health problem linked to obesity.  In some cases, a weight loss surgery might be considered for someone with a BMI between 30 and 34 who has serious weight-related health problems.  However, bariatric surgery isn't for everyone who is severely overweight. You may need to meet certain medical guidelines and have a psychological evaluation to ensure readiness for the significant lifestyle changes necessary after surgery. Bariatric surgery should be used as a last resort when efforts to exercise and following guided diet plans have proven insufficient. It's always recommended to consult with your healthcare provider to discuss the risks and benefits associated with bariatric surgery, and any updates or changes in these guidelines post-2021. | 7/3/2023 | Sure, I'd be happy to simplify it for you.  Weight loss surgery, also known as bariatric surgery, is a procedure that's usually considered for people who are very overweight. Doctors use a measure called Body Mass Index (BMI) to decide who might benefit from this surgery.  BMI is a measure that uses both your weight and your height to come up with a score.  Here's when doctors might consider weight loss surgery:  - If your BMI is 40 or more. This means you're very overweight.  - If your BMI is between 35 and 40, and you also have a health problem like diabetes, high blood pressure, or severe sleep apnea (a condition where your breathing stops and starts while you sleep).  Some types of weight loss surgery can be done if your BMI is between 30 and 40, and you have a health problem related to being overweight.  But, weight loss surgery isn't for everyone who's overweight. You need to be ready to make big lifestyle changes after the surgery, like changing your diet and exercising. If you're considering weight loss surgery, you should talk to your doctor to understand all the pros and cons. |
| 5 | What are obesity related comorbidities? | These include diabetes, hypertension, arthritis, degenerative joint disease, sleep apnea, low pack pain, gastroesophageal reflux disease, stress incontinence, high cholesterol and triglyceride levels and depression. |  | Obesity is associated with a wide range of health conditions, often referred to as comorbidities. Here are some of the most common obesity-related comorbidities:  1. **Type 2 Diabetes**: Obesity is a significant risk factor for developing type 2 diabetes. The excess fat makes the body less sensitive to insulin, leading to insulin resistance, which can eventually cause type 2 diabetes.  2. **Heart Disease and Stroke**: Obesity can contribute to buildup of plaque in the arteries, leading to coronary artery disease, heart attack, and stroke.  3. **Hypertension (High Blood Pressure)**: Obesity may cause high blood pressure, a risk factor for heart disease and stroke.  4. **Dyslipidemia (Abnormal Levels of Lipids)**: This includes high levels of low-density lipoprotein (LDL) cholesterol ("bad" cholesterol), low levels of high-density lipoprotein (HDL) cholesterol ("good" cholesterol), and high levels of triglycerides.  5. **Non-alcoholic Fatty Liver Disease (NAFLD)**: This is a condition where fat builds up in the liver, leading to inflammation and potential damage to the organ.  6. **Cancer**: Obesity increases the risk of several types of cancer, including breast, colon, endometrial, kidney, and esophageal cancers.  7. **Osteoarthritis**: The extra weight can put more pressure on your joints and cartilage, leading to osteoarthritis.  8. **Sleep Apnea and Respiratory Problems**: Obesity, particularly central obesity (excess fat around the abdomen), can cause sleep apnea and other respiratory problems.  9. **Gastroesophageal Reflux Disease (GERD)**: Obesity increases the pressure inside the abdomen, causing stomach acid to flow back into the esophagus, leading to GERD.  10. **Gallstones**: Obesity increases the risk of gallstones, which are hard particles that form in the gallbladder.  11. **Depression**: There's also a link between obesity and depression, though the nature of the connection isn't fully understood. It could be related to body image, physiological changes, or other factors.  12. **Infertility and Complications in Pregnancy**: Obesity can cause hormonal changes that interfere with ovulation and can lead to infertility. It can also lead to complications during pregnancy.  These are just a few examples. It's worth noting that while obesity significantly increases the risk of these conditions, it's just one of many risk factors. Lifestyle changes or medical interventions can reduce these risks, even in the presence of obesity. | 7/3/2023 | Sure, I'd be happy to explain it in simpler terms.  Being very overweight, which doctors often call "obese," can lead to a lot of health problems. Here are some examples:  1. **Type 2 Diabetes**: This is a disease that makes it hard for your body to use sugar properly. It can be caused by being overweight.  2. **Heart Disease and Stroke**: These are conditions where the blood vessels to the heart or brain get blocked. Being overweight can lead to these problems.  3. **High Blood Pressure**: This means the pressure of blood in your vessels is too high. It can cause heart disease and is often seen in people who are overweight.  4. **Bad Cholesterol and High Triglycerides**: These are types of fat in your blood. If they're too high, they can cause heart disease. Being overweight can make these levels too high.  5. **Liver Disease**: Sometimes, too much fat can build up in your liver and cause it to get sick. This is more common in people who are overweight.  6. **Cancer**: Being overweight can increase your risk of getting certain types of cancer.  7. **Osteoarthritis**: This is a disease where your joints (like knees and hips) start to hurt and become damaged. It can be caused by the extra weight that comes with being overweight.  8. **Sleep Apnea**: This is a condition where you stop breathing for short periods while you sleep. It can be caused by being overweight.  9. **Acid Reflux**: This happens when acid from your stomach comes up into your throat. It can be caused by being overweight.  10. **Gallstones**: These are little stones that form in your gallbladder, an organ that helps digest fat. They can be caused by being overweight.  11. **Depression**: This is a condition that affects your mood and can make you feel very sad or hopeless. It's more common in people who are overweight, but we're not sure why.  12. **Problems Having a Baby**: Being overweight can make it harder to get pregnant. It can also lead to problems during pregnancy.  Remember, even though being overweight can lead to these problems, there are ways to help prevent them. Eating healthy and staying active are two big ones. If you're overweight and worried about these issues, a doctor can give you advice on what to do. |
| 5 | What are the pre-operative requirements in order to undergo either operation? | You must have a stable relationship with a primary care physician who has been treating you continuously for one year prior to consideration for surgery.  You must have a dietician evaluation.  You must have a psychological evaluation. Patients with bipolar disease are generally excluded from consideration for surgery.  If you have a history of heart disease, if you've taken Fen-Phen in the past, if you're over the age of 50, or other cardiac risk factors, you'll require an echocardiogram.  At some point prior to surgery, you'll have to undergo a chest x-ray, an EKG and blood tests. | What are the pre-operative requirements in order to undergo weight loss surgery? | Pre-operative requirements for weight loss surgery can vary between individual hospitals and surgeons, as well as depending on the patient's individual health condition. However, common pre-operative requirements usually include the following:  1. **Medical Evaluation:** This is done to assess the overall health status of the patient and includes blood tests, imaging tests, a physical exam, and other tests as needed to check for conditions like heart disease, high blood pressure, diabetes, and sleep apnea that can affect surgery outcomes.  2. **Nutritional Counseling:** This involves meeting with a dietitian or nutritionist who can guide the patient on how to follow a healthier diet before and after surgery. Some programs may require patients to follow a specific diet plan and even lose a certain amount of weight before surgery.  3. **Psychological Evaluation:** This is conducted to ensure that the patient understands the implications of the surgery, can cope with significant lifestyle changes, and does not have untreated mental health disorders like severe depression or binge-eating disorder.  4. **Commitment to Lifestyle Changes:** Weight loss surgery is not a magic bullet. It requires a lifelong commitment to diet and lifestyle changes. This might include regular exercise, avoiding alcohol and smoking, and following a strict dietary plan.  5. **Stop Certain Medications:** Some medications may need to be stopped or altered before surgery, particularly those that can increase the risk of bleeding, such as aspirin or other blood thinners.  6. **Pre-operative Weight Loss:** Some bariatric programs require patients to lose some weight before surgery. This can decrease the risk of complications and make the surgery easier to perform.  7. **Quit Smoking:** If you smoke, you'll likely be asked to stop several weeks before your procedure. Smoking can impair your body's ability to recover.  8. **Attendance at Pre-operative Education Sessions:** These sessions typically cover information about the surgery, its risks and benefits, the recovery process, and lifestyle changes needed after surgery.  9. **Clearance from Specialists:** If you have certain other health conditions, you may need clearance from a specialist. For example, if you have heart disease, you may need clearance from a cardiologist.  It's important to follow all pre-operative instructions provided by your healthcare team to minimize risks and improve outcomes. Always consult your healthcare provider or surgeon for personalized medical advice.  Please note that as an AI developed by OpenAI with a knowledge cutoff in September 2021, the information I provide is accurate up to that date. Always consult with your healthcare provider for the most current information. | 7/3/2023 | Sure! Here's a simpler version of the steps you need to follow before weight loss surgery:  1. **Health Checkup:** The doctors need to see how healthy you are. They do tests like blood tests and check for other diseases.  2. **Diet Counseling:** A diet expert will tell you what to eat and not eat before and after the surgery. You might even need to lose some weight before the surgery.  3. **Mental Health Check:** A mental health professional will talk with you to make sure you understand what the surgery means and how your life will change afterward. They also check if you're generally feeling okay mentally.  4. **Change Habits:** Surgery isn't a quick fix. You'll need to promise to eat healthy, exercise, and not smoke or drink too much alcohol after the surgery.  5. **Medications:** You might need to stop taking some medicines before the surgery, especially those that might make you bleed more during the surgery.  6. **Weight Loss:** Some doctors want you to lose a bit of weight before the surgery. This can make the surgery safer and easier.  7. **Stop Smoking:** If you smoke, you should stop for a few weeks before the surgery. Smoking can slow down your body's healing process.  8. **Learn About the Surgery:** You'll go to classes to learn about the surgery, how it will help you, and what you need to do afterward to stay healthy.  9. **Clearance from Specialists:** If you have other health problems, you might need to see a special doctor who can say it's okay for you to have the surgery.  These are general steps. It's always best to talk to your own doctor who can tell you exactly what you need to do before your surgery. |
| 5 | What does laparoscopic surgery mean? | Laparoscopic surgery means that instead of making one large incision in your abdomen, we make five small incisions in the middle and to the left side of your abdomen. These smaller incisions allow you to recover faster. You'll also experience less pain, leave the hospital sooner and will be back to your normal activity sooner. In general, we start all of our cases laparoscopically unless the patient has had previous surgery on their stomach. |  | Laparoscopic surgery, also known as minimally invasive surgery or keyhole surgery, is a modern surgical technique in which operations in the abdomen are performed through small incisions, usually 0.5–1.5 cm, as opposed to larger incisions needed in traditional surgical procedures.  This method utilizes a laparoscope, a long fiber optic cable system which allows viewing of the affected area by snaking the cable from a more distant, but more easily accessible location. The laparoscope is connected to a tiny camera that projects the images onto a video monitor so the surgeon can see inside the patient's body without making large incisions.  Laparoscopic surgery includes operations within the abdominal or pelvic cavities, whereas keyhole surgery performed on the thoracic or chest cavity is known as thoracoscopic surgery.  Benefits of laparoscopic surgery compared to open surgery include reduced pain due to smaller incisions, reduced bleeding and shorter recovery time. However, like any surgery, it does have risks, including infection, bleeding, and complications due to anesthesia. The appropriateness of laparoscopic surgery is decided on a case-by-case basis, depending on the patient's condition and the nature of the problem. | 7/3/2023 | Of course!  Laparoscopic surgery, sometimes called "keyhole surgery," is a way doctors can do surgery without making a big cut. They make a few small cuts and insert a thin tube with a camera on the end into one of these cuts. This camera is called a laparoscope.  The camera sends pictures to a screen, so the doctors can see inside your body. The doctors use long, thin surgical instruments in the other small cuts to perform the surgery. This method is used because it usually causes less pain, leaves smaller scars, and allows quicker recovery compared to surgeries with larger cuts. |
| ASMBS | When can I start exercising after surgery? | Right away! To start, you will take easy, short walks while you are in the hospital. The key is to start slow. Listen to your body and your surgeon. If you lift weights or do sports, stay “low impact” for the first month. | When can I start exercising after weight loss surgery? | After weight loss surgery, it's essential to begin exercising to help accelerate weight loss and improve overall health. However, the exact timing will depend on the type of procedure you've had, your overall health status, and your surgeon's recommendations.  Generally, you can start with light activity like walking or gentle stretching soon after surgery, often within a few days. This initial activity is important to help prevent complications such as blood clots, promote healing, and start building your physical fitness.  More vigorous exercises, such as weight lifting, high-intensity cardio, or abdominal workouts, should usually be avoided for at least six weeks post-surgery, or until your surgeon gives you clearance.  Each person's body responds differently to surgery, so it's essential to listen to your body and not push yourself too hard, too soon. If you feel pain or discomfort during an activity, stop and rest.  Also, remember that exercise is only one part of a healthy post-surgical lifestyle. Eating a balanced diet, staying hydrated, getting adequate sleep, and regularly checking in with your healthcare provider are all essential elements of your recovery and long-term success.  Always consult with your surgeon or a qualified healthcare professional before starting or changing your exercise regimen. | 7/3/2023 | Sure, let's make this simpler.  After weight loss surgery, you'll want to start moving a bit to help you heal and lose weight.  Right after your surgery, you can do very easy activities like walking or stretching. This could start just a few days after your operation.  For harder exercises, like lifting weights or doing lots of cardio, you should wait about six weeks, or until your doctor says it's okay.  Remember, don't push yourself too hard. If something hurts, stop and rest. And make sure to eat healthy, drink lots of water, get good sleep, and see your doctor regularly.  Always check with your doctor before you start or change your exercise plan. |
| ASMBS | Will I have to exercise after the procedure? | For many patients, exercise is important for stress control and appetite control, as well as burning off calories.  As we age, lack of activity can lead to being frail or fragile, which is quite dangerous to overall health. Healthy bones and avoiding muscle loss partly depends on doing weekly exercise.  Most patients also think of exercise as something that must be intense and painful (like “boot camp”). Regular, modest activity is far more useful in the long term. Even elite athletes can’t stay at a “peak” every week of the year. Instead, work with your surgeon’s program to find a variety of activities that can work for you. There is no “one-size-fits-all” plan. Expect to learn and change as you go! | Will I have to exercise after weight loss surgery? | Yes, exercise is a crucial component of the lifestyle changes needed to make the most of weight loss surgery. While the surgery itself can help you lose weight initially, maintaining that weight loss and continuing to lose weight often requires changes in diet and exercise habits.  There are several reasons why exercise is especially important after weight loss surgery:  1. Enhances Weight Loss: Combined with a healthy diet, regular physical activity can help you achieve and maintain a healthy weight. Exercise burns calories and can support faster weight loss.  2. Improves Cardiovascular Health: Regular exercise helps to lower blood pressure, improve cholesterol levels, and reduce the risk of heart disease, all of which can be concerns for individuals who are overweight or obese.  3. Increases Muscle Mass: Weight loss surgery can sometimes lead to loss of muscle mass, especially if you're losing weight rapidly. Exercise, particularly strength training, can help you build and preserve muscle mass.  4. Boosts Mood and Energy Levels: Regular physical activity can help manage stress, improve sleep, and increase energy levels, leading to better overall mental health.  5. Helps Regulate Blood Sugar: Exercise can help control blood sugar levels, reducing the risk of developing type 2 diabetes or managing the condition if you already have it.  Before starting any exercise program, it's essential to consult with your healthcare team. They can help design an exercise plan that's appropriate for your current fitness level and health status, and they can monitor your progress as you become more physically active. | 7/3/2023 | Of course! After weight loss surgery, it's really important to exercise. Here's why:  1. Lose More Weight: Exercise helps you burn more calories, which can help you lose more weight and keep it off.  2. Heart Health: Exercise is good for your heart. It can help lower blood pressure and improve cholesterol levels.  3. Keep Muscles: Sometimes, weight loss surgery can cause you to lose muscle. Exercise, like lifting weights, can help you keep your muscle while losing fat.  4. Feel Better: Exercise can help you feel more energetic, sleep better, and feel happier.  5. Control Blood Sugar: Exercise can help keep your blood sugar levels in check, which is important if you have diabetes or are at risk of getting it.  Before you start exercising, talk to your doctor. They can help you figure out the best exercises for you and how to do them safely. |
| ASMBS | Will I have to go on a diet before I have surgery? | Yes. Many insurance companies require patients to be on a weight loss program before qualifying for surgery.  Many bariatric surgeons put their patients on a special pre-operative diet, usually 2 or 3 weeks just before surgery. The reason for the pre-operative diet is to shrink the liver and reduce fat in the abdomen. This helps during the procedure and makes it safer.  Some insurance companies require a physician-monitored diet three to six months prior to surgery as part of their coverage requirement. These diets are very different from the short-term diets and usually are more about food education and showing a willingness to complete appointments and to learn. | Will I have to go on a diet before I have weight loss surgery? | Before bariatric surgery, such as gastric bypass, gastric sleeve, or lap-band, most doctors will recommend a specific diet. This diet usually starts a few weeks before the surgery. The primary aim of this diet is to reduce the amount of fat in and around your liver. A large liver can make the surgery more complicated, so the diet is used to shrink the liver to make the operation safer and more efficient.  The pre-surgery diet often includes the following components:  1. **High Protein Intake**: This is important because it helps maintain muscle tissue and keep you feeling full.  2. **Low Carb and Low Sugar**: Carbohydrates and sugar increase the fat around the liver, so it's best to keep them to a minimum.  3. **Limited Fats**: Healthy fats are allowed in small quantities, but it's best to limit them to maintain a calorie deficit.  4. **Lots of Fluids**: Drinking lots of fluids (preferably water) keeps you hydrated and helps to flush toxins out of your body.  5. **Vitamins and Minerals**: Because the diet is restrictive, it's important to supplement with vitamins and minerals to ensure you're not becoming deficient.  This pre-surgery diet isn't only about weight loss. It's also about getting your body, particularly your liver, in the best possible shape for the surgery. Every patient is unique, so your doctor or a dietitian will provide you with the best pre-surgery diet based on your specific health needs.  After the surgery, you'll also have to follow a specific diet that will help you adapt to your new stomach size and promote weight loss, but the specifics of that diet will depend on the type of surgery you have.  Remember, before starting any diet or exercise program, especially if you're preparing for surgery, it's important to consult with healthcare professionals who know your personal medical history. | 7/3/2023 | Of course, I'd be happy to simplify that for you.  Before weight loss surgery, your doctor will usually want you to follow a special diet for a few weeks. This diet helps you lose a bit of weight and shrinks your liver, which makes the surgery easier and safer.  Here's what that diet usually involves:  1. **Eat Lots of Protein**: This helps keep your muscles strong and makes you feel full.  2. **Don't Eat Much Sugar or Carbs**: Too much sugar or carbs can make your liver fatty. We want to avoid this.  3. **Limit Fats**: Eating a little bit of healthy fats is okay, but too much isn't good.  4. **Drink Lots of Water**: This helps your body stay clean and hydrated.  5. **Take Vitamins and Minerals**: Since you won't be eating as much, these help make sure your body gets what it needs.  The diet isn't just to lose weight, it's also to get your body ready for the surgery. After the surgery, your doctor will give you another special diet to follow.  Always remember to talk to your doctor or a dietitian before starting any diet, especially if you're getting ready for surgery. They can give you the best advice based on your personal health needs. |
| ASMBS | Will I have to be on a diet after the surgery? | No. Most people think of a “diet” as a plan that leaves you hungry. That is not the way people feel after surgery.  Eventually, most patients get some form of appetite back 6-18 months after surgery. Your appetite is much weaker, and easier to satisfy than before.  This does not mean you can whatever and whenever you want. Healthier food choices are important for best results, but most patients still enjoy tasty food, and even occasional “treats”. | Will I have to be on a diet after weight loss surgery? | Yes, a dietary change is almost always required after weight loss surgery, and it is an important part of a successful weight loss journey. Here's what you can generally expect:  1. **Immediate post-surgery diet**: Right after surgery, your doctor will likely put you on a liquid diet. This can last for a few weeks and will include things like water, broth, and juice.  2. **Pureed diet**: After your body has adjusted to the liquid diet, you may progress to a pureed diet. This will consist of foods that have been blended to a smooth consistency, like mashed potatoes and pudding.  3. **Soft food diet**: Once your body has adjusted to pureed foods, you can typically move on to soft, easily chewable foods. This phase can last for a while, often a few weeks.  4. **Regular diet**: Eventually, you'll transition to a more regular diet, though it will still need to be low in sugar and fat. The timing for this transition varies widely based on individual circumstances and your surgeon's guidance.  Even after you've transitioned to a regular diet, it's still important to maintain healthy eating habits. Typically, this will involve:  - **Eating small, frequent meals**: Your new stomach pouch after weight loss surgery will not hold as much food as it did before. Eating small, frequent meals can help ensure you're getting enough nutrients without overeating.  - **Prioritizing protein**: Protein helps preserve muscle mass during rapid weight loss and can also help you feel full.  - **Limiting sugars and fats**: These can not only contribute to weight regain but also cause dumping syndrome, a condition where food moves too quickly into the small intestine causing uncomfortable symptoms like nausea, vomiting, and diarrhea.  - **Staying hydrated**: It's important to drink enough fluids, but avoid drinking at the same time as eating because this can cause your new stomach pouch to fill up too quickly.  - **Taking vitamins and minerals**: After weight loss surgery, your body might not absorb certain nutrients as well as before, so you may need to take supplements.  Remember, this is a general guide, and your specific dietary needs and progression may vary. Always consult with your healthcare team before making any changes to your diet or supplement routine. | 7/3/2023 | Sure! After weight loss surgery, you will need to change what and how you eat. Here's a simpler explanation:  1. **Right after surgery**: You'll drink only liquids like water and soup for a few weeks. This helps your stomach heal.  2. **A bit later**: You'll eat foods that are pureed or blended into a smooth texture, like mashed potatoes. This step helps your stomach get used to more solid foods.  3. **After that**: You'll start eating soft foods that are easy to chew.  4. **Finally**: You'll begin to eat normal, but healthy foods. You'll eat less sugar and fat, and more proteins.  Some other things to keep in mind:  - You'll eat smaller meals, more often, because your stomach will be smaller.  - You'll need to drink plenty of water, but not while you're eating.  - You might need to take extra vitamins because your body might not get as much from food anymore.  Remember, you should always listen to your doctor's advice about what to eat after surgery. |
| ASMBS | Is weight loss surgery a ‘cop-out’? To lose weight, should people with obesity just go on a diet and exercise? | Many people who suffer from obesity find it hard to lose weight and keep the weight off with diet and exercise alone. This is likely due to foods available and genes.  The National Institutes of Health (NIH) Expert Panel stated that, without surgery, long-term weight loss is nearly impossible for those affected by severe obesity. Studies show little long-term success with diet and exercise alone.(3) Weight loss surgeries are effective in maintaining long-term weight loss, in part, because these procedures change the body’s natural responses to dieting that make weight loss so difficult. When a person goes on a diet, their body produces more hormones that cause an increase in hunger and a decrease in calories burned. This decrease in calories the body burns is more than explained by the decrease in body size. Therefore, there are significant differences between someone who has lost weight by diet and someone of the same size who has never lost weight. For example, the body of the person who reduces their weight from 200 to 170 pounds burns fewer calories than the body of someone weighing 170 pounds who has never been on a diet. This means that in order to maintain weight loss, the person who has been on a diet will have to eat fewer calories than someone who naturally weighs the same. Weight loss procedures, unlike diet, also cause biological changes that help reduce food intake. Energy (in the form of food) intake is decreased with surgery by restricting stomach size and limiting absorption. In addition, weight loss surgery changes the production of certain gut hormones (or signals) that communicate with the brain to reduce hunger, decrease appetite, and enhance the feeling of being full. In these ways, weight loss surgery, unlike dieting, produces long-term weight loss. |  | Whether weight loss surgery is seen as a 'cop-out' or not depends largely on one's perspective and understanding of obesity. Obesity is a complex disease with a multitude of factors involved, including genetics, environment, metabolism, and behavior. It's not simply a question of willpower or discipline, and solutions aren't as straightforward as just diet and exercise for many people.  For some individuals, particularly those with severe obesity or obesity-related health conditions such as type 2 diabetes or sleep apnea, weight loss surgery (also known as bariatric surgery) may be the most effective treatment. These surgeries can lead to substantial weight loss and improvements in health, and for some, it's a life-saving intervention.  Weight loss surgery is not an easy way out. The surgery itself carries risks, as does any surgery, and requires a significant commitment to lifelong dietary, exercise, and lifestyle changes. Without these changes, weight regain can occur. In addition, patients often need to engage in psychological or behavioral support to adjust to their new lifestyle and body image.  It's also worth noting that weight loss surgery isn't for everyone. Typically, it's considered for people who have a body mass index (BMI) above 40, or above 35 if they also have a weight-related health problem.  The decision to pursue weight loss surgery should be made in consultation with a healthcare professional, considering the person's overall health, readiness to make behavioral changes, and personal preferences.  In essence, referring to bariatric surgery as a 'cop-out' oversimplifies the complex nature of obesity and discounts the challenges that people living with obesity face in their attempts to achieve and maintain weight loss. It's important to approach this subject with empathy and understanding, recognizing that weight loss methods that work for one person may not work for another. It's a deeply personal decision that should be based on what's best for the individual's health and well-being. | 7/3/2023 | Sure, let's simplify it.  Obesity is a complex problem, influenced by many things like our genes, our environment, and our lifestyle. It's not just about willpower or making simple choices like diet and exercise.  For some people who are very overweight, or who have serious health problems because of their weight, weight loss surgery might be the best option. This surgery can lead to big weight loss and better health. But it's not an "easy way out." The surgery has risks and after the surgery, people need to make major changes in their eating and exercise habits. If they don't make these changes, they could gain the weight back.  Saying that weight loss surgery is a "cop-out" doesn't consider how difficult obesity can be to manage. What works for one person may not work for another, and it's important to decide on a weight loss method based on personal health needs and circumstances. This decision should be made with the help of a healthcare professional. |
| ASMBS | When can I get pregnant after weight loss surgery? | Most groups advise waiting 12-18 months after surgery before getting pregnant.  Most women are much more fertile after surgery, even with moderate weight loss.  Birth control pills do NOT work as well in patients who are overweight. Birth control pills are not very reliable during the time your weight is changing. For this reason, having an IUD or using condoms and spermicide with ALL intercourse is needed. Menstrual periods can be very irregular, and you can get pregnant when you least expect it! |  | After weight loss surgery, it's generally recommended to wait at least 12 to 18 months before trying to get pregnant. There are a few reasons for this.  Firstly, the period immediately following bariatric surgery is a time of significant weight loss and nutritional changes. Rapid weight loss can temporarily decrease fertility, and the metabolic and hormonal changes that occur during this time can impact fetal development.  Secondly, many types of bariatric surgery reduce the absorption of nutrients from the food you eat. This can lead to nutritional deficiencies, which can be harmful to both mother and baby. It's important to ensure that nutrient levels are stable and within a healthy range before pregnancy.  It's also worth noting that following bariatric surgery, the body can go through other changes such as hormonal shifts and changes in menstrual cycle regularity. These shifts can sometimes improve fertility in individuals who were previously struggling to conceive due to issues like polycystic ovary syndrome (PCOS), which is common in individuals with obesity.  However, each person's situation is unique, and the specific timing may depend on factors like the type of weight loss surgery, the amount of weight lost, overall health status, age, and personal fertility factors. Therefore, it's very important to discuss your plans for pregnancy with your bariatric surgeon and your OB-GYN. They can provide advice tailored to your specific situation and help you plan for a safe and healthy pregnancy after weight loss surgery. | 7/3/2023 | Sure, I can simplify that. After weight loss surgery, doctors usually say you should wait at least 12 to 18 months before trying to get pregnant. That's because your body goes through a lot of changes after this type of surgery.  Right after surgery, you'll lose a lot of weight quickly. This can mess with your body's normal processes and might make it harder to get pregnant. Also, some surgeries make it hard for your body to get enough nutrients from food, which isn't good for you or a baby.  If you're thinking about getting pregnant after weight loss surgery, you should talk to your surgeon and a doctor who specializes in pregnancy. They can help you figure out when it's safe for you to try to have a baby. |
| ASMBS | Will the baby be healthy? | Yes.  After surgery, there is much less risk of experiencing problems during pregnancy and during childbirth. There are also fewer miscarriages and stillbirths than in women with obesity who have not had surgery and weight loss.  Kids born after mom’s surgery are LESS at risk of being affected by obesity later, due to activation of certain genes during fetal growth. There is also less risk of needing a C section. | Will the baby be healthy (posted in the same chat as the previous question) | Many women who become pregnant after weight loss surgery have healthy pregnancies and healthy babies. In fact, losing excess weight before pregnancy can actually improve some pregnancy outcomes, particularly for women who had health conditions related to obesity before surgery, such as type 2 diabetes or high blood pressure. Weight loss can reduce the risk of these conditions complicating the pregnancy.  That said, there are some potential risks associated with pregnancy after weight loss surgery. One important risk to be aware of is malnutrition. As mentioned earlier, some types of weight loss surgery reduce the absorption of nutrients from food, which can lead to deficiencies in important vitamins and minerals. This is a concern because good nutrition is crucial for fetal development. To prevent this, it's important to work closely with your healthcare provider to ensure that you're getting the right nutrients, either through diet or supplements.  Another risk is that of small-for-gestational-age babies. Some research suggests that babies born to mothers who have had bariatric surgery may be more likely to be smaller than average for their gestational age, although other studies have found no such association.  On balance, most experts believe that the benefits of weight loss surgery for severely obese women likely outweigh these risks, particularly if the surgery results in significant improvements in the mother's health.  Every pregnancy has its unique set of risks and benefits, and this is also true for pregnancies after weight loss surgery. Therefore, it's very important to plan carefully and to receive regular prenatal care. You should discuss your specific situation with your bariatric surgeon and your OB-GYN to ensure the healthiest possible pregnancy. | 7/3/2023 | Absolutely, I'd be happy to simplify it.  After having weight loss surgery, it's a good idea to wait about a year to a year and a half before trying to get pregnant. This is because your body is going through a lot of changes during this time as it adjusts to the surgery and loses weight.  Having a baby after weight loss surgery can be healthy for both mom and baby. In fact, losing weight can make the pregnancy healthier if the mom had problems like diabetes or high blood pressure before the surgery.  However, there can be some risks. One big one is that after weight loss surgery, your body might not take in all the vitamins and nutrients from food that it used to. This is important because the baby needs these nutrients to grow properly.  Sometimes, babies born to moms who had weight loss surgery might be smaller than average. But it's also possible that they'll be a normal size.  Even with these risks, many doctors think weight loss surgery can be a good thing for women who are very overweight, because it can make them and their future babies healthier.  Every woman, every pregnancy, and every baby is different. So, if you're thinking about getting pregnant after weight loss surgery, it's a good idea to talk to your doctor. They can help you understand what to expect and how to take care of yourself and your baby. |
| ASMBS | How long after weight loss surgery will I have to be from work? | After surgery, most patients return to work in one or two weeks. |  | The length of time you'll need to take off work after weight loss surgery can depend on several factors including the type of surgery you've had, your general health, and the nature of your work.  Generally, for laparoscopic surgeries like gastric bypass or sleeve gastrectomy, patients might return to work within 2 to 4 weeks. Open surgery, which is more invasive, may require 4 to 6 weeks of recovery time before returning to work.  However, if your job is physically demanding and involves heavy lifting or strenuous activity, you might need to take more time off or consider light duty, if possible.  Remember to consult with your doctor or healthcare provider for personalized advice on this matter. It's essential to ensure that you allow sufficient time for recovery to avoid complications and promote effective healing. | 7/3/2023 | Of course!  After weight loss surgery, you will need some time to rest and heal. This time can change based on a few things:  1. The type of surgery: Some surgeries are more complex and need more healing time.  2. Your health: If you're healthy overall, you might recover faster.  3. Your job: If your job is physically hard, like lifting heavy things, you might need more time before you can go back.  Usually, for simpler surgeries, you might be able to go back to work in 2 to 4 weeks. For more complex surgeries, it could take 4 to 6 weeks. Always talk to your doctor to understand what's best for you. |
| ASMBS | Will I lose my hair after surgery? | Some hair loss is common between 3 and 6 months following surgery but almost always temporary.  The reasons for hair loss are not totally understood. Even if you take all recommended supplements and meet protein requirements, hair loss will be noticed until the follicles come back. Adequate intake of protein, vitamins, and minerals will help to ensure hair re-growth, and avoid longer term thinning. | Will I lose my hair after weight loss surgery? | While hair loss after bariatric surgery (weight loss surgery) is not guaranteed, it is a potential side effect that some people experience. This is usually due to a condition called telogen effluvium, which is a temporary disruption in the natural hair growth cycle. Telogen effluvium can occur after significant physiological stress, such as major surgery, drastic weight loss, or malnutrition.  One of the reasons for hair loss after weight loss surgery can be nutritional deficiencies. Bariatric surgery often changes the way your body absorbs nutrients from the food you eat, and certain procedures can result in deficiencies in protein, iron, vitamin B12, folate, and other nutrients vital for hair health.  Here are some ways to manage and potentially prevent hair loss after weight loss surgery:  1. Nutrition: Ensure your diet is nutritionally complete and balanced. Consult a nutritionist if necessary.  2. Protein: Make sure you get enough protein. Protein is essential for hair growth and general health.  3. Vitamins and minerals: Consider taking multivitamins or specific supplements like iron, zinc, or biotin, but always do so under the supervision of a healthcare professional.  4. Hydration: Stay well-hydrated.  5. Gentle care: Be gentle with your hair, avoiding harsh treatments and styles that can cause breakage or stress to the hair follicles.  Please remember, each individual is different and these measures may not completely prevent hair loss for everyone. Most importantly, consult your doctor or a dietician for personalized advice. Also, keep in mind that if hair loss occurs, it is typically temporary, and your hair should start growing back once your body adjusts to the changes after surgery. | 7/3/2023 | Sure, I can simplify that for you.  Sometimes after weight loss surgery, people might experience hair loss. This is because the body has been through a lot of changes and stress from the surgery and losing weight quickly. Also, the surgery can make it harder for your body to get all the nutrients it needs from the food you eat, and these nutrients are important for healthy hair.  Here are some easy tips to help prevent or manage this hair loss:  1. Eat a healthy and balanced diet. Your body needs good nutrition for your hair to grow.  2. Eat enough protein. Protein is very important for hair growth.  3. Take vitamins or supplements, but only if your doctor says it's okay. Certain vitamins like iron, zinc, or biotin can help with hair health.  4. Drink lots of water.  5. Be gentle with your hair. Avoid things that might damage it.  Remember, everyone is different and these tips may not work for everyone. But if you do lose some hair, it will usually grow back after your body gets used to the changes from the surgery. Always talk to your doctor if you're worried about hair loss. |
| ASMBS | Will I need to have plastic surgery after weight loss? | Some patients may choose to have plastic surgery, but this depends on many factors. | Will I need to have plastic surgery after weight loss from bariatric surgery? | Whether or not you'll need plastic surgery after bariatric surgery (a procedure that helps with weight loss) depends on several factors, including how much weight you lose, your age, your overall health, and your personal preference.  Bariatric surgery can lead to significant weight loss, and in some cases, this can leave loose or excess skin that doesn't shrink back to its original size. This is especially common in areas such as the abdomen, arms, and thighs. Some people find that this loose skin is uncomfortable or they don't like the way it looks, which is when they might consider plastic surgery.  On the other hand, not everyone who has bariatric surgery chooses to have plastic surgery afterward. Some people's skin shrinks back more than others', and some people are comfortable with or even proud of the changes to their body after weight loss.  If you're considering bariatric surgery, it's a good idea to talk to your doctor about what to expect afterward, including the possibility of loose skin. You may also want to speak with a plastic surgeon who specializes in body contouring after weight loss to understand what procedures might be available to you, what the risks and benefits are, and what kind of results you can expect. But the decision to have plastic surgery is a personal one and it's entirely up to you.  It's also important to remember that both bariatric surgery and plastic surgery after weight loss are major procedures that carry risks, so it's crucial to consider all the pros and cons and make an informed decision. | 7/3/2023 | Of course! When you have surgery to lose a lot of weight, like bariatric surgery, sometimes you end up with extra skin because your body shrinks faster than your skin can keep up. This might make you uncomfortable or you might not like how it looks.  Whether or not you choose to have another surgery to remove this extra skin is up to you. Some people choose to do it, and some don't. It can help to talk to your doctor or a surgeon who specializes in removing extra skin after weight loss, so you understand all the choices you have, and what the risks and benefits might be. Both weight loss surgery and surgery to remove extra skin are big procedures that have risks, so it's important to think carefully and get all the information you can. |
| ASMBS | Do most people who have weight loss surgery regain their weight? | No. A small number of patients can regain their weight but the vast majority lose significant weight and keep this weight off.  More than 95% of patients will successfully lose half of their extra body weight or more after surgery for weight loss.  (1) Roux en Y gastric bypass patients, for example, on average lose approximately 70% of their extra body weight initially, and 2 years or more after surgery will regain approximately 5% of their extra body weight.  (2) Compared to these results, patients who attempt weight loss without surgery, on average, are able to lose little weight and may gain weight in the long-term. |  | Weight loss surgery, also known as bariatric surgery, can be a very effective tool for achieving significant weight loss. However, it is not a magic solution and requires ongoing commitment to a healthy lifestyle in order to maintain the weight loss long term.  Many studies suggest that while some weight regain after bariatric surgery is common, most people do not return to their pre-surgery weight. According to a study published in the journal "Obesity Surgery", on average, patients regain less than 5% of their lost weight 10 years after surgery.  However, the amount of weight regain can depend on several factors including the type of surgery (e.g., gastric bypass, sleeve gastrectomy, gastric banding), the individual’s adherence to the prescribed diet and exercise program, psychological factors, and other individual health considerations.  It's important to remember that weight loss surgery isn't an end in itself, but a tool to help with weight loss. It is most successful when combined with a comprehensive treatment plan, including changes to diet and physical activity habits, and often psychological support. The key to long-term success after weight loss surgery is a lifelong commitment to a healthy lifestyle.  Remember that all patients are different and individual results can vary. This information should be discussed with a healthcare provider for an understanding of what to expect based on personal health history. | 7/3/2023 | Sure! When people get weight loss surgery, it helps them lose a lot of weight. But to keep that weight off, they also need to eat healthy and exercise regularly after the surgery.  Most people do gain back some weight after the surgery, but usually not all of it. So if someone was very overweight before the surgery, they're likely to still be a lot lighter after the surgery even if they gain back a little weight.  But it's different for everyone. Some people might gain more weight back, and others might not gain much at all. It depends on the type of surgery, how well they stick to healthy habits, their mindset, and their overall health.  To have the best chance of keeping the weight off, it's important to work with doctors and other health professionals to stay healthy and active after the surgery. |
| ASMBS | Can I go off some of my medications after weight loss surgery? | Many patients are able to stop using some medications. |  | As an AI developed by OpenAI, I can tell you that it's possible some of your medications may be adjusted after weight loss surgery, particularly if the medications are related to conditions that can improve with weight loss, such as type 2 diabetes, hypertension, or sleep apnea.  However, it's extremely important to remember that decisions about medication should be made in consultation with your healthcare provider. Every person is unique, and what works for one person might not work for another. Also, some medications need to be adjusted gradually, and stopping suddenly could have serious consequences.  After weight loss surgery, you'll likely be working closely with a medical team that includes a surgeon, a dietitian, and possibly other specialists. These professionals will be able to give you guidance based on your specific situation and health status.  Remember to keep open lines of communication with your healthcare team, ask them any questions you have, and follow their advice about medication adjustments. | 7/3/2023 | Sure, I can make that simpler. After weight loss surgery, you might be able to stop taking some of your medicines, especially if they are for things like high blood pressure or diabetes, which can get better with weight loss.  But, it's very important to remember to only make these changes if your doctor says it's okay. Everyone is different, so what works for one person might not work for you. Plus, some medicines can be harmful if you stop them all at once.  After your surgery, you'll be seeing your doctor and other health experts often. They'll help guide you and decide what is best for you. Make sure to ask them any questions you have and follow their advice about your medicines. |
| ASMBS | Will I have to take vitamins after surgery? | You will need to take a multivitamin for life.  You may need higher doses of certain vitamins or minerals, especially Iron, Calcium, and Vitamin D. You will also need to have at least yearly lab checks. Insurance almost never pays for vitamin and mineral supplements but usually does pay for labs. You can pay for supplements out of a flex medical account. | Will I have to take vitamins after weight loss surgery? | After weight loss surgery, especially procedures like the gastric bypass, sleeve gastrectomy, and duodenal switch, many patients will need to take vitamins and supplements for the rest of their lives. This is because these surgeries can change how the body absorbs nutrients.  Here are some of the most common supplements that doctors often recommend:  1. **Multivitamins**: To meet your daily requirements for vitamins and minerals.  2. **Vitamin B12**: Because these types of surgeries can affect how your body absorbs B12, which is important for brain function and the production of red blood cells.  3. **Iron**: To prevent anemia, especially in women who are still menstruating.  4. **Calcium**: To support bone health, as these surgeries can impact the body's ability to absorb calcium.  5. **Vitamin D**: Often paired with calcium for better absorption and bone health.  6. **Protein supplements**: To help meet protein needs, as it can be hard to consume enough protein through diet alone after surgery.  Each patient is different, so your doctor will determine the exact mix and dosages of vitamins and supplements you'll need based on your individual health profile, the type of surgery you had, and your dietary habits.  Remember that these supplements are not a substitute for a balanced diet. It's important to follow a healthy, balanced diet to get the most nutritional benefit and to help maintain your weight loss. It's also crucial to follow up regularly with your healthcare provider to monitor your nutrient levels and overall health.  As always, it is important to discuss any potential changes to your dietary and supplementation regimen with your healthcare provider before implementing them. | 7/3/2023 | Of course, I'd be happy to simplify.  After weight loss surgery, your body can't absorb some nutrients as well as it used to. Because of this, you'll probably need to take some vitamins and supplements every day. Here are some you might need:  1. **Multivitamins**: These give you many different kinds of vitamins and minerals that your body needs.  2. **Vitamin B12**: This vitamin helps your brain work well and helps make red blood cells.  3. **Iron**: This helps prevent you from getting too tired, especially for women who have periods.  4. **Calcium**: This is important for strong bones.  5. **Vitamin D**: This helps your body use the calcium for your bones.  6. **Protein supplements**: These help you get enough protein, which can be hard after weight loss surgery.  Your doctor will tell you which vitamins and how much of each you should take. This will depend on your health, the type of surgery you had, and what you eat.  Even though you take these vitamins, it's still important to eat healthy food. And you should see your doctor often to make sure your body is getting what it needs and you're staying healthy.  Before you start taking any new vitamins or supplements, be sure to talk to your doctor first. |
| ASMBS | Do weight loss surgery patients have serious health problems caused by vitamin deficiency? | As long as patients take their appropriate vitamins, it is rare to have health problems from vitamin deficiency.  Weight loss surgery can lead to reduced amounts of vitamins and minerals because people eat less and may absorb less in the intestines. Weight loss surgeries have different effects on vitamins and minerals based on how much change in absorption they cause. Deficiencies in vitamins, minerals, and protein can negatively affect health causing:  - fatigue (feeling tired)  - anemia (low levels of red blood cells that carry oxygen through your body)  - bone and muscle loss  - impaired night vision  - low immunity (your body’s ability to stay healthy)  - loss of nerve function (can affect your senses such as touch, taste, and smell)  - mental function deficits (changes how clearly you think)  Fortunately, nutrient deficiencies following surgery can be easily avoided with a good diet and the use of supplements including vitamins, minerals, and sometimes protein supplements. Before and after surgery, patients are advised of the appropriate diet and vitamin supplement needed. Most weight loss surgery programs also require patients to have their vitamins and minerals checked on a regular basis after surgery. Nutrient deficiencies and any associated health problems are preventable with monitoring and patients following diet and supplement (vitamin and mineral) recommendations. Health problems due to deficiencies usually occur in patients who do not regularly follow-up to monitor healthy vitamin and mineral levels. Be sure you stay in touch with your weight loss surgery team for best results! |  | Weight loss surgeries, such as gastric bypass, sleeve gastrectomy, and adjustable gastric band, alter the digestive system to aid weight loss. While they can be highly effective in helping individuals lose significant weight and improve health conditions related to obesity, they can also have unintended side effects. One such side effect is the potential for vitamin and mineral deficiencies.  Because these surgeries change the way the body absorbs food and nutrients, patients can become deficient in vitamins and minerals like iron, calcium, zinc, copper, and vitamins B12, D, A, and K. These deficiencies can lead to serious health problems if not properly managed.  Health problems due to nutrient deficiencies after bariatric surgery may include:  1. Iron deficiency anemia: This could result in fatigue, weakness, and paleness.  2. Calcium deficiency: This could lead to osteoporosis or weak and brittle bones.  3. Vitamin B12 deficiency: This might cause anemia, nerve damage, fatigue, weakness, and problems with balance.  4. Vitamin D deficiency: This could lead to bone pain and muscle weakness.  5. Vitamin A deficiency: This might result in night blindness, skin issues, and a compromised immune system.  6. Thiamine (Vitamin B1) deficiency: This could lead to Wernicke-Korsakoff syndrome, a serious brain disorder.  Therefore, it is crucial that patients who undergo these surgeries follow a specific diet and take vitamin and mineral supplements to prevent deficiencies. Regular medical follow-up is necessary to monitor the nutritional status and make any necessary adjustments in supplementation. This is usually done in consultation with a healthcare professional, such as a dietitian or a doctor who specializes in nutrition or bariatric surgery.  Remember, it's essential to discuss the potential risks and benefits of weight loss surgery thoroughly with your healthcare provider before deciding on this form of treatment. | 7/3/2023 | Sure, I'll try to explain that in simpler words.  Weight loss surgeries, like gastric bypass or the "sleeve", help people lose weight by changing how their stomach and intestines handle the food they eat. While these surgeries can help people lose a lot of weight, they can also lead to other problems.  One problem can be not getting enough vitamins and minerals. This is because the surgeries change the way our body absorbs nutrients from the food we eat. This can make people who've had the surgery low in important vitamins and minerals like iron, calcium, and vitamin B12, D, A, and K.  If people don't get enough of these vitamins and minerals, they might feel tired, have weak bones, or even have nerve problems. For example, not having enough iron can make someone feel very tired. Not having enough calcium can make their bones weak.  To prevent these problems, people who've had weight loss surgery usually have to eat a special diet and take extra vitamins and minerals. They also need to see their doctor regularly to check that they're getting enough nutrients from their diet.  In short, while weight loss surgeries can help with weight loss, they can also lead to other problems like not getting enough vitamins and minerals. It's important to talk to your doctor about these risks before deciding to have the surgery. |
| ASMBS | Is obesity caused by food addiction, similar to alcoholism or drug dependency? | “Food addiction” as a cause of obesity is extremely rare.  Although some people with obesity have eating disorders, such as binge eating disorder syndrome, most people have obesity caused by many factors. When treating addiction, such as alcohol and drugs, one of the first steps is to stop using drugs or alcohol. This does not work with obesity as we need to eat to live. Also, there may be other issues causing a person’s weight gain. Weight gain generally occurs when the amount of food eaten is greater than the number of calories burned. There are other conditions, however, that affect weight gain that do not involve too much eating or a less active lifestyle including:  - Poor sleeping habits  - Eating foods that may increase body fat (sugar, high fructose corn syrup, trans fat, processed meats and processed grains)  - Low intake of fat-fighting foods (fruits, vegetables, legumes, nuts, seeds, quality protein)  - Stress and mental distress  - Many types of medications  - Pollutants  Obesity also leads to more obesity, which is one of the reasons why the disease is considered progressive. Weight gain causes a number of signals (hormones) in the body that increase the risk for even greater weight gain and obesity. To make matters worse, obesity affects certain body functions that control appetite and hunger in a manner that can cause an increase in the amount of food eaten at any given meal and the desire to eat more often. There are many causes for obesity and the disease of obesity is far more than just an ‘addiction’ toward food. The treatment of obesity only as an addiction may help for a very small percentage of individuals whose only underlying cause for obesity is excessive and addictive eating, but would be unlikely to benefit most people, particularly those individuals affected by severe obesity. |  | Obesity is a complex health condition with multiple contributing factors, and its relationship to food addiction is a topic of ongoing research. Not all people who are obese are "addicted" to food, and not all people who have an addictive-like behavior toward food are obese. However, some studies suggest that a subset of individuals with obesity may experience addiction-like behaviors in response to highly palatable foods, similar in some ways to the behaviors observed in substance use disorders.  "Food addiction" as a concept stems from observations that certain foods, particularly those high in sugar, fat, and salt, can stimulate reward and pleasure centers in the brain similar to the way drugs or alcohol can. Some people might develop an unhealthy relationship with these foods, leading to patterns of compulsive overeating, strong cravings, loss of control, and continued consumption despite negative consequences. These patterns are reminiscent of the criteria used to diagnose substance use disorders.  However, it's important to note that the concept of food addiction is not universally accepted. Critics argue that unlike substances of abuse, food is necessary for survival, and "addiction" may not be the most accurate or useful way to conceptualize problematic eating behaviors. The diagnostic criteria for food addiction also differ from those for substance use disorders, and the brain responses to food and addictive substances are not identical.  Furthermore, obesity is influenced by many factors beyond food intake, including genetics, metabolism, physical activity, sleep, stress, and environmental factors. Even if food addiction plays a role for some individuals, it's unlikely to be the sole cause of obesity for most people.  So, while there can be similarities between obesity, as it pertains to overconsumption of certain types of food, and alcoholism or drug dependency, they are not equivalent and involve a host of varying factors. As of my knowledge cutoff in September 2021, the concept of food addiction is still a matter of debate within the scientific community, and more research is needed to fully understand its role in obesity. | 7/3/2023 | Sure, I'd be happy to simplify!  Obesity happens when someone gains a lot of weight in an unhealthy way. This can happen for many reasons. Some of these include eating too much, not exercising enough, not getting enough sleep, and even because of some genetic factors.  The idea of "food addiction" is that some people might eat too much because they're addicted to food, similar to how some people might drink too much alcohol because they're addicted to it. This happens because certain types of food, especially those high in sugar, fat, and salt, can make the brain feel good, just like alcohol or drugs might. So, some people might start to rely on these types of food to feel good, and might eat too much of them even when they're not hungry or when eating them is causing problems.  However, not all experts agree that "food addiction" is real, because food is something we need to survive and it doesn't affect the brain exactly the same way drugs or alcohol do. Also, not everyone who is obese is addicted to food, and not everyone who is addicted to food is obese. Even if someone is addicted to food, there might be other reasons they are obese as well.  So, while some people might become obese because they have a sort of "addiction" to food, that's not the case for everyone. And even for those people, there might be other factors involved too. |
| ASMBS | Does weight loss surgery increase the risk for alcohol abuse? | There is a small group of patients after weight loss surgery who have a problem with alcohol abuse. Most of those people, but not all, already had experiences with alcohol abuse before surgery.  Recommended precautions after weight loss surgery:  - Avoid alcoholic beverages during the rapid weight loss period, and then drink only rarely  - Be aware that even small amounts of alcohol can cause intoxication (‘being drunk’)  - Avoid driving or operating heavy equipment after drinking any alcohol  - Remember that you may feel less intoxicated than you are, and will not be a reliable judge of whether it is safe for you to drive after drinking.  - Seek help if drinking becomes a problem  If you feel drinking alcohol may be an issue for you after surgery, please contact your primary care doctor or surgeon and discuss this further. They will be able to help you identify resources available to address any alcohol-related issues.  It has been found in scientific research that a small percentage of weight loss surgery patients report having problems with alcohol after surgery (7-10%). (6) Many who abuse alcohol after surgery had problems with alcohol abuse at some time prior to surgery, but occasionally alcohol problems can arise in patients who never had such issues before their surgery. Alcohol sensitivity is increased after weight loss surgery so that the effects of alcohol are felt with fewer drinks than before surgery. Studies also find that with certain weight loss procedures (such as the gastric bypass or sleeve gastrectomy), drinking an alcoholic beverage increases blood alcohol to levels that are considerably higher than before surgery or in comparison to the alcohol levels of individuals who have not had a weight loss procedure. In addition, studies have found that alcohol takes much longer to clear out of a person’s system if they have had weight loss surgery, which can pose safety and legal hazards for driving. Furthermore, it has been found that some patients may feel as though they are sober (‘not drunk’) even when they are over the legal blood alcohol limit for driving. For all of these reasons, weight loss surgery patients are advised to take precautions regarding alcohol. |  | Some research has suggested that weight loss surgeries, like gastric bypass or sleeve gastrectomy, may be associated with an increased risk of developing alcohol use disorder after the procedure.  One theory behind this is the "addiction transfer" phenomenon. This refers to the idea that individuals who undergo bariatric surgery might transfer their food addiction to other substances such as alcohol, as they may not be able to overeat to the same extent as before.  Another factor is that the physiological changes following certain types of weight loss surgery, particularly the gastric bypass, can significantly alter the way the body processes alcohol. After surgery, alcohol is absorbed more quickly and takes longer to metabolize, which can increase its effects and potentially lead to problems with alcohol.  This does not mean that everyone who has weight loss surgery will develop an alcohol use disorder. The overall risk is thought to be relatively low, but it is significant enough to be a consideration. It's important for patients considering bariatic surgery to discuss these risks with their doctors, and for those who have had the surgery to be aware of these potential risks.  Finally, the decision to have weight loss surgery should always be made in consultation with a healthcare provider, considering all potential risks and benefits. They can provide appropriate guidance based on a person's individual health profile and circumstances. | 7/3/2023 | Sure, I can simplify that for you!  Weight loss surgery can change how your body handles food and drinks, including alcohol. Some people who have had weight loss surgery might start to drink more alcohol than before. There are a couple of reasons why this might happen:  1. Some people used to eat a lot of food to feel better. After surgery, they can't eat as much, so they might start drinking more instead. This is called "addiction transfer".  2. Weight loss surgery can change how your body processes alcohol. Alcohol might hit you harder and last longer than before the surgery. This could make it easier to drink too much and develop a problem with alcohol.  But not everyone who has weight loss surgery will start drinking too much. It's just something to keep in mind, and to talk about with your doctor if you're considering this kind of surgery. |
| ASMBS | Does weight loss surgery increase the risk for suicide? | After surgery, most patients have improved mental health but there may be a small increase in the risk of suicide. This is why patients are required to have a mental health screening before surgery.  People affected by severe obesity who are seeking weight loss surgery are more likely to suffer from depression or anxiety and to have lower self-esteem and overall quality of life than someone who is normal weight. Weight loss surgery results in significant improvement in mental health for the majority of patients. However, studies have found a small but significant increase in suicide following weight loss surgery. (7, 8) In some cases, these may have been patients with undiagnosed mental disorders, and in others, patients may have experienced major life stressors that can increase the risk for suicide, but in rare cases, the suicides are unexplained. For this reason, comprehensive bariatric programs require psychological evaluations prior to weight loss surgery. Many programs have therapists available for patient counseling after surgery. In addition, if a patient sees a psychologist or psychiatrist before surgery, they should continue seeing them after surgery. |  | As of my knowledge cutoff in September 2021, there were indeed studies suggesting that some people who undergo weight loss surgery may be at an increased risk for suicide. This may be due to various factors including the presence of pre-existing mental health conditions, difficulties in adjusting to rapid physical changes, or the experience of complications or disappointments after the surgery.  It is important to note that the link between weight loss surgery and suicide is a complex one. Many people who undergo these procedures have a significantly improved quality of life, and not all people experience mental health problems post-surgery. However, the risk does underscore the importance of comprehensive care for those undergoing weight loss procedures, including psychological support before, during, and after the surgery.  Please consult with a healthcare professional for the most up-to-date information as research continues in this field beyond 2021. Mental health is as important as physical health, and anyone considering such a major surgical procedure should ensure they have a thorough understanding of all potential risks and benefits. | 7/3/2023 | Sure, I can explain it in simpler terms.  Some studies say that people who get weight loss surgery might have a higher risk of thinking about or attempting suicide. There could be many reasons for this. It might be because they already had mental health problems before the surgery, or because they have a hard time dealing with the big changes to their body after surgery.  But, it's important to remember that this doesn't happen to everyone. Many people feel a lot better and healthier after weight loss surgery. It just means that it's really important for doctors to also check on and help with their patients' mental health before and after the surgery.  If you're thinking about getting this type of surgery, you should talk to a doctor about all the possible risks and benefits. You need to understand all parts of it, both physical and mental, before making your decision. |
| ASMBS | Is the chance of dying from weight loss surgery more than the chance of dying from obesity? | No. Weight loss surgery is very safe and decreases the chance of dying from obesity. In fact, weight loss surgery is safer than removing the gallbladder or having a knee replacement.  A recent study of 209,116 patients found the risk of death from weight loss surgery was 0.16%, or approximately 1 in 600. (4) This rate is considerably less than most other surgeries, including gallbladder and hip replacement surgery. Large studies find that the risk of death from any cause is considerably less for patients after weight loss surgery than for those who have severe obesity and have never had the surgery. Patients who have weight loss surgery have a reduction in their risk of death by 40%. Death related to diabetes is reduced by more than 90% and from heart disease by more than 50% for patients after weight loss surgery. (3) The benefits of weight loss surgery far outweigh the risks. As with any serious surgical operation, the decision to have weight loss surgery should be discussed with your surgeon, family members, and loved ones. |  | As an AI, I can't provide real-time statistics or information past my last update in September 2021. However, based on the data available to me, the risk of death from weight loss surgery (also known as bariatric surgery) is generally low, and is often less than the risk of death from severe obesity itself. The death rate from bariatric surgery is often quoted as about 0.1%, which is similar to other major surgeries.  Obesity can lead to a variety of health problems, including heart disease, diabetes, high blood pressure, certain types of cancer, and more. These conditions can significantly increase the risk of premature death. The relative risk can vary depending on the severity of the obesity, the individual's overall health, and other factors.  It's important to remember that while bariatric surgery can be a useful tool for weight loss, it's not without its risks. These can include immediate postoperative complications, long-term nutritional deficiencies, and the need for further surgery, among others. Therefore, it's usually considered when other weight loss attempts have failed and the person's health is at significant risk from obesity.  For anyone considering bariatric surgery, a discussion with a healthcare professional is important to fully understand the potential benefits and risks. They can provide up-to-date and personalized advice based on a comprehensive assessment of the individual's health. | 7/3/2023 | Sure, I'd be happy to simplify!  Weight loss surgery (like a gastric bypass or gastric sleeve) can have risks, just like any major operation. However, these risks are typically very low. In fact, the chance of dying from weight loss surgery is about the same as from other major surgeries.  On the other hand, being very overweight can cause lots of health problems, like heart disease or diabetes. These can be very serious and increase the chance of dying early.  In many cases, the risks from being very overweight are higher than the risks from weight loss surgery. That's why doctors might recommend this surgery for people who are very overweight and haven't been able to lose weight in other ways.  But it's important to remember that everyone is different. If you or someone else is thinking about weight loss surgery, you should talk to a doctor about it. They can explain the possible benefits and risks in more detail, considering the person's specific health situation. |
