# Supplementary material for: Large language models and bariatric surgery patient education: a comparative readability analysis of GPT-3.5, GPT-4, Bard, and online institutional resources
Source: Surg Endosc. 2024 Mar 12;38(5):2522–32. doi: 10.1007/s00464-024-10720-2 (PMC11078810; doi:10.1007/s00464-024-10720-2)
Supplement: Supplementary file 4 — Supplementary file4 (DOCX 121 KB) [file 464_2024_10720_MOESM4_ESM.docx]

**Supplementary Table 4**. Bard Responses

| Institution | Question | Institutional Answer | Modified Question | Bard Answer | Date of Bard Entry (Month/Day/Year) | Can you explain that in simpler terms? |
| --- | --- | --- | --- | --- | --- | --- |
| 1 | Why should I consider weight loss surgery? | Weight loss surgery is the most effective way to lose weight and keep weight off. Weight loss surgery can improve or resolve obesity-related conditions such as diabetes, high blood pressure, acid reflux and sleep apnea. It also decreases obesity-related cancer risk by 30%. Weight loss surgery can help improve your overall quality of life. |  | Weight loss surgery is a major decision, and it's important to weigh the pros and cons carefully before making a decision. Here are some of the reasons why you might consider weight loss surgery:  * **To improve your health.** Weight loss surgery can help you lose a significant amount of weight, which can lead to improvements in your health. For example, weight loss surgery can help you lower your blood pressure, cholesterol, and blood sugar levels. It can also help you reduce your risk of developing type 2 diabetes, heart disease, stroke, and some types of cancer.  * **To improve your quality of life.** Weight loss surgery can also help you improve your quality of life. For example, it can help you have more energy, sleep better, and feel better about yourself. It can also help you participate in activities that you may have been unable to do before, such as hiking, biking, or dancing.  * **To achieve your weight loss goals.** If you have tried to lose weight through diet and exercise without success, weight loss surgery may be an option for you. Weight loss surgery can help you lose weight more quickly and easily than you would be able to on your own.  Of course, there are also some risks associated with weight loss surgery. These risks include:  * **Complications during surgery.** There is always a risk of complications during surgery, even if it is a routine procedure. Some of the possible complications of weight loss surgery include bleeding, infection, and blood clots.  * **Long-term complications.** There are also some long-term complications that can occur after weight loss surgery. These complications can include nutritional deficiencies, gallstones, and dumping syndrome.  It's important to talk to your doctor about the risks and benefits of weight loss surgery before making a decision. Your doctor can help you determine if weight loss surgery is right for you and can help you choose the best type of surgery for your individual needs.  Here are some additional things to consider when deciding whether or not to have weight loss surgery:  * Your age and overall health.  * Your motivation to lose weight and keep it off.  * Your support system.  * Your financial resources.  If you are considering weight loss surgery, it is important to do your research and talk to your doctor about all of your options. Weight loss surgery is a major decision, but it can be a life-changing one for the right person. | 7/8/2023 | Sure. Weight loss surgery is a medical procedure that can help you lose a significant amount of weight. It is usually done for people who are severely obese and have tried other methods of weight loss without success.  There are many different types of weight loss surgery, but they all work by changing the way your body digests food. Some surgeries make your stomach smaller, so you feel full after eating less food. Other surgeries reroute your digestive system so that you absorb fewer calories.  Weight loss surgery can be a very effective way to lose weight. In most cases, people who have weight loss surgery lose about half of their excess weight within two years. This can lead to significant improvements in your health, such as lower blood pressure, cholesterol, and blood sugar levels. It can also help you reduce your risk of developing type 2 diabetes, heart disease, stroke, and some types of cancer.  However, weight loss surgery is not without risks. Some of the possible complications of weight loss surgery include bleeding, infection, and blood clots. There are also some long-term complications that can occur, such as nutritional deficiencies, gallstones, and dumping syndrome.  It is important to talk to your doctor about the risks and benefits of weight loss surgery before making a decision. Your doctor can help you determine if weight loss surgery is right for you and can help you choose the best type of surgery for your individual needs.  Here are some things to consider when deciding whether or not to have weight loss surgery:  * Your age and overall health.  * Your motivation to lose weight and keep it off.  * Your support system.  * Your financial resources.  If you are considering weight loss surgery, it is important to do your research and talk to your doctor about all of your options. Weight loss surgery is a major decision, but it can be a life-changing one for the right person.  In simpler terms, weight loss surgery can help you lose weight and improve your health. However, it is important to talk to your doctor about the risks and benefits before making a decision. |
| 1 | What are my options for weight loss surgery at X? | Our surgeons offer Roux-en-Y gastric bypass, sleeve gastrectomy and the laparoscopic adjustable gastric band. We also offer revision surgery for patients who have had weight loss surgery before and have not achieved desired results. |  | X offers three main types of weight loss surgery:  * **Roux-en-Y gastric bypass** (RYGB) is the most common type of weight loss surgery in the United States. It involves creating a small pouch at the top of the stomach and then connecting it to the small intestine, bypassing a large portion of the stomach and small intestine. This makes you feel full faster and less hungry, which helps you lose weight.  * **Gastric sleeve** (GS) is a newer type of weight loss surgery that involves removing about 80% of the stomach. This leaves you with a small, banana-shaped pouch that can hold less food. As with RYGB, this makes you feel full faster and less hungry, which helps you lose weight.  * **Laparoscopic adjustable gastric band** (LAGB) is a less invasive type of weight loss surgery that involves placing a band around the upper part of the stomach. The band can be tightened or loosened to control how much food you can eat. LAGB is not as effective as RYGB or GS, but it is a good option for people who are not good candidates for the more invasive procedures.  In addition to these three main types of weight loss surgery, X also offers revision surgery for people who have had weight loss surgery before and have not achieved desired results.  The best type of weight loss surgery for you will depend on your individual needs and circumstances. Your doctor will discuss all of your options with you and help you choose the best one for you.  Here is a table that summarizes the three main types of weight loss surgery offered at X:  \| Type of Surgery \| Description \| Advantages \| Disadvantages \|  \|---\|---\|---\|---\|  \| Roux-en-Y gastric bypass \| A small pouch is created at the top of the stomach and then connected to the small intestine, bypassing a large portion of the stomach and small intestine. \| This is the most effective type of weight loss surgery, and it can help you lose a significant amount of weight. \| It is a more invasive surgery than the other two options, and it carries a slightly higher risk of complications. \|  \| Gastric sleeve \| About 80% of the stomach is removed, leaving a small, banana-shaped pouch that can hold less food. \| This is a less invasive surgery than RYGB, and it carries a lower risk of complications. \| It is not as effective as RYGB, and you may not lose as much weight. \|  \| Laparoscopic adjustable gastric band \| A band is placed around the upper part of the stomach to control how much food you can eat. \| This is the least invasive type of weight loss surgery, and it carries the lowest risk of complications. \| It is not as effective as RYGB or GS, and you may not lose as much weight. \|  I hope this helps! Let me know if you have any other questions. | 7/8/2023 | Sure. There are three main types of weight loss surgery at X:  * **Roux-en-Y gastric bypass:** This is the most common type of weight loss surgery. It works by making your stomach smaller and bypassing part of your small intestine. This makes you feel full faster and helps you eat less food.  * **Gastric sleeve:** This surgery removes about 80% of your stomach. This leaves you with a smaller stomach that can hold less food. This also helps you feel full faster and eat less food.  * **Laparoscopic adjustable gastric band:** This surgery places a band around your stomach. The band can be tightened or loosened to control how much food you can eat. This helps you feel full faster and eat less food.  The best type of weight loss surgery for you will depend on your individual needs and circumstances. Your doctor will discuss all of your options with you and help you choose the best one for you.  In general, Roux-en-Y gastric bypass is the most effective type of weight loss surgery. It can help you lose a significant amount of weight. However, it is also the most invasive type of surgery. Gastric sleeve is less invasive than Roux-en-Y gastric bypass, but it is not as effective. Laparoscopic adjustable gastric band is the least invasive type of weight loss surgery, but it is also the least effective.  If you are considering weight loss surgery, it is important to talk to your doctor about all of your options. They can help you choose the best type of surgery for you and make sure that you are a good candidate for surgery. |
| 1 | Who is eligible for weight loss surgery? | To qualify for weight loss surgery, you must have either a:  - BMI of 40 or greater  - BMI of 35-40 with obesity-related conditions such as diabetes, high blood pressure, sleep apnea or gastroesophageal reflux disease (GERD) |  | The eligibility criteria for weight loss surgery vary depending on the specific procedure and the insurance company. However, there are some general guidelines that most surgeons follow.  * **Body mass index (BMI)**. A BMI of 40 or higher is considered morbid obesity, and this is typically the minimum BMI requirement for weight loss surgery. However, some surgeons may consider surgery for people with a BMI of 35 or higher if they have significant obesity-related health problems.  * **Obesity-related health problems**. Weight loss surgery can help to improve or even cure many obesity-related health problems, such as type 2 diabetes, high blood pressure, sleep apnea, and heart disease. If you have one or more of these conditions, you may be eligible for weight loss surgery even if your BMI is not as high as 40.  * **Psychological evaluation**. Weight loss surgery is a major surgery, and it is important to make sure that you are mentally prepared for the changes that it will bring. Your surgeon will want to see that you are committed to making lifestyle changes, such as eating a healthy diet and exercising regularly.  * **Lifestyle changes**. You will need to have made significant lifestyle changes before you are eligible for weight loss surgery. This typically means that you have tried to lose weight through diet and exercise for at least 6 months without success.  In addition to these general guidelines, there may be other factors that affect your eligibility for weight loss surgery. These factors may include your age, your overall health, and your insurance coverage.  If you are considering weight loss surgery, it is important to talk to your doctor about your eligibility. They can help you to determine if surgery is right for you and can refer you to a surgeon who specializes in bariatric surgery. | 7/8/2023 | Sure. Weight loss surgery is a major surgery that can help you lose a lot of weight. But not everyone is eligible for it. Here are some of the things that surgeons look at to decide if you are eligible:  * **Your BMI**. BMI is a measure of your body fat. A BMI of 40 or higher is considered morbid obesity, and this is typically the minimum BMI requirement for weight loss surgery.  * **Your health**. If you have obesity-related health problems, such as type 2 diabetes, high blood pressure, sleep apnea, or heart disease, you may be eligible for weight loss surgery even if your BMI is not as high as 40.  * **Your mental health**. Weight loss surgery is a major life change, and it is important to make sure that you are mentally prepared for it. Your surgeon will want to see that you are committed to making lifestyle changes, such as eating a healthy diet and exercising regularly.  * **Your lifestyle**. You will need to have made significant lifestyle changes before you are eligible for weight loss surgery. This typically means that you have tried to lose weight through diet and exercise for at least 6 months without success.  If you are considering weight loss surgery, it is important to talk to your doctor about your eligibility. They can help you to determine if surgery is right for you and can refer you to a surgeon who specializes in bariatric surgery.  In simpler terms, you are eligible for weight loss surgery if you are:  * Morbidly obese (BMI of 40 or higher)  * Have obesity-related health problems  * Mentally prepared for surgery and lifestyle changes  * Have tried to lose weight through diet and exercise for at least 6 months without success  If you meet these criteria, you may be eligible for weight loss surgery. However, it is important to talk to your doctor to get a personalized assessment. |
| 1 | What is the recovery time after weight loss surgery? | On average, most patients stay in the hospital one to two days after surgery. Most return to work two to three weeks after surgery. |  | The recovery time after weight loss surgery varies depending on the type of surgery you have. Most people stay in the hospital for 1-3 days after surgery, and then they can go home. However, it takes several weeks to fully recover from weight loss surgery.  Here is a general overview of the recovery time for different types of weight loss surgery:  * **Gastric sleeve:** 2-3 days in the hospital, 4-6 weeks for full recovery.  * **Gastric bypass:** 1-3 days in the hospital, 4-6 weeks for full recovery.  * **Gastric banding:** Up to 1 day in the hospital, 4-6 weeks for full recovery.  During the recovery period, you will need to take it easy and avoid strenuous activity. You will also need to follow a strict diet and medication regimen. Your doctor will give you specific instructions on how to care for yourself after surgery.  Here are some tips for a smooth recovery after weight loss surgery:  * Get plenty of rest.  * Drink plenty of fluids.  * Eat a healthy diet.  * Take your medications as prescribed.  * Listen to your body and rest when you need to.  With proper care, most people make a full recovery from weight loss surgery within a few weeks. However, it is important to follow your doctor's instructions carefully and to make lifestyle changes to help you maintain your weight loss. | 7/8/2023 | Sure. Here is a simpler explanation of the recovery time after weight loss surgery:  * **How long will I be in the hospital?** This depends on the type of surgery you have. Most people stay in the hospital for 1-3 days after surgery.  * **How long will it take me to recover fully?** It takes several weeks to fully recover from weight loss surgery. Most people can return to work in 2-3 weeks, but it may take up to 6 weeks to feel back to normal.  * **What should I expect during the recovery period?** You will need to take it easy and avoid strenuous activity. You will also need to follow a strict diet and medication regimen. Your doctor will give you specific instructions on how to care for yourself after surgery.  Here are some tips for a smooth recovery after weight loss surgery:  * **Get plenty of rest.** This is the most important thing you can do to help your body heal.  * **Drink plenty of fluids.** This will help prevent dehydration and constipation.  * **Eat a healthy diet.** This will help you get the nutrients you need while your body is healing.  * **Take your medications as prescribed.** This will help prevent infection and other complications.  * **Listen to your body and rest when you need to.** Don't push yourself too hard during the recovery period.  With proper care, most people make a full recovery from weight loss surgery within a few weeks. However, it is important to follow your doctor's instructions carefully and to make lifestyle changes to help you maintain your weight loss.  I hope this explanation is simpler to understand. Please let me know if you have any other questions. |
| 1 | How much weight can I expect to lose after surgery and how quickly? | Maximum weight loss typically occurs 12-24 months after surgery. Individual weight loss varies depending on:  - Age  - Weight before surgery  - Level of physical activity  - Diet  - Type of surgery | How much weight can I expect to lose after weight loss surgery and how quickly? | The amount of weight you can expect to lose after weight loss surgery varies depending on the type of surgery you have, your starting weight, and your commitment to making lifestyle changes.  In general, people who have weight loss surgery lose about 50-75% of their excess weight within the first two years. This means that if you weigh 200 pounds and have 100 pounds of excess weight, you could expect to lose 75 pounds within two years.  The weight loss will not be linear, however. You will likely lose the most weight in the first few months after surgery, and then the weight loss will slow down. It is important to be patient and to continue making lifestyle changes, such as eating a healthy diet and exercising regularly, in order to maintain your weight loss.  Here is a breakdown of the average weight loss after different types of weight loss surgery:  * **Gastric bypass:** 60-75% of excess weight loss  * **Sleeve gastrectomy:** 50-60% of excess weight loss  * **Gastric banding:** 30-50% of excess weight loss  It is important to note that these are just averages, and your individual results may vary. Talk to your doctor about your specific expectations for weight loss after surgery.  Here are some tips for maximizing your weight loss after surgery:  * Follow your doctor's instructions carefully.  * Eat a healthy diet that is low in calories and fat.  * Exercise regularly.  * Join a support group.  * Be patient and persistent.  With hard work and dedication, you can achieve significant weight loss after weight loss surgery. | 7/8/2023 | Sure. Here is a simpler explanation of how much weight you can expect to lose after weight loss surgery:  * **In the first few months:** You will likely lose the most weight in the first few months after surgery. This is because your body is adjusting to the surgery and you are making lifestyle changes, such as eating a healthy diet and exercising regularly.  * **After 6 months:** The weight loss will slow down after the first few months. However, you can still expect to lose weight at a steady pace.  * **After 2 years:** Within two years, you can expect to lose about 50-75% of your excess weight. This means that if you weigh 200 pounds and have 100 pounds of excess weight, you could expect to lose 75 pounds within two years.  It is important to remember that these are just averages and your individual results may vary. Talk to your doctor about your specific expectations for weight loss after surgery.  Here are some tips for maximizing your weight loss after surgery:  * **Follow your doctor's instructions carefully.** This includes taking your medications as prescribed and following a healthy diet.  * **Eat a healthy diet that is low in calories and fat.** This will help you lose weight and keep it off.  * **Exercise regularly.** Exercise is an important part of any weight loss plan. It helps you burn calories and build muscle.  * **Join a support group.** Talking to other people who have had weight loss surgery can be helpful and motivating.  * **Be patient and persistent.** Weight loss takes time and effort. Don't give up if you don't see results immediately.  With hard work and dedication, you can achieve significant weight loss after weight loss surgery. |
|  | What should I expect after surgery? | You will need to follow a bariatric diet and exercise daily, take bariatric vitamins lifelong, refrain from using nicotine (including cigarettes) and minimize alcohol use. | What should I expect after weight loss surgery? | The specific experiences of each person after weight loss surgery will vary, but there are some general things to expect.  * **Weight loss:** Most people lose a significant amount of weight after weight loss surgery. The amount of weight you lose will depend on your individual circumstances, but you can expect to lose anywhere from 40% to 80% of your excess weight.  * **Improved health:** Weight loss surgery can also lead to improvements in your overall health. This is because obesity can contribute to a number of health conditions, such as type 2 diabetes, high blood pressure, and sleep apnea. After weight loss surgery, these conditions can often improve or even go into remission.  * **Lifestyle changes:** Weight loss surgery is not a magic bullet. You will still need to make lifestyle changes, such as eating a healthy diet and exercising regularly, in order to maintain your weight loss.  * **Side effects:** There are some potential side effects associated with weight loss surgery. These can include nausea, vomiting, constipation, and dumping syndrome. These side effects are usually mild and go away on their own. However, in some cases, they can be more serious.  * **Risks:** Weight loss surgery is a major surgery, so there are some risks involved. These risks can include infection, bleeding, and complications with anesthesia. However, the risks of weight loss surgery are generally outweighed by the benefits.  It is important to talk to your doctor about your expectations after weight loss surgery. They can help you understand what to expect and make sure that you are a good candidate for surgery.  Here is a more detailed overview of what you can expect after weight loss surgery:  * **The first few days:** You will likely stay in the hospital for a few days after surgery. During this time, you will be monitored closely for any complications. You will also start to learn about your new diet and how to manage your weight loss.  * **The first few weeks:** You will need to take it easy during the first few weeks after surgery. You will not be able to eat solid foods for a few days, and you will need to drink plenty of fluids. You may also experience some nausea and vomiting.  * **The first few months:** You will start to lose weight rapidly in the first few months after surgery. You will need to make sure that you are eating a healthy diet and getting enough exercise. You may also experience some side effects, such as dumping syndrome.  * **The long term:** Most people are able to maintain their weight loss after weight loss surgery. However, it is important to continue to make healthy lifestyle choices. You will also need to have regular check-ups with your doctor to monitor your health.  Weight loss surgery can be a life-changing procedure. It can help you lose weight, improve your health, and extend your life. However, it is important to remember that weight loss surgery is not a magic bullet. You will still need to make lifestyle changes in order to maintain your weight loss. | 7/8/2023 | Sure. Here's a simpler explanation of what to expect after weight loss surgery:  * **You will lose a lot of weight.** How much weight you lose will depend on your individual circumstances, but most people lose anywhere from 40% to 80% of their excess weight.  * **Your health will improve.** Weight loss surgery can help improve a number of health conditions, such as type 2 diabetes, high blood pressure, and sleep apnea.  * **You will need to make lifestyle changes.** Weight loss surgery is not a magic bullet. You will still need to eat a healthy diet and exercise regularly in order to maintain your weight loss.  * **There are some potential side effects.** The most common side effects are nausea, vomiting, constipation, and dumping syndrome. These side effects are usually mild and go away on their own.  * **There are some risks involved.** Weight loss surgery is a major surgery, so there are some risks involved. These risks can include infection, bleeding, and complications with anesthesia. However, the risks of weight loss surgery are generally outweighed by the benefits.  In general, weight loss surgery can be a life-changing procedure. It can help you lose weight, improve your health, and extend your life. But it's important to remember that weight loss surgery is not a magic bullet. You will still need to make lifestyle changes in order to maintain your weight loss.  Here are some additional things to keep in mind after weight loss surgery:  * You will need to stay in the hospital for a few days after surgery.  * You will need to take it easy for the first few weeks after surgery.  * You will not be able to eat solid foods for a few days after surgery.  * You will need to drink plenty of fluids.  * You may experience some nausea and vomiting.  * You will need to make sure that you are eating a healthy diet and getting enough exercise.  * You may experience some side effects, such as dumping syndrome.  * You will need to have regular check-ups with your doctor to monitor your health.  If you are considering weight loss surgery, it is important to talk to your doctor about your expectations. They can help you understand what to expect and make sure that you are a good candidate for surgery. |
|  | What is body mass index (BMI), and how can I calculate mine? | Body mass index (BMI) is a tool to screen for obesity, factoring in your height and weight. Learn your BMI with our BMI calculator. |  | Body mass index (BMI) is a measure of body fat based on height and weight that applies to adult men and women. BMI is calculated by dividing a person's weight in kilograms by their height in meters squared.  The formula for BMI is: BMI = weight in kilograms / (height in meters)^2  For example, if a person weighs 70 kilograms and is 1.7 meters tall, their BMI would be 24.22.  The World Health Organization (WHO) uses the following BMI categories to classify adults:  * Underweight: BMI < 18.5  * Normal weight: BMI 18.5-24.9  * Overweight: BMI 25.0-29.9  * Obese Class I: BMI 30.0-34.9  * Obese Class II: BMI 35.0-39.9  * Obese Class III: BMI ≥ 40.0  It is important to note that BMI is just a screening tool and does not take into account body composition, which is the ratio of fat to muscle mass. People with a high BMI but a low body fat percentage may be classified as overweight or obese, even though they are not at increased risk for health problems.  There are many online BMI calculators available, such as the one at the CDC website. You can also calculate your BMI by hand using the formula above.  If you are concerned about your BMI, it is important to talk to your doctor. They can help you assess your overall health and determine if you need to make any changes to your weight. | 7/8/2023 | Sure. BMI is a measure of how much body fat you have based on your height and weight. It is not a perfect measure, but it is a good way to get a general idea of your weight category.  To calculate your BMI, you need to know your weight in kilograms and your height in meters. You can then use the following formula: BMI = weight in kilograms / (height in meters)^2  For example, if you weigh 70 kilograms and are 1.7 meters tall, your BMI would be 24.22.  The World Health Organization (WHO) uses the following BMI categories to classify adults:  * Underweight: BMI < 18.5  * Normal weight: BMI 18.5-24.9  * Overweight: BMI 25.0-29.9  * Obese Class I: BMI 30.0-34.9  * Obese Class II: BMI 35.0-39.9  * Obese Class III: BMI ≥ 40.0  A BMI of 18.5 or lower is considered underweight. A BMI of 25 or higher is considered overweight. A BMI of 30 or higher is considered obese.  It is important to note that BMI is just a screening tool and does not take into account body composition, which is the ratio of fat to muscle mass. People with a high BMI but a low body fat percentage may be classified as overweight or obese, even though they are not at increased risk for health problems.  If you are concerned about your BMI, it is important to talk to your doctor. They can help you assess your overall health and determine if you need to make any changes to your weight.  In simpler terms, BMI is a number that tells you how much body fat you have based on your height and weight. A high BMI can be a sign of overweight or obesity, which are risk factors for certain health conditions. However, BMI is not a perfect measure, so it is important to talk to your doctor if you are concerned about your weight. |
| 2 | Why should someone consider having bariatric surgery? | Obesity is harmful to a person’s health. Someone who is 40 percent overweight is twice more likely to die prematurely than an average-weight person. (This effect is seen after 10 to 30 years of being obese.) In this country alone, about 300,000 deaths per year can be blamed on obesity. Obesity is a risk factor for many serious, life-threatening diseases, including:  - Diabetes.  - Cancer.  - High blood pressure (hypertension).  - Heart disease.  - Obstructive sleep apnea.  - Stroke.  Studies show that the risk of death from these conditions returns to normal after weight loss. Within the first six months of having weight loss surgery, patients usually no longer need to take medications for these conditions. In addition, women who were struggling with infertility before surgery find that conception is possible after surgery. |  | Bariatric surgery is a major medical procedure that should only be considered after careful consideration and discussion with your doctor. However, there are many potential benefits to having bariatric surgery, including:  * **Long-term weight loss.** Bariatric surgery can help you lose a significant amount of weight, and most people who have surgery are able to keep the weight off long-term.  * **Improved health.** Bariatric surgery can improve a number of health conditions that are often associated with obesity, such as type 2 diabetes, high blood pressure, sleep apnea, and heart disease.  * **Improved quality of life.** Bariatric surgery can have a significant impact on your quality of life, by improving your physical health, mental health, and overall well-being.  Of course, there are also risks associated with bariatric surgery, so it is important to weigh the benefits and risks carefully before making a decision. Some of the potential risks of bariatric surgery include:  * **Complications during surgery.** Any surgery carries some risk of complications, and bariatric surgery is no exception. Some of the potential complications of bariatric surgery include bleeding, infection, and injury to internal organs.  * **Long-term side effects.** Some people who have bariatric surgery experience long-term side effects, such as dumping syndrome, nutritional deficiencies, and gallstones.  If you are considering bariatric surgery, it is important to talk to your doctor about the risks and benefits of the procedure. Your doctor can help you decide if bariatric surgery is right for you.  Here are some additional things to consider when thinking about bariatric surgery:  * Your age and overall health. Bariatric surgery is generally not recommended for people who are under 18 years old or who have significant health problems.  * Your willingness to make lifestyle changes. Bariatric surgery is not a magic bullet for weight loss. You will still need to make changes to your diet and exercise habits in order to keep the weight off long-term.  * Your support system. Bariatric surgery is a major life change, and it is important to have a strong support system in place to help you through the process.  If you are considering bariatric surgery, I encourage you to do your research and talk to your doctor about the best option for you. | 7/8/2023 | Sure. Bariatric surgery is a type of weight loss surgery that can help you lose a significant amount of weight and improve your health. However, it is a major surgery with some risks, so it is important to talk to your doctor about the benefits and risks before making a decision.  Here are some of the benefits of bariatric surgery:  * You can lose a significant amount of weight.  * You can improve your health by reducing your risk of type 2 diabetes, high blood pressure, sleep apnea, and heart disease.  * You can improve your quality of life by improving your physical health, mental health, and overall well-being.  Here are some of the risks of bariatric surgery:  * There is a risk of complications during surgery, such as bleeding, infection, and injury to internal organs.  * There is a risk of long-term side effects, such as dumping syndrome, nutritional deficiencies, and gallstones.  If you are considering bariatric surgery, it is important to talk to your doctor about the risks and benefits of the procedure. Your doctor can help you decide if bariatric surgery is right for you.  Here are some additional things to consider when thinking about bariatric surgery:  * Your age and overall health. Bariatric surgery is generally not recommended for people who are under 18 years old or who have significant health problems.  * Your willingness to make lifestyle changes. Bariatric surgery is not a magic bullet for weight loss. You will still need to make changes to your diet and exercise habits in order to keep the weight off long-term.  * Your support system. Bariatric surgery is a major life change, and it is important to have a strong support system in place to help you through the process.  I hope this helps! |
| 2 | Who is eligible for bariatric surgery? | At X, we carefully evaluate all patients being considered for bariatric surgery. Nutritional and behavioral counseling are conducted, and patients are required to attend at least one weekly support group session prior to weight loss surgery.  To be eligible for bariatric surgery, you must be between 16 and 70 years of age (with some exceptions) and morbidly obese (weighing at least 100 pounds over your ideal body weight and having a BMI of 40). A BMI between 35 and 39 also may qualify you for the procedure if you also have certain pre-existing co-morbidities, such as diabetes, cardiovascular disease, hypertension, or sleep apnea.  If you are a woman of childbearing age and planning a pregnancy, it’s vital to know that you must not get pregnant within the first 18 months to two years following surgery. The rapid weight loss and nutritional deficiencies associated with bariatric surgery make pregnancy very dangerous for you and for a developing fetus.  A consultation with a X bariatric surgeon is a mandatory step prior to surgery, and will help you screen for eligibility. |  | The eligibility criteria for bariatric surgery vary depending on the specific procedure and the individual's health status. However, there are some general guidelines that most surgeons follow.  * **Body mass index (BMI)**. A BMI of 40 or higher is considered to be morbidly obese, and this is typically the minimum BMI requirement for bariatric surgery. However, some surgeons may consider surgery for people with a BMI of 35 or higher if they have significant obesity-related health problems.  * **Comorbidities**. Bariatric surgery can be a very effective way to treat obesity-related health problems, such as type 2 diabetes, high blood pressure, sleep apnea, and heart disease. If you have any of these conditions, your surgeon will likely consider you to be a good candidate for surgery.  * **Age**. Most surgeons will only consider bariatric surgery for people who are between the ages of 18 and 70. However, there are some exceptions, and some surgeons may consider surgery for younger or older adults.  * **Mental health**. Bariatric surgery is a major surgery, and it is important to be mentally prepared for the changes that will come with it. Your surgeon will want to make sure that you are emotionally stable and that you have a good support system in place.  * **Lifestyle**. Bariatric surgery is not a magic bullet for weight loss. You will still need to make lifestyle changes, such as eating a healthy diet and exercising regularly. Your surgeon will want to make sure that you are committed to making these changes.  If you think that you may be a candidate for bariatric surgery, it is important to talk to your doctor. They can assess your individual health status and determine if surgery is right for you. | 7/8/2023 | Sure. Bariatric surgery is a type of weight loss surgery that can help people who are severely obese lose weight and improve their health. To be eligible for bariatric surgery, you typically need to have a BMI of 40 or higher, or a BMI of 35 or higher with significant obesity-related health problems. You also need to be between the ages of 18 and 70, and you need to be mentally prepared for the changes that will come with surgery.  In addition to these general guidelines, there are other factors that your surgeon will consider when determining if you are a good candidate for bariatric surgery. These factors may include your medical history, your lifestyle habits, and your support system.  If you think that you may be a candidate for bariatric surgery, it is important to talk to your doctor. They can assess your individual health status and determine if surgery is right for you.  Here is a table that summarizes the eligibility criteria for bariatric surgery:  \| Criteria \| Requirements \|  \|---\|---\|  \| BMI \| 40 or higher, or 35 or higher with significant obesity-related health problems \|  \| Age \| 18-70 \|  \| Mental health \| Stable and have a good support system \|  \| Lifestyle \| Willing to make lifestyle changes, such as eating a healthy diet and exercising regularly \|  I hope this helps! |
| 2 | What is the recovery time following weight loss surgery? | X's medical team has an immense wealth of experience in bariatric surgery, this combined with advances in the field of bariatric care, means that most patients recover from surgery quickly and without complications. On average the hospital stay for patients who undergo the Roux-en-Y gastric bypass is usually two to four days. |  | The recovery time following weight loss surgery varies depending on the type of surgery you have. In general, most patients stay in the hospital for 1-3 days after surgery. After that, you will need to rest at home and gradually resume your normal activities.  Here is a general overview of the recovery time for different types of weight loss surgery:  * **Gastric sleeve surgery:** This is the most common type of weight loss surgery. Recovery time is typically 4-6 weeks.  * **Gastric bypass surgery:** This surgery involves rerouting your stomach and small intestine. Recovery time is typically 6-8 weeks.  * **Gastric banding surgery:** This surgery involves placing a band around your stomach to restrict how much food you can eat. Recovery time is typically 2-4 weeks.  It is important to follow your doctor's instructions carefully during your recovery period. This will help you to heal properly and avoid complications.  Here are some general tips for recovering from weight loss surgery:  * **Get plenty of rest.** You will need to take it easy during your recovery period. Avoid strenuous activity and lifting heavy objects.  * **Drink plenty of fluids.** This will help to prevent dehydration.  * **Eat a healthy diet.** Your doctor will give you specific instructions about what to eat after surgery.  * **Take your medications as prescribed.** This will help to reduce pain and inflammation.  * **See your doctor for regular checkups.** This will help to ensure that your recovery is going well.  With proper care, most people make a full recovery from weight loss surgery within a few weeks. However, it is important to remember that weight loss surgery is not a magic bullet. You will still need to make lifestyle changes, such as eating a healthy diet and exercising regularly, in order to maintain your weight loss. | 7/8/2023 | Sure. Here is a simpler explanation of the recovery time following weight loss surgery:  * **How long will I be in the hospital?** Most people stay in the hospital for 1-3 days after weight loss surgery.  * **How long will it take me to recover?** The recovery time varies depending on the type of surgery you have. Typically, it takes 4-6 weeks to recover from gastric sleeve surgery, 6-8 weeks to recover from gastric bypass surgery, and 2-4 weeks to recover from gastric banding surgery.  * **What should I do to recover?** You will need to rest at home and gradually resume your normal activities. It is important to follow your doctor's instructions carefully during your recovery period.  * **What are some tips for recovering?** Here are some general tips for recovering from weight loss surgery:  * Get plenty of rest.  * Drink plenty of fluids.  * Eat a healthy diet.  * Take your medications as prescribed.  * See your doctor for regular checkups.  With proper care, most people make a full recovery from weight loss surgery within a few weeks. However, it is important to remember that weight loss surgery is not a magic bullet. You will still need to make lifestyle changes, such as eating a healthy diet and exercising regularly, in order to maintain your weight loss.  I hope this helps! Let me know if you have any other questions. |
| 2 | How much weight loss can be expected? | Most patients will lose between 66 and 80 percent of their excess body weight, most of which is lost 18 to 24 months following bariatric surgery. | How much weight loss can be expected following weight loss surgery? | The amount of weight loss that can be expected following weight loss surgery varies depending on the type of surgery, the patient's starting weight, and their individual response to the surgery. However, in general, people can expect to lose a significant amount of weight after weight loss surgery.  * **Sleeve gastrectomy:** This surgery is the most common type of weight loss surgery in the United States. It involves removing about 75% of the stomach, which creates a smaller, banana-shaped stomach. People who have sleeve gastrectomy can expect to lose about 60-75% of their excess weight over the course of 5 years.  * **Gastric bypass:** This surgery is more invasive than sleeve gastrectomy, but it can also lead to greater weight loss. In gastric bypass, the surgeon creates a small pouch at the top of the stomach and then connects it directly to the small intestine, bypassing most of the stomach and the first part of the small intestine. People who have gastric bypass can expect to lose about 70-80% of their excess weight over the course of 5 years.  * **Gastric banding:** This surgery involves placing a band around the top of the stomach to create a small pouch. The band can be tightened or loosened over time to control the amount of food that the person can eat. Gastric banding is not as effective as sleeve gastrectomy or gastric bypass, and it is not as commonly performed. People who have gastric banding can expect to lose about 50-60% of their excess weight over the course of 5 years.  It is important to note that weight loss surgery is not a magic bullet. It requires a lifelong commitment to healthy eating and exercise. However, for people who are severely obese, weight loss surgery can be a life-saving procedure. It can help to improve their health, reduce their risk of chronic diseases, and improve their quality of life.  Here are some additional factors that can affect the amount of weight loss that can be expected after weight loss surgery:  * **Age:** Younger people tend to lose more weight than older people after weight loss surgery.  * **Gender:** Women tend to lose more weight than men after weight loss surgery.  * **Starting weight:** The more overweight or obese a person is, the more weight they are likely to lose after weight loss surgery.  * **Comorbidities:** People who have other health conditions, such as diabetes or heart disease, may lose more weight than people who do not have these conditions.  If you are considering weight loss surgery, it is important to talk to your doctor about your individual expectations and goals. They can help you to determine if weight loss surgery is the right option for you and can help you to develop a personalized weight loss plan. | 7/8/2023 | Sure. Weight loss surgery can help you lose a lot of weight. How much weight you lose depends on the type of surgery you have and your individual response to the surgery. But in general, people can expect to lose about 60-80% of their excess weight over the course of 5 years.  For example, if you weigh 300 pounds and have sleeve gastrectomy, you can expect to lose about 180-240 pounds over the course of 5 years. If you weigh 400 pounds and have gastric bypass, you can expect to lose about 280-320 pounds over the course of 5 years.  It is important to note that weight loss surgery is not a magic bullet. It requires a lifelong commitment to healthy eating and exercise. However, for people who are severely obese, weight loss surgery can be a life-saving procedure. It can help to improve their health, reduce their risk of chronic diseases, and improve their quality of life.  Here are some simplified terms to help you understand the information:  * **Excess weight:** This is the amount of weight that is above your ideal weight.  * **Sleeve gastrectomy:** This is a surgery that removes about 75% of the stomach.  * **Gastric bypass:** This is a surgery that creates a small pouch at the top of the stomach and then connects it directly to the small intestine.  * **Gastric banding:** This is a surgery that places a band around the top of the stomach to create a small pouch.  * **Comorbidities:** These are other health conditions that a person may have, such as diabetes or heart disease.  I hope this helps! |
| 2 | What are the dietary restrictions following weight loss surgery? | Following surgery, you will need to follow specific eating guidelines. Registered dietitians at X work directly with bariatric surgery patients to help plan healthful, appropriate meals. Additionally, you will need to stop smoking and discontinue use of NSAIDS. A list of NSAIDS is available for you to reference. |  | The dietary restrictions following weight loss surgery vary depending on the type of surgery you have. However, there are some general guidelines that all patients should follow. These include:  * **Eating small, frequent meals.** This will help you feel full and prevent you from overeating.  * **Choosing low-calorie, low-fat foods.** This will help you lose weight and maintain a healthy weight.  * **Avoiding sugary drinks.** Sugary drinks are high in calories and can contribute to weight gain.  * **Eating plenty of protein.** Protein helps you feel full and can help you maintain muscle mass.  * **Taking a daily multivitamin.** Bariatric surgery can make it difficult to absorb vitamins and minerals, so it's important to take a daily multivitamin.  In addition to these general guidelines, there are some specific foods that you should avoid after weight loss surgery. These include:  * **Greasy, fatty foods.** These foods can be difficult to digest and can cause stomach upset.  * **Raw fruits and vegetables.** Raw fruits and vegetables can be difficult to chew and digest.  * **Spicy foods.** Spicy foods can irritate your stomach and cause pain.  * **Carbonated beverages.** Carbonated beverages can cause gas and bloating.  * **Alcohol.** Alcohol can dehydrate you and make it difficult to absorb nutrients.  It's important to talk to your doctor about the specific dietary restrictions that are right for you. They can help you create a personalized plan that will help you lose weight and maintain a healthy weight after surgery.  Here are some additional restrictions that may apply after weight loss surgery:  * **Do not eat until you are full.** It is important to eat slowly and chew your food thoroughly.  * **Do not drink liquids with your meals.** This can dilute your digestive juices and make it difficult to digest your food.  * **Avoid straws.** Straws can cause air to enter your stomach, which can lead to gas and bloating.  * **Do not eat too quickly.** Eating too quickly can cause you to overeat and can also lead to stomach upset.  * **Do not eat large meals.** It is better to eat several small meals throughout the day.  * **Do not eat late at night.** This can give you heartburn and make it difficult to sleep.  Following these dietary restrictions will help you have a successful recovery from weight loss surgery and reach your weight loss goals. | 7/8/2023 | Sure. Here are the dietary restrictions following weight loss surgery in simpler terms:  * **Eat small meals throughout the day.** This will help you feel full and prevent you from overeating.  * **Choose healthy foods like fruits, vegetables, and lean protein.** These foods are low in calories and fat, and they will help you lose weight and maintain a healthy weight.  * **Avoid unhealthy foods like sugary drinks, fatty foods, and processed foods.** These foods are high in calories and fat, and they can contribute to weight gain.  * **Take a daily multivitamin.** Bariatric surgery can make it difficult to absorb vitamins and minerals, so it's important to take a daily multivitamin.  * **Be patient.** It takes time to adjust to your new diet after weight loss surgery. Be patient and don't give up.  Here are some additional restrictions that may apply after weight loss surgery:  * **Do not eat until you are full.** It is important to eat slowly and chew your food thoroughly.  * **Do not drink liquids with your meals.** This can dilute your digestive juices and make it difficult to digest your food.  * **Avoid straws.** Straws can cause air to enter your stomach, which can lead to gas and bloating.  * **Do not eat too quickly.** Eating too quickly can cause you to overeat and can also lead to stomach upset.  * **Do not eat large meals.** It is better to eat several small meals throughout the day.  * **Do not eat late at night.** This can give you heartburn and make it difficult to sleep.  Following these dietary restrictions will help you have a successful recovery from weight loss surgery and reach your weight loss goals.  I hope this helps! |
| 2 | Is it possible to gain the weight back after surgery? | Bariatric surgery has an excellent long-term track record for helping morbidly obese individuals lose weight. However, there are no guarantees with any method of weight loss, even surgery. Success is possible only if you are committed to making lifestyle and dietary changes for the rest of your life. | Is it possible to gain the weight back after weight loss surgery? | Yes, it is possible to gain weight back after weight loss surgery. In fact, it is estimated that up to 50% of people who have bariatric surgery will regain some of the weight they lost. There are a number of reasons why this can happen, including:  * **Changes in eating habits.** After surgery, people may start to eat more frequently or larger portions than they should. They may also start to eat foods that are high in calories or unhealthy fats.  * **Lack of exercise.** Exercise is important for weight loss and maintenance. After surgery, people may become less active, which can lead to weight gain.  * **Medical conditions.** Some medical conditions, such as hypothyroidism, can make it difficult to lose weight or keep it off.  * **Psychological factors.** Stress, depression, and other psychological factors can also contribute to weight gain.  If you are concerned about gaining weight back after surgery, there are a number of things you can do to help prevent it. These include:  * **Following a healthy diet.** This means eating plenty of fruits, vegetables, and whole grains, and limiting processed foods, sugary drinks, and unhealthy fats.  * **Getting regular exercise.** Aim for at least 30 minutes of moderate-intensity exercise most days of the week.  * **Monitoring your weight regularly.** This will help you to identify any weight gain early on so that you can take steps to prevent it from becoming a problem.  * **Talking to your doctor.** If you are struggling to keep the weight off, talk to your doctor. They can help you develop a plan to address the specific factors that are contributing to your weight gain.  It is important to remember that weight loss surgery is not a magic bullet. It is a tool that can help you to lose weight, but it is up to you to make the lifestyle changes necessary to keep the weight off. With careful planning and effort, you can achieve your weight loss goals and maintain a healthy weight for life. | 7/8/2023 | Sure. Weight loss surgery can help you lose a lot of weight, but it's not a guarantee that you'll keep it off forever. About half of people who have weight loss surgery will regain some of the weight they lost. There are a few reasons for this, including:  * **Eating too much or unhealthy foods.** After surgery, your stomach is smaller, so you feel full faster. But if you eat too much or eat unhealthy foods, you can still gain weight.  * **Not exercising enough.** Exercise is important for weight loss and maintenance. If you become less active after surgery, you're more likely to gain weight.  * **Medical conditions.** Some medical conditions can make it difficult to lose weight or keep it off. If you have a medical condition, talk to your doctor about how it might affect your weight loss goals.  * **Psychological factors.** Stress, depression, and other psychological factors can also contribute to weight gain. If you're struggling with these issues, talk to a therapist or counselor.  There are a few things you can do to help prevent weight regain after surgery:  * **Eat a healthy diet.** This means eating plenty of fruits, vegetables, and whole grains, and limiting processed foods, sugary drinks, and unhealthy fats.  * **Get regular exercise.** Aim for at least 30 minutes of moderate-intensity exercise most days of the week.  * **Monitor your weight regularly.** This will help you to identify any weight gain early on so that you can take steps to prevent it from becoming a problem.  * **Talk to your doctor.** If you're struggling to keep the weight off, talk to your doctor. They can help you develop a plan to address the specific factors that are contributing to your weight gain.  With careful planning and effort, you can achieve your weight loss goals and maintain a healthy weight for life. |
| 2 | What are the risks for bariatric surgery? | All surgical procedures have risks, particularly when the patient is morbidly obese. Different procedures involve different risks, and depending upon your individual circumstances, your risks may be higher or lower than average. It’s also important to know that bariatric surgeons with more experience performing weight loss surgery techniques report fewer complications. Potential risks of surgery should be discussed with your surgeon so you can make an informed choice. |  | Bariatric surgery, also known as weight loss surgery, is a major medical procedure that can help people with obesity lose weight and improve their health. However, like any surgery, it has risks.  The short-term risks of bariatric surgery can include:  * **Infection**. The surgical site can become infected.  * **Bleeding**. There is a risk of bleeding during or after surgery.  * **Adverse reactions to anesthesia**. Some people may have an allergic reaction to the anesthesia used during surgery.  * **Blood clots**. Blood clots can form in the legs or lungs after surgery.  * **Lung or breathing problems**. Some people may experience lung or breathing problems after surgery.  * **Leaks in your gastrointestinal system**. There is a risk of a leak in the stomach or intestines after surgery. This can be a serious complication that requires immediate medical attention.  The long-term risks of bariatric surgery can include:  * **Dumping syndrome**. This is a condition that can cause symptoms such as nausea, vomiting, diarrhea, and lightheadedness after eating.  * **Low blood sugar**. Some people may experience low blood sugar after surgery. This is a serious condition that can require medical attention.  * **Malnutrition**. There is a risk of malnutrition after surgery. This is because the body may not be getting enough nutrients from food.  * **Ulcers**. Some people may develop ulcers after surgery.  * **Bowel obstruction**. This is a blockage in the intestines. It is a serious complication that requires immediate medical attention.  * **Hernias**. There is a risk of hernias developing after surgery.  It is important to talk to your doctor about the risks of bariatric surgery before you decide if it is right for you. They can help you understand the risks and benefits of surgery and decide if it is the best option for you.  Here are some additional things to consider before having bariatric surgery:  * **Your age**. Bariatric surgery is generally not recommended for people under the age of 18.  * **Your health**. You should be in good overall health before having bariatric surgery.  * **Your lifestyle**. You should be willing to make changes to your lifestyle after surgery, such as eating a healthy diet and exercising regularly.  If you are considering bariatric surgery, it is important to talk to your doctor about your individual risks and benefits. They can help you decide if surgery is the right option for you. | 7/8/2023 | Sure. Bariatric surgery is a major surgery that can help you lose weight. But like any surgery, it has risks.  Some of the short-term risks of bariatric surgery include infection, bleeding, and allergic reaction to anesthesia. Some of the long-term risks of bariatric surgery include dumping syndrome, low blood sugar, malnutrition, ulcers, bowel obstruction, and hernias.  It's important to talk to your doctor about the risks of bariatric surgery before you decide if it's right for you. They can help you understand the risks and benefits of surgery and decide if it's the best option for you.  Here are some additional things to consider before having bariatric surgery:  * **Your age.** Bariatric surgery is generally not recommended for people under the age of 18.  * **Your health.** You should be in good overall health before having bariatric surgery.  * **Your lifestyle.** You should be willing to make changes to your lifestyle after surgery, such as eating a healthy diet and exercising regularly.  If you're considering bariatric surgery, it's important to talk to your doctor about your individual risks and benefits. They can help you decide if surgery is the right option for you.  In simpler terms, bariatric surgery is a big decision that should not be taken lightly. There are risks involved, but it can be a very effective way to lose weight and improve your health. Talk to your doctor to learn more about the risks and benefits of bariatric surgery and decide if it's right for you. |
| 2 | Will insurance cover bariatric surgery? | Many insurance companies now recognize obesity as a substantial health risk and are paying for bariatric surgery. You can check our list of accepted insurers or contact your insurance provider to find out if gastric bypass surgery (CPT code 43644) is a covered benefit under your insurance contract. If you have other questions, please contact Debra Cash at 216.445.1745 / 800.223.2273, ext. 51745. |  | Whether insurance will cover bariatric surgery depends on the specific insurance plan and the patient's individual circumstances. However, many insurance plans do cover bariatric surgery, especially if the patient meets certain criteria.  Here are some of the criteria that insurance companies typically require for bariatric surgery coverage:  * A body mass index (BMI) of 40 or higher, or a BMI between 35 and 39.9 with a co-morbidity, such as type 2 diabetes, high blood pressure, or sleep apnea.  * A history of unsuccessful weight loss attempts.  * A mental health evaluation to assess the patient's readiness for surgery.  * A letter of medical necessity from the patient's doctor.  The specific criteria for coverage may vary from plan to plan, so it is important to check with your insurance company to see if bariatric surgery is covered.  In addition to the criteria listed above, some insurance companies may also require that the patient meet certain weight loss goals before surgery. For example, the patient may be required to lose a certain percentage of their body weight through diet and exercise.  The cost of bariatric surgery can vary depending on the type of surgery, the surgeon's fees, and the location of the surgery. However, the average cost of bariatric surgery in the United States is between $17,000 and $26,000. If insurance covers some of the cost, the patient may still have to pay a significant amount out of pocket.  If you are considering bariatric surgery, it is important to talk to your doctor about your insurance coverage. Your doctor can help you understand the criteria for coverage and help you file the necessary paperwork. | 7/8/2023 | Sure. Here are the criteria for insurance coverage of bariatric surgery in simpler terms:  * You must be very obese (BMI of 40 or higher) or have moderate obesity (BMI of 35-39.9) with a co-morbidity.  * You must have tried to lose weight in the past and been unsuccessful.  * You must have a mental health evaluation to make sure you are ready for surgery.  * You must get a letter from your doctor stating that bariatric surgery is medically necessary.  If you meet these criteria, your insurance company may cover some or all of the cost of bariatric surgery. The specific amount of coverage will vary depending on your insurance plan.  It is important to talk to your doctor about your insurance coverage before you decide to have bariatric surgery. Your doctor can help you understand the criteria for coverage and help you file the necessary paperwork.  Here are some additional things to keep in mind:  * The cost of bariatric surgery can vary depending on the type of surgery, the surgeon's fees, and the location of the surgery.  * Insurance companies may require that you meet certain weight loss goals before surgery.  * Bariatric surgery is a major surgery and there are risks involved. It is important to talk to your doctor about the risks before you decide to have surgery. |
| 2 | Can bariatric surgery be reversed? | Laparoscopic gastric banding surgery is reversible in a minimally invasive manner. Gastric bypass surgery is potentially reversible. Reversal requires another operation of the same, or greater, magnitude with the same, or greater, risks. |  | Yes, bariatric surgery can be reversed in some cases. However, it is important to note that reversal is not always possible, and it is not always the best option for patients.  The most common type of bariatric surgery that can be reversed is gastric bypass surgery. This surgery involves creating a small pouch in the stomach and then connecting it directly to the small intestine. The rest of the stomach is bypassed. Reversal of gastric bypass surgery involves reconnecting the stomach and intestine.  Other types of bariatric surgery, such as gastric sleeve surgery and adjustable gastric banding, are generally not reversible. This is because these surgeries involve removing or stapling part of the stomach, which cannot be undone.  There are a few reasons why a patient might want to have their bariatric surgery reversed. One reason is if they experience complications from the surgery. For example, some patients develop nutritional deficiencies or have difficulty absorbing nutrients after gastric bypass surgery. Another reason for reversal is if the patient regains a significant amount of weight after surgery.  However, it is important to remember that reversal of bariatric surgery is not always successful. In some cases, patients may not be able to regain their full range of motion after surgery. Additionally, there is a risk of complications, such as infection or bleeding.  If you are considering having bariatric surgery, it is important to talk to your doctor about the risks and benefits of reversal. Your doctor can help you decide if reversal is the right option for you.  Here are some additional things to consider before having bariatric surgery:  * The risks and benefits of the surgery  * The potential for complications  * The possibility of needing to have the surgery reversed  * The long-term effects of the surgery on your health  It is also important to find a qualified surgeon who has experience performing bariatric surgery. You should feel comfortable asking your surgeon questions about the surgery and its risks. | 7/8/2023 | Sure. Bariatric surgery is a type of surgery that can help people lose weight. There are different types of bariatric surgery, but some of them can be reversed. This means that if you have bariatric surgery and then decide that you want to have the surgery reversed, it is possible to do so.  However, not all types of bariatric surgery can be reversed. For example, gastric sleeve surgery and adjustable gastric banding are generally not reversible because they involve removing or stapling part of the stomach, which cannot be undone.  The most common type of bariatric surgery that can be reversed is gastric bypass surgery. This surgery involves creating a small pouch in the stomach and then connecting it directly to the small intestine. The rest of the stomach is bypassed. Reversal of gastric bypass surgery involves reconnecting the stomach and intestine.  There are a few reasons why a patient might want to have their bariatric surgery reversed. One reason is if they experience complications from the surgery. For example, some patients develop nutritional deficiencies or have difficulty absorbing nutrients after gastric bypass surgery. Another reason for reversal is if the patient regains a significant amount of weight after surgery.  However, it is important to remember that reversal of bariatric surgery is not always successful. In some cases, patients may not be able to regain their full range of motion after surgery. Additionally, there is a risk of complications, such as infection or bleeding.  If you are considering having bariatric surgery, it is important to talk to your doctor about the risks and benefits of reversal. Your doctor can help you decide if reversal is the right option for you.  Here is a simplified table of the different types of bariatric surgery and whether or not they can be reversed:  \| Type of Bariatric Surgery \| Can Be Reversed? \|  \|---\|---\|  \| Gastric Bypass Surgery \| Yes \|  \| Gastric Sleeve Surgery \| No \|  \| Adjustable Gastric Banding \| No \|  \| Biliopancreatic Diversion with Duodenal Switch \| Yes, but rarely done \|  I hope this helps! |
| 2 | What if I need revision surgery? | Weight loss surgery is not a magic bullet and while most patients are successful after weight loss surgery, there are instances where revision weight loss surgery is required. Whether reasons for seeking revision weight loss surgery are due to inadequate weight loss/weight regain, unresolved co-morbidities and/or medical complications, there is possibly a revision solution to these problems.  Revisional bariatric operations, regardless of the previous surgery, are always challenging for a surgeon. They require adequate expertise to achieve desired results. Bariatric Centers of Excellence certified by the American Society for Bariatric Surgery provide a multi-disciplinary team and facilities for managing patients requiring revision of their surgery. It is highly recommended that patients requiring gastric bypass revision or any other kind of revisional surgery find a surgeon at a bariatric center that has much experience with revisional bariatric surgery. | What if I need revision surgery after weight loss surgery? | Revision surgery is a procedure that corrects or improves prior weight loss surgery. It might be appropriate for you if you've had less-than-optimal weight loss or weight regain, or if you experienced complications from your original procedure.  There are a number of reasons why you might need revision surgery after weight loss surgery. Some of the most common reasons include:  * **Inadequate weight loss:** If you have not lost enough weight after your initial surgery, you may be a candidate for revision surgery. This is especially true if you have a high body mass index (BMI) or if you have health problems related to your weight.  * **Weight regain:** If you have regained weight after your initial surgery, you may be a candidate for revision surgery. This is especially true if you have regained a significant amount of weight or if you are experiencing health problems related to your weight.  * **Complications from the initial surgery:** If you have experienced complications from your initial surgery, you may be a candidate for revision surgery. This is especially true if the complications are causing you significant problems or if they are preventing you from losing weight.  The type of revision surgery that you will need will depend on the reason why you are having the surgery and the type of surgery that you had originally. Some of the most common revision procedures include:  * **Sleeve gastrectomy conversion to gastric bypass:** This procedure is done for people who have had a sleeve gastrectomy but have not lost enough weight. The sleeve is converted to a gastric bypass, which is a more restrictive procedure.  * **Gastric bypass revision:** This procedure is done for people who have had a gastric bypass but have experienced complications or have not lost enough weight. The revision may involve changing the size of the pouch or the length of the roux limb.  * **Band removal:** This procedure is done for people who have had an adjustable gastric band but have not lost enough weight or have experienced complications. The band is removed, and the stomach is allowed to expand back to its normal size.  Revision surgery is a major surgery, and it is important to weigh the risks and benefits carefully before deciding to have it. However, for some people, revision surgery can be a life-changing procedure that allows them to lose weight and improve their health.  If you are considering revision surgery, it is important to talk to your doctor about your options. They can help you determine if revision surgery is right for you and can refer you to a surgeon who specializes in revisional bariatric surgery. | 7/8/2023 | Sure. Revision surgery is a type of surgery that is done to fix or improve a previous weight loss surgery. There are a few reasons why you might need revision surgery, including:  * You didn't lose enough weight after your initial surgery.  * You regained weight after your initial surgery.  * You had complications from your initial surgery.  The type of revision surgery that you will need will depend on the reason why you are having the surgery and the type of surgery that you had originally.  For example, if you had a sleeve gastrectomy but didn't lose enough weight, you might need to have the sleeve converted to a gastric bypass. If you had a gastric bypass but had complications, you might need to have the pouch or the roux limb resized.  Revision surgery is a major surgery, so it's important to talk to your doctor about your options before you decide to have it. However, for some people, revision surgery can be a life-changing procedure that allows them to lose weight and improve their health.  Here is a table that summarizes the different types of revision surgery:  \| Type of revision surgery \| What it does \|  \|---\|---\|  \| Sleeve gastrectomy conversion to gastric bypass \| The sleeve is converted to a gastric bypass, which is a more restrictive procedure. \|  \| Gastric bypass revision \| The pouch or the roux limb is resized. \|  \| Band removal \| The band is removed, and the stomach is allowed to expand back to its normal size. \|  I hope this helps! Let me know if you have any other questions. |
| 2 | What is my ideal body weight? | X's Bariatric and Metabolic Institute follows the guidelines set out by the body mass index scale. This scale recommends a BMI of between 18.5 and 24.9. You can use the calculator above to find your current BMI and ideal BMI. |  | I can't tell you your ideal body weight without knowing your height, gender, and age. However, I can give you a general idea of how to calculate your ideal body weight.  There are a few different formulas that can be used to calculate ideal body weight. One common formula is the Hamwi method, which is as follows:  * Male ideal body weight = 48 kilograms (106 lb) + 1.1 kilograms (2.4 lb) × (height (cm) − 152)  * Female ideal body weight = 45.4 kilograms (100 lb) + 0.9 kilograms (2.0 lb) × (height (cm) − 152)  For example, if you are a 5'10" (178 cm) male, your ideal body weight using the Hamwi method would be 73 kilograms (161 lb).  It's important to remember that these are just estimates. Your ideal body weight may be different depending on your individual body composition, muscle mass, and other factors. It's also important to focus on overall health and fitness, rather than just weight loss.  If you're concerned about your weight, it's a good idea to talk to your doctor or a registered dietitian. They can help you create a healthy weight loss plan that's right for you. | 7/8/2023 | Sure. Here's a simpler explanation of how to calculate your ideal body weight using the Hamwi method:  1. Start by finding your height in centimeters. For example, if you are 5'10", your height in centimeters is 178 cm.  2. Subtract 152 from your height in centimeters. This will give you a number that represents the difference between your height and 5 feet.  3. Multiply the number you just calculated by 1.1 kilograms (2.4 lb) for men, or 0.9 kilograms (2.0 lb) for women.  4. Add 48 kilograms (106 lb) for men, or 45.4 kilograms (100 lb) for women, to the number you just calculated.  The answer you get is your estimated ideal body weight.  For example, if you are a 5'10" male, your ideal body weight using the Hamwi method would be 73 kilograms (161 lb).  Again, it's important to remember that these are just estimates. Your ideal body weight may be different depending on your individual body composition, muscle mass, and other factors. It's also important to focus on overall health and fitness, rather than just weight loss.  If you're concerned about your weight, it's a good idea to talk to your doctor or a registered dietitian. They can help you create a healthy weight loss plan that's right for you. |
| 2 | Is there support from surgeons and staff following weight loss surgery? | Xs gastric bypass surgery health care team is there to guide patients after each procedure. Regular follow-up visits are scheduled during the first year after weight loss surgery to check overall physical and mental health, metabolism and nutritional status. |  | Yes, there is usually support from surgeons and staff following weight loss surgery. This support can take many forms, including:  * **Follow-up appointments:** You will typically have regular follow-up appointments with your surgeon or bariatric team in the months and years following surgery. These appointments will allow your doctor to monitor your progress and make sure you are recovering well.  * **Nutritional counseling:** Many surgeons and bariatric teams offer nutritional counseling to help you make healthy changes to your diet after surgery. This counseling can help you learn about the foods you can and cannot eat, as well as how to get the nutrients you need.  * **Exercise counseling:** Some surgeons and bariatric teams also offer exercise counseling to help you get started with a safe and effective exercise program. This counseling can help you learn how to exercise safely after surgery and how to gradually increase your activity level.  * **Support groups:** There are also many support groups available for people who have had weight loss surgery. These groups can provide emotional support and practical advice from people who have been through the same experience.  The amount and type of support you receive will vary depending on your surgeon and bariatric team. However, most surgeons and teams offer some form of support to help you succeed after surgery.  In addition to support from your surgeon and staff, there are also many other resources available to help you after weight loss surgery. These resources include:  * **Books and websites:** There are many books and websites available that provide information about weight loss surgery and how to live a healthy life after surgery.  * **Online forums:** There are also many online forums where you can connect with other people who have had weight loss surgery. These forums can provide support and advice from people who understand what you are going through.  If you are considering weight loss surgery, it is important to talk to your doctor about the support that will be available to you after surgery. This will help you make an informed decision about whether or not surgery is right for you. | 7/8/2023 | Sure. After weight loss surgery, you will need to make some changes to your diet and lifestyle. Your surgeon or bariatric team will help you with this, and they may also offer support groups or other resources.  Here are some of the things you can expect after weight loss surgery:  * You will need to eat smaller meals more often.  * You will need to avoid certain foods, such as fatty foods, sugary foods, and carbonated beverages.  * You will need to take vitamins and supplements to make sure you are getting the nutrients you need.  * You will need to exercise regularly.  Your surgeon or bariatric team will help you create a plan that is right for you. They will also be there to support you as you make these changes.  Here are some of the resources that are available to help you after weight loss surgery:  * **Support groups:** There are many support groups available for people who have had weight loss surgery. These groups can provide emotional support and practical advice from people who have been through the same experience.  * **Books and websites:** There are many books and websites available that provide information about weight loss surgery and how to live a healthy life after surgery.  * **Online forums:** There are also many online forums where you can connect with other people who have had weight loss surgery. These forums can provide support and advice from people who understand what you are going through.  If you are considering weight loss surgery, it is important to talk to your doctor about the support that will be available to you after surgery. This will help you make an informed decision about whether or not surgery is right for you.  I hope this explanation is simpler. Let me know if you have any other questions. |
| 2 | What bariatric surgical procedures are performed by surgeons at X? | "Xc offers many surgical options for patients who are interested in bariatric surgery.  The most commonly performed malabsorptive procedure is called the Roux-en-Y gastric bypass. This method makes the stomach smaller and alters digestion. Less food can be eaten and fewer calories can be absorbed. The bariatric surgeon creates a small pouch by dividing the upper end of the stomach. Next, a Y-shaped section of the small intestine is attached to the pouch to allow food to bypass the lower stomach, the duodenum (the first segment of the small intestine), as well as the first portion of the jejunum (the second segment of the small intestine). The procedure creates a direct connection from the stomach to the lower segment of the small intestine, literally bypassing portions of the digestive tract that absorb calories and nutrients.  We can also perform the sleeve gastrectomy as a laparoscopic procedure. This involves making five or six small incisions in the abdomen and performing the procedure using a video camera (laparoscope) and long instruments that are placed through these small incisions. During the laparoscopic sleeve gastrectomy (LSG), about 75% of the stomach is removed leaving a narrow gastric “tube” or “sleeve”. No intestines are removed or bypassed during the sleeve gastrectomy. The LSG takes one to two hours to complete. We have an animated video of a sleeve gastrectomy procedure available as well.  In select patients, depending on their situations and needs, X bariatric surgeons can perform other surgical options such as duodenal switch." |  | The surgeons at X perform a variety of bariatric surgical procedures, including:  * **Gastric sleeve:** This procedure removes about 80% of the stomach, leaving behind a narrow "sleeve." This reduces the amount of food you can eat and helps you feel full faster.  * **Roux-en-Y gastric bypass:** This procedure creates a small pouch at the top of your stomach and then connects it to the small intestine, bypassing part of your stomach and small intestine. This reduces the amount of food you can eat and also reduces the absorption of calories and nutrients.  * **Adjustable gastric band:** This procedure involves placing a band around the top of your stomach to create a small pouch. The band can be tightened or loosened over time to adjust the amount of food you can eat.  * **Duodenal switch:** This procedure is a more complex version of the Roux-en-Y gastric bypass. It creates a smaller pouch at the top of your stomach and then connects it to the small intestine, bypassing even more of your stomach and small intestine. This results in even greater weight loss than the Roux-en-Y gastric bypass.  The type of bariatric surgery that is right for you will depend on your individual needs and medical history. The surgeons at X will work with you to determine the best option for you.  Here are some additional details about each of these procedures:  * **Gastric sleeve:** The gastric sleeve is the most commonly performed bariatric surgery in the United States. It is a relatively safe and effective procedure that can lead to significant weight loss. However, it is not reversible.  * **Roux-en-Y gastric bypass:** The Roux-en-Y gastric bypass is a more complex procedure than the gastric sleeve, but it can lead to even greater weight loss. It is also a more effective way to control blood sugar levels in people with diabetes. However, the Roux-en-Y gastric bypass is not reversible.  * **Adjustable gastric band:** The adjustable gastric band is a less invasive procedure than the gastric sleeve or Roux-en-Y gastric bypass. It is also reversible, which means that if you decide you no longer want the band, it can be removed. However, the adjustable gastric band is not as effective as the other two procedures at leading to long-term weight loss.  * **Duodenal switch:** The duodenal switch is the most complex bariatric surgery procedure. It is also the most effective at leading to long-term weight loss. However, it is also the most risky procedure.  If you are considering bariatric surgery, it is important to talk to your doctor about the risks and benefits of each procedure. The surgeons at X can help you make the best decision for your individual needs. | 7/8/2023 | Sure. The surgeons at X perform a variety of procedures to help people lose weight. These procedures are called bariatric surgeries. There are four main types of bariatric surgeries: gastric sleeve, Roux-en-Y gastric bypass, adjustable gastric band, and duodenal switch.  * **Gastric sleeve:** This procedure removes about 80% of the stomach, leaving behind a narrow "sleeve." This makes you feel full faster and helps you eat less food.  * **Roux-en-Y gastric bypass:** This procedure creates a small pouch at the top of your stomach and then connects it to the small intestine, bypassing part of your stomach and small intestine. This makes you feel full faster and also reduces the amount of calories and nutrients you absorb from food.  * **Adjustable gastric band:** This procedure involves placing a band around the top of your stomach to create a small pouch. The band can be tightened or loosened over time to adjust the amount of food you can eat.  * **Duodenal switch:** This procedure is a more complex version of the Roux-en-Y gastric bypass. It creates a smaller pouch at the top of your stomach and then connects it to the small intestine, bypassing even more of your stomach and small intestine. This results in even greater weight loss than the Roux-en-Y gastric bypass.  The type of bariatric surgery that is right for you will depend on your individual needs and medical history. The surgeons at X will work with you to determine the best option for you.  In general, bariatric surgeries are safe and effective. However, they are also major surgeries and there are some risks involved. It is important to talk to your doctor about the risks and benefits of each procedure before making a decision.  I hope this explanation is simpler. Let me know if you have any other questions. |
| 3 | How do I know if I am eligible? | Patients must meet specific criteria before they will be considered for an evaluation with the X Bariatric & Metabolic Surgery program.  - Have a Body Mass Index (BMI) of greater than 40, or a BMI of35 accompanied by other issues related to health (co-morbidities). Calculate your BMI. These co-morbidities include diabetes, high blood pressure, coronary artery disease, obstructive sleep apnea, osteoarthritis, hyperlipidemia (high cholesterol, triglycerides, etc), metabolic syndrome, polycystic ovarian disease, pseudotumor cerebri, etc.  - Be mobile, medically stable, and have an actively involved and supportive primary care physician.  - Have a stable psychological status with adequate social support system to help them through the course of surgery and the postoperative period. | How do I know if I am eligible for weight loss surgery? | The eligibility criteria for weight loss surgery vary depending on the type of surgery you are considering and your individual circumstances. However, there are some general guidelines that most surgeons follow.  * **Body mass index (BMI)**. Most surgeons require a BMI of 40 or higher for adults to be eligible for weight loss surgery. However, some surgeons may consider you for surgery if your BMI is between 35 and 40 if you have other obesity-related health conditions.  * **Obesity-related health conditions**. You may also be eligible for weight loss surgery if you have obesity-related health conditions such as type 2 diabetes, high blood pressure, sleep apnea, or heart disease.  * **Age**. Most surgeons will not perform weight loss surgery on people under the age of 18. However, there are some exceptions for adolescents who have severe obesity and other obesity-related health conditions.  * **Psychological evaluation**. You will need to undergo a psychological evaluation to make sure that you are mentally prepared for weight loss surgery and the lifestyle changes that come with it.  * **Commitment to lifestyle changes**. Weight loss surgery is not a magic bullet. You will still need to make significant changes to your diet and exercise habits in order to maintain your weight loss after surgery.  If you think you may be eligible for weight loss surgery, talk to your doctor. They can assess your individual circumstances and help you determine if surgery is right for you.  Here are some additional factors that may affect your eligibility for weight loss surgery:  * **Your weight loss history**. If you have tried to lose weight through diet and exercise in the past and have not been successful, you may be more likely to be approved for surgery.  * **Your overall health**. If you have other health conditions, such as heart disease or diabetes, your surgeon will need to consider how surgery will affect your overall health.  * **Your insurance coverage**. If you have insurance, you will need to check with your insurance company to see if they cover weight loss surgery.  If you are considering weight loss surgery, it is important to do your research and talk to your doctor to make sure that it is the right decision for you. | 7/9/2023 | Sure. To be eligible for weight loss surgery, you usually need to have a BMI of 40 or higher, or a BMI of 35 or higher with other obesity-related health conditions. You also need to be at least 18 years old and be mentally prepared for surgery and the lifestyle changes that come with it. You will need to make significant changes to your diet and exercise habits in order to maintain your weight loss after surgery.  Here are some simple terms to understand weight loss surgery eligibility:  * **BMI**: Body mass index is a measure of body fat based on your height and weight. A BMI of 25 or higher is considered overweight, and a BMI of 30 or higher is considered obese.  * **Obesity-related health conditions**: These are health conditions that are caused or made worse by obesity. They include type 2 diabetes, high blood pressure, sleep apnea, and heart disease.  * **Psychological evaluation**: This is a test that helps to assess your mental health and readiness for weight loss surgery.  * **Lifestyle changes**: These are changes to your diet and exercise habits that you will need to make in order to maintain your weight loss after surgery.  If you are considering weight loss surgery, talk to your doctor to learn more about the eligibility criteria and to see if surgery is right for you. |
| 3 | Do I have to wait to get pregnant after bariatric surgery? Will the baby be healthy? | Women generally have significantly increased fertility after weight loss surgery. Birth control pills do NOT work as well in heavy patients and during the time of rapid weight loss. We strongly recommend all child bearing age women to use other ways of contraception after bariatric surgery.  Most surgeons recommend waiting 12-18 months after weight loss surgery before getting pregnant. After losing most of the excess weight, there is much less risk of having problems during pregnancy (gestational diabetes, eclampsia, macrosomia) and during childbirth. There is also decreased risk of having miscarriages and stillbirths. The kids born after mon’s bariatric surgery are less prone to being obese later in their life. Bariatric surgery also reduces the chance of needing a C-section. |  | Yes, you should wait to get pregnant after bariatric surgery. The American College of Obstetricians and Gynecologists (ACOG) recommends waiting at least 12 to 24 months after surgery before trying to conceive. This is because rapid weight loss during pregnancy can be harmful to the fetus.  Pregnancy after bariatric surgery can be safe and healthy, but it is important to be aware of the risks. These risks include:  * **Malnutrition:** Bariatric surgery can lead to malabsorption of nutrients, which can increase the risk of nutrient deficiencies in the mother and the baby.  * **Premature birth:** Women who have had bariatric surgery are more likely to have premature births.  * **Low birth weight:** Babies born to mothers who have had bariatric surgery are more likely to be born with low birth weight.  * **Birth defects:** There is a slightly increased risk of birth defects in babies born to mothers who have had bariatric surgery.  If you are considering pregnancy after bariatric surgery, it is important to talk to your doctor about the risks and benefits. Your doctor can help you make a decision about when is the best time for you to get pregnant.  Here are some tips for having a healthy pregnancy after bariatric surgery:  * **Make sure you are nutritionally stable.** This means that you are getting enough of all the essential vitamins and minerals.  * **Take prenatal vitamins.** Prenatal vitamins can help to ensure that you and your baby are getting the nutrients you need.  * **See your doctor regularly.** Your doctor will monitor your health throughout your pregnancy and make sure that you are doing well.  * **Be aware of the signs of complications.** If you experience any problems during your pregnancy, be sure to tell your doctor right away.  With careful planning and monitoring, you can have a healthy and successful pregnancy after bariatric surgery. | 7/9/2023 | Sure. Bariatric surgery is a weight loss surgery that can help you lose a lot of weight quickly. However, it can also affect your pregnancy. If you are considering pregnancy after bariatric surgery, it is important to talk to your doctor about the risks and benefits.  One of the main risks of pregnancy after bariatric surgery is that you may not get enough nutrients. This is because bariatric surgery can change the way your body absorbs nutrients. As a result, you may need to take prenatal vitamins to make sure you are getting the nutrients you need.  Another risk of pregnancy after bariatric surgery is that you may have a premature birth. This is because rapid weight loss during pregnancy can put stress on your body and increase the risk of preterm labor.  Finally, there is a slightly increased risk of birth defects in babies born to mothers who have had bariatric surgery. However, this risk is still very low.  If you are considering pregnancy after bariatric surgery, it is important to talk to your doctor about the risks and benefits. Your doctor can help you make a decision about when is the best time for you to get pregnant.  Here are some tips for having a healthy pregnancy after bariatric surgery:  * **Make sure you are nutritionally stable.** This means that you are getting enough of all the essential vitamins and minerals.  * **Take prenatal vitamins.** Prenatal vitamins can help to ensure that you and your baby are getting the nutrients you need.  * **See your doctor regularly.** Your doctor will monitor your health throughout your pregnancy and make sure that you are doing well.  * **Be aware of the signs of complications.** If you experience any problems during your pregnancy, be sure to tell your doctor right away.  With careful planning and monitoring, you can have a healthy and successful pregnancy after bariatric surgery.  In simpler terms, if you have had bariatric surgery, it is best to wait at least 12 to 24 months before trying to get pregnant. This will give your body time to adjust to the surgery and to make sure you are getting enough nutrients. If you do get pregnant before then, it is important to see your doctor regularly and to take prenatal vitamins. With careful planning and monitoring, you can have a healthy and successful pregnancy. |
| 3 | How long will I stay in the hospital after bariatric surgery? | All surgeries are performed at the new X Medical Center. The average surgical time for gastric bypass is about 2 hours and sleeve gastrectomy is about 1-2 hours. The laparoscopy totally changed bariatric surgery. Almost all our patients can walk a few hours after surgery. They start to have liquids the day after surgery. The majority (more than 95%) of patients only stay in the hospital for one night or two nights. |  | The length of your hospital stay after bariatric surgery will depend on the type of surgery you have and how well you recover. Generally speaking, patients who have laparoscopic surgery stay in the hospital for 1-2 days, while those who have open surgery stay for 5-7 days.  Here is a breakdown of the average hospital stay for different types of bariatric surgery:  * **Laparoscopic gastric band:** 1-2 days  * **Laparoscopic sleeve gastrectomy:** 1-2 days  * **Laparoscopic Roux-en-Y gastric bypass:** 2-3 days  * **Open gastric band:** 3-5 days  * **Open sleeve gastrectomy:** 5-7 days  * **Open Roux-en-Y gastric bypass:** 5-7 days  However, there are some factors that can affect your hospital stay, such as:  * Your overall health and fitness before surgery  * Any complications that arise during surgery or afterward  * Your ability to tolerate pain medication  * Your ability to eat and drink normally  If you have any questions about your hospital stay, be sure to talk to your surgeon. They will be able to give you a more accurate estimate based on your individual circumstances.  Here are some other things to keep in mind after bariatric surgery:  * You will need to have someone stay with you for the first few days after you are discharged from the hospital.  * You will need to follow a strict diet and activity plan.  * You may experience some side effects, such as nausea, vomiting, and constipation.  * It is important to take your pain medication as prescribed.  * You should schedule follow-up appointments with your surgeon to monitor your progress.  Bariatric surgery is a major surgery, but it can be a life-changing procedure. With careful planning and follow-up, you can expect a full recovery and a healthier life. | 7/9/2023 | Sure. The length of your hospital stay after bariatric surgery will depend on the type of surgery you have and how well you recover. Most people who have laparoscopic surgery stay in the hospital for 1-2 days, while those who have open surgery stay for 5-7 days.  Here is a simple table to summarize the average hospital stay for different types of bariatric surgery:  \| Type of surgery \| Average hospital stay \|  \|---\|---\|  \| Laparoscopic gastric band \| 1-2 days \|  \| Laparoscopic sleeve gastrectomy \| 1-2 days \|  \| Laparoscopic Roux-en-Y gastric bypass \| 2-3 days \|  \| Open gastric band \| 3-5 days \|  \| Open sleeve gastrectomy \| 5-7 days \|  \| Open Roux-en-Y gastric bypass \| 5-7 days \|  Some factors that can affect your hospital stay include your overall health and fitness before surgery, any complications that arise during surgery or afterward, your ability to tolerate pain medication, and your ability to eat and drink normally.  It is important to talk to your surgeon about your individual circumstances so they can give you a more accurate estimate of your hospital stay.  I hope this helps! |
| 3 | When can I go back to work after surgery? | After surgery, most patients return to work after two to four weeks. Some may be able to work from home as early as one week after surgery. Because of the significantly reduced calorie intake, many patients have low energy for 1-2 weeks after surgery. However, most patients gain their energy back once they start to have soft food. | When can I go back to work after weight loss surgery? | The amount of time you need to take off work after weight loss surgery will vary depending on the type of surgery you have, your overall health, and the demands of your job.  * **Lap-Band surgery:** This is the shortest recovery time, and most people can return to work after 1-2 weeks.  * **Gastric sleeve surgery:** This surgery typically requires a 2-3 day hospital stay, and most people can return to work after 2-4 weeks.  * **Gastric bypass surgery:** This surgery requires a 3-5 day hospital stay, and most people can return to work after 4-6 weeks.  If your job requires strenuous activity, you may need to wait even longer to return to work. Your surgeon will be able to give you a more specific timeline based on your individual circumstances.  Here are some other factors that may affect your recovery time:  * **Your age and overall health:** If you are older or have other health conditions, you may need to take more time to recover.  * **The type of anesthesia used:** General anesthesia can have a longer recovery time than local anesthesia.  * **Any complications that occur during surgery:** If you have any complications, such as bleeding or infection, you may need to take more time to recover.  It is important to listen to your body and take things slowly after surgery. If you are feeling tired or sore, take a break. You should also avoid lifting anything heavy or doing any strenuous activity until your surgeon gives you the okay.  Here are some tips for returning to work after weight loss surgery:  * **Talk to your employer about your surgery and recovery plan.** They may be able to make accommodations for you, such as allowing you to work from home or starting back part-time.  * **Take things slowly at first.** Don't try to do too much too soon.  * **Listen to your body and rest when you need to.**  * **Stay hydrated and eat healthy foods.**  * **Take your pain medication as prescribed.**  * **See your surgeon for regular follow-up appointments.**  With proper care and planning, you can return to work after weight loss surgery without any problems. | 7/9/2023 | Sure. The recovery time for weight loss surgery depends on the type of surgery you have and your overall health. Most people can return to work after 1-2 weeks for lap-band surgery, 2-4 weeks for gastric sleeve surgery, and 4-6 weeks for gastric bypass surgery. However, if your job requires strenuous activity, you may need to wait even longer.  Here is a table to summarize the recovery times for different types of weight loss surgery:  \| Type of Surgery \| Recovery Time \|  \|---\|---\|  \| Lap-Band surgery \| 1-2 weeks \|  \| Gastric sleeve surgery \| 2-4 weeks \|  \| Gastric bypass surgery \| 4-6 weeks \|  It is important to listen to your body and take things slowly after surgery. If you are feeling tired or sore, take a break. You should also avoid lifting anything heavy or doing any strenuous activity until your surgeon gives you the okay.  Here are some tips for returning to work after weight loss surgery:  * Talk to your employer about your surgery and recovery plan.  * Take things slowly at first.  * Listen to your body and rest when you need to.  * Stay hydrated and eat healthy foods.  * Take your pain medication as prescribed.  * See your surgeon for regular follow-up appointments.  With proper care and planning, you can return to work after weight loss surgery without any problems. |
| 3 | How bad is the pain after surgery? | Most patients have some pain after surgery. However, the pain is usually just a little bit more than laparoscopic gallbladder surgery. Most patients just need to take oral pain medications for a few days after surgery. The majority of our patients felt that pain was not a major issue during their recovery. | How bad is the pain after weight loss surgery? | The pain after weight loss surgery can vary depending on the type of surgery you have and your individual experience. However, in general, most people experience some level of pain after surgery. The pain is usually most severe in the first few days after surgery, and it gradually decreases over time.  The most common type of pain after weight loss surgery is incision pain. This is the pain at the site of the incisions made during surgery. The incisions are usually made in the abdomen, and they may be either laparoscopic or open incisions. Laparoscopic incisions are smaller than open incisions, and they usually heal more quickly.  Other types of pain that can occur after weight loss surgery include:  * Shoulder pain: This pain is caused by the gas that is used during surgery. The gas can irritate the lining of the shoulder joint, causing pain.  * Gas pain: This pain is caused by the air that is swallowed during surgery. The air can get trapped in the intestines, causing gas pain.  * Muscle pain: This pain is caused by the muscle stretching that occurs during surgery. The muscles in the abdomen and chest may be sore for a few days after surgery.  Pain after weight loss surgery is usually managed with medication. Your doctor will prescribe pain medication to help you manage your pain. You may also be given a heating pad or ice pack to help relieve pain.  If you are experiencing pain after weight loss surgery, it is important to talk to your doctor. They can help you determine the cause of the pain and recommend the best treatment.  Here are some tips to help you manage pain after weight loss surgery:  * Take your pain medication as prescribed by your doctor.  * Apply heat or ice to the affected area.  * Get plenty of rest.  * Avoid strenuous activity.  * Eat small, frequent meals.  * Drink plenty of fluids.  If you are experiencing severe pain, or if the pain does not improve with medication, contact your doctor. | 7/9/2023 | Sure. After weight loss surgery, most people will feel some pain. The pain is usually most severe in the first few days after surgery, and it gradually decreases over time. The pain is usually in the incision area, but it can also be in the shoulder or abdomen.  The pain is usually managed with medication. Your doctor will prescribe pain medication to help you manage your pain. You may also be given a heating pad or ice pack to help relieve pain.  If you are experiencing pain after weight loss surgery, it is important to talk to your doctor. They can help you determine the cause of the pain and recommend the best treatment.  Here are some tips to help you manage pain after weight loss surgery:  * Take your pain medication as prescribed by your doctor.  * Apply heat or ice to the affected area.  * Get plenty of rest.  * Avoid strenuous activity.  * Eat small, frequent meals.  * Drink plenty of fluids.  If you are experiencing severe pain, or if the pain does not improve with medication, contact your doctor.  In simpler terms, the pain after weight loss surgery is usually not too bad, but it is important to take care of yourself and manage the pain so that you can heal properly. |
| 3 | When can I start exercising again after surgery? | As soon as patients totally wake up from anesthesia, we encourage patients to walk. The key is to start slow and listen to your body. If you lift weights or do sports, stay “low impact” for the first month (avoid competition, think participation). If you experience pain or discomfort, stop and relax. Avoid lifting heavy objects for the first 6 weeks. If you swim, your wounds need to be healed over before you get back in the water and always swim slowly at the beginning. | When can I start exercising again after weight loss surgery? | The timeline for starting exercise after weight loss surgery will vary depending on individual factors, such as overall health and the extent of the surgery. However, in general, most patients can start light exercise, such as walking, as soon as a week after the surgery and begin a full workout regimen 4 to 6 weeks post-op.  Here are some general guidelines for when you can start exercising after weight loss surgery:  * **Walking:** You can start walking as soon as you feel up to it, typically within a few days of surgery. Start with short walks and gradually increase the distance and duration as you feel stronger.  * **Other low-impact exercises:** Other low-impact exercises, such as swimming, biking, or yoga, can be started as soon as 2 weeks after surgery.  * **High-impact exercises:** High-impact exercises, such as running or jumping, should be avoided for at least 4 to 6 weeks after surgery.  It is important to listen to your body and not push yourself too hard when you are starting to exercise after weight loss surgery. If you experience any pain, stop and rest. You should also talk to your doctor before starting any new exercise program.  Here are some tips for exercising safely after weight loss surgery:  * Start slowly and gradually increase the intensity and duration of your workouts.  * Listen to your body and stop if you experience any pain.  * Stay hydrated.  * Wear comfortable clothing and shoes.  * Avoid exercising in hot or humid weather.  Exercising after weight loss surgery is an important part of your overall recovery and weight loss journey. It can help you to:  * Lose weight more effectively.  * Improve your cardiovascular health.  * Strengthen your muscles and bones.  * Improve your mood and energy levels.  If you have any questions about when you can start exercising after weight loss surgery, be sure to talk to your doctor. | 7/9/2023 | Sure. Here are the simplified guidelines for when you can start exercising after weight loss surgery:  * **Walking:** You can start walking as soon as you feel up to it.  * **Other low-impact exercises:** You can start other low-impact exercises, such as swimming, biking, or yoga, 2 weeks after surgery.  * **High-impact exercises:** You should avoid high-impact exercises, such as running or jumping, for at least 4 to 6 weeks after surgery.  It is important to listen to your body and not push yourself too hard when you are starting to exercise after weight loss surgery. If you experience any pain, stop and rest.  Here are some tips for exercising safely after weight loss surgery:  * Start slowly and gradually increase the intensity and duration of your workouts.  * Listen to your body and stop if you experience any pain.  * Stay hydrated.  * Wear comfortable clothing and shoes.  * Avoid exercising in hot or humid weather.  I hope this helps! |
| 3 | Do most patients need to have plastic surgery after weight loss? | After losing most of the excess weight, most patients may have some loose or sagging skin.  For many patients, this might be just temporary. The degree of loose or sagging skin depends upon several things, including how much weight you lose, your age, your genetics and whether or not you exercise or smoke.  Generally, many patients can hide loose skin by clothing or compression garments.  We generally recommend wait for at least 18 months before having plastic surgery.  Plastic surgery to remove excess abdominal and breast skin is often covered by insurance because it may cause rash.  You may have to pay yourself for the areas such as arms because it is considered cosmetic by your insurer. | Do most patients need to have plastic surgery after weight loss following weight loss surgery? | According to the American Society of Plastic Surgeons, about 70-90% of patients who have undergone weight loss surgery will eventually need some form of plastic surgery to address excess skin and other problems that can arise from significant weight loss. The most common procedures include:  * Abdominoplasty (tummy tuck) to remove excess skin and tighten the abdominal muscles  * Breast lift to improve the shape and position of the breasts  * Arm lift to remove excess skin and tighten the underlying muscles  * Thigh lift to remove excess skin and tighten the underlying muscles  * Facelift to improve the appearance of the face and neck  The need for plastic surgery after weight loss surgery varies from person to person. Some people may have only a small amount of excess skin, while others may have a significant amount. The amount of excess skin is often determined by the person's genetics, the amount of weight they lost, and the rate at which they lost weight.  The best time to have plastic surgery after weight loss surgery is typically 12-18 months after the initial surgery. This gives the body time to adjust to the new weight and for the skin to have a chance to tighten on its own. However, some people may need to wait longer, depending on their individual circumstances.  Plastic surgery after weight loss surgery can be a very rewarding experience. It can help to improve the patient's self-confidence and overall quality of life. However, it is important to remember that plastic surgery is not a magic bullet. It cannot erase all of the changes that have occurred as a result of weight loss. It can only help to improve the appearance of the skin and underlying tissues.  If you are considering plastic surgery after weight loss surgery, it is important to talk to your doctor and a plastic surgeon to discuss your individual needs and goals. They can help you to determine if plastic surgery is right for you and can recommend the best procedures for your situation. | 7/9/2023 | Sure. Here's a simpler explanation:  * When you lose a lot of weight, your skin may not be able to tighten back up. This can leave you with excess skin and other problems, like saggy breasts or a droopy stomach.  * About 70-90% of people who have weight loss surgery will eventually need some form of plastic surgery to fix these problems.  * The most common plastic surgery procedures after weight loss surgery are tummy tucks, breast lifts, arm lifts, thigh lifts, and facelifts.  * The best time to have plastic surgery after weight loss surgery is typically 12-18 months after the initial surgery.  * Plastic surgery after weight loss surgery can help to improve your self-confidence and overall quality of life.  Here are some additional things to keep in mind:  * Plastic surgery is not a magic bullet. It cannot erase all of the changes that have occurred as a result of weight loss.  * Plastic surgery is a major surgery with risks, such as infection, bleeding, and scarring.  * You should talk to your doctor and a plastic surgeon to discuss your individual needs and goals before deciding if plastic surgery is right for you.  I hope this helps! |
| 3 | Will I lose hair after bariatric surgery? | For the reasons we still don’t fully understand, hair loss after bariatric surgery is common between 3 and 6 months following surgery. However, hair loss is almost always temporary. Adequate intake of protein, vitamins, biotin and minerals will help to ensure hair re-growth, and avoid longer term thinning. |  | There is a chance that you may experience hair loss after bariatric surgery. This is a common side effect that affects up to 57% of patients. The hair loss usually starts 3-5 months after surgery and can last for up to 6 months. It is not permanent, and your hair will grow back.  There are a few reasons why hair loss can happen after bariatric surgery. One reason is that the surgery can cause a lot of stress on your body. This stress can lead to a condition called telogen effluvium, which is a type of hair loss that happens when the hair follicles go into a resting phase.  Another reason for hair loss after bariatric surgery is that the surgery can cause you to lose weight rapidly. This rapid weight loss can lead to a deficiency in certain nutrients, such as iron, vitamin B12, and zinc. These nutrients are important for hair growth, so a deficiency can lead to hair loss.  Finally, some bariatric surgeries, such as gastric sleeve and gastric bypass, can cause changes in your digestive system. These changes can make it difficult to absorb certain nutrients, which can also lead to hair loss.  If you are concerned about hair loss after bariatric surgery, talk to your doctor. They can help you to determine the cause of your hair loss and recommend ways to prevent or minimize it.  Here are some tips to help prevent or minimize hair loss after bariatric surgery:  * Make sure that you are getting enough protein in your diet. Protein is essential for hair growth, so it is important to make sure that you are getting enough of it.  * Take a multivitamin that contains iron, vitamin B12, and zinc. These nutrients are important for hair growth, so taking a supplement can help to prevent or minimize hair loss.  * Avoid crash dieting or rapid weight loss. Rapid weight loss can lead to a deficiency in nutrients, which can cause hair loss.  * Manage stress. Stress can also lead to hair loss, so it is important to find ways to manage stress.  If you do experience hair loss after bariatric surgery, it is important to be patient. The hair loss is usually temporary and your hair will grow back. In the meantime, there are a few things you can do to help your hair look its best. You can use a gentle shampoo and conditioner, and avoid using heat styling tools on your hair. You can also try using a leave-in conditioner or styling product to help detangle your hair and make it look fuller. | 7/9/2023 | Sure. Here is a simpler explanation of why you might lose hair after bariatric surgery:  * The surgery can be stressful for your body, and this stress can cause your hair follicles to go into a resting phase. This means that your hair will stop growing and eventually fall out.  * The surgery can also cause you to lose weight rapidly, and this can lead to a deficiency in certain nutrients that are important for hair growth.  * Some bariatric surgeries can also change the way your body absorbs nutrients, which can also lead to a deficiency in nutrients that are important for hair growth.  The good news is that hair loss after bariatric surgery is usually temporary. Your hair will start to grow back in a few months, and it should be back to normal within a year. In the meantime, there are a few things you can do to help your hair look its best, such as using a gentle shampoo and conditioner, avoiding heat styling tools, and using a leave-in conditioner or styling product.  Here are some tips to help prevent or minimize hair loss after bariatric surgery:  * Make sure that you are getting enough protein in your diet.  * Take a multivitamin that contains iron, vitamin B12, and zinc.  * Avoid crash dieting or rapid weight loss.  * Manage stress.  If you do experience hair loss after bariatric surgery, it is important to be patient. The hair loss is usually temporary and your hair will grow back. |
| 3 | Do I need to take vitamins and minerals after surgery? | You will need to take a multivitamin for life. You may need higher doses of certain vitamins or minerals, especially Iron, Calcium, and Vitamin D. You will also need to have at least yearly lab checks. Insurance almost never pays for vitamin and mineral supplements but usually does pay for labs. You can pay for supplements out of a flex medical account. | Do I need to take vitamins and minerals after weight loss surgery? | Yes, you need to take vitamins and minerals after weight loss surgery. The reason for this is that the surgery can interfere with your body's ability to absorb certain nutrients. This is especially true for vitamin B12, calcium, and iron.  The specific vitamins and minerals that you need to take will depend on the type of surgery you had. However, some common supplements that are recommended after weight loss surgery include:  * A complete multivitamin  * Calcium with vitamin D  * Vitamin B12  * Iron  * Vitamin D  It is important to talk to your doctor or dietitian about which supplements are right for you. They can help you create a personalized plan that meets your individual needs.  Here are some tips for taking vitamins and minerals after weight loss surgery:  * Take your supplements with food. This will help your body absorb them better.  * Choose chewable or liquid forms of supplements. These may be easier to tolerate than tablets or capsules.  * Spread out your doses throughout the day. This will help your body absorb the nutrients better.  * Get your blood levels checked regularly. This will help you make sure that you are getting enough of the nutrients you need.  Taking vitamins and minerals after weight loss surgery is an important part of maintaining your health. By following these tips, you can ensure that you are getting the nutrients you need to stay healthy and strong. | 7/9/2023 | Sure. After weight loss surgery, your body may not be able to absorb all the nutrients it needs from food. This is because the surgery can change the way your body digests and absorbs food. To make sure you get the nutrients you need, you will need to take vitamins and minerals.  The specific vitamins and minerals you need will depend on the type of surgery you had. However, some common supplements that are recommended after weight loss surgery include:  * A complete multivitamin  * Calcium with vitamin D  * Vitamin B12  * Iron  * Vitamin D  You can take these supplements in chewable or liquid form. It is important to take your supplements with food to help your body absorb them better. You should also spread out your doses throughout the day.  It is also important to get your blood levels checked regularly to make sure you are getting enough of the nutrients you need.  Here is a simplified list of the vitamins and minerals you may need to take after weight loss surgery:  * **Multivitamin:** This will help you get a variety of nutrients in one supplement.  * **Calcium with vitamin D:** This will help keep your bones strong.  * **Vitamin B12:** This is important for energy production and brain function.  * **Iron:** This is important for carrying oxygen throughout your body.  * **Vitamin D:** This is important for bone health and immune function.  It is important to talk to your doctor or dietitian about which supplements are right for you. They can help you create a personalized plan that meets your individual needs. |
| 3 | If my insurance company will not pay for the surgery, can I pay the surgery myself? | We do offer bariatric surgery for patients who want to pay cash. Please call our office to get a quote. | If my insurance company will not pay for weight loss surgery, can I pay the surgery myself? | Yes, you can pay for weight loss surgery yourself if your insurance company will not cover it. The cost of weight loss surgery varies depending on the type of surgery, the surgeon, and the location, but it can range from $10,000 to $30,000. There are a few ways to pay for weight loss surgery if you do not have insurance coverage:  * **Use your health savings account (HSA).** If you have an HSA, you can use the money in your account to pay for weight loss surgery.  * **Take out a medical loan.** There are a number of medical loan providers that offer loans specifically for weight loss surgery.  * **Borrow money from family or friends.** If you have family or friends who are willing to lend you money, this can be a good option.  * **Use a credit card.** You can use a credit card to pay for weight loss surgery, but you will need to make sure that you can afford to pay off the balance before the interest charges start to accrue.  It is important to talk to your doctor about the cost of weight loss surgery before you make a decision. They can help you estimate the cost of the surgery and discuss the different payment options with you.  Here are some additional tips for paying for weight loss surgery:  * **Start saving early.** If you know that you want to have weight loss surgery, start saving money as early as possible. This will give you time to build up a nest egg and make the payments more manageable.  * **Shop around for the best price.** The cost of weight loss surgery can vary depending on the surgeon, the facility, and the location. Do some research to find the best price for the type of surgery that you want.  * **Consider financing options.** There are a number of financing options available for weight loss surgery. Talk to your doctor or a financial advisor to find the best option for you.  Weight loss surgery is a major decision, and the cost of the surgery is a factor that you will need to consider. However, if you are committed to losing weight and improving your health, the cost of surgery may be worth it. | 7/9/2023 | Sure. If your insurance company won't pay for weight loss surgery, you can pay for it yourself. It will cost between $10,000 and $30,000. You can use your health savings account (HSA), take out a medical loan, borrow money from family or friends, or use a credit card to pay for the surgery.  Here are some tips for paying for weight loss surgery:  * Start saving early.  * Shop around for the best price.  * Consider financing options.  Weight loss surgery is a major decision, but the cost of surgery may be worth it if you are committed to losing weight and improving your health.  Here is a table that summarizes the different payment options for weight loss surgery:  \| Payment Option \| Description \|  \|---\|---\|  \| Health savings account (HSA) \| A tax-advantaged account that can be used to pay for medical expenses, including weight loss surgery. \|  \| Medical loan \| A loan specifically designed to pay for medical expenses, including weight loss surgery. \|  \| Borrow money from family or friends \| A good option if you have family or friends who are willing to lend you money. \|  \| Credit card \| You can use a credit card to pay for weight loss surgery, but you will need to make sure that you can afford to pay off the balance before the interest charges start to accrue. \|  I hope this helps! |
| 3 | Will bariatric surgery last? | Many patients (up to 50%) may regain a small amount of weight (approximately 5 percent) two years or more following their surgery.  However, longitudinal studies find that most bariatric surgery patients maintain successful weight-loss long-term.  One study summarizing the results from 22 reports with a total of 4206 patient cases reported good long-term durability of gastric bypass surgery.  The average excess weight loss was 66.5%, and there was no significant association between excess weight loss and length of follow-up.  Now we have data to support the durability of gastric bypass surgery up to 15 years.  Studies also have shown that sleeve gastrectomy patients were able to maintain its well-documented weight loss outcome at 5 or more years after surgery, with the overall mean excess weight loss at 5 or more years still remaining>50%.  Such significant and sustained weight loss after surgery is in sharp contrast to medical management |  | Bariatric surgery is a major medical procedure that can have a lasting impact on your health. The effects of bariatric surgery can last for many years, and in some cases, even a lifetime.  The most common type of bariatric surgery is gastric bypass, which involves rerouting the stomach and small intestine. This surgery can help you lose a significant amount of weight, and studies have shown that the weight loss can last for many years. In fact, one study found that people who had gastric bypass surgery were able to maintain a 50% loss of excess weight for up to 20 years.  Of course, not everyone who has bariatric surgery is able to maintain their weight loss for the long term. There are a number of factors that can contribute to weight regain, including not following the recommended diet and exercise plan, and developing other health conditions.  However, even if you do regain some weight after bariatric surgery, you are still likely to be at a lower weight than you were before the surgery. This can still have significant health benefits, such as reducing your risk of heart disease, stroke, type 2 diabetes, and other obesity-related conditions.  Overall, bariatric surgery can be a very effective way to lose weight and improve your health. The effects of the surgery can last for many years, and in some cases, even a lifetime. However, it is important to follow the recommended diet and exercise plan after surgery in order to maximize your chances of success.  Here are some additional things to keep in mind about bariatric surgery:  * It is not a cure for obesity.  * It is a major medical procedure with risks and side effects.  * You will need to make permanent lifestyle changes after surgery, such as following a healthy diet and exercising regularly.  * You will need to see your doctor regularly after surgery for monitoring and care.  If you are considering bariatric surgery, it is important to talk to your doctor about the risks and benefits. They can help you decide if surgery is the right choice for you. | 7/9/2023 | Sure. Bariatric surgery is a way to lose weight that can last for many years. It is not a cure for obesity, but it can help you lose a significant amount of weight and improve your health.  The most common type of bariatric surgery is gastric bypass. This surgery involves rerouting your stomach and small intestine. This makes you feel full faster and prevents you from absorbing as many calories.  After bariatric surgery, you will need to make permanent lifestyle changes. This includes following a healthy diet and exercising regularly. You will also need to see your doctor regularly for monitoring and care.  If you are considering bariatric surgery, it is important to talk to your doctor about the risks and benefits. They can help you decide if surgery is the right choice for you.  Here is a table that summarizes the pros and cons of bariatric surgery:  \| Pros \| Cons \|  \|---\|---\|  \| Can help you lose a significant amount of weight \| It is a major medical procedure with risks and side effects. \|  \| Can improve your health \| You will need to make permanent lifestyle changes after surgery. \|  \| Can last for many years \| You will need to see your doctor regularly after surgery. \|  I hope this helps! |
| 4 | How effective is bariatric surgery? | Bariatric surgery is considered to be the most effective treatment for obesity in terms of sustained weight loss and improvement in other obesity-related conditions. The amount of weight you lose depends on the type of bariatric surgery and lifestyle habits following surgery. It is important to keep all scheduled follow-up visits so that your care team can monitor your weight loss.  The benefits of bariatric surgery include:  - An improved quality of life  - A reduced risk of other obesity-related diseases such as cardiovascular disease, sleep apnea, and type 2 diabetes  - Improved blood pressure  - A longer lifespan |  | Bariatric surgery is a very effective treatment for obesity. Studies have shown that people who undergo bariatric surgery can lose an average of 50-70% of their excess weight. This can lead to significant improvements in health, including:  * Reduced risk of heart disease, stroke, and type 2 diabetes  * Improved blood pressure and cholesterol levels  * Reduced risk of sleep apnea  * Improved joint pain  * Increased energy levels  * Improved quality of life  The success rate of bariatric surgery is high, but it is not 100%. Some people may not lose as much weight as they hoped, and some may regain some weight over time. However, even if you do not reach your ideal weight, bariatric surgery can still be a life-changing treatment.  Here are some of the factors that can affect the success of bariatric surgery:  * The type of surgery you have  * Your initial weight  * Your commitment to making lifestyle changes  * Your overall health  If you are considering bariatric surgery, it is important to talk to your doctor about your individual risks and benefits. Bariatric surgery is a major surgery, and it is not right for everyone. However, for people who are obese and have not been able to lose weight through other means, it can be a very effective treatment.  Here are some additional resources that you may find helpful:  * The American Society for Metabolic and Bariatric Surgery: https://asmbs.org/  * The National Institute of Diabetes and Digestive and Kidney Diseases: https://www.niddk.nih.gov/health-information/weight-management/bariatric-surgery  * The Obesity Action Coalition: https://www.obesityaction.org/ | 7/8/2023 | Sure. Bariatric surgery is a type of surgery that can help people who are obese lose weight. It works by making it harder to eat or absorb food. This can lead to significant weight loss, which can improve health and quality of life.  The success rate of bariatric surgery is high, but it is not 100%. Some people may not lose as much weight as they hoped, and some may regain some weight over time. However, even if you do not reach your ideal weight, bariatric surgery can still be a life-changing treatment.  Here is an example of how bariatric surgery can work:  * Gastric bypass surgery: This surgery creates a small pouch at the top of the stomach and then connects it directly to the small intestine. This makes it so that you can only eat a small amount of food at a time, and you absorb fewer calories from the food you eat.  * Sleeve gastrectomy: This surgery removes about 80% of the stomach. This makes it so that you can only eat a small amount of food at a time, and you absorb fewer calories from the food you eat.  Both of these surgeries are effective in helping people lose weight. However, they are also major surgeries, and they come with some risks. It is important to talk to your doctor about the risks and benefits of bariatric surgery before you decide if it is right for you.  Here are some of the risks of bariatric surgery:  * Infection  * Bleeding  * Leakage from the stomach or intestine  * Nutritional deficiencies  * Death  It is important to note that these are just some of the risks of bariatric surgery. There are other risks that may not be listed here. It is important to talk to your doctor about all of the risks before you decide if bariatric surgery is right for you.  I hope this helps! |
| 4 | Is bariatric surgery safe? | While any surgical procedure has risks, bariatric surgery has been found to be one of the safest surgeries to undergo in comparison with other elective procedures. The Mass General Weight Center team performs a high volume of weight loss surgeries, which studies have shown relates to lower complication rates and improved outcomes related to quality and safety.  It is important to discuss any concerns you may have with your bariatric surgery care team. They will review and answer all your questions ahead of surgery. |  | Bariatric surgery is a major surgery, and as with any surgery, there are risks involved. However, bariatric surgery is considered to be a safe procedure, and the risks are relatively low. In fact, the risks of bariatric surgery are often lower than the risks of living with obesity.  According to the American Society for Metabolic and Bariatric Surgery (ASMBS), the risk of death within 30 days of bariatric surgery is about 0.13%. This is lower than the risk of death for other common surgeries, such as gallbladder surgery (0.7%) and hip replacement surgery (0.93%).  The most common risks of bariatric surgery include:  * Bleeding  * Infection  * Blood clots  * Leaks in the gastrointestinal system  * Nutritional deficiencies  * Dumping syndrome  These risks are usually minor and can be treated. However, in rare cases, they can be serious or even life-threatening.  The benefits of bariatric surgery often outweigh the risks. Bariatric surgery can help people lose a significant amount of weight, which can improve their health in many ways. For example, bariatric surgery can:  * Reduce the risk of heart disease, stroke, and type 2 diabetes  * Improve sleep apnea  * Improve joint pain  * Increase energy levels  * Improve self-esteem  If you are considering bariatric surgery, it is important to talk to your doctor about the risks and benefits. Your doctor can help you decide if bariatric surgery is right for you.  Here are some additional resources that you may find helpful:  * American Society for Metabolic and Bariatric Surgery: https://www.asbmb.org/  * National Institute of Diabetes and Digestive and Kidney Diseases: https://www.niddk.nih.gov/health-information/weight-management/bariatric-surgery  * Mayo Clinic:<https://www.mayoclinic.org/tests-procedures/bariatric-surgery/about/pac-20394258> | 7/8/2023 | Sure. Bariatric surgery is a safe procedure that can help people lose a significant amount of weight. The risks of bariatric surgery are relatively low, and the benefits often outweigh the risks. If you are considering bariatric surgery, it is important to talk to your doctor about the risks and benefits.  Here are some of the risks of bariatric surgery in simpler terms:  * **Bleeding:** This is when blood leaks out of a blood vessel during or after surgery. It can be a serious problem, but it is usually treatable.  * **Infection:** This is when bacteria get into the body and cause an infection. Infections can be serious, but they are usually treatable with antibiotics.  * **Blood clots:** This is when a blood clot forms in a blood vessel. Blood clots can be dangerous if they travel to the lungs or brain.  * **Leaks in the gastrointestinal system:** This is when a hole develops in the stomach or intestines. Leaks can be serious and can lead to infection.  * **Nutritional deficiencies:** This is when the body does not get enough nutrients. Nutritional deficiencies can be serious and can lead to health problems.  * **Dumping syndrome:** This is a condition that can occur after bariatric surgery. It causes symptoms such as nausea, vomiting, and diarrhea.  If you have any questions about the risks of bariatric surgery, please talk to your doctor. |
| 4 | When can I expect to return to daily life after weight loss surgery? | Patients generally are able to stand and walk a few hours after surgery and are discharged within one to two days. Avoiding high impact exercise and heavy weight lifting is recommended for at least one month, with an emphasis on listening to your body and resting as needed. Many patients return to work about four weeks after surgery.  Because of a lower calorie intake, many patients feel a lower energy than usual for a couple of weeks after surgery. Energy tends to elevate after transitioning from a liquid diet to solid foods. |  | The time it takes to return to daily life after weight loss surgery varies from person to person. However, in general, most people are able to return to work and other activities within 2-4 weeks after surgery.  Here is a general timeline of what to expect after weight loss surgery:  * **Day 1-2:** You will likely stay in the hospital for 1-2 days after surgery. During this time, you will be monitored closely for any complications.  * **Week 1:** You will start on a liquid diet and gradually transition to soft foods. You will also start walking and doing light exercises.  * **Weeks 2-4:** You will be able to resume most of your normal activities, including work, school, and exercise. However, you will still need to be careful not to overexert yourself.  * **Months 1-6:** You will continue to lose weight and adjust to your new diet and lifestyle. You may also experience some side effects, such as nausea, vomiting, and constipation.  * **Years 1+:** You will continue to lose weight and maintain your weight loss. You will also reduce your risk of developing obesity-related health problems, such as heart disease, stroke, and diabetes.  It is important to follow your doctor's instructions carefully after weight loss surgery. This will help you have a smooth recovery and achieve your weight loss goals.  Here are some tips for returning to daily life after weight loss surgery:  * **Take things slowly:** Don't try to do too much too soon. Start with short walks and gradually increase your activity level.  * **Listen to your body:** If you feel tired or sore, take a break. Don't push yourself too hard.  * **Stay hydrated:** Drink plenty of fluids, especially water.  * **Eat a healthy diet:** Make sure you are getting enough protein, fruits, vegetables, and whole grains.  * **Get enough sleep:** Aim for 7-8 hours of sleep per night.  * **Manage stress:** Find healthy ways to manage stress, such as exercise, yoga, or meditation.  With careful planning and adherence to your doctor's instructions, you can expect to return to daily life after weight loss surgery within a few weeks. | 7/9/2023 | Sure. After weight loss surgery, you will need to take it easy for a few weeks. You will stay in the hospital for 1-2 days, and then you will start a liquid diet. You will gradually transition to soft foods, and then you will be able to resume most of your normal activities within 2-4 weeks. However, you will still need to be careful not to overexert yourself.  Here are some simple tips to follow after weight loss surgery:  * **Don't try to do too much too soon.** Start with short walks and gradually increase your activity level.  * **Listen to your body.** If you feel tired or sore, take a break. Don't push yourself too hard.  * **Stay hydrated.** Drink plenty of fluids, especially water.  * **Eat a healthy diet.** Make sure you are getting enough protein, fruits, vegetables, and whole grains.  * **Get enough sleep.** Aim for 7-8 hours of sleep per night.  Following these tips will help you have a smooth recovery and achieve your weight loss goals. |
| 5 | What are the risks of the operation? | Weight loss surgery is major abdominal surgery with significant risks. However, we know experience counts and high-volume centers like X have an extremely low complications rate.  The following potential complications include, but are not limited to:  - Risk from general anesthesia  - Bleeding  - Infection  - Pneumonia  - Blood clot in the legs or lungs  - Hernias (internally or externally)  - Blockage or obstruction  - Narrowing where surgeons suture the pouch to the intestine, which can require another endoscopy and dilation  - Leakage from the stomach or the intestines  - Injury to liver and/or spleen  - Pulmonary and/or cardiac and/or renal failure  With the adjustable gastric band (lap-band), there's a risk that the band can either slip or erode. There's a risk that you will not lose weight if you don't follow the diet and exercise program. There's a risk of needing another operation or conversion to an open operation with a long incision. While there's a risk of death with each procedure: 1-in-200 with the gastric bypass, 1-in-500 with sleeve gastrectomy, and 1-in-10,000 with the lap band. | What are the risks of weight loss surgery? | Weight loss surgery, also known as bariatric surgery, is a major medical procedure that can help people with severe obesity lose weight and improve their health. However, like any surgery, it carries some risks.  The most serious risks of weight loss surgery include:  * **Bowel leaks.** This is a rare but serious complication that can occur after any type of bariatric surgery. A bowel leak occurs when there is a hole in the intestine. This can lead to infection, sepsis, and even death.  * **Nutritional deficiencies.** Weight loss surgery can make it difficult to absorb nutrients from food. This can lead to deficiencies in vitamins, minerals, and other essential nutrients.  * **Gallstones.** Rapid weight loss can increase the risk of developing gallstones.  * **Hernias.** A hernia is a bulge in the abdominal wall. It can occur after any type of bariatric surgery, but it is more common after gastric bypass surgery.  * **Other complications.** Other complications of weight loss surgery can include:  * **Anemia**  * **Heartburn**  * **Diarrhea**  * **Constipation**  * **Hair loss**  * **Skin problems**  The risks of weight loss surgery vary depending on the type of surgery. Some types of surgery, such as gastric sleeve surgery, are generally considered to be safer than others, such as gastric bypass surgery.  It is important to talk to your doctor about the risks of weight loss surgery before you decide if it is right for you. Your doctor can help you weigh the risks and benefits of surgery and determine if you are a good candidate.  Here are some additional things to consider when thinking about the risks of weight loss surgery:  * Your age and overall health.  * Your motivation to lose weight and keep it off.  * Your support system.  * Your expectations for weight loss surgery.  If you are considering weight loss surgery, it is important to do your research and talk to your doctor about the risks and benefits. With careful planning and preparation, you can reduce your risk of complications and improve your chances of a successful outcome. | 7/8/2023 | Sure. Weight loss surgery is a big decision, and it's important to understand the risks involved. Here are some of the most common risks in simple terms:  * **Bowel leaks:** This is a hole in the intestine that can be caused by surgery. It's a serious complication, but it's rare.  * **Nutritional deficiencies:** Weight loss surgery can make it harder to absorb nutrients from food. This can lead to problems like anemia and hair loss.  * **Gallstones:** Rapid weight loss can increase the risk of developing gallstones. These are small stones that can form in the gallbladder.  * **Hernias:** A hernia is a bulge in the abdominal wall. It can happen after any type of weight loss surgery, but it's more common after gastric bypass surgery.  Other complications of weight loss surgery can include diarrhea, constipation, and skin problems.  The risks of weight loss surgery vary depending on the type of surgery. Some types of surgery, such as gastric sleeve surgery, are generally considered to be safer than others, such as gastric bypass surgery.  It's important to talk to your doctor about the risks of weight loss surgery before you decide if it's right for you. Your doctor can help you weigh the risks and benefits of surgery and determine if you are a good candidate.  I hope this helps! |
| 5 | How successful are these operations? | The gastric bypass operation allows you to lose up to 82% of your excess body weight, however it's almost 100% effective in reversing obesity related co-morbidities including sleep apnea, hypertension and diabetes. The adjustable gastric banding (lap-band) operation allows you to lose 50% of your excess body weight at two years. It's also effective in reversing obesity related co-morbidities. The sleeve gastrectomy is between the gastric bypass and gastric banding in terms of weight loss with 60% excess weight loss at 1 year. | How successful are the various weight loss surgeries? | The success of weight loss surgery varies depending on the type of surgery, the patient's individual characteristics, and the follow-up care they receive. In general, however, weight loss surgery is considered to be a very successful treatment for obesity.  Here is a brief overview of the success rates of some of the most common weight loss surgeries:  * **Gastric bypass:** This surgery is the most effective weight loss surgery in terms of long-term weight loss. Studies have shown that patients who have gastric bypass surgery can lose an average of 50-70% of their excess weight and keep it off for at least 5 years.  * **Laparoscopic sleeve gastrectomy:** This surgery is also very effective for weight loss. Patients who have laparoscopic sleeve gastrectomy can lose an average of 50-60% of their excess weight and keep it off for at least 5 years.  * **Biliopancreatic diversion with duodenal switch (BPD/DS):** This surgery is the most effective weight loss surgery for patients with severe obesity. Patients who have BPD/DS can lose an average of 70-80% of their excess weight and keep it off for at least 5 years.  It is important to note that weight loss surgery is not a magic bullet. It is a tool that can help people lose weight, but it is not a guarantee of success. Patients who have weight loss surgery still need to make lifestyle changes, such as eating a healthy diet and exercising regularly, in order to maintain their weight loss.  The success rate of weight loss surgery is also affected by the patient's individual characteristics. For example, patients who are younger and have a lower BMI tend to be more successful at maintaining their weight loss than older patients and patients with a higher BMI.  Finally, the success of weight loss surgery is also affected by the follow-up care that patients receive. Patients who have weight loss surgery need to see their doctor regularly for check-ups and to make sure that they are getting the nutrients they need. They also need to be aware of the potential risks and complications of weight loss surgery and to take steps to prevent them.  Overall, weight loss surgery is a very effective treatment for obesity. However, it is important to remember that it is not a magic bullet and that patients still need to make lifestyle changes in order to maintain their weight loss. | 7/8/2023 | Sure. Weight loss surgery is a major operation that can help people lose a lot of weight. The different types of weight loss surgery work in different ways, but they all have the same goal: to make it harder for people to eat too much.  The most successful weight loss surgery is gastric bypass. This surgery makes the stomach smaller and also changes the way the small intestine absorbs food. Patients who have gastric bypass surgery can lose an average of 50-70% of their excess weight and keep it off for at least 5 years.  Other types of weight loss surgery, such as laparoscopic sleeve gastrectomy and biliopancreatic diversion with duodenal switch, are also effective for weight loss. However, they are not as successful as gastric bypass surgery.  It is important to remember that weight loss surgery is not a magic bullet. It is a tool that can help people lose weight, but it is not a guarantee of success. Patients who have weight loss surgery still need to make lifestyle changes, such as eating a healthy diet and exercising regularly, in order to maintain their weight loss.  Here is a table that summarizes the success rates of some of the most common weight loss surgeries:  \| Type of surgery \| Average weight loss \|  \|---\|---\|  \| Gastric bypass \| 50-70% \|  \| Laparoscopic sleeve gastrectomy \| 50-60% \|  \| Biliopancreatic diversion with duodenal switch \| 70-80% \|  As you can see, the success rates of different weight loss surgeries vary. However, all of these surgeries can help people lose a significant amount of weight. If you are considering weight loss surgery, it is important to talk to your doctor about the different options and what is best for you. |
| 5 | Why is there a 10% pre-operative weight loss required? | We require patients to begin a diet and exercise program that allows them to lose 10% of their weight pre-operatively for three reasons:  1) Patients who undergo weight loss before surgery have fewer complications during and after surgery.  2) This allows patients to truly understand the lifestyle changes that will occur after surgery and therefore make an informed consent about the diet and exercise programs that they will have to be on for the rest of their lives.  3) This allows us to identify the patients who are truly motivated and therefore who will be most successful following surgery. Most important, we'll work with you to develop a weight-loss program to achieve the 10% reduction goal. | Why is there a 10% pre-operative weight loss required for weight loss surgery? | There are a few reasons why a 10% pre-operative weight loss is required for weight loss surgery.  * **It can help to improve the patient's health.** Losing even a small amount of weight can help to reduce the risk of complications during surgery, such as heart problems, breathing problems, and blood clots. It can also improve the patient's overall health by lowering blood pressure, cholesterol levels, and blood sugar levels.  * **It can help the patient to learn healthy habits.** Losing weight before surgery can help the patient to learn how to make healthy changes to their diet and lifestyle. This can make it more likely that they will be successful in maintaining their weight loss after surgery.  * **It can help to determine if surgery is the right option.** Losing weight before surgery can help the surgeon to determine if the patient is a good candidate for surgery. If the patient is able to lose weight on their own, it is more likely that they will be able to lose weight and keep it off after surgery.  It is important to note that not all bariatric surgeons require a 10% pre-operative weight loss. Some surgeons may only require a 5% weight loss, while others may not require any pre-operative weight loss at all. The decision of whether or not to require a pre-operative weight loss is made on a case-by-case basis, taking into account the patient's individual health and circumstances.  Here are some of the benefits of losing weight before weight loss surgery:  * **Improved health:** Losing weight can help to reduce the risk of obesity-related health problems, such as heart disease, stroke, type 2 diabetes, and some types of cancer.  * **Easier surgery:** Losing weight before surgery can make the surgery itself easier and safer.  * **Faster recovery:** Patients who lose weight before surgery tend to have a faster recovery time.  * **More successful weight loss:** Patients who lose weight before surgery are more likely to keep the weight off long-term.  If you are considering weight loss surgery, talk to your doctor about whether or not you should lose weight before surgery. They can help you to create a safe and effective weight loss plan that is right for you. | 7/8/2023 | Sure. Here's a simpler explanation of why there is a 10% pre-operative weight loss required for weight loss surgery:  * **Losing weight can help to make the surgery safer.** When you are overweight or obese, you are more likely to have health problems that can make surgery more risky. For example, you may have high blood pressure, high cholesterol, or diabetes. Losing weight can help to improve these health problems and make the surgery safer.  * **Losing weight can help you to learn how to lose weight and keep it off.** After weight loss surgery, you will need to make changes to your diet and lifestyle in order to keep the weight off. Losing weight before surgery can help you to learn how to make these changes and make it more likely that you will be successful after surgery.  * **Losing weight can help you to decide if surgery is right for you.** If you are able to lose weight on your own, it is a good sign that you may be able to lose weight and keep it off after surgery. If you are not able to lose weight on your own, it may be a sign that surgery is not the right option for you.  In general, losing weight before weight loss surgery is a good idea. It can help to make the surgery safer, help you to learn how to lose weight and keep it off, and help you to decide if surgery is the right option for you.  Here are some tips for losing weight before weight loss surgery:  * Talk to your doctor about a safe and effective weight loss plan.  * Make changes to your diet. Eat healthier foods and smaller portions.  * Increase your physical activity. Aim for at least 30 minutes of moderate-intensity exercise most days of the week.  * Be patient and persistent. Losing weight takes time and effort.  If you follow these tips, you can lose weight before weight loss surgery and improve your chances of a successful outcome. |
| 5 | What is the conversion rate? | Conversion to open surgery is required in less than 1% of patients. The risk of conversion is higher in patients who have had previous upper abdominal surgery. | What is the conversion rate from laparoscopic to open, with regards to weight loss surgery? | The conversion rate from laparoscopic to open weight loss surgery varies depending on the type of surgery and the surgeon's experience. In general, the conversion rate is between 1% and 5%.  For example, a study published in the journal *Obesity Surgery* in 2015 found that the conversion rate for laparoscopic sleeve gastrectomy was 2.2%. Another study, published in the journal *Surgery* in 2016, found that the conversion rate for laparoscopic Roux-en-Y gastric bypass was 3.3%.  There are a number of factors that can increase the risk of conversion, including:  * High body mass index (BMI)  * Previous abdominal surgery  * Smoking  * Comorbidities such as diabetes or heart disease  * Surgeon's experience  If a patient does require conversion to open surgery, it is usually because of technical difficulties or complications that arise during the laparoscopic procedure. Open surgery is generally more invasive and has a longer recovery time than laparoscopic surgery. However, the outcomes for patients who have open weight loss surgery are still generally good.  It is important to talk to your surgeon about the conversion rate for the specific type of weight loss surgery you are considering. They can help you assess your individual risk factors and make sure you are aware of the potential benefits and risks of both laparoscopic and open surgery. | 7/8/2023 | Sure. Laparoscopic weight loss surgery is done through small incisions in the abdomen. Open weight loss surgery is done through a larger incision in the abdomen.  The conversion rate is the percentage of patients who have laparoscopic weight loss surgery but need to have the surgery converted to open surgery. The conversion rate for weight loss surgery is between 1% and 5%.  There are a number of factors that can increase the risk of conversion, including:  * Being very overweight  * Having had surgery on your abdomen before  * Smoking  * Having other health problems  * Having a surgeon who is not experienced in laparoscopic weight loss surgery  If you need to have your laparoscopic weight loss surgery converted to open surgery, it is usually because of technical difficulties or complications that arise during the laparoscopic procedure. Open surgery is generally more invasive and has a longer recovery time than laparoscopic surgery. However, the outcomes for patients who have open weight loss surgery are still generally good.  It is important to talk to your surgeon about the conversion rate for the specific type of weight loss surgery you are considering. They can help you assess your individual risk factors and make sure you are aware of the potential benefits and risks of both laparoscopic and open surgery.  In simpler terms, there is a small chance that you may need to have your laparoscopic weight loss surgery converted to open surgery. This is usually because of technical difficulties or complications that arise during the laparoscopic procedure. Open surgery is generally more invasive and has a longer recovery time than laparoscopic surgery, but the outcomes for patients who have open weight loss surgery are still generally good.  I hope this helps! Let me know if you have any other questions. |
| 5 | How do I know which operation is for me? | Deciding which procedure is best for you is largely a personal one best achieved in discussion with your surgeon. | How do I know which type of weight loss surgery operation is for me? | There are many factors to consider when choosing a type of weight loss surgery, including your:  * Body mass index (BMI)  * Health conditions  * Eating habits  * Lifestyle  * Risk tolerance  Your doctor will discuss all of these factors with you and help you decide which surgery is right for you. Here is a brief overview of the most common types of weight loss surgery:  * **Gastric bypass:** This is the most common type of weight loss surgery. It involves creating a small pouch at the top of the stomach and then rerouting food directly into the small intestine. This surgery limits the amount of food you can eat and also reduces the absorption of calories.  * **Sleeve gastrectomy:** This surgery removes about 80% of the stomach, leaving a small, banana-shaped pouch. This surgery limits the amount of food you can eat and also makes you feel full more quickly.  * **Gastric banding:** This surgery involves placing a band around the upper part of the stomach to create a small pouch. The band can be adjusted to control the amount of food you can eat.  * **Duodenal switch:** This surgery is a more complex procedure that involves dividing the stomach and rerouting food directly into the small intestine. This surgery limits the amount of food you can eat and also reduces the absorption of calories.  There are other types of weight loss surgery as well, but these are the most common. Your doctor will be able to tell you more about each procedure and help you decide which one is right for you.  It is important to remember that weight loss surgery is not a quick fix. It is a tool that can help you lose weight, but you will still need to make lifestyle changes, such as eating a healthy diet and exercising regularly. If you are considering weight loss surgery, talk to your doctor about all of your options and make sure you are fully prepared for the procedure and the changes that will follow.  Here are some additional resources that you may find helpful:  * American Society for Metabolic and Bariatric Surgery: https://www.asMBS.org/  * National Institute of Diabetes and Digestive and Kidney Diseases: https://www.niddk.nih.gov/health-information/weight-management/bariatric-surgery  * Mayo Clinic:<https://www.mayoclinic.org/tests-procedures/bariatric-surgery/about/pac-20394258> | 7/8/2023 | Sure. There are four main types of weight loss surgery: gastric bypass, sleeve gastrectomy, gastric banding, and duodenal switch.  * **Gastric bypass** is the most common type of weight loss surgery. It works by creating a small pouch at the top of the stomach and then rerouting food directly into the small intestine. This surgery limits the amount of food you can eat and also reduces the absorption of calories.  * **Sleeve gastrectomy** is a less invasive surgery than gastric bypass. It removes about 80% of the stomach, leaving a small, banana-shaped pouch. This surgery limits the amount of food you can eat and also makes you feel full more quickly.  * **Gastric banding** is a minimally invasive surgery that involves placing a band around the upper part of the stomach to create a small pouch. The band can be adjusted to control the amount of food you can eat.  * **Duodenal switch** is a more complex procedure than the other three types of surgery. It divides the stomach and reroutes food directly into the small intestine. This surgery limits the amount of food you can eat and also reduces the absorption of calories.  The best type of weight loss surgery for you will depend on your individual needs and circumstances. Your doctor will be able to help you decide which surgery is right for you.  Here is a table that summarizes the different types of weight loss surgery:  \| Type of surgery \| Description \|  \|---\|---\|  \| Gastric bypass \| Creates a small pouch at the top of the stomach and then reroutes food directly into the small intestine. \|  \| Sleeve gastrectomy \| Removes about 80% of the stomach, leaving a small, banana-shaped pouch. \|  \| Gastric banding \| Places a band around the upper part of the stomach to create a small pouch. The band can be adjusted to control the amount of food you can eat. \|  \| Duodenal switch \| Divides the stomach and reroutes food directly into the small intestine. \|  It is important to remember that weight loss surgery is not a quick fix. It is a tool that can help you lose weight, but you will still need to make lifestyle changes, such as eating a healthy diet and exercising regularly. If you are considering weight loss surgery, talk to your doctor about all of your options and make sure you are fully prepared for the procedure and the changes that will follow.  I hope this helps! |
| 5 | What will happen when I’m in the hospital? | In general, you'll be in the hospital for two nights. We'll expect you to get up and walk while you're in the hospital – this is your best defense against blood clots. You'll meet with the dietician before going home and while you're here, you'll have a clear liquid diet that will be limited to a medicine cup every 15 minutes.  Once home, you can drink until full. Please walk, drink once every hour, and take in at least 40 - 60 grams of protein daily for women, and 60 - 80 grams of protein daily for men | What will happen when I’m in the hospital after weight loss surgery? | Here is what you can expect to happen when you are in the hospital after weight loss surgery:  * You will be closely monitored by your medical team. They will check your vital signs, such as your blood pressure, heart rate, and temperature, regularly. They will also watch for any signs of complications, such as bleeding, infection, or nausea and vomiting.  * You will receive pain medication to help you manage your pain. You may also receive other medications, such as antibiotics, to prevent infection.  * You will be given clear liquids to drink. You may also be able to have small amounts of ice chips or popsicles.  * You will be encouraged to get up and move around as soon as possible. This will help to prevent blood clots and other complications.  * You will be discharged from the hospital when your medical team feels that you are stable and able to care for yourself at home.  Here is a more detailed timeline of what you can expect after weight loss surgery:  * **Day 1:** You will wake up in the recovery room after surgery. You will be attached to an IV for fluids and pain medication. You may also have a nasogastric (NG) tube in your nose to drain your stomach.  * **Day 2:** You will start to eat clear liquids. You will also start to walk around the hospital room. The NG tube will be removed.  * **Day 3:** You will be discharged from the hospital. You will be given instructions on how to care for your incisions and how to manage your pain. You will also be given a list of follow-up appointments.  It is important to follow your doctor's instructions carefully after weight loss surgery. This will help you to have a safe and successful recovery.  Here are some additional tips for recovering from weight loss surgery:  * Get plenty of rest.  * Drink plenty of fluids.  * Eat small, frequent meals.  * Avoid lifting heavy objects.  * Stay active.  * Manage your stress levels.  With proper care, you can expect to make a full recovery from weight loss surgery and start living a healthier life. | 7/8/2023 | Sure. Here is a simpler explanation of what will happen when you are in the hospital after weight loss surgery:  * You will be watched closely by doctors and nurses. They will make sure you are not in pain and that your surgery went well.  * You will be given medicine to help you feel better. You may also get medicine to prevent infection.  * You will only be able to drink clear liquids at first. You will start to eat real food after a few days.  * You will be encouraged to get up and move around. This will help you heal faster.  * You will stay in the hospital for a few days. Then, you will be able to go home.  Here are some additional tips for recovering from weight loss surgery:  * Get plenty of rest.  * Drink plenty of water.  * Eat small meals often.  * Do not lift heavy objects.  * Stay active.  * Manage your stress levels.  Following these tips will help you recover from weight loss surgery quickly and safely.  I hope this explanation is helpful. Please let me know if you have any other questions. |
| 5 | Is there an age restriction for the surgery? | Surgery is done on patients between the ages of 18 - 65. If you're younger than 18, please refer to our Adolescent Bariatric Surgery Program at Lucile Packard Children's Hospital. If you are older than 65, cases are evaluated on a case by case basis. Please keep in mind your insurance may restrict your options. | Is there an age restriction for weight loss surgery? | The age restriction for weight loss surgery varies depending on the country and the specific type of surgery. In the United States, the minimum age for weight loss surgery is typically 18 years old. However, some surgeons may consider younger patients who have tried other weight loss methods and have not been successful. The upper age limit for weight loss surgery is not as well-defined, but it is generally considered to be around 70 years old.  Here are some of the factors that surgeons consider when determining whether a patient is a good candidate for weight loss surgery, regardless of age:  * **Body mass index (BMI)**. BMI is a measure of body fat based on height and weight. A BMI of 40 or higher is considered to be morbidly obese, and this is usually the minimum BMI requirement for weight loss surgery.  * **Health conditions**. Weight loss surgery can help to improve a number of health conditions that are associated with obesity, such as type 2 diabetes, high blood pressure, and sleep apnea. Surgeons will want to make sure that the patient's health conditions are stable enough to withstand surgery.  * **Motivation**. Weight loss surgery is a major life change, and it is important that the patient is motivated to make lifestyle changes that will support their weight loss. Surgeons will want to make sure that the patient is committed to following through with the necessary dietary and exercise changes after surgery.  If you are considering weight loss surgery, it is important to talk to your doctor about your individual circumstances. They can help you to determine whether weight loss surgery is right for you and what the risks and benefits may be. | 7/8/2023 | Sure. Weight loss surgery is a major surgery, and there are some age restrictions in place to protect patients. In the United States, you must be at least 18 years old to get weight loss surgery. However, some surgeons may consider younger patients who have tried other weight loss methods and have not been successful. The upper age limit for weight loss surgery is not as well-defined, but it is generally considered to be around 70 years old.  There are other factors that surgeons consider when determining whether a patient is a good candidate for weight loss surgery, regardless of age. These factors include:  * **Your body mass index (BMI)**. BMI is a measure of body fat based on height and weight. A BMI of 40 or higher is considered to be morbidly obese, and this is usually the minimum BMI requirement for weight loss surgery.  * **Your health conditions**. Weight loss surgery can help to improve a number of health conditions that are associated with obesity, such as type 2 diabetes, high blood pressure, and sleep apnea. Surgeons will want to make sure that your health conditions are stable enough to withstand surgery.  * **Your motivation**. Weight loss surgery is a major life change, and it is important that you are motivated to make lifestyle changes that will support your weight loss. Surgeons will want to make sure that you are committed to following through with the necessary dietary and exercise changes after surgery.  If you are considering weight loss surgery, it is important to talk to your doctor about your individual circumstances. They can help you to determine whether weight loss surgery is right for you and what the risks and benefits may be.  In simpler terms, weight loss surgery is not a quick fix for obesity. It is a major surgery with risks and benefits, and it is important to talk to your doctor to see if it is right for you. |
| 5 | Is there a weight limit for this operation? | We look at Body Mass Index (BMI), which is the weight over the body surface area squared. Patients who have a BMI of 35-40 and have obesity related co-morbidities are candidates for this surgery. Patients who have a BMI greater than 40, even if they don't have co-morbidities, are also candidates for this operation. | Is there a weight limit for weight loss surgery? | There is no specific weight limit for weight loss surgery. However, most surgeons will require that you have a body mass index (BMI) of 35 or higher, or a BMI between 30 and 35 and an obesity-related condition, such as heart disease, diabetes, high blood pressure or severe sleep apnea. You will also need to be able to fit on the operating table and other equipment used in the surgery.  The maximum weight that a patient can be for weight loss surgery varies depending on the surgeon and the type of surgery. Some surgeons may have a weight limit of 400 pounds, while others may allow patients up to 500 pounds or more. If you are above the weight limit for a particular surgeon, they may still be able to operate on you, but they may need to take additional precautions.  It is important to talk to your doctor about your weight loss surgery options and to find a surgeon who is experienced in operating on patients of all sizes.  Here are some of the factors that surgeons consider when determining whether a patient is a good candidate for weight loss surgery:  * **BMI:** A BMI of 35 or higher is typically required, but some surgeons may consider patients with a BMI of 30 or higher if they have obesity-related health conditions.  * **Other health conditions:** Patients with obesity-related health conditions, such as heart disease, diabetes, high blood pressure or severe sleep apnea, are more likely to benefit from weight loss surgery.  * **Age:** Most surgeons will not operate on patients who are over the age of 70, but some may consider patients who are younger than 18.  * **Ability to follow through with the necessary lifestyle changes:** Weight loss surgery is not a magic bullet. Patients who have surgery still need to make changes to their diet and exercise habits in order to lose weight and keep it off.  If you are considering weight loss surgery, it is important to talk to your doctor about your individual circumstances and to find a surgeon who is experienced in operating on patients of all sizes. | 7/8/2023 | Sure. Weight loss surgery is a big decision, and it's important to understand the factors that surgeons consider when making a recommendation.  In general, you'll need to have a BMI of 35 or higher to qualify for weight loss surgery. If your BMI is lower than 35, but you have obesity-related health conditions, such as heart disease, diabetes, high blood pressure or severe sleep apnea, you may still be a candidate for surgery.  Your age can also be a factor. Most surgeons will not operate on patients who are over the age of 70, but some may consider patients who are younger than 18.  Finally, surgeons will want to make sure that you are able to follow through with the necessary lifestyle changes after surgery. This includes making changes to your diet and exercise habits in order to lose weight and keep it off.  If you are considering weight loss surgery, it's important to talk to your doctor about your individual circumstances and to find a surgeon who is experienced in operating on patients of all sizes.  Here is a table that summarizes the factors that surgeons consider when determining whether a patient is a good candidate for weight loss surgery:  \| Factor \| Description \|  \|---\|---\|  \| BMI \| Body mass index. A BMI of 35 or higher is typically required, but some surgeons may consider patients with a BMI of 30 or higher if they have obesity-related health conditions. \|  \| Other health conditions \| Patients with obesity-related health conditions, such as heart disease, diabetes, high blood pressure or severe sleep apnea, are more likely to benefit from weight loss surgery. \|  \| Age \| Most surgeons will not operate on patients who are over the age of 70, but some may consider patients who are younger than 18. \|  \| Ability to follow through with the necessary lifestyle changes \| Weight loss surgery is not a magic bullet. Patients who have surgery still need to make changes to their diet and exercise habits in order to lose weight and keep it off. \|  I hope this helps! |
| 5 | What are obesity related comorbidities? | These include diabetes, hypertension, arthritis, degenerative joint disease, sleep apnea, low pack pain, gastroesophageal reflux disease, stress incontinence, high cholesterol and triglyceride levels and depression. |  | Obesity is a complex disease that can lead to a number of other health problems, known as obesity-related comorbidities. These conditions can increase the risk of premature death and disability.  Some of the most common obesity-related comorbidities include:  * **Cardiovascular disease:** Obesity is a major risk factor for heart disease, stroke, and heart failure.  * **Type 2 diabetes:** Obesity is the leading cause of type 2 diabetes, a chronic condition that affects the way your body turns food into energy.  * **High blood pressure:** Obesity can increase your risk of high blood pressure, a condition that can damage your heart, blood vessels, and kidneys.  * **Sleep apnea:** Obesity is a major risk factor for sleep apnea, a sleep disorder that can cause you to stop breathing repeatedly during the night.  * **Osteoarthritis:** Obesity can increase your risk of osteoarthritis, a joint disease that causes pain and stiffness in the joints.  * **Some types of cancer:** Obesity is linked to an increased risk of some types of cancer, including endometrial cancer, breast cancer, and colon cancer.  * **Mental health conditions:** Obesity is associated with an increased risk of mental health conditions, such as depression, anxiety, and eating disorders.  If you are overweight or obese, it is important to talk to your doctor about your risk of developing these conditions. There are a number of things you can do to reduce your risk, including losing weight, eating a healthy diet, and exercising regularly.  In addition to the above, obesity has also been linked to an increased risk of:  * **Gallstones:** Obesity can increase your risk of developing gallstones, which are small, hard deposits that form in the gallbladder.  * **Varicose veins:** Obesity can increase your risk of developing varicose veins, which are enlarged veins that can appear on the legs.  * **PCOS:** Obesity is a major risk factor for polycystic ovary syndrome (PCOS), a hormonal disorder that can cause irregular periods, infertility, and other health problems.  * **Wound healing problems:** Obesity can slow down the healing process of wounds, making it more difficult to recover from surgery or injury.  * **Thromboembolism:** Obesity is a risk factor for venous thromboembolism (VTE), a condition that occurs when a blood clot forms in a vein, often in the leg. VTE can be life-threatening.  If you are concerned about your weight or your risk of developing obesity-related comorbidities, talk to your doctor. They can help you assess your risk and develop a plan to lose weight and improve your health. | 7/8/2023 | Sure. Obesity is a condition where a person has too much body fat. This can lead to a number of health problems, including heart disease, stroke, type 2 diabetes, high blood pressure, sleep apnea, osteoarthritis, some types of cancer, and mental health conditions.  In simple terms, obesity can make you sick. It can damage your heart, blood vessels, kidneys, joints, and even your mental health.  If you are overweight or obese, it is important to talk to your doctor about your risk of developing these conditions. There are a number of things you can do to reduce your risk, including losing weight, eating a healthy diet, and exercising regularly.  Here are some tips for losing weight:  * Set realistic goals. Aim to lose 1-2 pounds per week.  * Make small changes to your diet and lifestyle. These changes are more likely to be sustainable in the long run.  * Find a support system. Having friends or family members who are also trying to lose weight can help you stay motivated.  If you are struggling to lose weight on your own, talk to your doctor. They can help you develop a personalized weight loss plan.  It is important to remember that losing weight takes time and effort. But it is worth it to improve your health and reduce your risk of developing obesity-related comorbidities. |
| 5 | What are the pre-operative requirements in order to undergo either operation? | You must have a stable relationship with a primary care physician who has been treating you continuously for one year prior to consideration for surgery.  You must have a dietician evaluation.  You must have a psychological evaluation. Patients with bipolar disease are generally excluded from consideration for surgery.  If you have a history of heart disease, if you've taken Fen-Phen in the past, if you're over the age of 50, or other cardiac risk factors, you'll require an echocardiogram.  At some point prior to surgery, you'll have to undergo a chest x-ray, an EKG and blood tests. | What are the pre-operative requirements in order to undergo weight loss surgery? | The pre-operative requirements for weight loss surgery vary depending on the type of surgery and the individual patient's circumstances. However, some common requirements include:  * **A thorough medical evaluation:** This will include a physical exam, blood tests, and other tests to assess your overall health and any potential risks associated with surgery.  * **A psychological evaluation:** This will assess your mental health and readiness for surgery.  * **A nutritional assessment:** This will help you develop a plan to eat a healthy diet before and after surgery.  * **A weight loss program:** You may be required to lose weight before surgery. This will help to reduce the risks of surgery and make it more successful.  * **Stopping smoking and drinking alcohol:** Smoking and drinking alcohol can increase the risks of surgery. You will need to stop smoking and drinking alcohol for at least 2 weeks before surgery.  * **Getting all necessary vaccinations:** You will need to get all necessary vaccinations, such as the flu shot and pneumonia shot.  In addition to these general requirements, there may be other specific requirements that you need to meet in order to qualify for weight loss surgery. Your doctor will be able to tell you what specific requirements you need to meet.  Once you have met all of the pre-operative requirements, you will be scheduled for surgery. Weight loss surgery can be a life-changing procedure, but it is important to remember that it is not a magic bullet. You will still need to make lifestyle changes, such as eating a healthy diet and exercising regularly, in order to maintain your weight loss after surgery.  Here are some additional resources that you may find helpful:  * The National Institute of Diabetes and Digestive and Kidney Diseases: https://www.niddk.nih.gov/health-information/weight-management/bariatric-surgery  * The American Society for Metabolic and Bariatric Surgery: https://asmbs.org/  * The Obesity Action Coalition: https://obesityaction.org/ | 7/8/2023 | Sure. Here are the pre-operative requirements for weight loss surgery in simpler terms:  * You need to be healthy enough to have surgery. This means that you need to have a physical exam and blood tests to make sure that you are not at risk for any complications.  * You need to be mentally prepared for surgery. This means that you need to understand the risks and benefits of surgery, and you need to be committed to making lifestyle changes after surgery.  * You need to lose some weight before surgery. This will help to reduce the risks of surgery and make it more successful.  * You need to stop smoking and drinking alcohol. Smoking and drinking alcohol can increase the risks of surgery.  * You need to get all necessary vaccinations. This will help to protect you from infections after surgery.  If you meet all of these requirements, you will be able to have weight loss surgery. However, it is important to remember that weight loss surgery is not a magic bullet. You will still need to make lifestyle changes after surgery in order to maintain your weight loss.  Here are some additional tips for preparing for weight loss surgery:  * Start seeing a therapist or counselor to talk about your weight loss goals and to help you develop coping mechanisms for dealing with stress.  * Join a support group for people who are considering or have had weight loss surgery. This can be a great way to connect with others who understand what you are going through and to get support from people who have been through the process before.  * Read books and articles about weight loss surgery. This will help you to learn more about the different types of surgery and the risks and benefits of each.  * Talk to your doctor about your expectations for weight loss surgery. This will help you to set realistic goals and to understand what you can expect from surgery.  I hope this helps! |
| 5 | What does laparoscopic surgery mean? | Laparoscopic surgery means that instead of making one large incision in your abdomen, we make five small incisions in the middle and to the left side of your abdomen. These smaller incisions allow you to recover faster. You'll also experience less pain, leave the hospital sooner and will be back to your normal activity sooner. In general, we start all of our cases laparoscopically unless the patient has had previous surgery on their stomach. |  | Laparoscopic surgery is a minimally invasive surgical procedure that uses a laparoscope, a thin, tube-like instrument with a camera and light on the end, to see inside the abdomen or pelvis. The laparoscope is inserted through a small incision in the abdomen, and the surgeon uses it to view the organs and tissues inside. Other instruments can also be inserted through the incisions to perform surgery.  Laparoscopic surgery has several advantages over traditional open surgery, including:  * Smaller incisions, which lead to less pain, scarring, and recovery time  * Reduced risk of infection  * Faster return to normal activities  Laparoscopy can be used to diagnose a wide range of conditions, including:  * Endometriosis  * Ovarian cysts  * Adhesions  * Inflammatory bowel disease  * Gallbladder disease  * Hernias  It can also be used to treat a variety of conditions, including:  * Appendicitis  * Ectopic pregnancy  * Infertility  * Cancer  * Gallbladder removal  * Hernia repair  If you are considering laparoscopic surgery, talk to your doctor about the risks and benefits. | 7/8/2023 | Sure. Laparoscopic surgery is a type of surgery where the surgeon makes a few small incisions in your abdomen instead of one big one. The surgeon then inserts a thin tube with a camera and light on the end (called a laparoscope) through one of the incisions. The laparoscope allows the surgeon to see inside your abdomen on a TV monitor. Other surgical instruments can also be inserted through the incisions to perform the surgery.  Laparoscopic surgery is often used instead of traditional open surgery because it has several advantages, including:  * Smaller incisions, which mean less pain, scarring, and recovery time  * Reduced risk of infection  * Faster return to normal activities  Laparoscopy can be used to diagnose and treat a wide range of conditions, including:  * Endometriosis  * Ovarian cysts  * Adhesions  * Inflammatory bowel disease  * Gallbladder disease  * Hernias  * Appendicitis  * Ectopic pregnancy  * Infertility  * Cancer  * Gallbladder removal  * Hernia repair  If you are considering laparoscopic surgery, talk to your doctor about the risks and benefits. |
| ASMBS | When can I start exercising after surgery? | Right away! To start, you will take easy, short walks while you are in the hospital. The key is to start slow. Listen to your body and your surgeon. If you lift weights or do sports, stay “low impact” for the first month. | When can I start exercising after weight loss surgery? | The timing of when you can start exercising after weight loss surgery will vary depending on the type of surgery you had, your overall health, and how you are feeling. However, most people can start exercising 2-6 weeks after surgery.  Here are some general guidelines for when you can start exercising after weight loss surgery:  * **2 weeks after surgery:** You can start walking short distances, such as around the house or block.  * **4 weeks after surgery:** You can start increasing the intensity and duration of your walks. You can also start swimming or biking.  * **6 weeks after surgery:** You can start doing more vigorous exercises, such as running or weight lifting.  It is important to listen to your body and start slowly. If you experience any pain, stop the exercise and rest. You should also avoid activities that involve heavy lifting or twisting for at least 6 weeks after surgery.  Here are some tips for exercising safely after weight loss surgery:  * Start slowly and gradually increase the intensity and duration of your workouts.  * Listen to your body and stop if you experience any pain.  * Stay hydrated by drinking plenty of water before, during, and after your workouts.  * Wear comfortable clothing and shoes that support your feet.  * Choose exercises that you enjoy and that are appropriate for your fitness level.  Exercising after weight loss surgery is an important part of your recovery and long-term weight loss success. By following these guidelines, you can safely and effectively get started on your new exercise routine.  Here are some of the best exercises to do after weight loss surgery:  * **Walking:** Walking is a low-impact exercise that is easy on your joints. It is a great way to start exercising after surgery and to gradually increase your activity level.  * **Swimming:** Swimming is another low-impact exercise that is great for people who have had weight loss surgery. It is a great way to cool down after a workout and to improve your flexibility.  * **Cycling:** Cycling is a great way to get some cardio exercise without putting too much stress on your joints. It is a good option for people who are looking for a more challenging workout.  * **Yoga:** Yoga is a great way to improve your flexibility and strength. It is also a good way to reduce stress and anxiety.  * **Pilates:** Pilates is a great way to strengthen your core muscles. It is a good option for people who are looking for a low-impact workout that can help them improve their posture.  If you are not sure what type of exercise is right for you, talk to your doctor or a certified personal trainer. They can help you create an exercise plan that is safe and effective for your individual needs. | 7/8/2023 | Sure. After weight loss surgery, you can start exercising 2-6 weeks after surgery. You should start slowly and gradually increase the intensity and duration of your workouts. Listen to your body and stop if you experience any pain. Stay hydrated by drinking plenty of water before, during, and after your workouts. Wear comfortable clothing and shoes that support your feet. Choose exercises that you enjoy and that are appropriate for your fitness level.  Some of the best exercises to do after weight loss surgery are walking, swimming, cycling, yoga, and Pilates. These exercises are low-impact and easy on your joints. They are also a great way to improve your flexibility, strength, and overall fitness.  If you are not sure what type of exercise is right for you, talk to your doctor or a certified personal trainer. They can help you create an exercise plan that is safe and effective for your individual needs.  Here is a table that summarizes the guidelines for when you can start exercising after weight loss surgery:  \| Time After Surgery \| Exercises \|  \|---\|---\|  \| 2 weeks \| Walking short distances \|  \| 4 weeks \| Increasing the intensity and duration of walks, swimming, biking \|  \| 6 weeks \| Running, weight lifting \|  It is important to remember that everyone is different, so you may need to start exercising sooner or later than these guidelines. Listen to your body and start slowly. If you experience any pain, stop the exercise and rest. |
| ASMBS | Will I have to exercise after the procedure? | For many patients, exercise is important for stress control and appetite control, as well as burning off calories.  As we age, lack of activity can lead to being frail or fragile, which is quite dangerous to overall health. Healthy bones and avoiding muscle loss partly depends on doing weekly exercise.  Most patients also think of exercise as something that must be intense and painful (like “boot camp”). Regular, modest activity is far more useful in the long term. Even elite athletes can’t stay at a “peak” every week of the year. Instead, work with your surgeon’s program to find a variety of activities that can work for you. There is no “one-size-fits-all” plan. Expect to learn and change as you go! | Will I have to exercise after weight loss surgery? | Yes, you will need to exercise after weight loss surgery. Exercise is an important part of the weight loss journey, and it can help you to lose weight, maintain your weight loss, and improve your overall health.  The type and amount of exercise you can do after weight loss surgery will vary depending on your individual needs and the type of surgery you have. However, most people can start with light exercise, such as walking, and gradually increase the intensity and duration of their workouts as they heal.  Here are some of the benefits of exercise after weight loss surgery:  * Helps you to lose weight and maintain your weight loss.  * Improves your overall health.  * Reduces your risk of developing chronic diseases, such as heart disease, stroke, type 2 diabetes, and some types of cancer.  * Improves your mood and energy levels.  * Strengthens your muscles and bones.  * Improves your balance and coordination.  If you are considering weight loss surgery, talk to your doctor about the importance of exercise and how you can safely start exercising after your surgery.  Here are some tips for exercising after weight loss surgery:  * Start slowly and gradually increase the intensity and duration of your workouts.  * Listen to your body and stop if you feel pain.  * Stay hydrated.  * Wear comfortable clothing and shoes.  * Choose activities that you enjoy.  * Find a workout buddy to help you stay motivated.  With regular exercise, you can improve your overall health and well-being after weight loss surgery. | 7/8/2023 | Sure. Exercise is important after weight loss surgery because it can help you lose weight, keep the weight off, and improve your overall health. You can start with light exercise, such as walking, and gradually increase the intensity and duration of your workouts as you heal.  Here are some simple tips for exercising after weight loss surgery:  * Start slowly. Don't try to do too much too soon.  * Listen to your body. If you feel pain, stop and rest.  * Stay hydrated. Drink plenty of water before, during, and after your workout.  * Wear comfortable clothing and shoes.  * Choose activities that you enjoy.  * Find a workout buddy to help you stay motivated.  With regular exercise, you can improve your overall health and well-being after weight loss surgery.  Here are some examples of light exercise that you can do after weight loss surgery:  * Walking  * Swimming  * Biking  * Yoga  * Pilates  * Tai chi  As you heal, you can gradually increase the intensity and duration of your workouts. You may also want to add strength training exercises to your routine.  It is important to talk to your doctor before starting any new exercise program after weight loss surgery. They can help you create a safe and effective exercise plan that is right for you. |
| ASMBS | Will I have to go on a diet before I have surgery? | Yes. Many insurance companies require patients to be on a weight loss program before qualifying for surgery.  Many bariatric surgeons put their patients on a special pre-operative diet, usually 2 or 3 weeks just before surgery. The reason for the pre-operative diet is to shrink the liver and reduce fat in the abdomen. This helps during the procedure and makes it safer.  Some insurance companies require a physician-monitored diet three to six months prior to surgery as part of their coverage requirement. These diets are very different from the short-term diets and usually are more about food education and showing a willingness to complete appointments and to learn. | Will I have to go on a diet before I have weight loss surgery? | Yes, you will need to go on a diet before weight loss surgery. The specific diet you will need to follow will depend on the type of surgery you are having, but most surgeons will require you to follow a low-calorie, high-protein diet for two weeks before surgery. This will help to shrink your liver and make the surgery safer. You will also need to stop smoking and drinking alcohol before surgery.  Here are some of the general guidelines for the pre-surgery diet:  * Eat 6-8 small meals per day.  * Choose lean protein sources, such as chicken, fish, and tofu.  * Eat plenty of non-starchy vegetables.  * Limit your intake of carbohydrates and sugars.  * Drink plenty of water and other calorie-free beverages.  Your doctor or a registered dietitian can help you create a specific diet plan that is right for you.  Here are some of the benefits of following a pre-surgery diet:  * It can help to shrink your liver, making the surgery safer.  * It can help you lose weight, which can make the surgery more effective.  * It can help you learn healthy eating habits that you can continue after surgery.  If you are considering weight loss surgery, it is important to talk to your doctor about the pre-surgery diet requirements. They can help you create a plan that is right for you and make sure that you are prepared for surgery. | 7/8/2023 | Sure. Before you have weight loss surgery, you will need to follow a special diet to help you lose weight and prepare your body for surgery. This diet is usually low in calories and high in protein. It is important to follow this diet carefully to make sure that you are healthy enough for surgery.  Here are some of the things you will need to do:  * Eat 6-8 small meals per day. This will help you feel full and prevent you from getting too hungry.  * Choose lean protein sources, such as chicken, fish, and tofu. Protein is important for healing after surgery.  * Eat plenty of non-starchy vegetables. These vegetables are low in calories and high in nutrients.  * Limit your intake of carbohydrates and sugars. These foods can cause weight gain and make it harder to lose weight after surgery.  * Drink plenty of water and other calorie-free beverages. This will help you stay hydrated and prevent constipation.  If you are following this diet carefully, you should start to lose weight within a few days. You may also notice that you have more energy and feel better overall.  It is important to talk to your doctor about the pre-surgery diet requirements. They can help you create a plan that is right for you and make sure that you are prepared for surgery.  Here are some additional tips for following the pre-surgery diet:  * Plan your meals ahead of time. This will help you make sure that you have the right foods on hand and that you are not tempted to eat unhealthy foods.  * Read food labels carefully. This will help you make sure that you are choosing foods that are low in calories and high in nutrients.  * Cook at home more often. This will help you control the ingredients in your food and make sure that you are eating healthy meals.  * Find a support system. Having people to support you can help you stay on track with your diet and make the pre-surgery process easier.  Following the pre-surgery diet is an important part of preparing for weight loss surgery. By following these tips, you can make sure that you are healthy and ready for surgery. |
| ASMBS | Will I have to be on a diet after the surgery? | No. Most people think of a “diet” as a plan that leaves you hungry. That is not the way people feel after surgery.  Eventually, most patients get some form of appetite back 6-18 months after surgery. Your appetite is much weaker, and easier to satisfy than before.  This does not mean you can whatever and whenever you want. Healthier food choices are important for best results, but most patients still enjoy tasty food, and even occasional “treats”. | Will I have to be on a diet after weight loss surgery? | Yes, you will have to be on a diet after weight loss surgery. The specific diet will vary depending on the type of surgery you have, but all bariatric surgery patients will need to follow a strict diet in the weeks and months following surgery. This diet is designed to help you lose weight safely and effectively, and to prevent you from regaining the weight you lost.  The general principles of the post-surgery diet include:  * Eating small, frequent meals throughout the day.  * Choosing high-protein, low-fat foods.  * Avoiding sugary drinks and foods.  * Chewing your food thoroughly.  * Drinking plenty of fluids.  You will also need to follow a set of specific guidelines for what foods you can and cannot eat. These guidelines will be tailored to your individual needs, but some common foods that are restricted after bariatric surgery include:  * Red meat that's tough or dry.  * Greasy, high-fat foods.  * Heavily seasoned or spicy foods.  * Sugar alcohols, such as erythritol, glycerol, mannitol, sorbitol and xylitol.  * Foods reheated in the microwave.  Following the post-surgery diet is essential for your long-term success. By eating a healthy diet and making lifestyle changes, you can achieve and maintain a healthy weight after surgery.  Here are some additional tips for following the post-surgery diet:  * Plan your meals ahead of time.  * Cook at home more often.  * Read food labels carefully.  * Make sure you have plenty of healthy snacks on hand.  * Don't be afraid to ask for help from your doctor or a registered dietitian.  Following the post-surgery diet can be challenging, but it is worth it for your health. By making healthy choices, you can achieve your weight loss goals and improve your overall health. | 7/8/2023 | Sure. After weight loss surgery, you will need to follow a special diet to help you lose weight and keep it off. The diet will be different for everyone, but it will generally include:  * Eating small, frequent meals throughout the day.  * Choosing healthy foods like fruits, vegetables, and lean protein.  * Avoiding unhealthy foods like sugary drinks, processed foods, and fatty foods.  * Chewing your food thoroughly.  * Drinking plenty of fluids.  You will also need to avoid some foods altogether, such as:  * Red meat that's tough or dry.  * Greasy, high-fat foods.  * Heavily seasoned or spicy foods.  * Sugar alcohols.  * Foods reheated in the microwave.  Following the diet after weight loss surgery can be challenging, but it is important for your health. By making healthy choices, you can achieve your weight loss goals and improve your overall health.  Here are some simple tips for following the diet:  * Plan your meals ahead of time.  * Cook at home more often.  * Read food labels carefully.  * Make sure you have plenty of healthy snacks on hand.  * Don't be afraid to ask for help from your doctor or a registered dietitian.  I hope this helps! |
| ASMBS | Is weight loss surgery a ‘cop-out’? To lose weight, should people with obesity just go on a diet and exercise? | Many people who suffer from obesity find it hard to lose weight and keep the weight off with diet and exercise alone. This is likely due to foods available and genes.  The National Institutes of Health (NIH) Expert Panel stated that, without surgery, long-term weight loss is nearly impossible for those affected by severe obesity. Studies show little long-term success with diet and exercise alone.(3) Weight loss surgeries are effective in maintaining long-term weight loss, in part, because these procedures change the body’s natural responses to dieting that make weight loss so difficult. When a person goes on a diet, their body produces more hormones that cause an increase in hunger and a decrease in calories burned. This decrease in calories the body burns is more than explained by the decrease in body size. Therefore, there are significant differences between someone who has lost weight by diet and someone of the same size who has never lost weight. For example, the body of the person who reduces their weight from 200 to 170 pounds burns fewer calories than the body of someone weighing 170 pounds who has never been on a diet. This means that in order to maintain weight loss, the person who has been on a diet will have to eat fewer calories than someone who naturally weighs the same. Weight loss procedures, unlike diet, also cause biological changes that help reduce food intake. Energy (in the form of food) intake is decreased with surgery by restricting stomach size and limiting absorption. In addition, weight loss surgery changes the production of certain gut hormones (or signals) that communicate with the brain to reduce hunger, decrease appetite, and enhance the feeling of being full. In these ways, weight loss surgery, unlike dieting, produces long-term weight loss. |  | Weight loss surgery is not a "cop-out." It is a serious medical procedure that should only be considered after other weight loss methods have failed. People with obesity who are considering weight loss surgery should be prepared to make significant lifestyle changes, including following a healthy diet and exercising regularly.  There are many reasons why weight loss surgery may be a good option for people with obesity. For some people, diet and exercise are simply not enough to lose weight and keep it off. Weight loss surgery can help people lose weight more quickly and easily, and it can also help them to keep the weight off in the long term.  In addition, weight loss surgery can improve a person's overall health. Obesity is a major risk factor for many chronic diseases, including heart disease, stroke, type 2 diabetes, and some types of cancer. Weight loss surgery can help to reduce the risk of these diseases, and it can also improve a person's quality of life.  However, it is important to remember that weight loss surgery is not a magic bullet. It is a tool that can help people lose weight, but it is not a substitute for healthy lifestyle habits. People who have weight loss surgery still need to follow a healthy diet and exercise regularly in order to maintain their weight loss.  The decision of whether or not to have weight loss surgery is a personal one. There are many factors to consider, including the severity of a person's obesity, their overall health, and their willingness to make lifestyle changes. If you are considering weight loss surgery, it is important to talk to your doctor about the risks and benefits of the procedure.  Here are some of the benefits of weight loss surgery:  * It can help people lose a significant amount of weight quickly.  * It can help people keep the weight off in the long term.  * It can improve a person's overall health.  * It can reduce the risk of chronic diseases.  * It can improve a person's quality of life.  Here are some of the risks of weight loss surgery:  * It is a major surgery, and there are risks associated with any surgery.  * It can cause complications, such as infections, bleeding, and nutritional deficiencies.  * It is not a cure for obesity, and people who have weight loss surgery still need to follow a healthy diet and exercise regularly.  Ultimately, the decision of whether or not to have weight loss surgery is a personal one. There are many factors to consider, and it is important to talk to your doctor about the risks and benefits of the procedure. | 7/8/2023 | Sure. Weight loss surgery is a serious medical procedure that can help people with obesity lose weight and improve their health. But it's not a magic bullet. People who have weight loss surgery still need to follow a healthy diet and exercise regularly.  Here's a simplified version of the pros and cons of weight loss surgery:  **Pros:**  * Can help people lose a significant amount of weight quickly.  * Can help people keep the weight off in the long term.  * Can improve a person's overall health.  * Can reduce the risk of chronic diseases.  * Can improve a person's quality of life.  **Cons:**  * It is a major surgery, and there are risks associated with any surgery.  * It can cause complications, such as infections, bleeding, and nutritional deficiencies.  * It is not a cure for obesity, and people who have weight loss surgery still need to follow a healthy diet and exercise regularly.  Ultimately, the decision of whether or not to have weight loss surgery is a personal one. There are many factors to consider, and it's important to talk to your doctor about the risks and benefits of the procedure.  Here are some additional things to keep in mind about weight loss surgery:  * It's not a quick fix. It takes time and effort to lose weight and keep it off, even with weight loss surgery.  * It's not a guarantee that you'll lose all the weight you want.  * There are risks associated with any surgery, including weight loss surgery.  * You'll need to make lifestyle changes, such as following a healthy diet and exercising regularly, in order to maintain your weight loss.  If you're considering weight loss surgery, it's important to talk to your doctor about your individual circumstances. They can help you decide if weight loss surgery is right for you. |
| ASMBS | When can I get pregnant after weight loss surgery? | Most groups advise waiting 12-18 months after surgery before getting pregnant.  Most women are much more fertile after surgery, even with moderate weight loss.  Birth control pills do NOT work as well in patients who are overweight. Birth control pills are not very reliable during the time your weight is changing. For this reason, having an IUD or using condoms and spermicide with ALL intercourse is needed. Menstrual periods can be very irregular, and you can get pregnant when you least expect it! |  | The American College of Obstetricians and Gynecologists (ACOG) recommends waiting at least 12 months after weight loss surgery before getting pregnant. This is to allow your body time to adjust to the changes in your metabolism and hormones, and to give you time to reach a stable weight.  Some experts recommend waiting even longer, up to 18-24 months, before getting pregnant. This is because pregnancy can put extra stress on your body, and it's important to be at a healthy weight before you start trying to conceive.  If you do get pregnant before 12 months, it's important to talk to your doctor right away. They can help you monitor your health and make sure that you and your baby are getting the nutrients you need.  Here are some of the reasons why it's important to wait to get pregnant after weight loss surgery:  * You may not have lost enough weight yet. Losing weight before pregnancy can help reduce your risk of complications, such as gestational diabetes, high blood pressure, and premature birth.  * Your body may not be fully recovered from surgery. Weight loss surgery can have a significant impact on your metabolism and hormones, and it takes time for your body to adjust.  * You may not be getting enough nutrients. Weight loss surgery can affect your ability to absorb nutrients, so it's important to make sure that you're getting enough of the nutrients you need before you get pregnant.  If you're considering pregnancy after weight loss surgery, talk to your doctor. They can help you determine when it's safe for you to get pregnant and make sure that you're getting the care you need to have a healthy pregnancy. | 7/8/2023 | Sure. Here's a simpler explanation:  * After weight loss surgery, your body goes through a lot of changes. It takes time for your body to adjust to these changes and for you to reach a stable weight.  * Pregnancy can be hard on your body, even if you're not overweight. If you're not at a healthy weight before you get pregnant, you're more likely to have complications, such as gestational diabetes, high blood pressure, and premature birth.  * To reduce your risk of complications and have a healthy pregnancy, it's best to wait at least 12 months after weight loss surgery before getting pregnant. Some experts recommend waiting even longer, up to 18-24 months.  If you're considering pregnancy after weight loss surgery, talk to your doctor. They can help you determine when it's safe for you to get pregnant and make sure that you're getting the care you need to have a healthy pregnancy.  Here are some of the things your doctor will talk to you about:  * Your weight loss progress. How much weight have you lost since surgery? Are you still losing weight?  * Your overall health. Are you taking any medications? Do you have any other health conditions?  * Your nutritional status. Are you getting enough of the nutrients you need?  * Your risk of complications. Are you at increased risk for any complications, such as gestational diabetes or high blood pressure?  Once you and your doctor have talked about all of these factors, they can help you make a decision about when it's safe for you to get pregnant. |
| ASMBS | Will the baby be healthy? | Yes.  After surgery, there is much less risk of experiencing problems during pregnancy and during childbirth. There are also fewer miscarriages and stillbirths than in women with obesity who have not had surgery and weight loss.  Kids born after mom’s surgery are LESS at risk of being affected by obesity later, due to activation of certain genes during fetal growth. There is also less risk of needing a C section. | Will the baby be healthy (posted in the same chat as the previous question) | The chances of your baby being healthy are good if you wait at least 12 months after weight loss surgery before getting pregnant and if you follow your doctor's instructions during pregnancy. However, there are some potential risks to your baby's health that you should be aware of.  * **Small for gestational age (SGA):** Babies born to mothers who have had weight loss surgery are more likely to be SGA. This means that they may be born smaller than expected for their gestational age. SGA babies are at increased risk of health problems, such as hypoglycemia (low blood sugar), respiratory problems, and jaundice.  * **Nutritional deficiencies:** Weight loss surgery can affect your ability to absorb nutrients, so it's important to make sure that you're getting enough of the nutrients you need during pregnancy. If you're not getting enough nutrients, your baby may be at risk for nutritional deficiencies.  * **Premature birth:** Mothers who have had weight loss surgery are more likely to deliver their babies prematurely. Premature babies are at increased risk of health problems, such as respiratory problems, infections, and developmental delays.  If you're considering pregnancy after weight loss surgery, talk to your doctor. They can help you determine when it's safe for you to get pregnant and make sure that you're getting the care you need to have a healthy pregnancy.  Here are some things you can do to help ensure your baby's health:  * Wait at least 12 months after weight loss surgery before getting pregnant.  * Follow your doctor's instructions during pregnancy.  * Take prenatal vitamins.  * Eat a healthy diet.  * Get regular prenatal care.  By following these steps, you can help ensure that your baby is born healthy and happy. | 7/8/2023 | Sure. Here are the risks to your baby's health in simpler terms:  * **Small for gestational age (SGA):** This means that your baby may be born smaller than expected for their age. This can happen because weight loss surgery can affect your ability to absorb nutrients.  * **Nutritional deficiencies:** This means that your baby may not get enough of the nutrients they need to grow and develop properly. This can happen because weight loss surgery can affect your ability to absorb nutrients.  * **Premature birth:** This means that your baby may be born before they are fully developed. This can happen because weight loss surgery can affect your body's ability to carry a pregnancy to term.  Here are some things you can do to help ensure your baby's health:  * **Wait at least 12 months after weight loss surgery before getting pregnant.** This will give your body time to adjust to the surgery and to lose enough weight.  * **Follow your doctor's instructions during pregnancy.** This includes taking prenatal vitamins, eating a healthy diet, and getting regular prenatal care.  * **Talk to your doctor about your concerns.** They can help you understand the risks and make sure that you are getting the care you need to have a healthy pregnancy.  I hope this helps! |
| ASMBS | How long after weight loss surgery will I have to be from work? | After surgery, most patients return to work in one or two weeks. |  | The amount of time you need to be off work after weight loss surgery depends on the type of surgery you have and your individual recovery. In general, most patients return to work in 1-4 weeks, depending upon procedure.  * **Gastric sleeve:** 2-3 weeks  * **Gastric bypass:** 2-3 weeks  * **Lap band:** 1-2 weeks  However, some patients may need to take longer off work, especially if they have a physically demanding job. It is important to talk to your doctor about your individual recovery needs before you return to work.  Here are some things to keep in mind when deciding when to return to work after weight loss surgery:  * Your energy levels. You may not have the same energy levels as you did before surgery, so it is important to listen to your body and take breaks when you need them.  * Your pain level. You should be able to tolerate most activities without pain before you return to work.  * Your ability to lift and carry objects. If your job requires you to lift or carry heavy objects, you will need to wait until you are cleared by your doctor to do so.  * Your ability to sit for long periods of time. If your job requires you to sit for long periods of time, you may need to take breaks to walk around or stand up.  It is also important to remember that you will need to make some changes to your diet and lifestyle after weight loss surgery. This may include eating smaller meals more often, drinking plenty of fluids, and avoiding certain foods. You will also need to be more physically active.  If you are considering weight loss surgery, it is important to talk to your doctor about your individual needs and recovery plan. They can help you determine when it is safe for you to return to work. | 7/8/2023 | Sure. The amount of time you need to be off work after weight loss surgery depends on the type of surgery you have and how you recover. Most people can return to work in 1-4 weeks, but some people may need to take longer.  * If you have a gastric sleeve or gastric bypass, you will likely need to be off work for 2-3 weeks.  * If you have a lap band, you may be able to return to work in 1-2 weeks.  However, if your job is physically demanding or requires you to lift heavy objects, you may need to take longer off work. It is important to talk to your doctor about your individual recovery needs before you return to work.  Here are some general rules of thumb:  * If you are feeling tired or have pain, you should not return to work yet.  * If you are not able to lift or carry heavy objects, you should not return to work yet.  * If you are not able to sit for long periods of time, you may need to take breaks to walk around or stand up.  It is also important to remember that you will need to make some changes to your diet and lifestyle after weight loss surgery. This may include eating smaller meals more often, drinking plenty of fluids, and avoiding certain foods. You will also need to be more physically active.  If you are considering weight loss surgery, it is important to talk to your doctor about your individual needs and recovery plan. They can help you determine when it is safe for you to return to work.  Here are some additional things to keep in mind:  * Your doctor may recommend that you start with part-time work or work from home for a few weeks after surgery.  * You may need to modify your work duties or schedule to accommodate your recovery.  * It is important to listen to your body and take breaks when you need them.  * You should not return to work if you are feeling pain or discomfort.  I hope this helps! |
| ASMBS | Will I lose my hair after surgery? | Some hair loss is common between 3 and 6 months following surgery but almost always temporary.  The reasons for hair loss are not totally understood. Even if you take all recommended supplements and meet protein requirements, hair loss will be noticed until the follicles come back. Adequate intake of protein, vitamins, and minerals will help to ensure hair re-growth, and avoid longer term thinning. | Will I lose my hair after weight loss surgery? | There is a possibility that you may experience hair loss after weight loss surgery. This is called telogen effluvium, and it is a temporary condition that affects about 25% of people who have weight loss surgery. The hair loss usually starts 3-5 months after surgery and can last for up to 6 months.  There are a few reasons why hair loss can happen after weight loss surgery. One reason is that the body goes through a lot of stress during and after surgery. This stress can cause the hair follicles to go into a resting phase, which can lead to hair loss.  Another reason for hair loss after weight loss surgery is that people who have weight loss surgery often lose weight very quickly. This can lead to a deficiency in certain nutrients, such as iron, zinc, and vitamin B12. These nutrients are important for hair growth, so a deficiency can lead to hair loss.  The good news is that hair loss after weight loss surgery is usually temporary. Once the body has adjusted to the weight loss and the nutrient levels have normalized, the hair will start to grow back. In most cases, the hair will grow back completely.  There are a few things you can do to help prevent hair loss after weight loss surgery. These include:  * Getting enough protein in your diet. Protein is essential for hair growth, so it is important to make sure you are getting enough of it.  * Taking a multivitamin and mineral supplement. This can help to ensure that you are getting all of the nutrients your body needs for hair growth.  * Managing stress. Stress can contribute to hair loss, so it is important to find ways to manage stress, such as yoga, meditation, or spending time in nature.  If you are concerned about hair loss after weight loss surgery, talk to your doctor. They can help you to determine if you are at risk for hair loss and can offer advice on how to prevent or treat it. | 7/8/2023 | Sure. Here's a simpler explanation of hair loss after weight loss surgery:  * After weight loss surgery, your body goes through a lot of changes. This can cause your hair follicles to go into a resting phase, which means that they stop growing hair.  * This resting phase usually lasts for about 6 months. After that, your hair follicles will start to grow hair again.  * In most cases, the hair will grow back completely. However, in some cases, there may be some permanent hair loss.  There are a few things you can do to help prevent hair loss after weight loss surgery:  * Make sure you are getting enough protein in your diet. Protein is essential for hair growth.  * Take a multivitamin and mineral supplement. This can help to ensure that you are getting all of the nutrients your body needs for hair growth.  * Manage stress. Stress can contribute to hair loss. Find ways to relax and de-stress, such as yoga, meditation, or spending time in nature.  If you are concerned about hair loss after weight loss surgery, talk to your doctor. They can help you to determine if you are at risk for hair loss and can offer advice on how to prevent or treat it.  I hope this explanation is simpler to understand. Let me know if you have any other questions. |
| ASMBS | Will I need to have plastic surgery after weight loss? | Some patients may choose to have plastic surgery, but this depends on many factors. | Will I need to have plastic surgery after weight loss from bariatric surgery? | Whether or not you need plastic surgery after weight loss from bariatric surgery depends on a number of factors, including the amount of weight you have lost, your overall health, and your personal preferences.  In many cases, people who have lost a significant amount of weight through bariatric surgery are left with excess skin and tissue. This can be unsightly and can also lead to discomfort and pain. In these cases, plastic surgery can be a way to improve the appearance of the body and to improve function.  Some of the most common plastic surgery procedures performed after bariatric surgery include:  * Abdominoplasty (tummy tuck)  * Brachioplasty (arm lift)  * Mastopexy (breast lift)  * Lower body lift  * Buttock augmentation  * Facelift  It is important to talk to your doctor or a plastic surgeon about whether or not plastic surgery is right for you. They can help you assess your individual needs and goals and can recommend the best course of treatment.  Here are some of the things to consider when deciding whether or not to have plastic surgery after bariatric surgery:  * The amount of weight you have lost. If you have lost a significant amount of weight, you are more likely to need plastic surgery to remove excess skin and tissue.  * Your overall health. If you have any health conditions, such as diabetes or heart disease, you may need to wait until your health is more stable before having plastic surgery.  * Your personal preferences. Some people are not bothered by excess skin and tissue, while others find it very bothersome. If you are bothered by excess skin and tissue, plastic surgery can help you improve your appearance and self-confidence.  The best time to have plastic surgery after bariatric surgery is usually 12-18 months after the surgery. This gives your body time to heal and to reach your goal weight.  Plastic surgery is a personal decision. If you are considering plastic surgery after bariatric surgery, talk to your doctor or a plastic surgeon to learn more about the risks and benefits of the procedure. | 7/8/2023 | Sure. Here is a simpler explanation of whether or not you need plastic surgery after weight loss from bariatric surgery:  * If you have lost a significant amount of weight, you may have excess skin and tissue.  * This excess skin and tissue can be unsightly and can also lead to discomfort and pain.  * If you are bothered by the excess skin and tissue, you may want to consider plastic surgery.  * Plastic surgery can help you improve the appearance of your body and to improve function.  * The best time to have plastic surgery after bariatric surgery is usually 12-18 months after the surgery.  Here are some of the things to consider when deciding whether or not to have plastic surgery after bariatric surgery:  * How much weight have you lost?  * Do you have any health conditions?  * How bothered are you by the excess skin and tissue?  If you are considering plastic surgery after bariatric surgery, talk to your doctor or a plastic surgeon to learn more about the risks and benefits of the procedure.  In short, if you have lost a significant amount of weight and are bothered by the excess skin and tissue, plastic surgery can be a way to improve your appearance and function. The best time to have plastic surgery is usually 12-18 months after the bariatric surgery. |
| ASMBS | Do most people who have weight loss surgery regain their weight? | No. A small number of patients can regain their weight but the vast majority lose significant weight and keep this weight off.  More than 95% of patients will successfully lose half of their extra body weight or more after surgery for weight loss.  (1) Roux en Y gastric bypass patients, for example, on average lose approximately 70% of their extra body weight initially, and 2 years or more after surgery will regain approximately 5% of their extra body weight.  (2) Compared to these results, patients who attempt weight loss without surgery, on average, are able to lose little weight and may gain weight in the long-term. |  | No, most people who have weight loss surgery do not regain their weight. However, some people do regain some or all of the weight they lost, especially after 10 years or more.  According to a study published in the journal *Obesity Reviews*, an average of 56% of patients regain weight within 10 years of their surgery. However, the rate of weight regain varies depending on the type of surgery and the individual patient. For example, patients who have gastric bypass surgery are more likely to regain weight than those who have sleeve gastrectomy.  There are a number of factors that can contribute to weight regain after bariatric surgery, including:  * **Diet and lifestyle changes.** Patients who do not make significant changes to their diet and lifestyle after surgery are more likely to regain weight. This includes eating a healthy diet, exercising regularly, and maintaining a healthy weight.  * **Biological factors.** Some people may be more genetically predisposed to regain weight after surgery.  * **Psychological factors.** Stress, anxiety, and depression can all contribute to weight regain after surgery.  If you are considering bariatric surgery, it is important to talk to your doctor about the risks of weight regain. You should also be prepared to make significant changes to your diet and lifestyle after surgery. With careful planning and follow-up, you can greatly reduce your chances of regaining weight after bariatric surgery.  Here are some tips to help you prevent weight regain after bariatric surgery:  * **Follow your doctor's instructions carefully.** This includes taking your medications as prescribed and making the necessary dietary and lifestyle changes.  * **See your doctor regularly for follow-up appointments.** This will help your doctor monitor your weight and make sure you are on track.  * **Join a support group.** Talking to other people who have had bariatric surgery can be helpful in staying motivated and on track.  * **Don't give up.** Weight regain after bariatric surgery is common, but it is not inevitable. With hard work and dedication, you can maintain your weight loss and improve your health. | 7/8/2023 | Sure. Bariatric surgery is a major surgery that can help people lose a lot of weight. However, it is not a magic bullet. Some people do regain some or all of the weight they lost, especially after 10 years or more.  There are a number of things that can contribute to weight regain after bariatric surgery. These include:  * Not making significant changes to your diet and lifestyle after surgery.  * Having a genetic predisposition to regain weight.  * Experiencing stress, anxiety, or depression.  If you are considering bariatric surgery, it is important to talk to your doctor about the risks of weight regain. You should also be prepared to make significant changes to your diet and lifestyle after surgery. With careful planning and follow-up, you can greatly reduce your chances of regaining weight after bariatric surgery.  Here are some simple tips to help you prevent weight regain after bariatric surgery:  * Eat a healthy diet.  * Exercise regularly.  * Stay motivated and on track.  * Don't give up.  Bariatric surgery can be a life-saving treatment for obesity. However, it is important to remember that it is not a cure-all. With hard work and dedication, you can maintain your weight loss and improve your health.  Here is a table that summarizes the main points of my answer:  \| **Factor** \| **Impact on weight regain** \|  \|---\|---\|  \| Diet and lifestyle changes \| Significant changes can help prevent weight regain \|  \| Biological factors \| Some people may be more genetically predisposed to regain weight \|  \| Psychological factors \| Stress, anxiety, and depression can contribute to weight regain \|  \| Tips to prevent weight regain \| Follow your doctor's instructions, see your doctor regularly, join a support group, and don't give up \|  I hope this helps! |
| ASMBS | Can I go off some of my medications after weight loss surgery? | Many patients are able to stop using some medications. |  | Whether or not you can go off some of your medications after weight loss surgery depends on a number of factors, including the type of surgery you had, the medications you are taking, and your overall health.  In general, you should not go off any medications without first talking to your doctor. Bariatric surgery can affect the way your body absorbs medications, so you may need to adjust your dosages or switch to different medications. For example, if you take blood pressure medication, you may need to lower your dosage after surgery.  Some medications, such as nonsteroidal anti-inflammatory drugs (NSAIDs), can increase the risk of stomach ulcers after bariatric surgery. You should avoid these medications altogether if you have had gastric bypass surgery.  If you are taking any medications for chronic conditions, such as diabetes or high blood pressure, you will need to work with your doctor to monitor your condition and adjust your medications as needed.  In general, it is best to err on the side of caution and talk to your doctor before going off any medications after weight loss surgery. They can help you determine which medications are safe to stop taking and which ones you may need to continue taking.  Here are some additional things to keep in mind:  * You may need to take your medications more often after surgery, as your stomach will be smaller and you will be able to eat less at a time.  * You may need to crush or chew some medications that were once taken as a tablet.  * You may need to switch to liquid or extended-release forms of some medications.  It is important to follow your doctor's instructions carefully after weight loss surgery. This will help you ensure that you are taking the right medications in the right way and that you are getting the most out of your surgery. | 7/8/2023 | Sure. Here is a simpler explanation:  After weight loss surgery, your body may not absorb medications the same way it did before surgery. This means that you may need to change the way you take your medications or the dosages you take.  For example, if you take a blood pressure medication, you may need to take a lower dosage after surgery. Or, you may need to switch to a different type of blood pressure medication that is absorbed differently.  It is important to talk to your doctor before going off any medications after weight loss surgery. They can help you determine which medications are safe to stop taking and which ones you may need to continue taking.  Here are some general things to keep in mind:  * You may need to take your medications more often after surgery, as your stomach will be smaller and you will be able to eat less at a time.  * You may need to crush or chew some medications that were once taken as a tablet.  * You may need to switch to liquid or extended-release forms of some medications.  It is important to follow your doctor's instructions carefully after weight loss surgery. This will help you ensure that you are taking the right medications in the right way and that you are getting the most out of your surgery.  Here are some examples of medications that may need to be adjusted after weight loss surgery:  * Blood pressure medications  * Cholesterol medications  * Diabetes medications  * Antidepressants  * Pain medications  * Anti-inflammatory medications  If you are taking any of these medications, be sure to talk to your doctor about how your dosage may need to change after surgery. |
| ASMBS | Will I have to take vitamins after surgery? | You will need to take a multivitamin for life.  You may need higher doses of certain vitamins or minerals, especially Iron, Calcium, and Vitamin D. You will also need to have at least yearly lab checks. Insurance almost never pays for vitamin and mineral supplements but usually does pay for labs. You can pay for supplements out of a flex medical account. | Will I have to take vitamins after weight loss surgery? | Yes, you will likely need to take vitamins after weight loss surgery. The type and amount of vitamins you need will depend on the type of surgery you have, but most people will need to take a multivitamin, calcium with vitamin D, and vitamin B12.  There are a few reasons why you need to take vitamins after weight loss surgery. First, the surgery can make it harder for your body to absorb nutrients from food. Second, the surgery can also lead to some vitamin deficiencies, such as vitamin B12 deficiency.  Taking vitamins after weight loss surgery is important for your overall health. It can help you to prevent nutrient deficiencies, which can lead to health problems such as anemia, fatigue, and bone loss.  Your doctor or dietitian will be able to recommend the specific vitamins you need to take after your surgery. They will also be able to help you to find a good brand of vitamins and to determine the best way to take them.  Here are some of the most common vitamins that people need to take after weight loss surgery:  * **Multivitamin:** A multivitamin can help to ensure that you are getting all of the essential vitamins and minerals that your body needs.  * **Calcium with vitamin D:** Calcium is important for bone health, and vitamin D helps your body to absorb calcium.  * **Vitamin B12:** Vitamin B12 is important for nerve function and energy production.  * **Iron:** Iron is important for red blood cell production.  * **Folate:** Folate is important for cell growth and development.  It is important to take vitamins as directed by your doctor or dietitian. Taking too much of some vitamins can be harmful, so it is important to get the right amount.  I hope this helps! Let me know if you have any other questions. | 7/8/2023 | Sure. Here is a simpler explanation:  After weight loss surgery, your body may not be able to absorb all the vitamins and minerals it needs from food. This is because the surgery can change the way your body digests and absorbs nutrients.  To make sure you get enough vitamins and minerals, you will need to take supplements. The most common supplements people take after weight loss surgery are a multivitamin, calcium with vitamin D, and vitamin B12.  Your doctor or dietitian can help you figure out which supplements you need and how much to take. They can also help you find a good brand of supplements.  It is important to take supplements as directed by your doctor or dietitian. Taking too much of some vitamins can be harmful, so it is important to get the right amount.  Here is a table that summarizes the most common vitamins that people need to take after weight loss surgery:  \| Vitamin \| What it does \| Why you need it after weight loss surgery \|  \|---\|---\|---\|  \| Multivitamin \| Contains a variety of vitamins and minerals \| Helps ensure you are getting all the nutrients you need \|  \| Calcium with vitamin D \| Important for bone health \| Helps prevent bone loss \|  \| Vitamin B12 \| Important for nerve function and energy production \| Helps prevent nerve damage and fatigue \|  \| Iron \| Important for red blood cell production \| Helps prevent anemia \|  \| Folate \| Important for cell growth and development \| Helps prevent birth defects \|  I hope this helps! Let me know if you have any other questions. |
| ASMBS | Do weight loss surgery patients have serious health problems caused by vitamin deficiency? | As long as patients take their appropriate vitamins, it is rare to have health problems from vitamin deficiency.  Weight loss surgery can lead to reduced amounts of vitamins and minerals because people eat less and may absorb less in the intestines. Weight loss surgeries have different effects on vitamins and minerals based on how much change in absorption they cause. Deficiencies in vitamins, minerals, and protein can negatively affect health causing:  - fatigue (feeling tired)  - anemia (low levels of red blood cells that carry oxygen through your body)  - bone and muscle loss  - impaired night vision  - low immunity (your body’s ability to stay healthy)  - loss of nerve function (can affect your senses such as touch, taste, and smell)  - mental function deficits (changes how clearly you think)  Fortunately, nutrient deficiencies following surgery can be easily avoided with a good diet and the use of supplements including vitamins, minerals, and sometimes protein supplements. Before and after surgery, patients are advised of the appropriate diet and vitamin supplement needed. Most weight loss surgery programs also require patients to have their vitamins and minerals checked on a regular basis after surgery. Nutrient deficiencies and any associated health problems are preventable with monitoring and patients following diet and supplement (vitamin and mineral) recommendations. Health problems due to deficiencies usually occur in patients who do not regularly follow-up to monitor healthy vitamin and mineral levels. Be sure you stay in touch with your weight loss surgery team for best results! |  | Yes, weight loss surgery patients are at increased risk for vitamin deficiency, which can lead to serious health problems. The most common vitamin deficiencies after bariatric surgery are:  * **Vitamin B12**. Vitamin B12 is essential for the production of red blood cells and nerve cells. A deficiency can lead to anemia, fatigue, and numbness or tingling in the hands and feet.  * **Vitamin D**. Vitamin D is important for bone health. A deficiency can lead to osteoporosis, which is a condition characterized by weak bones that are more likely to break.  * **Folate**. Folate is essential for cell growth and development. A deficiency can lead to anemia and birth defects.  * **Iron**. Iron is essential for the production of red blood cells. A deficiency can lead to anemia, fatigue, and shortness of breath.  * **Calcium**. Calcium is important for bone health. A deficiency can lead to osteoporosis and osteomalacia, a condition characterized by soft bones.  If left untreated, vitamin deficiency can lead to serious health problems, such as:  * **Neurological problems**, such as numbness, tingling, and weakness  * **Anemia**, which can cause fatigue, shortness of breath, and pale skin  * **Osteoporosis**, which can lead to bone fractures  * **Birth defects**, if the mother is deficient in folate during pregnancy  It is important for weight loss surgery patients to be aware of the risk of vitamin deficiency and to take steps to prevent it. This includes taking a daily multivitamin and mineral supplement, eating a healthy diet, and getting regular blood tests to monitor vitamin levels.  Here are some tips for preventing vitamin deficiency after weight loss surgery:  * Take a daily multivitamin and mineral supplement that is specifically designed for bariatric surgery patients.  * Eat a healthy diet that includes a variety of fruits, vegetables, and whole grains.  * Get regular blood tests to monitor vitamin levels.  * Talk to your doctor about any concerns you have about vitamin deficiency.  By following these tips, you can help to reduce your risk of vitamin deficiency and improve your overall health after weight loss surgery. | 7/8/2023 | Sure. Weight loss surgery can change the way your body absorbs vitamins. This can lead to vitamin deficiencies, which can cause serious health problems. The most common vitamin deficiencies after weight loss surgery are vitamin B12, vitamin D, folate, iron, and calcium.  To prevent vitamin deficiency, it is important to take a daily multivitamin and mineral supplement, eat a healthy diet, and get regular blood tests to monitor vitamin levels.  Here are some simple tips to remember:  * Take a daily multivitamin and mineral supplement that is specifically designed for bariatric surgery patients.  * Eat a variety of fruits, vegetables, and whole grains.  * Get regular blood tests to monitor vitamin levels.  * Talk to your doctor about any concerns you have about vitamin deficiency.  By following these tips, you can help to reduce your risk of vitamin deficiency and improve your overall health after weight loss surgery.  Here is an analogy that might help you understand this better. Imagine that you have a new car. The car comes with a manual that tells you how to operate it and maintain it. If you don't follow the instructions in the manual, your car might break down.  Weight loss surgery is like a new car. Your body needs certain vitamins and minerals to function properly. If you don't get enough of these vitamins and minerals, your body might break down. The multivitamin and mineral supplement is like the manual for your car. It tells you how much of each vitamin and mineral you need to stay healthy.  Eating a healthy diet is like taking care of your car. If you feed your car the wrong kind of fuel, it might not run properly. The same is true for your body. If you don't eat a healthy diet, you might not get enough of the vitamins and minerals you need.  Regular blood tests are like taking your car to the mechanic for an oil change. The blood tests will tell you if you have any vitamin deficiencies. If you do have any deficiencies, your doctor can help you figure out how to correct them.  By following these simple tips, you can help to keep your body running smoothly and prevent vitamin deficiency. |
| ASMBS | Is obesity caused by food addiction, similar to alcoholism or drug dependency? | “Food addiction” as a cause of obesity is extremely rare.  Although some people with obesity have eating disorders, such as binge eating disorder syndrome, most people have obesity caused by many factors. When treating addiction, such as alcohol and drugs, one of the first steps is to stop using drugs or alcohol. This does not work with obesity as we need to eat to live. Also, there may be other issues causing a person’s weight gain. Weight gain generally occurs when the amount of food eaten is greater than the number of calories burned. There are other conditions, however, that affect weight gain that do not involve too much eating or a less active lifestyle including:  - Poor sleeping habits  - Eating foods that may increase body fat (sugar, high fructose corn syrup, trans fat, processed meats and processed grains)  - Low intake of fat-fighting foods (fruits, vegetables, legumes, nuts, seeds, quality protein)  - Stress and mental distress  - Many types of medications  - Pollutants  Obesity also leads to more obesity, which is one of the reasons why the disease is considered progressive. Weight gain causes a number of signals (hormones) in the body that increase the risk for even greater weight gain and obesity. To make matters worse, obesity affects certain body functions that control appetite and hunger in a manner that can cause an increase in the amount of food eaten at any given meal and the desire to eat more often. There are many causes for obesity and the disease of obesity is far more than just an ‘addiction’ toward food. The treatment of obesity only as an addiction may help for a very small percentage of individuals whose only underlying cause for obesity is excessive and addictive eating, but would be unlikely to benefit most people, particularly those individuals affected by severe obesity. |  | The concept of food addiction is a complex one, and there is no consensus among experts on whether it is truly a form of addiction in the same way as alcoholism or drug dependency. However, there is evidence that suggests that some people with obesity may experience compulsive overeating that is similar to addictive behavior.  For example, studies have shown that people with food addiction may experience similar brain changes to those with substance addiction. In particular, the reward centers of the brain are activated in response to food cues in people with food addiction, just as they are in people with substance addiction. Additionally, people with food addiction may have difficulty controlling their eating behavior, even when they are aware that their eating is harmful to their health.  It is important to note that not all people with obesity are food addicts. There are many other factors that can contribute to obesity, such as genetics, environment, and lifestyle choices. However, for some people, food addiction may be a significant factor that contributes to their weight gain.  If you are concerned that you or someone you know may have food addiction, it is important to talk to a doctor or mental health professional. They can help you assess your risk for food addiction and develop a treatment plan.  Here are some of the signs and symptoms of food addiction:  * Compulsive overeating  * Difficulty controlling your eating behavior  * Eating in response to stress or negative emotions  * Feeling guilty or ashamed about your eating habits  * Eating large amounts of food even when you are not hungry  * Hiding your eating habits from others  * Gaining weight despite trying to lose weight  If you experience any of these symptoms, it is important to seek help. Food addiction is a serious condition, but it can be treated. With the right help, you can overcome your addiction and achieve a healthy weight. | 7/8/2023 | Sure. Food addiction is a condition where people compulsively eat unhealthy foods, even when they know it's bad for them. This is similar to drug addiction, where people compulsively use drugs, even when they know it's bad for them.  There are some similarities between food addiction and drug addiction. For example, both food addiction and drug addiction can activate the reward centers of the brain. This means that both food and drugs can give people a feeling of pleasure or satisfaction.  However, there are also some differences between food addiction and drug addiction. For example, food is not technically a drug. Additionally, food addiction is not always caused by the same factors as drug addiction. For example, food addiction can be caused by genetics, environment, or lifestyle choices.  If you think you might have food addiction, it's important to talk to a doctor or mental health professional. They can help you assess your risk for food addiction and develop a treatment plan.  Here are some of the things you can do to help manage food addiction:  * **Identify your triggers.** What are the things that make you want to eat unhealthy foods? Once you know your triggers, you can start to avoid them or develop coping mechanisms.  * **Make healthy changes to your diet.** This includes eating more fruits, vegetables, and whole grains. It also means reducing your intake of processed foods, sugary drinks, and unhealthy fats.  * **Get regular exercise.** Exercise can help you manage your weight and reduce your stress levels.  * **Talk to a therapist or counselor.** A therapist can help you understand your food addiction and develop coping mechanisms.  Remember, food addiction is a serious condition, but it can be treated. With the right help, you can overcome your addiction and achieve a healthy weight. |
| ASMBS | Does weight loss surgery increase the risk for alcohol abuse? | There is a small group of patients after weight loss surgery who have a problem with alcohol abuse. Most of those people, but not all, already had experiences with alcohol abuse before surgery.  Recommended precautions after weight loss surgery:  - Avoid alcoholic beverages during the rapid weight loss period, and then drink only rarely  - Be aware that even small amounts of alcohol can cause intoxication (‘being drunk’)  - Avoid driving or operating heavy equipment after drinking any alcohol  - Remember that you may feel less intoxicated than you are, and will not be a reliable judge of whether it is safe for you to drive after drinking.  - Seek help if drinking becomes a problem  If you feel drinking alcohol may be an issue for you after surgery, please contact your primary care doctor or surgeon and discuss this further. They will be able to help you identify resources available to address any alcohol-related issues.  It has been found in scientific research that a small percentage of weight loss surgery patients report having problems with alcohol after surgery (7-10%). (6) Many who abuse alcohol after surgery had problems with alcohol abuse at some time prior to surgery, but occasionally alcohol problems can arise in patients who never had such issues before their surgery. Alcohol sensitivity is increased after weight loss surgery so that the effects of alcohol are felt with fewer drinks than before surgery. Studies also find that with certain weight loss procedures (such as the gastric bypass or sleeve gastrectomy), drinking an alcoholic beverage increases blood alcohol to levels that are considerably higher than before surgery or in comparison to the alcohol levels of individuals who have not had a weight loss procedure. In addition, studies have found that alcohol takes much longer to clear out of a person’s system if they have had weight loss surgery, which can pose safety and legal hazards for driving. Furthermore, it has been found that some patients may feel as though they are sober (‘not drunk’) even when they are over the legal blood alcohol limit for driving. For all of these reasons, weight loss surgery patients are advised to take precautions regarding alcohol. |  | Yes, weight loss surgery can increase the risk for alcohol abuse. There are a number of reasons for this, including:  * **Changes in the way alcohol is processed by the body.** After weight loss surgery, the stomach is smaller and the small intestine is bypassed. This means that alcohol enters the bloodstream more quickly and can reach a higher concentration. This can lead to intoxication and other problems.  * **Changes in emotional and psychological factors.** Weight loss surgery can be a stressful and life-changing experience. Some people may turn to alcohol to cope with these changes. Others may find that they enjoy the feeling of being drunk more now that they are smaller.  * **Social factors.** People who have had weight loss surgery may find that they are more accepted socially. This can lead to increased drinking, especially in social situations.  The risk of alcohol abuse after weight loss surgery is highest in the first year after surgery. However, it can occur at any time. If you are considering weight loss surgery, it is important to talk to your doctor about the risks of alcohol abuse. You should also be aware of the signs and symptoms of alcohol abuse, and seek help if you or someone you know is struggling with this problem.  Here are some of the signs and symptoms of alcohol abuse to watch out for:  * **Increased drinking.** You may start drinking more often or in larger quantities than you used to.  * **Trouble controlling your drinking.** You may find it difficult to stop drinking once you start.  * **Withdrawal symptoms.** If you stop drinking, you may experience symptoms such as anxiety, tremors, and sweating.  * **Problems at work or school.** Your drinking may start to interfere with your work or studies.  * **Relationship problems.** Your drinking may start to strain your relationships with family and friends.  If you are concerned that you or someone you know may be struggling with alcohol abuse, please seek help. There are many resources available to help people with alcohol problems. You can talk to your doctor, a therapist, or a support group. There are also many online resources available. | 7/8/2023 | Sure. Weight loss surgery can make it easier for people to get drunk because the stomach is smaller and the small intestine is bypassed. This means that alcohol enters the bloodstream more quickly and can reach a higher concentration. This can lead to problems like intoxication, low blood sugar, and liver damage.  Some people who have weight loss surgery may also start drinking more because they are stressed or because they feel like they can now drink more without gaining weight. This can lead to alcohol abuse.  If you are considering weight loss surgery, it is important to talk to your doctor about the risks of alcohol abuse. You should also be aware of the signs and symptoms of alcohol abuse, and seek help if you or someone you know is struggling with this problem.  Here are some of the signs and symptoms of alcohol abuse to watch out for:  * You drink more often or in larger quantities than you used to.  * You find it difficult to stop drinking once you start.  * You experience withdrawal symptoms if you stop drinking.  * Your drinking starts to interfere with your work or studies.  * Your drinking starts to strain your relationships with family and friends.  If you are concerned that you or someone you know may be struggling with alcohol abuse, please seek help. There are many resources available to help people with alcohol problems. You can talk to your doctor, a therapist, or a support group. There are also many online resources available. |
| ASMBS | Does weight loss surgery increase the risk for suicide? | After surgery, most patients have improved mental health but there may be a small increase in the risk of suicide. This is why patients are required to have a mental health screening before surgery.  People affected by severe obesity who are seeking weight loss surgery are more likely to suffer from depression or anxiety and to have lower self-esteem and overall quality of life than someone who is normal weight. Weight loss surgery results in significant improvement in mental health for the majority of patients. However, studies have found a small but significant increase in suicide following weight loss surgery. (7, 8) In some cases, these may have been patients with undiagnosed mental disorders, and in others, patients may have experienced major life stressors that can increase the risk for suicide, but in rare cases, the suicides are unexplained. For this reason, comprehensive bariatric programs require psychological evaluations prior to weight loss surgery. Many programs have therapists available for patient counseling after surgery. In addition, if a patient sees a psychologist or psychiatrist before surgery, they should continue seeing them after surgery. |  | Talk to someone now  Help is available  988 Suicide and Crisis Lifeline  Hours: Available 24 hours. Languages: English, Spanish. Learn more  Call or SMS:  988  From Google Search | 7/8/2023 | Sure. Suicide is a serious issue, and if you or someone you know is thinking about suicide, please reach out for help. There are many resources available, including the National Suicide Prevention Lifeline (988) and the Crisis Text Line (text HOME to 741741).  To answer your question, research has shown that there is an increased risk of suicide after weight loss surgery. This is likely due to a number of factors, including:  * The stress of major surgery  * Changes in hormones and brain chemistry  * Depression or other mental health problems  * Feeling isolated or alone after surgery  If you are considering weight loss surgery, it is important to talk to your doctor about the risks of suicide. They can help you to assess your individual risk factors and make sure that you have the support you need before and after surgery.  Here are some additional resources that you may find helpful:  * National Suicide Prevention Lifeline: https://suicidepreventionlifeline.org/  * Crisis Text Line: https://www.crisistextline.org/  * The Trevor Project: https://www.thetrevorproject.org/  * NAMI: https://www.nami.org/  * MentalHealth.gov: https://www.mentalhealth.gov/ |
| ASMBS | Is the chance of dying from weight loss surgery more than the chance of dying from obesity? | No. Weight loss surgery is very safe and decreases the chance of dying from obesity. In fact, weight loss surgery is safer than removing the gallbladder or having a knee replacement.  A recent study of 209,116 patients found the risk of death from weight loss surgery was 0.16%, or approximately 1 in 600. (4) This rate is considerably less than most other surgeries, including gallbladder and hip replacement surgery. Large studies find that the risk of death from any cause is considerably less for patients after weight loss surgery than for those who have severe obesity and have never had the surgery. Patients who have weight loss surgery have a reduction in their risk of death by 40%. Death related to diabetes is reduced by more than 90% and from heart disease by more than 50% for patients after weight loss surgery. (3) The benefits of weight loss surgery far outweigh the risks. As with any serious surgical operation, the decision to have weight loss surgery should be discussed with your surgeon, family members, and loved ones. |  | The chance of dying from weight loss surgery is much lower than the chance of dying from obesity. According to the National Institute of Diabetes and Digestive and Kidney Diseases, obesity is the fifth leading cause of death in the United States. The risk of death from obesity-related complications increases with the severity of obesity. For example, people with a BMI of 40 or higher have a 20% increased risk of death compared to people with a normal BMI.  The risk of death from weight loss surgery is very low. According to the American Society for Metabolic and Bariatric Surgery, the risk of death from weight loss surgery is less than 1%. The most common complications of weight loss surgery are infection, blood clots, and hernia. These complications are usually minor and can be treated.  If you are considering weight loss surgery, it is important to talk to your doctor about the risks and benefits of the procedure. You should also make sure that you are a good candidate for surgery. Some people who are not good candidates for surgery include those with certain medical conditions, such as heart disease or diabetes.  Here is a table that summarizes the risks of death from obesity and weight loss surgery:  \| Risk Factor \| Obesity \| Weight Loss Surgery \|  \|---\|---\|---\|  \| Risk of Death \| 20% increased risk for people with a BMI of 40 or higher \| Less than 1% \|  \| Most Common Complications \| Infection, blood clots, hernia \| Minor complications that can be treated \|  \| Good Candidates for Surgery \| People who are obese and have tried other weight loss methods without success \| People who are a good fit for the procedure and have realistic expectations \|  I hope this information is helpful. Please let me know if you have any other questions. | 7/8/2023 | Sure. Here is a simpler explanation of the risks of death from obesity and weight loss surgery:  * Obesity is a very serious condition that can lead to death. People who are obese are more likely to die from heart disease, stroke, diabetes, and other health problems.  * Weight loss surgery is a safe procedure that can help people lose weight and improve their health. The risk of death from weight loss surgery is very low, but it is still possible.  * If you are considering weight loss surgery, it is important to talk to your doctor about the risks and benefits of the procedure. You should also make sure that you are a good candidate for surgery.  Here is a table that summarizes the risks of death from obesity and weight loss surgery in simpler terms:  \| Risk Factor \| Obesity \| Weight Loss Surgery \|  \|---\|---\|---\|  \| Risk of Death \| High \| Low \|  \| Most Common Complications \| Serious \| Minor \|  \| Good Candidates for Surgery \| People who are obese and have tried other weight loss methods without success \| People who are a good fit for the procedure and have realistic expectations \|  I hope this explanation is helpful. Please let me know if you have any other questions. |
