# Supplementary material for: Large language models and bariatric surgery patient education: a comparative readability analysis of GPT-3.5, GPT-4, Bard, and online institutional resources
Source: Surg Endosc. 2024 Mar 12;38(5):2522–32. doi: 10.1007/s00464-024-10720-2 (PMC11078810; doi:10.1007/s00464-024-10720-2)
Supplement: Supplementary file 5 — Supplementary file5 (DOCX 8 KB) [file 464_2024_10720_MOESM5_ESM.docx]

**Supplementary Table 5**. Institutional Readability

|  | **American Society for Metabolic and Bariatric Surgery** | **Institution 1** | **Institution 2** | **Institution 3** | **Institution 4** | **Institution 5** |
| --- | --- | --- | --- | --- | --- | --- |
| **Flesch Reading Ease Formula** | 56.8 (15.7) | 43.0 (24.9) | 39.7 (16.9) | 53.7 (18.2) | 40.7 (12.6) | 45.3 (22.1) |
| **Gunning Fog Scale** | 12.4 (4.0) | 16.3 (6.1) | 15.5 (4.1) | 12.5 (3.0) | 16.4 (4.2) | 15.1 (4.7) |
| **Flesch-Kincaid Grade Level** | 9.6 (3.5) | 12.0 (5.2) | 12.0 (3.4) | 9.5 (3.1) | 12.7 (3.9) | 12.0 (3.9) |
| **Coleman-Liau Index** | 10.2 (2.1) | 11.3 (3.5) | 12.7 (3.6) | 10.4 (2.7) | 11.5 (1.9) | 11.0 (4.9) |
| **SMOG Index** | 8.9 (2.8) | 11.5 (4.1) | 11.4 (2.7) | 9.2 (2.2) | 12.0 (3.0) | 11.3 (2.9) |
| **Automated Readability Index** | 9.5 (4.3) | 11.3 (5.5) | 11.6 (4.6) | 8.6 (3.3) | 12.4 (4.7) | 11.9 (5.6) |
| **Linsear Write Formula** | 10.6 (5.0) | 13.5 (7.7) | 12.4 (5.5) | 9.3 (2.9) | 14.4 (7.1) | 14.1 (4.8) |

Data is presented as Mean (Standard Deviation)
