# Supplementary material for: Large language models and bariatric surgery patient education: a comparative readability analysis of GPT-3.5, GPT-4, Bard, and online institutional resources
Source: Surg Endosc. 2024 Mar 12;38(5):2522–32. doi: 10.1007/s00464-024-10720-2 (PMC11078810; doi:10.1007/s00464-024-10720-2)
Supplement: Supplementary file 6 — Supplementary file6 (DOCX 9 KB) [file 464_2024_10720_MOESM6_ESM.docx]

**Supplementary Table 6**. Comparison of Readability Scores for Institutional and LLM Responses to Bariatric Surgery FAQs

|  | **Flesch Reading Ease Formula** | | **Gunning Fog Scale** | | **Flesch-Kincaid Grade Level** | | **Coleman-Liau Index** | | **SMOG Index** | | **Automated Readability Index** | | **Linsear Write Formula** | |
| --- | --- | --- | --- | --- | --- | --- | --- | --- | --- | --- | --- | --- | --- | --- |
|  | Mean (SD) | T-Test | Mean (SD) | T-Test | Mean (SD) | T-Test | Mean (SD) | T-Test | Mean (SD) | T-Test | Mean (SD) | T-Test | Mean (SD) | T-Test |
| Institution | 48.1 (19.0) |  | 14.2 (4.4) |  | 11.0 (3.8) |  | 11.1 (3.3) |  | 10.4 (3.0) |  | 10.6 (4.7) |  | 12.0 (5.5) |  |
| GPT-3.5 Initial | **31.4 (11.4)** | **P<0.001*** | **18.1 (2.7)** | **P<0.001*** | **13.6 (2.3)** | **P<0.001*** | **14.2 (1.8)** | **P<0.001*** | **13.0 (1.8)** | **P<0.001*** | **13.8 (2.7)** | **P<0.001*** | **14.7 (3.4)** | **P=0.001*** |
| GPT-3.5 Simplified | 53.2 (10.7) | P=0.059 | 13.4 (2.6) | P=0.177 | **9.6 (2.0)** | **P=0.012*** | 11.6 (1.6) | P=0.266 | 9.9 (1.7) | P=0.266 | 9.7 (2.3) | P=0.147 | **10.1 (2.8)** | **P=0.016*** |
| GPT-4 Initial | **42.7 (9.7)** | **P=0.043*** | **15.6 (2.6)** | **P=0.033*** | 11.8 (2.0) | P=0.142 | **12.4 (1.6)** | **P=0.005*** | **11.5 (1.7)** | **P=0.013*** | 11.7 (2.4) | P=0.105 | 12.8 (3.3) | P=0.265 |
| GPT-4 Simplified | 74.0 (7.2) | **P<0.001*** | **9.4 (1.9)** | **P<0.001*** | **6.2 (1.5)** | **P<0.001*** | **8.0 (1.4)** | **P<0.001*** | **7.0 (1.2)** | **P<0.001*** | **5.8 (1.9)** | **P<0.001*** | **7.1 (2.1)** | **P<0.001*** |
| Bard Initial | **56.3 (11.6)** | **P=0.003*** | 13.3 (2.7) | P=0.160 | **9.8 (2.6)** | **P=0.038*** | **9.5 (1.6)** | **P<0.001*** | 9.9 (2.0) | P=0.285 | **9.2 (2.9)** | **P=0.036*** | 11.4 (4.1) | P=0.543 |
| Bard Simplified | **62.8 (11.1)** | **P<0.001*** | **12.1 (2.6)** | **P=0.001*** | **8.5 (2.4)** | **P<0.001*** | **8.8 (1.4)** | **P<0.001*** | **9.0 (2.0)** | **P=0.002*** | **7.8 (2.6)** | **P<0.001*** | **9.9 (3.5)** | **P=0.011*** |

**FAQ**: frequently asked question; **LLM**: large language model; **SD**: standard deviation

*p<0.05 when comparing scores with institutions
