# Supplementary material for: Large language models and bariatric surgery patient education: a comparative readability analysis of GPT-3.5, GPT-4, Bard, and online institutional resources
Source: Surg Endosc. 2024 Mar 12;38(5):2522–32. doi: 10.1007/s00464-024-10720-2 (PMC11078810; doi:10.1007/s00464-024-10720-2)
Supplement: Supplementary file 7 — Supplementary file7 (DOCX 16 KB) [file 464_2024_10720_MOESM7_ESM.docx]

**Supplementary Table 7**. Comparison of Readability Scores Between Various LLM Responses to Bariatric Surgery Frequently Asked Questions

| Readability Instrument | GPT-3.5 Initial vs. GPT-3.5 Simplified | | GPT-4 Initial vs. GPT-4 Simplified | | Bard Initial vs. Bard Simplified | | GPT-3.5 Initial vs. GPT-4 Initial | | GPT-3.5 Simplified vs. GPT-4 Simplified | | GPT-4 Initial vs. Bard Initial | | GPT-4 Simplified vs. Bard Simplified | |
| --- | --- | --- | --- | --- | --- | --- | --- | --- | --- | --- | --- | --- | --- | --- |
|  | Mean (SD) | T-Test* | Mean (SD) | T-Test* | Mean (SD) | T-Test* | Mean (SD) | T-Test* | Mean (SD) | T-Test* | Mean (SD) | T-Test* | Mean (SD) | T-Test* |
| Flesch Reading Ease Formula | **31.4 (11.4) vs. 53.2 (10.7)** | **P<0.001*** | **42.7 (9.7) vs. 74.0 (7.2)** | **P<0.001*** | **56.3 (11.6) vs. 62.8 (11.1)** | **P=0.001*** | **31.4 (11.4) vs. 42.7 (9.7)** | **P<0.001*** | **53.2 (10.7) vs. 74.0 (7.2)** | **P<0.001*** | **42.7 (9.7) vs. 56.3 (11.6)** | **P<0.001*** | **74.0 (7.2) vs. 62.8 (11.1)** | **P<0.001*** |
| Gunning Fog Scale | **18.1 (2.7) vs. 13.4 (2.6)** | **P<0.001*** | **15.6 (2.6) vs. 9.4 (1.9)** | **P<0.001*** | **13.3 (2.7) vs. 12.1 (2.6)** | **P=0.012*** | **18.1 (2.7) vs. 15.6 (2.6)** | **P<0.001*** | **13.4 (2.6) vs. 9.4 (1.9)** | **P<0.001*** | **15.6 (2.6) vs. 13.3 (2.7)** | **P<0.001*** | **9.4 (1.9) vs. 12.1 (2.6)** | **P<0.001*** |
| Flesch-Kincaid Grade Level | **13.6 (2.3) vs. 9.6 (2.0)** | **P<0.001*** | **11.8 (2.0) vs. 6.2 (1.5)** | **P<0.001*** | **9.8 (2.6) vs. 8.5 (2.4)** | **P=0.004*** | **13.6 (2.3) vs. 11.8 (2.0)** | **P<0.001*** | **9.6 (2.0) vs. 6.2 (1.5)** | **P<0.001*** | **11.8 (2.0) vs. 9.8 (2.6)** | **P<0.001*** | **6.2 (1.5) vs. 8.5 (2.4)** | **P<0.001*** |
| Coleman-Liau Index | **14.2 (1.8) vs. 11.6 (1.6)** | **P<0.001*** | **12.4 (1.6) vs. 8.0 (1.4)** | **P<0.001*** | **9.5 (1.6) vs. 8.8 (1.4)** | **P=0.008*** | **14.2 (1.8) vs. 12.4 (1.6)** | **P<0.001*** | **11.6 (1.6) vs. 8.0 (1.4)** | **P<0.001*** | **12.4 (1.6) vs. 9.5 (1.6)** | **P<0.001*** | **8.0 (1.4) vs. 8.8 (1.4)** | **P=0.001*** |
| SMOG Index | **13.0 (1.8) vs. 9.9 (1.7)** | **P<0.001*** | **11.5 (1.7) vs. 7.0 (1.2)** | **P<0.001*** | **9.9 (2.0) vs. 9.0 (2.0)** | **P=0.011*** | **13.0 (1.8) vs. 11.5 (1.7)** | **P<0.001*** | **9.9 (1.7) vs. 7.0 (1.2)** | **P<0.001*** | **11.5 (1.7) vs. 9.9 (2.0)** | **P<0.001*** | **7.0 (1.2) vs. 9.0 (2.0)** | **P<0.001*** |
| Automated Readability Index | **13.8 (2.7) vs. 9.7 (2.3)** | **P<0.001*** | **11.7 (2.4) vs. 5.8 (1.9)** | **P<0.001*** | **9.2 (2.9) vs. 7.8 (2.6)** | **P=0.006*** | **13.8 (2.7) vs. 11.7 (2.4)** | **P<0.001*** | **9.7 (2.3) vs. 5.8 (1.9)** | **P<0.001*** | **11.7 (2.4) vs. 9.2 (2.9)** | **P<0.001*** | **5.8 (1.9) vs. 7.8 (2.6)** | **P<0.001*** |
| Linsear Write Formula | **14.7 (3.4) vs. 10.1 (2.8)** | **P<0.001*** | **12.8 (3.3) vs. 7.1 (2.1)** | **P<0.001*** | **11.4 (4.1) vs. 9.9 (3.5)** | **P=0.022*** | **14.7 (3.4) vs. 12.8 (3.3)** | **P=0.002*** | **10.1 (2.8) vs. 7.1 (2.1)** | **P<0.001*** | **12.8 (3.3) vs. 11.4 (4.1)** | **P=0.033*** | **7.1 (2.1) vs. 9.9 (3.5)** | **P<0.001*** |

**LLM**: large language model; **SD**: standard deviation

*****p<0.05
