# Supplementary material for: Large language models and bariatric surgery patient education: a comparative readability analysis of GPT-3.5, GPT-4, Bard, and online institutional resources
Source: Surg Endosc. 2024 Mar 12;38(5):2522–32. doi: 10.1007/s00464-024-10720-2 (PMC11078810; doi:10.1007/s00464-024-10720-2)
Supplement: Supplementary file 8 — Supplementary file8 (DOCX 7 KB) [file 464_2024_10720_MOESM8_ESM.docx]

**Supplementary Table 8**. Comparison of Accuracy and Comprehensiveness Between Initial and Simplified Responses from GPT-3.5, GPT-4, and Bard

|  | **Accuracy** | | | **Comprehensiveness** | | |
| --- | --- | --- | --- | --- | --- | --- |
|  | The Simplified Response is more accurate than the Initial Response. | The Simplified Response is equal in accuracy to the Initial Response. | The Simplified Response is less accurate than the Initial Response. | The Simplified Response is more comprehensive than the Initial Response. | The Simplified Response is equal in comprehensiveness to the Initial Response. | The Simplified Response is less comprehensive than the Initial Response. |
| GPT-3.5 | 0% (0/66) | 100% (66/66) | 0% (0/66) | 0% (0/66) | 92.4% (61/66) | 7.6% (5/66) |
| GPT-4 | 0% (0/66) | 100% (66/66) | 0% (0/66) | 0% (0/66) | 92.4% (61/66) | 7.6% (5/66) |
| Bard | 1.5% (1/66) | 97.0% (64/66) | 1.5% (1/66) | 1.5% (1/66) | 63.6% (42/66) | 34.8% (23/66) |

Answers provided as percentage of all 66 FAQs prompted to large language models, raw data in parentheses
